# BrainSignals Revisited: Simplifying a Computational Model of Cerebral Physiology

**DOI:** 10.1371/journal.pone.0126695

**Published:** 2015-05-11

**Authors:** Matthew Caldwell, Tharindi Hapuarachchi, David Highton, Clare Elwell, Martin Smith, Ilias Tachtsidis

**Affiliations:** 1 Department of Medical Physics and Biomedical Engineering, University College London, London, UK; 2 Centre for Mathematics and Physics in the Life Sciences and Experimental Biology, University College London, London, UK; 3 Neurocritical Care Unit, University College Hospitals, London, UK; Georgia State University, UNITED STATES

## Abstract

Multimodal monitoring of brain state is important both for the investigation of healthy cerebral physiology and to inform clinical decision making in conditions of injury and disease. Near-infrared spectroscopy is an instrument modality that allows non-invasive measurement of several physiological variables of clinical interest, notably haemoglobin oxygenation and the redox state of the metabolic enzyme cytochrome c oxidase. Interpreting such measurements requires the integration of multiple signals from different sources to try to understand the physiological states giving rise to them. We have previously published several computational models to assist with such interpretation. Like many models in the realm of Systems Biology, these are complex and dependent on many parameters that can be difficult or impossible to measure precisely. Taking one such model, BrainSignals, as a starting point, we have developed several variant models in which specific regions of complexity are substituted with much simpler linear approximations. We demonstrate that model behaviour can be maintained whilst achieving a significant reduction in complexity, provided that the linearity assumptions hold. The simplified models have been tested for applicability with simulated data and experimental data from healthy adults undergoing a hypercapnia challenge, but relevance to different physiological and pathophysiological conditions will require specific testing. In conditions where the simplified models are applicable, their greater efficiency has potential to allow their use at the bedside to help interpret clinical data in near real-time.

## Introduction

The cells of the brain make very high energy demands relative to most other body tissues. These demands must be continuously met by oxidative metabolism, usually of glucose. Even a brief interruption in oxygen supply can have adverse consequences, including brain damage and death. Oxygen availability is normally maintained by a complex and robust system of haemodynamic regulation, which adjusts the cerebral blood flow (CBF) in response to variations in both supply—e.g. arterial pressure and oxygen saturation—and demand—notably energy consumption due to neuronal activity [1, 2].

These regulatory mechanisms are frequently impaired in disease states. Because of the potential harm that can result from oxygen and metabolic substrate deprivation, monitoring of cerebral oxygenation and metabolism has been proposed in clinical settings, in particular during the neurocritical care management of acute brain injury [3–9]. A range of multimodal neuromonitoring is available [9–11] including invasive probes such as brain tissue oxygen tension and microdialysis [12–14] and imaging techniques such as positron emission tomography [13, 15] and magnetic resonance spectroscopy (MRS) [16–19]. However, the latter are not usually available at the bedside.

Near-infrared spectroscopy (NIRS), which measures changes in the absorption spectra of naturally-occurring chromophores [20], provides a cheap, non-invasive and relatively portable modality that can be used at the bedside [21, 22]. NIRS has been used experimentally in both healthy and injured human adults [23–26] and neonates [27–35], as well as in animal models, notably piglets [36–40]. It provides a useful complement to techniques such as MRS in physiological investigations [41, 42]. However, it has yet to be widely adopted in the clinic [9, 43], in part due to difficulties of interpretation.

We have previously developed several computational models of cerebral physiology to investigate the relationships between measurable signals and the underlying physiological state. In particular, the BrainSignals model [44–46] is designed to assist the interpretation of two major NIRS signals: haemoglobin oxygen saturation [47], and the oxidation state of the metabolic enzyme cytochrome c oxidase [48–52].

Such models are necessarily complex. BrainSignals is a simplification of an earlier model, BrainCirc [53], eliminating or caricaturing many processes not relevant to the NIRS problem. Still, it remains complex enough to be computationally expensive and difficult to analyse. Like most Systems Biology models, it depends on a large number of parameters—135 in the core model, relative to just 12 state variables—many of which are not accurately known. Some do not correspond to a measurable physical property. For others, measurements may only exist for different organisms or in unrealistic conditions, or they may vary significantly between individuals.

Typically, not all parameters of a model need to be known with perfect accuracy. Sensitivity analysis [54–57] can help identify parameters for which a lack of precision is acceptable. Moreover, many Systems Biology models exhibit a property known as ‘sloppiness’ [58, 59]. In these models, the behaviour is dominated by a small number of (so-called ‘stiff’) directions in parameter space, while other directions are much less constrained (‘sloppy’). The parameter space can be thought of as a high-dimensional projection of a lower-dimensional space of model behaviour [60, 61]. Since the behaviour arises jointly from the parameter ensemble, individual parameter values are often uninformative.

To improve interpretability and computational efficiency, we set out to simplify BrainSignals further, retaining the essential model structure but reducing the number of parameters and the complexity of the calculations. We employed a hybrid mechanistic-statistical approach, whereby some local subsystems in the model were replaced with simpler functional approximations. Our focus was on preserving the model’s empirical behaviour. Thus, substitution was attempted where a recognisable behavioural simplicity—in fact, linearity—arose from the more complex underlying processes. Success was judged on the ability of the new models to mimic the original over a range of different physiological conditions.

## Methods

### Ethics statement

The study was approved by the National Hospital for Neurology and Neurosurgery and Institute of Neurology Research Ethics Committee, study number 04/Q0512/67. All subjects provided written informed consent.

### Software and modelling

Models were implemented in the open source Brain/Circulation Model Developer (BCMD) environment, a replacement for the BRAINCIRC interface with which BrainSignals was originally developed [44]. Like its predecessor, BCMD employs the RADAU5 library [62] to solve numerically the models’ differential-algebraic equation systems. The software and all model implementations are freely available from http://tinyurl.com/ucl-bcmd. Details of all model definitions are included in S1 Text.

To illustrate the model structures, dependency diagrams are presented in several figures below. These were produced using GraphViz [63], from DOT language descriptions generated by BCMD. Several node types are distinguished in these graphs. The reader is not expected to unpick these in detail—the minutiae of the model are more easily understood via the implementation files referenced above—but we briefly outline the differences here as an aid to interpretation:

- Circular nodes with a double border are the internal state variables. These are further separated into those defined via a differential equation (depicted in orange) and those defined via an algebraic relation (green). An example of the former is the membrane potential *ψ* in Eq 10, while the vessel radius *r* in Eq B1 is an example of the latter.
- Circular nodes with a single border (and blue fill) are ‘temporary’ variables. These represent intermediate expressions that must be computed *en route* to solving for the state variables. There is some implementation leeway in how these are defined, so they provide only an approximate metric of complexity, but they indicate the presence of a non-trivial, usually non-linear, calculation. The variables *G* and *μ* in Eqs 2 and 3 are examples of temporary variables.
- Rectangular nodes are values external to the model. These may be input values (red), variables from other submodels (pale green) or parameters. (The latter are omitted from the main body figures as they make the graphs excessively complicated. However, they are included in S1–S7 Figs)
- Dependencies are represented by arrows, indicating the direction of data flow: the arrowhead points to the dependent variable.

Variable names in these figures are those used in the model implementations. In most cases the relationship to the mathematical notation used in the text should be obvious. Both forms are given for all variables in the model definitions in S1 Text.

**Fig 1:**
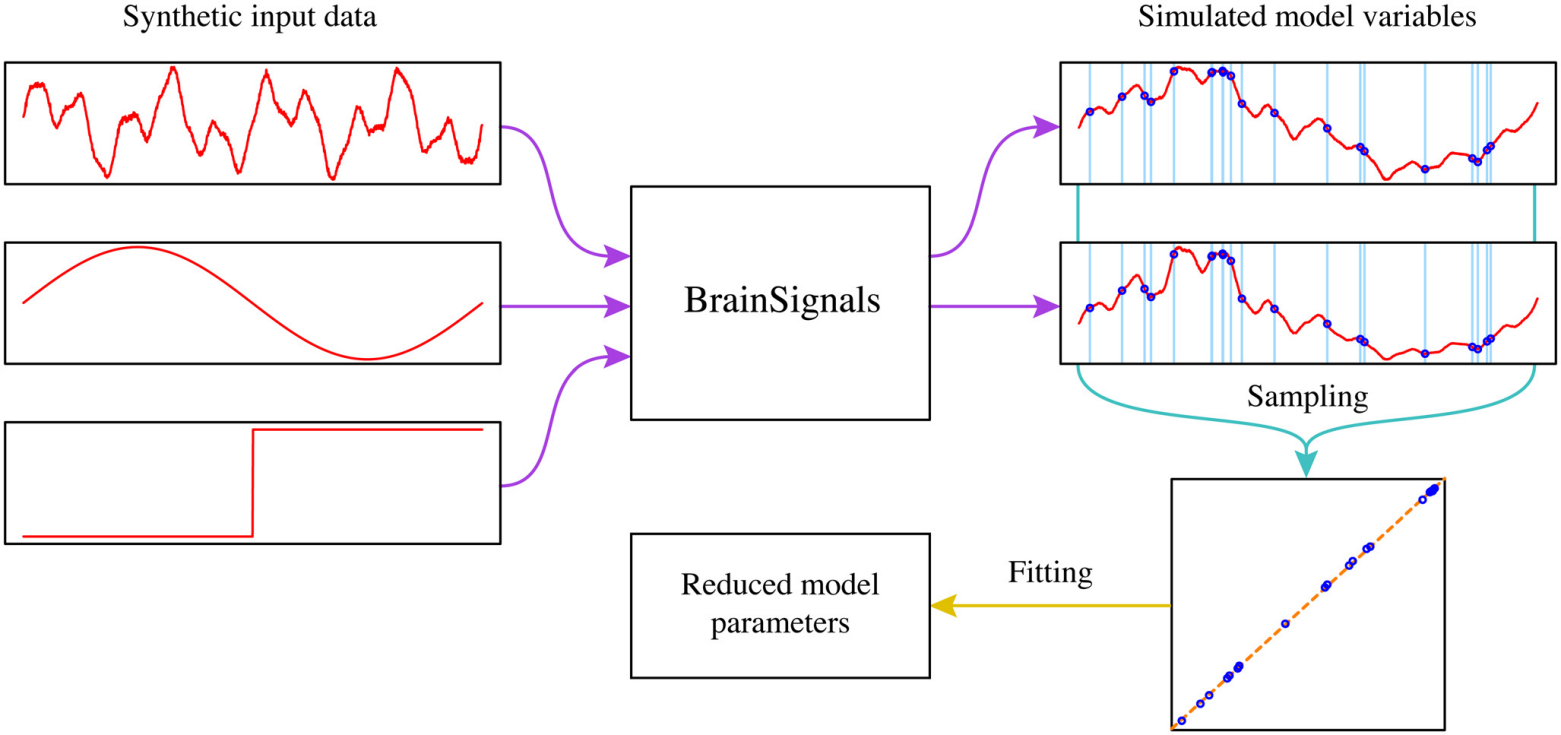
Fitting procedure for the simplified parameters. A variety of synthetic signals, including oscillations, noise and step changes, were used as inputs to the BrainSignals model, generating a large number of simulated time series for the model variables. A subset of 10,000 data points was randomly sampled from the full set of simulation data, and parameters for the simplified linear models were estimated from the sampled values by ordinary least-squares regression.

Parameters for simplified submodels were fitted using the following procedure, illustrated schematically in Fig 1. Data were simulated from the full BrainSignals model, using a range of synthetic input signals designed to explore different aspects of the model behaviour. The inputs were generated in R [64], and included noise, simple oscillations, slow ramps, large step changes, and combinations of the above, across plausible physiological ranges for each input parameter. Since the full set of simulations was very large (and highly redundant), fits were performed with randomly-sampled subsets of 10,000 data points; consequently the exact fit values could vary somewhat, but these variations were typically small. In some cases, notably the *P_a_* dependence in the blood flow variants, additional steady state data for more extreme parameter ranges were also included to improve the fitted behaviour. Parameters were estimated using the standard R linear modelling function lm. The complete set of synthetic data files and the R script used to generate them are available from http://dx.doi.org/doi:10.5281/zenodo.16776.

Sensitivity analyses were performed with the extended Fourier amplitude sensitivity test (eFAST) [55, 65], implemented using the SALib Python library (http://jdherman.github.io/SALib/).

Instrumental data were filtered, detrended and resampled in MATLAB. Data analysis, visualisation and parameter fitting for linear models were performed in R. Figures were prepared in Adobe Illustrator CS3 and Adobe PhotoShop CS3.

### Experimental data

The experimental datasets used for model testing are described in more detail in a previous paper [52]. In brief, hypercapnia was induced in healthy adult volunteers by supplementing the inhaled gas mixture with carbon dioxide and observing the induced change in cerebral blood flow and oxygenation. Monitoring included broadband NIRS [66], transcranial Doppler ultrasound-derived flow velocity in the middle cerebral artery, continuous arterial blood pressure, pulse oximetry and end tidal CO_2_, captured at 125 Hz. Data were low-pass filtered at 0.1 Hz with a 5th-order Butterworth filter and resampled to a uniform 3.2 s sampling interval to match the integration time of the NIRS. Only individual data recordings were used, not group averages. Experimental data used are available from http://dx.doi.org/doi:10.5281/zenodo.16776.

### Model reduction

Our strategy for simplifying the model may be summarised in the following steps:

1. **Decomposition** The model was first decomposed into weakly-connected submodels. This partitioning was done manually, guided by structural information from the BCMD tools together with *a priori* knowledge of the model design.
2. **Algebraic simplification** Where feasible without altering their substantive meaning, model expressions were restructured and rearranged to simplify their terms.
3. **Refactoring** Model implementation code was cleaned up and refactored, removing superfluous elements and unreached contingencies.
4. **Lumping** Where multiple unknown parameters contributed to an expression in broadly-unidentifiable ways, these were substituted with lumped parameters. Our criteria for such lumping were pragmatic rather than strict: some parameters might in principle be identifiable given sufficient data, but the quantity and quality required are unrealistic.
5. **Functional substitution** Local functional relationships were isolated and replaced with fitted approximators. Candidate relationships were identified by inspection, using knowledge of the model structure. The approximating functions were (in the general sense) linear models, allowing parameters to be estimated from data by standard regression methods.

Steps 1–3 concern implementation details and do not materially alter the model, although they can involve a substantial reduction in the *apparent* complexity. Their application should be uncontroversial, and indeed they might be considered ‘not worth mentioning’. Nevertheless, they form an important part of the simplification process. Steps 4 and 5 constitute ‘model reduction’ more properly, in the sense that they result in a new and different model that only approximates the original. Inevitably this raises questions of validity and applicability, which we shall address in the Discussion section, below.

### Model structure

**Fig 2:**
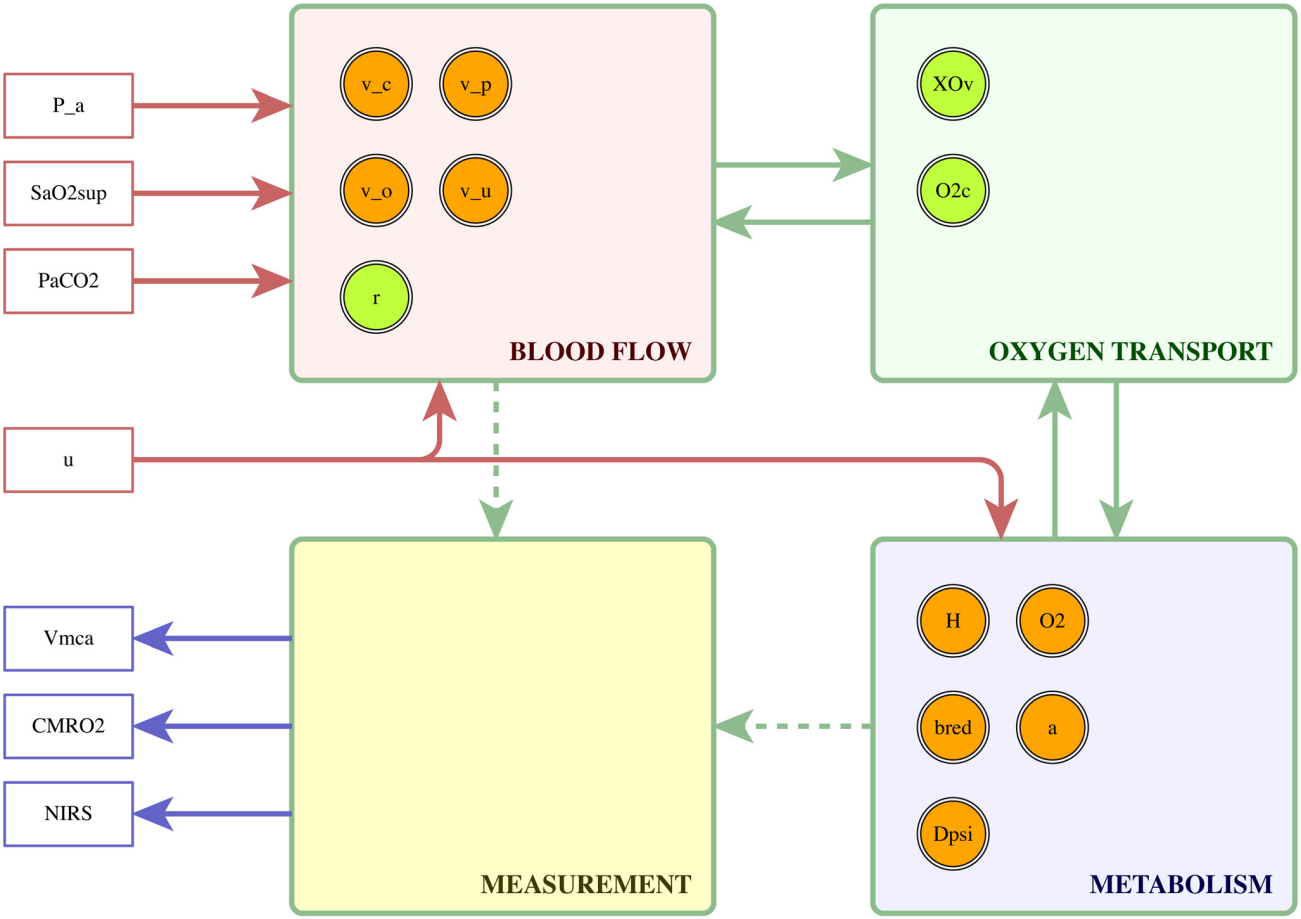
Overall structure shared by BrainSignals and the simplified models. Systemic measurements of mean arterial blood pressure, arterial oxygen saturation and partial pressure of CO_2_, together with a parameter specifying the relative demand, serve as model inputs. A blood flow submodel represents the delivery of oxygenated blood from the arteries through the capillary bed to the veins, and an oxygen transport submodel estimates diffusion of dissolved O_2_ from the capillary blood to the brain tissue. Delivered oxygen is utilised by a metabolic submodel, with an external dependence on the demand. Finally, a measurement submodel translates the internal states of the blood flow and metabolic submodels into observable outputs. Circles within each submodel indicate the local state variables—note that the measurement submodel has no state of its own.

All model variants retain the gross structure of BrainSignals, illustrated in Fig 2, with the same inputs, outputs and state variables. There are four constituent submodels, representing blood flow, oxygen transport from blood to tissue, oxidative metabolism within the tissue, and measurement. Although the submodels are not wholly decoupled, the boundaries were chosen to minimise interdependence. A single state variable, the vessel radius *r*, feeds forward from the blood flow submodel to the oxygen transport submodel, while another, the capillary oxygen concentration *O_2,c_*, feeds back. Similarly, one output from the oxygen transport submodel, the oxygen flux *J_O_2__*, feeds forward to the metabolic submodel, and one variable, the tissue oxygen concentration *O_2_*, feeds back.

All submodels underwent rationalisation of implementation, corresponding to steps 2 and 3 of the strategy discussed in the previous section, but only the haemodynamic and metabolic submodels were substituted with multiple simplified alternatives. The measurement submodel is purely an output layer, translating the model state into forms comparable with instrumental data, and thus has no effect on the model behaviour. The oxygen transport model is already heavily simplified, and there is little scope for further reduction without loss of utility.

### Blood flow

Oxygen delivery to the brain is dependent on cerebral blood flow (CBF), which carries oxygen bound to haemoglobin and, to a lesser extent, dissolved in plasma. The flow is driven by the pressure difference across the cerebral blood vessels, and depends on the vessels’ conductance, which is passively affected by factors such as intracranial pressure but also actively regulated in response to various physiological stimuli to maintain oxygen supply [1].

The BrainSignals blood flow submodel is based on a substantial simplification of a popular series of models developed by Ursino and Lodi [67, 68]. A number of model features are omitted, notably compartmental compliances, the distinction between distal and proximal vessels and the production and absorption of cerebrospinal fluid. The rationale for these omissions is that the model is designed for the simulation of longer time scale changes such as those induced in the experimental challenges described below, rather than those occurring on the timescale of individual heart beats.

Intracranial pressure and venous sinus pressure (*P_v_*) are not modelled explicitly, but are included as parameters. There are three nominal conductive compartments, but only the cerebral arterial-arteriolar compartment is modelled in detail. CBF is a function of the conductance of this compartment, *G*, and the arterial blood pressure, *P_a_*:

$$\begin{array}{c} CBF=(P_{a}-P_{v})G \end{array}$$ (1)

Conductance is taken to be determined by a shared vessel radius, *r*, which is uniform throughout the compartment, according to a version of Poiseuille’s Law

$$\begin{array}{c} G=K_{G}r^{4} \end{array}$$ (2)

where the parameter *K_G_* subsumes the unknown (and in this context fairly meaningless) values of length and viscosity. Blood is non-Newtonian and the capillary bed is a non-uniform network, so this is obviously a crude approximation, but it is widely adopted in the literature and provides a useful conceptual relationship between blood flow and volume. Since the vessel walls are not rigid, the radius is affected passively by the pressure, but is also subject to active autoregulation.

Four autoregulatory stimuli are admitted: arterial pressure, blood partial pressure of CO_2_, capillary O_2_ concentration and metabolic demand. Each stimulus is passed through a first order filter with its own characteristic time constant, representing differences in the time course of the regulatory responses, and a linear combination of the filtered values is taken as the overall stimulus level, *η*.

**Fig 3:**
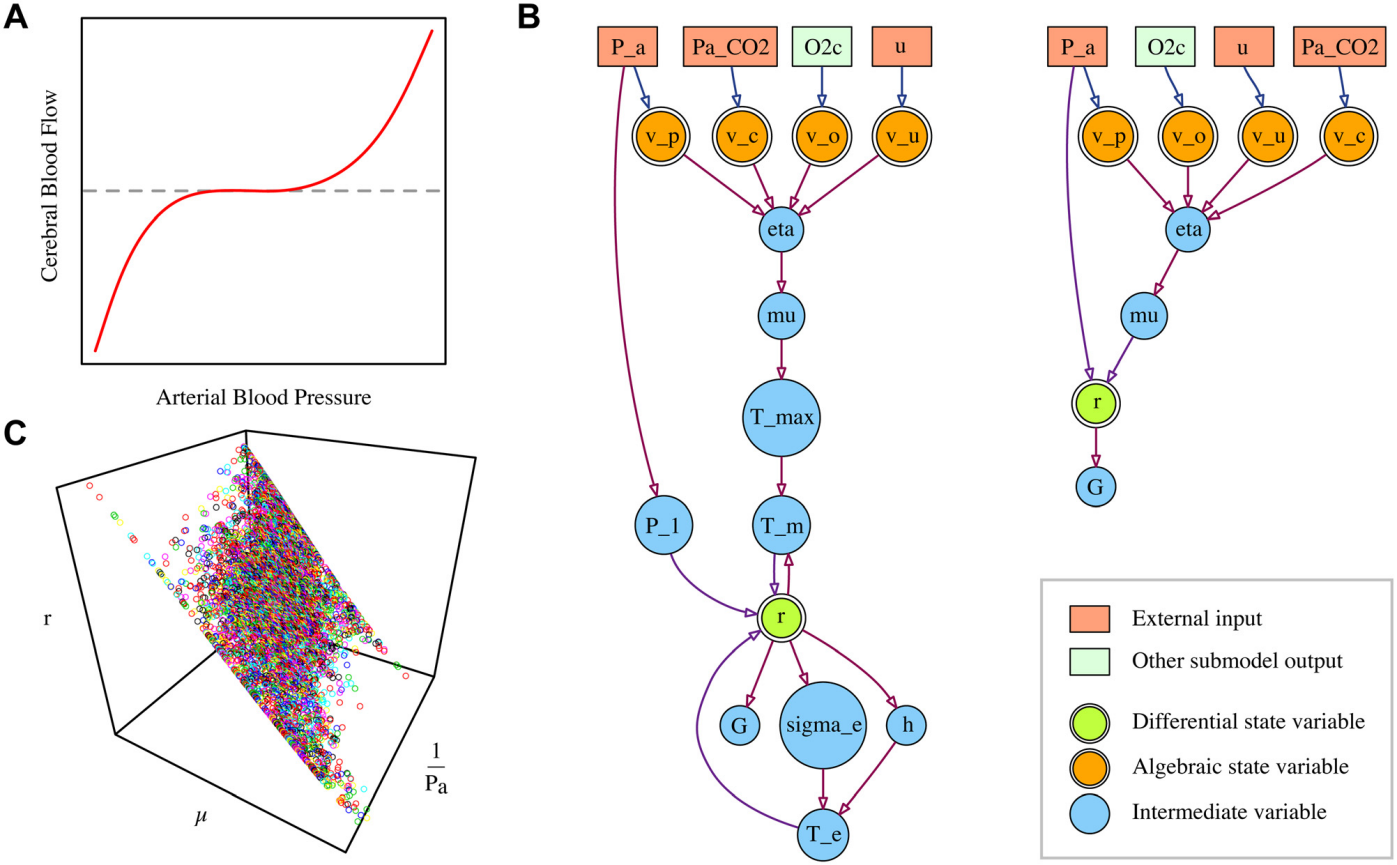
Simplifying the blood flow submodel. **A** Expected shape of the autoregulatory response. An approximately constant supply is maintained for variations in blood pressure, until conditions exceed the body’s capacity to regulate. **B** Main variables and dependencies of the BrainSignals blood flow submodel and the simplified equivalent. **C** BrainSignals simulations exhibit a nearly planar response to ‘normal’ values of *μ* and $\frac{1}{P_{a}}$.

Autoregulation is commonly assumed to manifest as shown in Fig 3A, with supply being maintained approximately constant over a range of ‘normal’ conditions but control failing when conditions become more extreme. This bounded range of response effectiveness is modelled using a sigmoidal transformation of *η*:

$$\begin{array}{c} \mu =\frac{e^{\eta }-1}{e^{\eta }+1} \end{array}$$ (3)

Autoregulation is often impaired following acute brain injury [69, 70], so an important control parameter, *k_aut_*, is introduced to represent the level of functional autoregulatory response. The central relationship of the blood flow submodel can then be summarised as:

$$\begin{array}{c} r=f(P_{a},\mu ,k_{aut}) \end{array}$$ (4)

Here and subsequently, f() stands for some unknown function.

Following Ursino and Lodi, BrainSignals defines *r* through the balance of pressures inside and outside the vessel and the tension in the vessel wall. The latter is in turn broken down into a passive elastic component, *T_e_*, and a muscular component, *T_m_*, which is under autoregulatory control. The relationship is formulated implicitly, requiring the simultaneous solution of several non-linear equations, and cannot be directly expressed in the form of Eq 4. Although there is an underlying biophysical justification for the model structure, the equations are opaque and shaped by a number of tuning parameters with no straightforward physiological meaning. The relationship is thus an obvious choice for substitution.

Since *μ* and *k_aut_* jointly define the strength of the autoregulatory response, we merge these into a single term, $\tilde{\mu }=k_{aut}\mu$, for the purposes of fitting. A coarse examination of the equations for *r* indicates that, while *μ* enters in the numerator, *P_a_* does so via the denominator, so we use the reciprocal of the latter term. Simulation data from BrainSignals is shown in Fig 3C. The planar form suggests the relationship can indeed be approximated as linear over a reasonable range of values for these variables. We note that, since arterial pressure is also one of the regulatory stimuli, the terms are not independent. Nevertheless, we adopt the following linear model, which we call B1, as our initial candidate:

$$\begin{array}{c} r=\lambda_{r}+\frac{\lambda_{r,p}}{P_{a}}+\lambda_{r,\mu }\tilde{\mu }+\frac{\lambda_{r,p,\mu }\tilde{\mu }}{P_{a}} \end{array}$$ (B1)

The four *λ*_(⋅)_ coefficients are parameters to be estimated from the synthetic data: *λ*_*r*_ is the intercept term, *λ*_*r*,*p*_ and *λ*_*r*,*μ*_ estimate linear dependences on $\frac{1}{P_{a}}$ and $\tilde{\mu }$ respectively, while *λ*_*r*,*p*,*μ*_ represents an interaction between both factors. Since it is possible that one or more of these terms is negligible, we also define three simpler variants, B2–B4, with terms omitted:

$$\begin{array}{c} r=\lambda_{r}+\frac{\lambda_{r,p}}{P_{a}}+\lambda_{r,\mu }\tilde{\mu } \end{array}$$ (B2)

$$\begin{array}{c} r=\lambda_{r}+\lambda_{r,\mu }\tilde{\mu } \end{array}$$ (B3)

$$\begin{array}{c} r=\lambda_{r}+\frac{\lambda_{r,p}}{P_{a}} \end{array}$$ (B4)

For comparison, the core structure of the original bloodflow submodel and the simplified version B1 are depicted in graphical form in Fig 3B. Diagrams of the full structures, including parameters, can be found in S1 and S2 Figs. The relative sizes of the blood flow variants, in terms of parameters and temporary variables, are given in Table 1.

**Table 1:** Relative sizes of the different variants of the blood flow submodel.

Model	State Variables	Parameters	Temporary Variables
BrainSignals (blood flow)	5	45	8
B1	5	27	3
B2	5	26	3
B3	5	25	3
B4	5	25	3

### Metabolism

Oxidative metabolism represents the ‘consumption’ portion of the model, where the delivered oxygen is used in the satisfaction of energy demand. Only a small fraction of the real biochemical network involved in metabolism is modelled, concentrating on the final portion of the mitochondrial electron transport chain. This focus is motivated by the fact that near-infrared absorption spectrum of cytochrome c oxidase (CCO) is dependent on the oxidation state of the Cu_A_ centre, providing a marker of metabolic activity that can be measured by NIRS [48].

**Fig 4:**
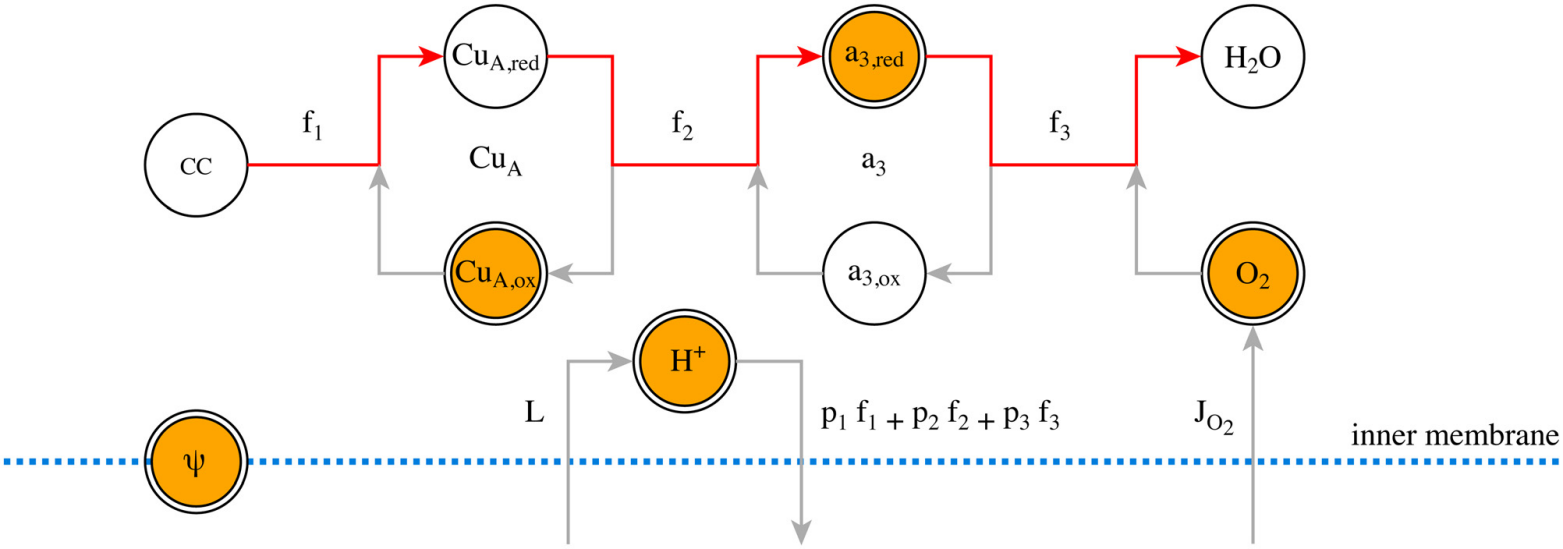
The modelled part of the mitochondrial electron transport chain. The electron transfer is indicated by red arrows, while the grey arrows show corresponding species changes. Quantities enclosed in unfilled circles are not explicitly modelled. The CCO Cu_A_ and a_3_ centres are assumed present at constant concentration, so any change in the oxidised form implies an opposite change in the reduced form. The initial reducing substrate (denoted CC) is neglected on the assumption that O_2_ and H^+^ are limiting, and the H_2_O product vanishes into an effectively-infinite background. Protons are exported from the matrix by each electron transport step, returning at rate *L*, and the membrane potential *ψ* is affected by the net proton flux.

The modelled portion of the reaction network is illustrated in Fig 4. We assume a constant total concentration of mitochondrial CCO, with varying oxidation states for the a_3_ and Cu_A_ centres. Mitochondrial H^+^ and O_2_ are consumed or exported as a result of the three electron transport reactions, summarised in the following formulae:

$$\begin{array}{c} 4{\mathrm{Cu}}_{A,ox}+p_{1}H^{+}\overset{f_{1}}{\to } \end{array}$$ (5)

$$\begin{array}{c} p_{2}H^{+}\overset{f_{2}}{\to }4{\mathrm{Cu}}_{A,ox}+4a_{3,red} \end{array}$$ (6)

$$\begin{array}{c} O_{2}+4a_{3,red}+p_{3}H^{+}\overset{f_{3}}{\to } \end{array}$$ (7)

Rates *f_1_*, *f_2_* and *f_3_* are for the transfer of 4 electrons, corresponding to the consumption of 1 molecule of O_2_. The parameters *p_1_*, *p_2_* and *p_3_* represent the proton ‘cost’ of each reaction, always set to 12, 4 and 4, respectively. Two supply reactions model the return of protons to the mitochondrial matrix and the delivery of oxygen from the blood:

$$\begin{array}{c} \overset{L}{\to }H^{+} \end{array}$$ (8)

$$\begin{array}{c} \overset{J_{O_{2}}}{\to }O_{2} \end{array}$$ (9)

Finally, the proton current out of the mitochondria affects the inner membrane potential by way of the membrane capacitance *C_im_*:

$$\begin{array}{c} \frac{d\psi }{dt}=\frac{p_{1}f_{1}+p_{2}f_{2}+p_{3}f_{3}-L}{C_{im}} \end{array}$$ (10)

**Fig 5:**
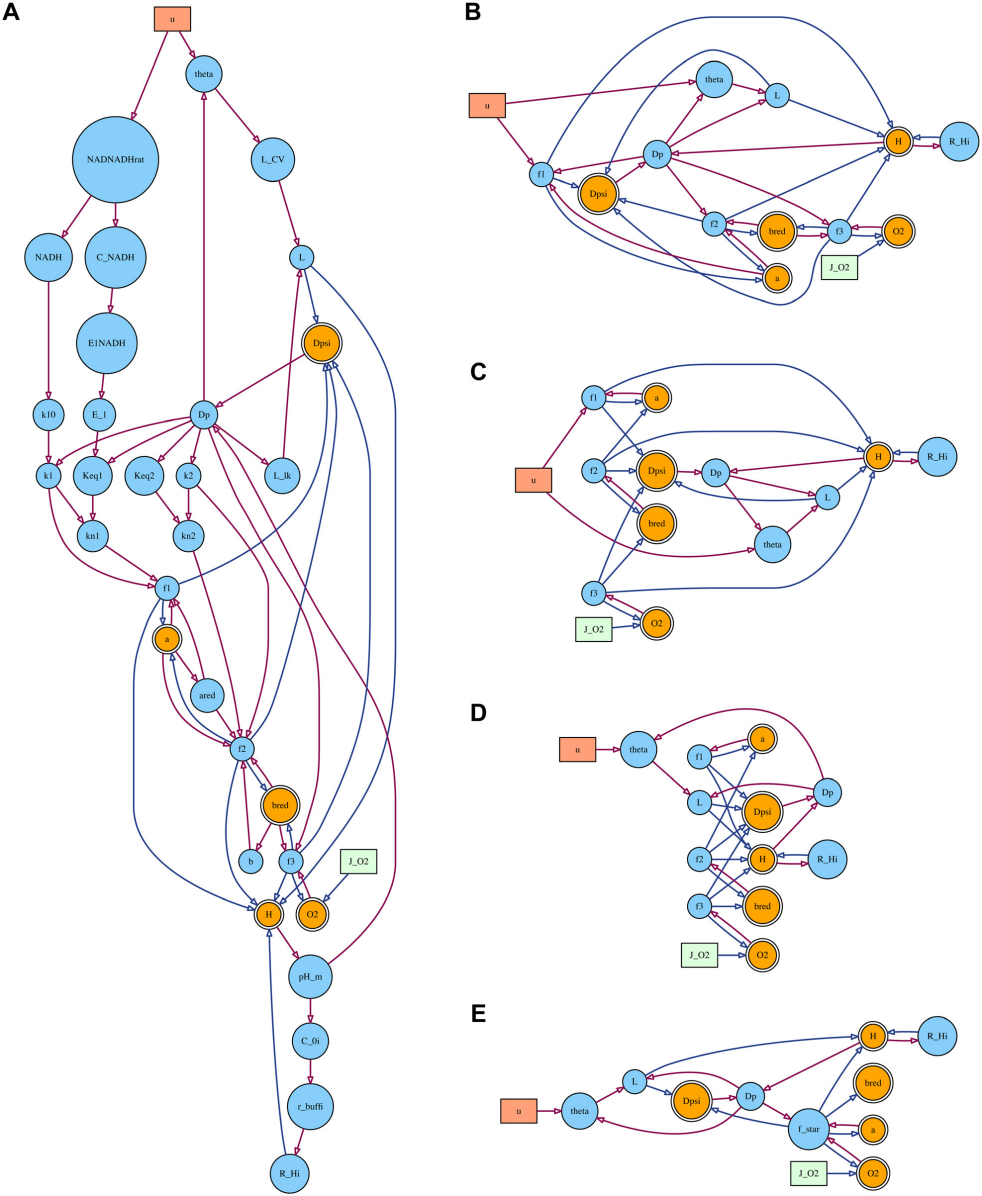
Simplifying the metabolic submodel. **A** The main structure of the BrainSignals metabolic submodel, with parameters omitted. Although the number of species is small and the reactions apparently simple, the reaction rates are governed by a complex network of interactions. **B, C, D, E** Progressively simplified submodel variants M0–M3, corresponding to Eqs M0.1–M3.2 in the text.

While the reactions are apparently straightforward, a great deal of complexity is wrapped within the definition of the rate terms, which are dependent on the species concentrations and membrane potential in intricately-parameterised ways. This network of relations is illustrated in Fig 5A; for the full details see the original description [44] (also summarised in S1 Text). The equations resist algebraic simplification, but we can consolidate the main functional dependencies of the rate constants as follows:

$$\begin{array}{c} f_{1}=f\left(\Delta p,\left[{\mathrm{Cu}}_{A,ox}\right],u\right) \end{array}$$ (11)

$$\begin{array}{c} f_{2}=f\left(\Delta p,\left[{\mathrm{Cu}}_{A,ox}\right],\left[a_{3,red}\right]\right) \end{array}$$ (12)

$$\begin{array}{c} f_{3}=f\left(\Delta p,\left[O_{2}\right],\left[a_{3,red}\right]\right) \end{array}$$ (13)

$$\begin{array}{c} L=f(\theta ,\Delta p) \end{array}$$ (14)

where Δ*p* = f([H^+^], *ψ*) is the driving force for protons and *θ* = f(Δ*p*, *u*) is the driving force for Complex V.

**Fig 6:**
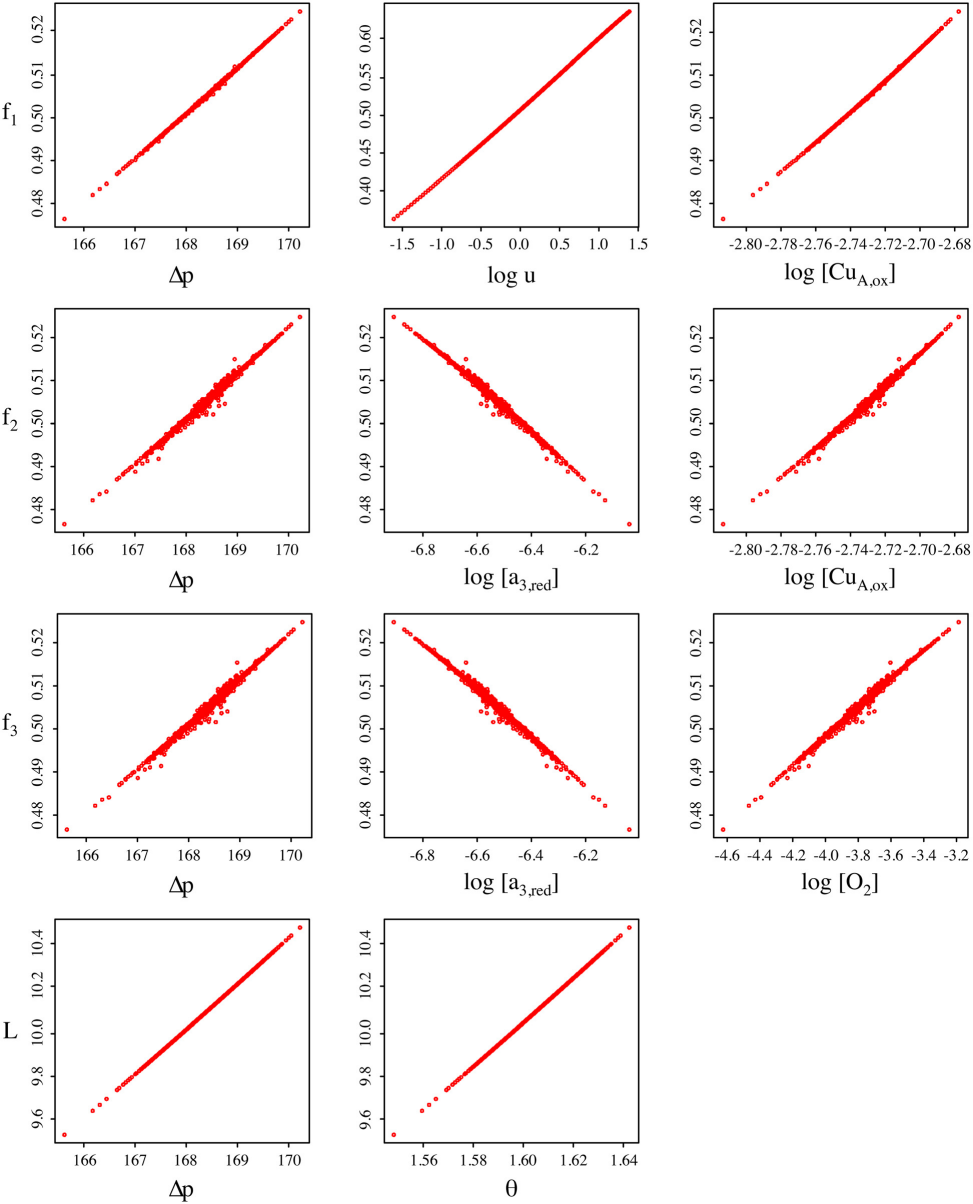
Dependencies between the reaction rate constants and variables in the metabolic submodel. All rate constants exhibit a very nearly linear relationship to the model variables on which they are primarily dependent, suggesting that a good approximation can be obtained with a linear model. However, the relationships are also very highly correlated, implying the model is overdetermined and further reduction is required.

By inspection of the original model equations we expect the dependencies on species concentrations and demand to be approximately logarithmic. Simulation data are plotted in Fig 6, using log values where appropriate. The relationships appear strongly linear, suggesting a model of the following form, which we will refer to as variant M0:

$$\begin{array}{c} f_{1}=\lambda_{f_{1}}+\lambda_{f_{1},p}\Delta p+\lambda_{f_{1},a}\log\left[{\mathrm{Cu}}_{A,ox}\right]+\lambda_{f_{1},u}\log u \end{array}$$ (M0.1)

$$\begin{array}{c} f_{2}=\lambda_{f_{2}}+\lambda_{f_{2},p}\Delta p+\lambda_{f_{2},a}\log\left[{\mathrm{Cu}}_{A,ox}\right]+\lambda_{f_{1},b}\log\left[a_{3,red}\right] \end{array}$$ (M0.2)

$$\begin{array}{c} f_{3}=\lambda_{f_{3}}+\lambda_{f_{3},p}\Delta p+\lambda_{f_{3},O}\log\left[O_{2}\right]+\lambda_{f_{3},b}\log\left[a_{3,red}\right] \end{array}$$ (M0.3)

$$\begin{array}{c} L=\lambda_{L}+\lambda_{L,p}\Delta p+\lambda_{L,\theta }\theta \end{array}$$ (M0.4)

As in the previous section, the *λ*_(⋅)_ coefficients are simply weights for each linear term, which we can estimate from the simulation data by linear regression. The model structure is illustrated in Fig 5B. However, it is obvious from the plots in Fig 6 that the relationships are very highly correlated. If the model is fitted with parameters for all degrees of freedom, the resulting system is overdetermined, with no feasible solution. We therefore seek a less connected model with fewer parameters. One approach to this reduction is simply to eliminate some of the competing dependencies from the above equations. A variety of choices for removal are possible. We show one such in Fig 5C, denoted variant M1 and defined as follows:

$$\begin{array}{c} f_{1}=\lambda_{f_{1}}+\lambda_{f_{1},a}\log\left[{\mathrm{Cu}}_{A,ox}\right]+\lambda_{f_{1},u}\log u \end{array}$$ (M1.1)

$$\begin{array}{c} f_{2}=\lambda_{f_{2}}+\lambda_{f_{1},b}\log\left[a_{3,red}\right] \end{array}$$ (M1.2)

$$\begin{array}{c} f_{3}=\lambda_{f_{3}}+\lambda_{f_{3},O}\log\left[O_{2}\right] \end{array}$$ (M1.3)

$$\begin{array}{c} L=\lambda_{L}+\lambda_{L,p}\Delta p+\lambda_{L,\theta }\theta \end{array}$$ (M1.4)

In this variant, each dependency is included directly for only one of the rates. The terms are still not independent, since the demand parameter *u* enters the system via multiple routes, both directly in Eq M1.1 and also via the Complex V term *θ*, which in turn also depends on Δ*p*. A further simplification (variant M2, Fig 5D), eliminates the dependency of *f_1_* on *u*, replacing Eq M1.1 with:

$$\begin{array}{c} f_{1}=\lambda_{f_{1}}+\lambda_{f_{1},a}\log\left[{\mathrm{Cu}}_{A,ox}\right] \end{array}$$ (M2.1)

An alternative approach to reducing model M0 may be made by first noting that the three rates *f_1_*, *f_2_* and *f_3_* are, by design, identical under baseline conditions. We can make this into an explicit constraint by merging these into a single shared rate, *f* ^⋆^. This is a significant structural change, implying that the three metabolic reactions proceed in step—a questionable assumption. Nevertheless, we trial this as the further variant M3 (Fig 5E):

$$\begin{array}{c} f^{⋆}=\lambda_{f^{⋆}}+\lambda_{f^{⋆},p}\Delta p+\lambda_{f^{⋆},O}\log\left[O_{2}\right]+\lambda_{f^{⋆},a}\log\left[{\mathrm{Cu}}_{A,ox}\right] \end{array}$$ (M3.1)

$$\begin{array}{c} L=\lambda_{L}+\lambda_{L,p}\Delta p+\lambda_{L,\theta }\theta \end{array}$$ (M3.2)

Diagrams of the full structures of all the metabolic submodel variants, including parameters, can be found in S3–S7 Figs Table 2 lists the relative sizes in terms of numbers of parameters and temporary variables.

**Table 2:** Relative sizes of the different variants of the metabolic submodel.

Model	State Variables	Parameters	Temporary Variables
BrainSignals (metabolism)	5	74	20
M0	5	30	7
M1	5	25	7
M2	5	24	7
M3	5	22	5

## Results

### Fitting and testing the variant models

Fitting was performed to synthetic data as described in the Methods section. For all variants discussed here, the fitting indicated the existence of a non-zero dependent relationship with high significance: *p* < 2 × 10^−16^ for both individual parameter *t* tests and overall model *F* tests, with standard errors < 1% on all parameter estimates. However, this is in part due to the large simulated sample sizes and does not imply model correctness. As explicit simplifications, the variant models are, by definition, *not* correct.

### Blood flow

Although fitting to all the variants B1–B4 confirmed highly significant dependencies on their respective variables, B3 and B4 were unable adequately to explain the data variance (*R^2^* = 0.75 and *R^2^* = 0.52, respectively), consistent with our analysis of the model structure. The models including both *P_a_* and $\tilde{\mu }$ each had *R* ^2^ > 0.99, with inclusion of the interaction term in B1 providing a marginal additional improvement.

**Fig 7:**
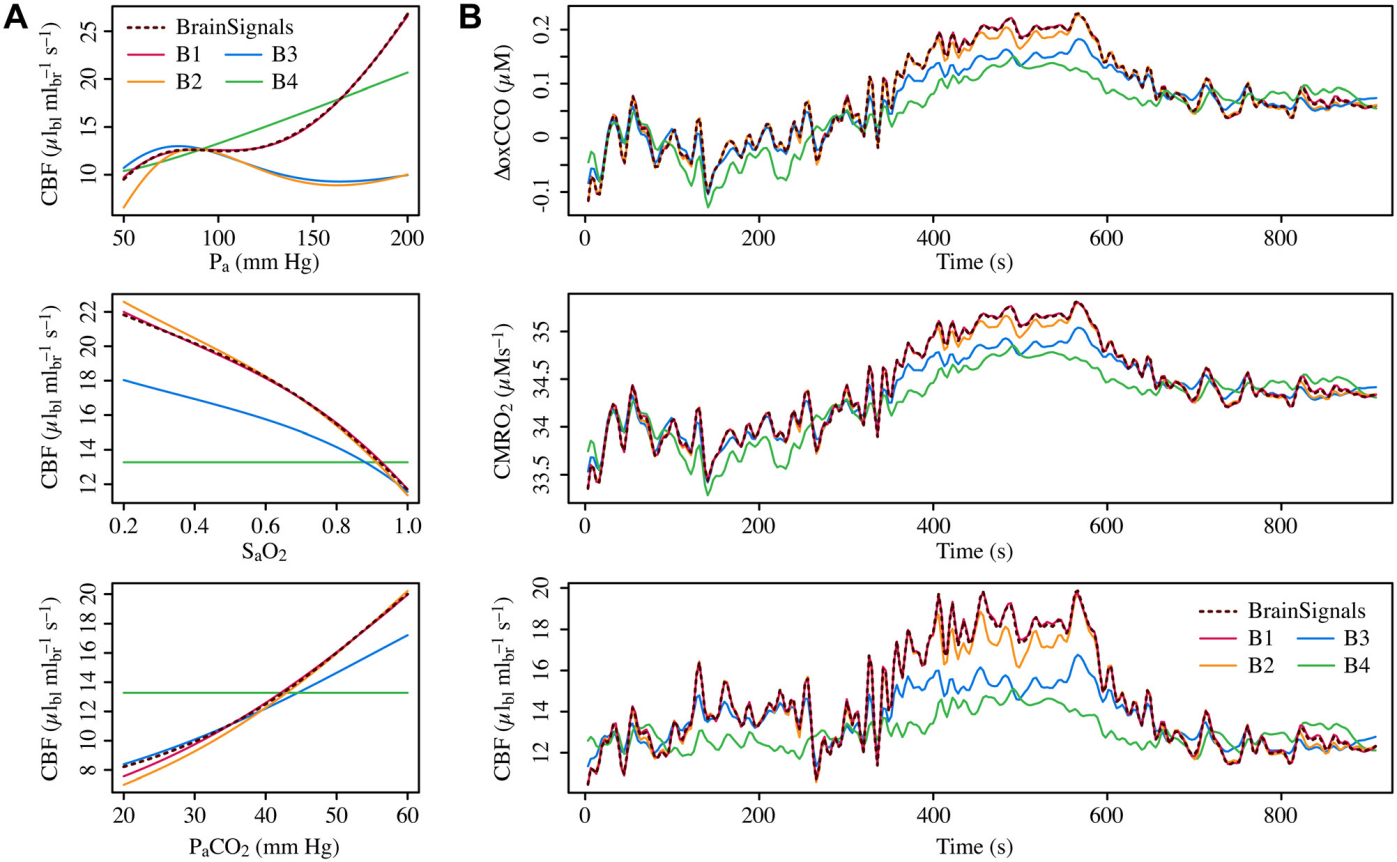
Comparing behaviour of simplified blood flow submodels to BrainSignals. **A** Steady state simulations of blood flow for different levels of arterial blood pressure (*P_a_*), oxygen saturation (*S_a_O_2_*) and partial pressure of carbon dioxide (*P_a_CO_2_*). Model variant B4 lacks a dependence on *μ*, and so does not autoregulate. Variants B1–B3 do so to varying degrees, with B1 providing the best approximation of the BrainSignals behaviour. B2 is very similar for changes in *S_a_O_2_* and *P_a_CO_2_*, but fails for *P_a_*. **B** Time courses of model outputs driven by real experimental data. All three plots show a representative example of output signals simulated using inputs recorded from a healthy adult undergoing a hypercapnia challenge. Blood flow variant B1 is again able to replicate BrainSignals almost exactly, and B2 also approximates quite closely. Variants B3 and B4 do not. (Average output distances for simulations from all experimental subjects are given in Table 3.)

Comparisons of the original BrainSignals steady-state behaviour with that of each blood flow variant, with all other model components kept unchanged, are shown in Fig 7A. Comparisons of dynamic behaviour for inputs from a representative experimental subject are shown in Fig 7B, while summary results for all subjects are given in Table 3. Predictably, variants B3 and B4 produce very poor approximations for both dynamic and steady state simulations, in particular B4, which is unable to capture autoregulation since it lacks any dependence on $\tilde{\mu }$. It can be seen that variant B1 mimics BrainSignals almost exactly in all cases. B2 is close in most cases, but fails notably in the *P_a_* steady state simulation. We can attribute this failure to the conflicting roles of *P_a_* on the active and passive changes to *r*, which cannot be readily captured without the interaction term.

**Table 3:** Summary output distances for the different model variants.

Model	CBF (μl_bl_ ml_br_^-1^ s^-1^)	CMRO_2_ (μMs^-1^)	ΔoxCCO (μM)
B1	2.6 ± 2.5	1.1 ± 1.5	0.2 ± 0.3
B2	9.3 ± 7.0	2.0 ± 2.2	0.4 ± 0.4
B3	17.5 ± 7.2	2.7 ± 1.9	0.5 ± 0.3
B4	37.6 ± 6.3	7.1 ± 4.0	1.3 ± 0.7
M1	0.0 ± 0.0	0.3 ± 0.1	0.2 ± 0.1
M2	0.0 ± 0.0	0.3 ± 0.1	0.1 ± 0.0
M3	0.6 ± 0.1	5.4 ± 1.0	1.9 ± 0.4
B1M1	2.6 ± 2.5	1.2 ± 1.3	0.3 ± 0.1
B1M2	2.6 ± 2.5	1.2 ± 1.3	0.2 ± 0.2
B2M1	9.3 ± 7.0	1.9 ± 2.1	0.3 ± 0.2
B2M2	9.3 ± 7.0	1.9 ± 2.1	0.4 ± 0.4

Outputs from the different model variants were compared to those from BrainSignals for simulations with 10 sets of hypercapnia input data. Euclidean distance was used as the metric of dissimilarity; higher values indicate a worse fit. Results are shown as median ± median absolute deviation.

**Fig 8:**
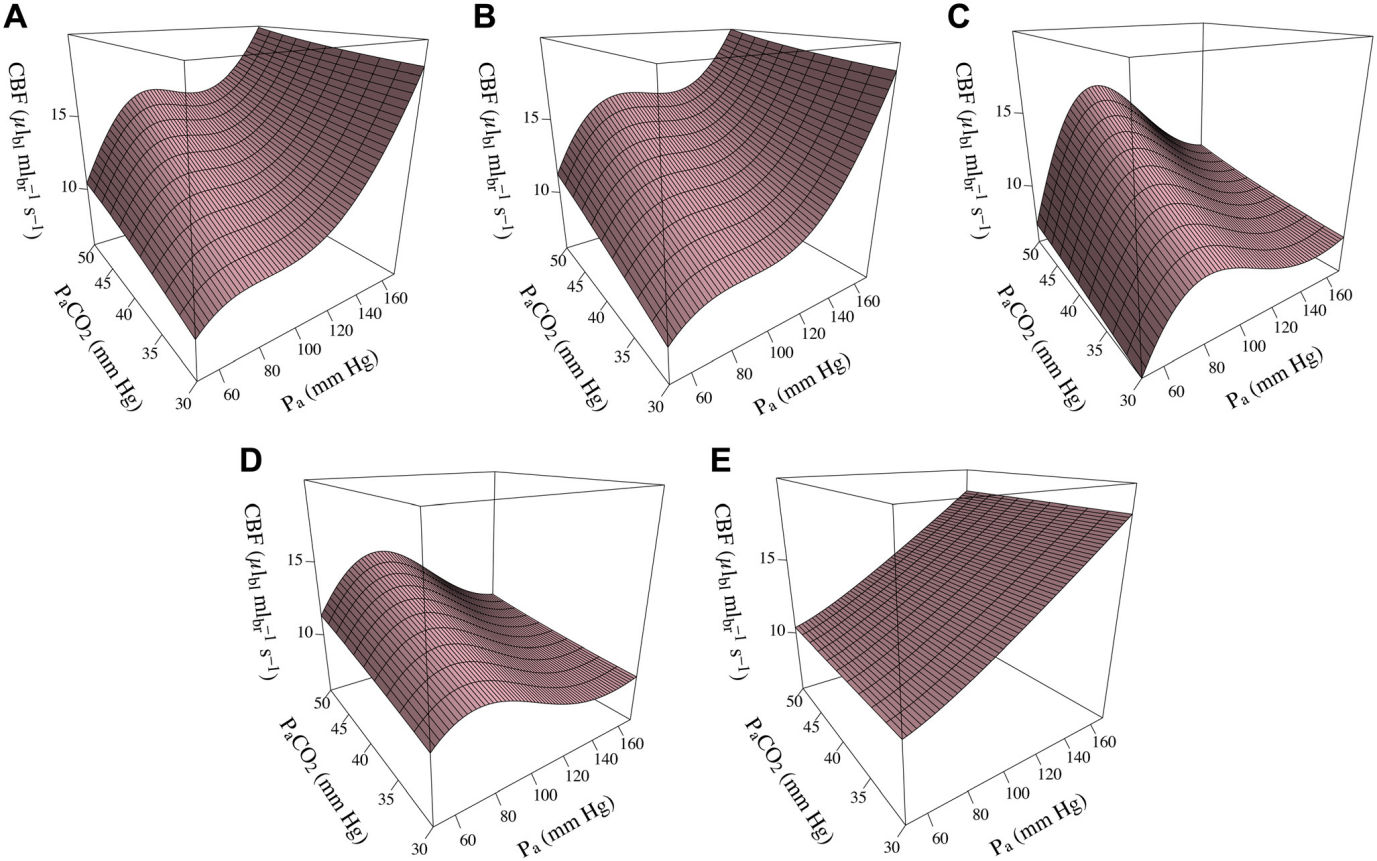
Interaction between blood pressure autoregulation and CO_2_. Steady state simulations of blood flow with varying levels of both arterial blood pressure (*P_a_*) and partial pressure of carbon dioxide (*P_a_CO_2_*). Identical inputs were used with each model. **A** BrainSignals. **B** B1. **C** B2. **D** B3. **E** B4. It is evident that only blood flow variant B1, which includes an explicit interaction term for both $\tilde{\mu }$ and $\frac{1}{P_{a}}$, is able to reproduce the behaviour seen in the original model.

We can further investigate this by considering the interaction between blood pressure autoregulation and the other regulatory factors. Although all the stimuli in BrainSignals contribute to the single term *η*, blood pressure also participates in the elastic tension response. Interaction can therefore occur in the model, as it is known to do physiologically. This is illustrated in Fig 8A, which shows the BrainSignals steady state for CBF with variations in both P_a_ and P_a_CO_2_. Corresponding results for the simplified models are shown in panels B–E of Fig 8. It can be seen that only the blood flow variant B1 is able to reproduce this behaviour, confirming that the interaction term is needed.

### Metabolism

As might be suspected from the graphs in Fig 6, our linear models readily fitted the data, and all three variants M1–M3 explained the variance well, with *R* ^2^ > 0.99.

**Fig 9:**
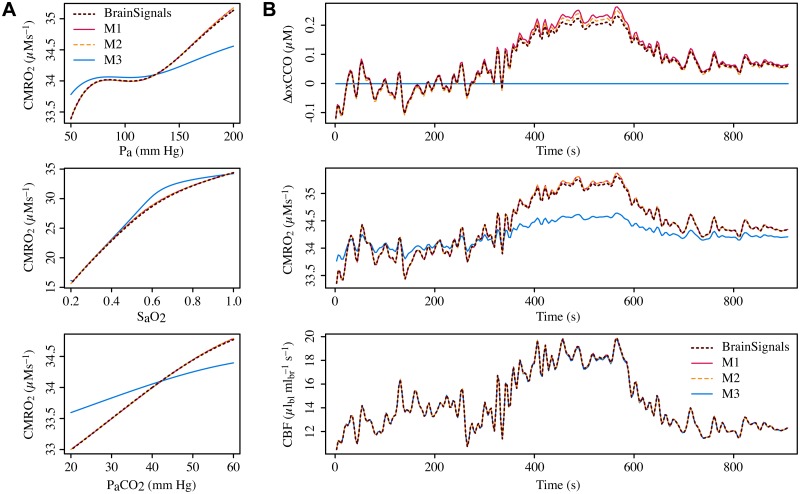
Comparing behaviour of simplified metabolic submodels to BrainSignals. **A** Steady state simulations of CMRO_2_ for different levels of arterial blood pressure (*P_a_*), oxygen saturation (*S_a_O_2_*) and partial pressure of carbon dioxide (*P_a_CO_2_*). Model variants M1 and M2 reproduce the BrainSignals behaviour closely, whereas M3 exhibits substantial deviations. **B** Time courses of model outputs driven by real experimental data. The three plots show values simulated from the same adult hypercapnia data as in Fig 7. For estimation of some output values, such as CBF (bottom), behaviour is dominated by the blood flow submodel and the impact of swapping the metabolic submodels is negligible. More metabolically-relevant outputs such as CMRO_2_ and ΔoxCCO again show good correspondence for variants M1 and M2, while M3’s behaviour is very poor. (Average output distances for simulations from all experimental subjects are given in Table 3.)

Once again BrainSignals was compared with models in which the metabolic variants were substituted, keeping the remaining components the same. Steady state plots are shown in Fig 9A, using the metabolically-relevant output value CMRO_2_ rather than CBF. (The latter is dominated by the unchanged blood flow submodel and so does not satisfactorily reveal behavioural differences between the variants.) The steady state values for model variants M1 and M2 are essentially identical to those for the original BrainSignals metabolic model. By contrast, variant M3 diverges markedly, suggesting that the unification of rates is a bad choice of approximation.

This is borne out by the evidence of dynamic simulations (Fig 9B). Again, many model outputs are not strongly affected by changes to the metabolic submodel, since their behaviour is almost entirely determined by the blood flow model. An example of CBF output is shown in the lowest panel. The failure of variant M3 is obvious, however, in metabolically-relevant outputs. Importantly, the ΔoxCCO prediction (representing changes in CCO oxidation state) is completely abolished—if oxidation and reduction always occur at the same rate, the level of oxidised CCO cannot change—rendering M3 useless for this NIRS quantity of interest. Variants M1 and M2 again follow BrainSignals behaviour closely, with M2 producing slightly better results. As it is also simpler and performs equivalently at steady state, it would appear to be the best metabolic substitute of those presented here.

### Combined variants

**Fig 10:**
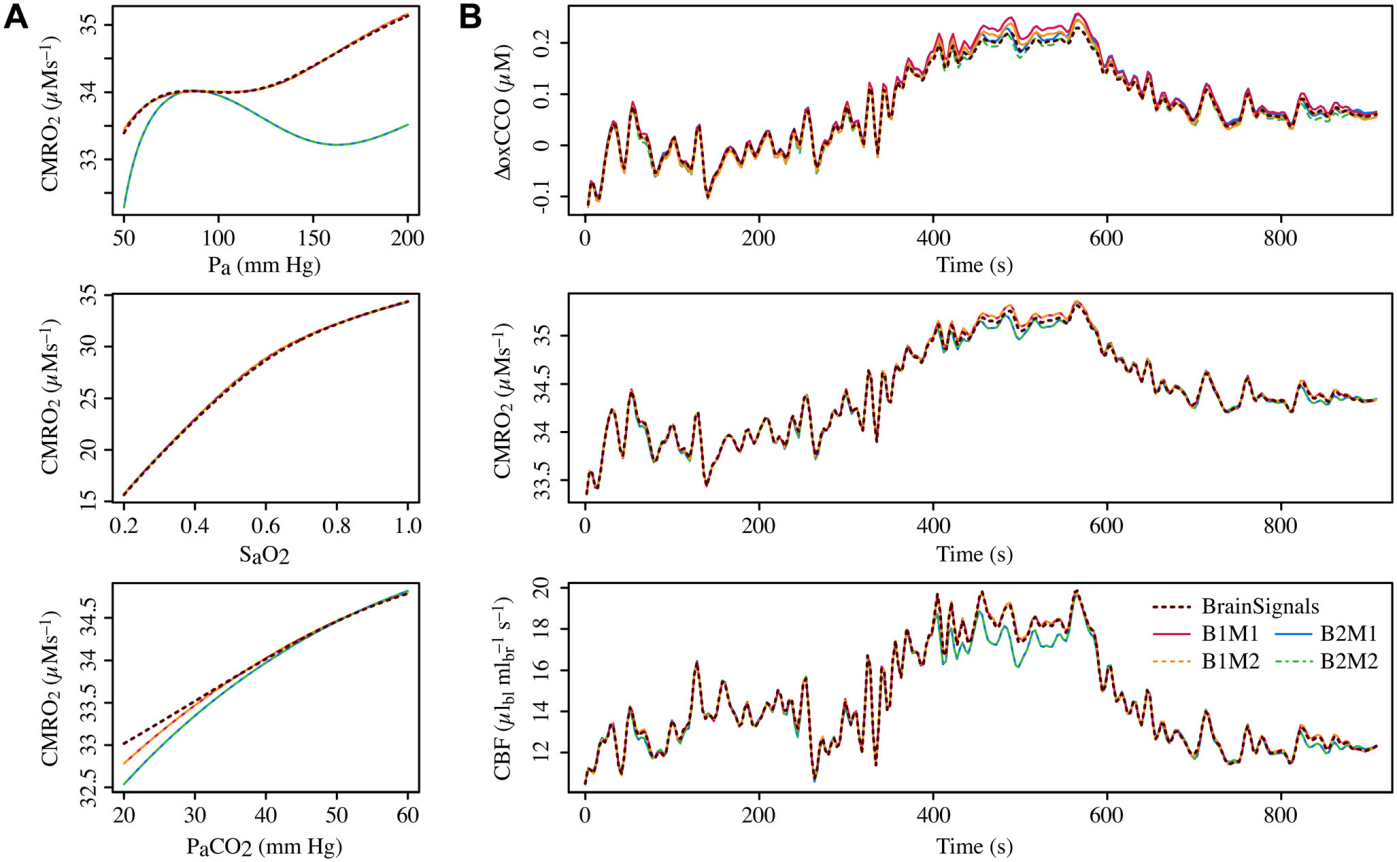
Comparing submodel combinations. **A** Steady state simulations of CMRO_2_ for different levels of arterial blood pressure (*P_a_*), oxygen saturation (*S_a_O_2_*) and partial pressure of carbon dioxide (*P_a_CO_2_*). Behaviour is dominated by the blood flow submodel, with different metabolic submodels producing no discernible effect. As in the isolated tests, variant B1 is clearly superior for *P_a_* autoregulation. **B** Time courses of model outputs driven by real experimental data. The three plots show values simulated from the same adult hypercapnia data as in previous figures. All models produce rather similar dynamic results. CBF and CMRO_2_ are both largely determined by blood flow. ΔoxCCO varies also with choice of metabolic submodel, although the deviations are generally small. (Average output distances for simulations from all experimental subjects are given in Table 3.)

Having tested the variant models individually, we proceeded to check their behaviour in combination. For these purposes variants B3, B4 and M3 were discarded, since they had already proved inadequate substitutes. Simulations from the remaining submodel combinations are shown in Fig 10. It is evident from the steady state results in panel Fig 10A that, even using a metabolic output signal like CMRO_2_, the blood flow submodel dominates behaviour. In all three plots, the differences between the models with metabolic variant M1 and their counterparts with M2 are negligible.

For dynamic simulations, the situation is similar. The blood flow submodels again dominate the results for most outputs. Unsurprisingly, this is best seen in the CBF trace, but even here the distinction is less pronounced than for the steady state simulations. Differences between the metabolic submodels are most apparent in the ΔoxCCO output, which is directly affected by the rate constant values. Consistent with the tests of the metabolic models in the previous section, the simpler variant M2 reproduces the BrainSignals behaviour slightly better than the more complex M1. Overall, the B1M2 combination is the most successful simplified version.

For comparison, Table 4 lists the relative sizes of the combined model variants, along with estimates of the average improvement in execution speed.

**Table 4:** Relative sizes of the different variants of the full model.

Model	State Variables	Parameters	Temporary Variables	Speed Increase (%)
BrainSignals	12	135	40	4
B1M1	12	70	22	17
B1M2	12	69	22	19
B2M1	12	69	22	18
B2M2	12	68	22	19

Execution speed increases are calculated relative to a direct translation of the original BrainSignals model into the BCMD framework. Thus, some improvements are seen from refactoring and optimisation even with no model reduction. Values were averaged over 1400 simulations for each model (100 repetitions for each of 10 sets of volunteer input data and 4 steady state simulations).

### Sensitivity analysis

Sensitivity analyses were performed to compare the parameter dependencies of the original model with those of the best simplified variant, B1M2. The models were driven with inputs recorded from 10 healthy adults undergoing a hypercapnia challenge. The sensitivities were calculated for each subject using the eFAST method [55], which estimates both a first order sensitivity index (how much a parameter affects model output when varied alone) and a total sensitivity index (how influential the parameter is when interactions with all other parameters are considered). Simulated outputs were compared against real experimental signals recorded by NIRS and Trans-Cranial Doppler, using Euclidean distance as the measure of effect.

**Fig 11:**
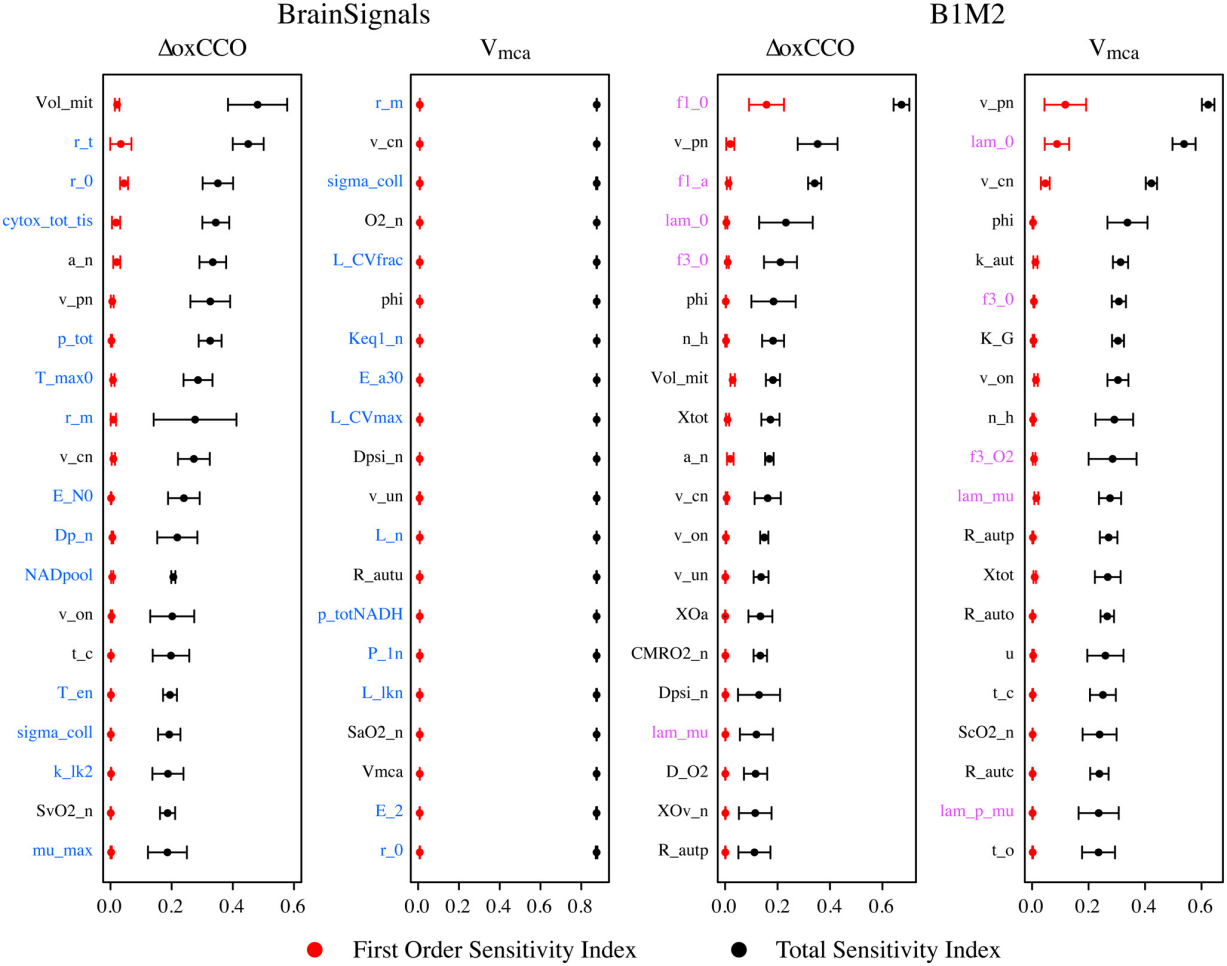
Sensitivity comparison between BrainSignals and the combined variant model B1M2. Results are shown for the metabolic output ΔoxCCO and the haemodynamic output V_mca_. Extended Fourier amplitude sensitivity tests (eFAST) [55] were performed using input data recorded from 10 healthy adults undergoing a hypercapnia challenge. Plotted points represent the median values across all 10 data sets, while error bars show the median absolute deviation, indicating variability between subjects. Parameter names written in black are shared by both models, while those in blue are specific to BrainSignals and those in purple to B1M2. Definitions of all parameters can be found in S1 Text.

Results are shown in Fig 11 for the 20 most influential parameters on the output signals ΔoxCCO and V_mca_ (mean blood velocity in the middle cerebral artery, a measure of blood flow) for both models over all 10 volunteer data sets.

The simplified model has fewer parameters and its sensitivity distribution is compressed, with more influence aggregated in the lumped parameters of the fitted linear subsystems. The sensitivity distributions are skewed and first order effects much smaller than total, suggesting that the models remain ‘sloppy’. Many parameters are not well aligned with the dimensions of behavioural variability.

It is notable that the BrainSignals V_mca_ output variance is especially poorly attributed to any individual parameter. Many of the parameters affecting this behaviour are evidently not identifiable. However, this is also in part due to degeneracy of the parameter space. There are many infeasible combinations of these parameters, leading to failed simulations and inadequate sampling of individual contributions. Once again, by compressing the parameter space, the simplified model reduces this problem and improves identifiability, though it cannot solve the problem completely.

## Discussion

### Simplified models can reproduce the behaviour of BrainSignals

We have shown that it is possible to mimic the behaviour of one physiological model, BrainSignals, by replacing some of its more intractable subsystems with simpler approximations. The simplification is pragmatic, and we have prioritised behavioural similarity over biophysical correctness. Nevertheless, the model is not simply a black-box, as might be obtained by training an artificial neural network or similar machine learning model [71]. We retain the overall model structure, state variables and key relationships, because they represent features of the system that we are interested in.

The ability to obtain similar behaviour in simpler ways suggests that some of the complexity in the full model is superfluous. While that may sometimes be true, it depends on the application for which the model is intended. Where a model detail relates to instrumental outputs or therapeutic interventions, then its inclusion can be important. Even discovering that it does not contribute significantly may itself be a valuable result. However, where complex models with many difficult-to-measure parameters give rise to simple behaviour, it can make sense to model the behaviour directly rather than being distracted by misleading precision.

In the case of BrainSignals, the ability to construct a smaller and more tractable model that still captures important aspects of the problem structure opens up new application opportunities. For example, we are investigating the possibility of running the simplified model in near-real time in an embedded system environment, providing dynamically-updated information to clinicians to assist the interpretation of complex datasets and aid clinical decision making.

### Validity

The simplification of BrainSignals raises concerns of validity in two key ways.

First, the abstraction away from mechanisms to behaviour risks a loss of explanatory power. If the model only replicates observable features of the system without telling us anything about the internal state, then it is of little value. Our simplified models preserve the state variables, but they omit numerous details of the internal processes. We may be discarding information that could contribute to the physiological interpretation. However, these details were removed in part because their intractability made them difficult to interpret. Since the system evolution is captured by the state variables, it would in principle be possible to reconstruct the other details from them. That would, of course, entail the same problems of parameterisation as were present in the original model.

The second, and more serious, concern is that a simplified model might fail empirically. If the assumptions used to make the simplifications are violated, then the behaviour of the model is likely to be wrong. In particular, even though it adequately reproduces cases similar to those from which it was fitted, it may fail in unanticipated scenarios. This would be clinically relevant because the injured brain is, almost by definition, in abnormal physiological states with marked regional and temporal heterogeneity.

Eliminating detail from the model can certainly result in loss of behaviour. Several of our candidate models exhibit such loss, notably metabolic variant M3, in which the CCO signal is abolished, and blood flow variant B4, which does not autoregulate. We cannot guarantee that important behaviour has not also been lost from the apparently more viable variants.

On the other hand, we also cannot guarantee that the original model behaviour was correct in all cases—in fact, we can be confident it was not. Any model can exhibit questionable behaviour, especially at the edge cases where the underlying assumptions fail or the parameter uncertainties come into play. Such cases proliferate with model complexity. The BrainSignals behaviour space is complex and fragile, with many degenerate regions. This is less true of the reduced models, because several loci of complexity have been removed. There is thus a trade-off between detail and stability, with no single correct point of balance in between.

Modelling complex biological systems always requires compromises, so it is important to ensure that the assumptions made are taken into account when the model is used. This is true for the simplified variants just as for BrainSignals and its predecessors. We can identify some limitations *a priori*, when the corresponding model features have been explicitly removed. For example, none of the simplified blood flow variants would be suitable for applications requiring estimation of the blood vessel muscular tension. However, a more important class of errors are those that cannot be easily predicted. These can only be assessed by testing the model against a wide range of inputs, and being cautious about using it in situations very different from those tested. At present the models have been tested with a range of synthetic inputs, but with physiological data only from healthy adults. Extensive testing with data from patients with a range of different pathologies is necessary before the model could be applied in a clinical setting.

A further possible objection to the simplification involves the collapsed parameters. Even if the behaviour is correct, these parameters are divorced from reality and may not provide useful information. We can fit generic values that give the desired ‘normal’ behaviour, but we cannot relate these to experimental measurements, because they do not correspond to real physical quantities. Similarly, we can fit the model to experimental or clinical data to obtain estimates of such parameters for an individual, but the results may not have a useful interpretation.

Once again, context is paramount. In situations where the missing detail is important, the simplified models cannot be used. However, given the uncertainties attendant upon parameter estimates from ‘sloppy’ models [59], it may be unwise to attach too much significance to them in any case. We have previously found that attempts to explain observations through individual parameter fitting can lead to unrealistic predictions [72, 73]. Using simpler models should allow for a more coherent overall picture, at the cost of a loss of detail.

### Generalisation

While our simplification approach worked for BrainSignals, it is not clear that it generalises. We expect there will be *some* wider applicability, but it will vary for different models. We cannot make strong assertions without more empirical testing, but we offer the following observations.

The initial decomposition step was useful in the case of BrainSignals because the model was already a composite, drawing on elements of previous models to inform different functional compartments. Aggregation of interacting subsystems is common in Systems Biology [74], and such models often have an intuitive decomposition. For models that do not, it is unlikely that imposing an arbitrary division will be productive.

Algebraic simplification and code refactoring provide obvious benefits, but both are often neglected in practice. The multi-scale, multi-disciplinary nature of Systems Biological modelling can encourage compartmentalisation rather than simplification. The language, units and conventions appropriate for one part of a model may differ significantly from those employed elsewhere. It is often beneficial to use a representation that accords with the ‘local language’ of the modelled domain. On the other hand, rationalising calculations across these conceptual boundaries can identify redundancies and lead to more efficient and comprehensible models. This is especially so when considered in conjunction with the lumping of parameters—the identification of parameters that can usefully be collapsed together is easier when they are expressed in commensurable terms.

Large and complex models typically build on previous code rather than implement everything from scratch. There are obvious benefits to such an approach, and improving the capacity for model reuse has been an important topic of research in recent years [75–77]. However, legacy implementations can also include details that were relevant for a previous purpose but have since been obviated, or relics of modelling strategies that failed, or features that people now working on the code do not fully understand and are reluctant to alter. Identifying and eliminating such redundancies demands a significant effort, for which there is typically little incentive.

BrainSignals is conceptually simple enough for manual identification and fitting of simplified subsystems to be practical. Generalising the process to larger and more highly-connected models may prove challenging. The choice of approximating functions is dependent on the characteristics of the model, but the fitting process should be as transparent as possible. This is an advantage of using linear models—there is no confusion as to what is actually being fitted. Using a ‘black box’ function such as a neural network or kernel method [71] risks merely shifting the complexity from an explicit and visible site to an implicit, hidden one.

### Conclusion

The approach outlined here has allowed us to create simpler versions of our model BrainSignals, and may be applicable to other physiological models. Simplification makes the model more tractable and comprehensible, and reduces the number of parameters that need to be obtained or estimated. By compressing the parameter space, it may also improve the alignment between the parameters and the behavioural variability in the model.

However, simplification also risks loss of usefulness because of the reduced detail. The linearity assumptions underlying the simplification may not hold in other physiological conditions. The applicability of reduced models to any particular problem must be considered with care. The goal is always to improve physiological understanding and aid interpretation. Our simplified models do not supplant the original model or others of even greater complexity that may be necessary in other contexts—the very process of model reduction implies a prior requirement for *un*-reduced models. Rather they expand the range of tools available.

## Supporting Information

S1 Text Model Definitions. Specification of all model variants used, including the defining equations, reactions and parameter values.
(PDF)

S1 Fig Structure of BrainSignals blood flow submodel. (PDF)

S2 Fig Structure of simplified blood flow variant B1. (PDF)

S3 Fig Structure of BrainSignals metabolic submodel. (PDF)

S4 Fig Structure of simplified metabolic variant M0. (PDF)

S5 Fig Structure of simplified metabolic variant M1. (PDF)

S6 Fig Structure of simplified metabolic variant M2. (PDF)

S7 Fig Structure of simplified metabolic variant M3. (PDF)
