# Supplementary material for: BrainSignals Revisited: Simplifying a Computational Model of Cerebral Physiology
Source: PLoS One. 2015 May 11;10(5):e0126695. doi: 10.1371/journal.pone.0126695 (PMC4427507; doi:10.1371/journal.pone.0126695)
Supplement: S1 Text — Specification of all model variants used, including the defining equations, reactions and parameter values. (PDF) [file pone.0126695.s001.pdf]

Supplementary Model Details

BrainSignals Revisited:
simplifying a computational model of cerebral physiology

Matthew Caldwell, Tharindi Hapuarachchi, David Highton, Clare Elwell, Martin Smith, Ilias Tachtsidis

(Model documentation generated by
BCMD module `doc_latex.py`)

Contents

1 BrainSignals **7**
1.1 Overview 7
1.2 Differential Equations 7
1.3 Algebraic Equations 8
1.4 Chemical Reactions 8
1.5 State Variables 8
1.6 Intermediate Variables 10
1.7 Parameters 14

2 BSB1 **29**
2.1 Overview 29
2.2 Differential Equations 29
2.3 Algebraic Equations 30
2.4 Chemical Reactions 30
2.5 State Variables 30
2.6 Intermediate Variables 32
2.7 Parameters 35

3 BSB2 **49**
3.1 Overview 49
3.2 Differential Equations 49
3.3 Algebraic Equations 50
3.4 Chemical Reactions 50
3.5 State Variables 50
3.6 Intermediate Variables 52
3.7 Parameters 55

4 BSB3 **69**
4.1 Overview 69
4.2 Differential Equations 69
4.3 Algebraic Equations 70
4.4 Chemical Reactions 70
4.5 State Variables 70
4.6 Intermediate Variables 72
4.7 Parameters 75

5 BSB4 **89**
5.1 Overview 89
5.2 Differential Equations 89
5.3 Algebraic Equations 90
5.4 Chemical Reactions 90
5.5 State Variables 90
5.6 Intermediate Variables 92
5.7 Parameters 95

6 BSM0 **109**
6.1 Overview 109
6.2 Differential Equations 109

6.3 Algebraic Equations . . . 110
6.4 Chemical Reactions . . . 110
6.5 State Variables . . . 110
6.6 Intermediate Variables . . . 112
6.7 Parameters . . . 114

7 BSM1 **125**
7.1 Overview . . . 125
7.2 Differential Equations . . . 125
7.3 Algebraic Equations . . . 126
7.4 Chemical Reactions . . . 126
7.5 State Variables . . . 126
7.6 Intermediate Variables . . . 128
7.7 Parameters . . . 130

8 BSM2 **141**
8.1 Overview . . . 141
8.2 Differential Equations . . . 141
8.3 Algebraic Equations . . . 142
8.4 Chemical Reactions . . . 142
8.5 State Variables . . . 142
8.6 Intermediate Variables . . . 144
8.7 Parameters . . . 146

9 BSM3 **157**
9.1 Overview . . . 157
9.2 Differential Equations . . . 157
9.3 Algebraic Equations . . . 158
9.4 Chemical Reactions . . . 158
9.5 State Variables . . . 158
9.6 Intermediate Variables . . . 160
9.7 Parameters . . . 162

10 B1M1 **173**
10.1 Overview . . . 173
10.2 Differential Equations . . . 173
10.3 Algebraic Equations . . . 174
10.4 Chemical Reactions . . . 174
10.5 State Variables . . . 174
10.6 Intermediate Variables . . . 176
10.7 Parameters . . . 178

11 B1M2 **187**
11.1 Overview . . . 187
11.2 Differential Equations . . . 187
11.3 Algebraic Equations . . . 188
11.4 Chemical Reactions . . . 188
11.5 State Variables . . . 188
11.6 Intermediate Variables . . . 190
11.7 Parameters . . . 192

12 B2M1 **201**
12.1 Overview . . . 201
12.2 Differential Equations . . . 201
12.3 Algebraic Equations . . . 202
12.4 Chemical Reactions . . . 202
12.5 State Variables . . . 202

12.6 Intermediate Variables 204
12.7 Parameters 206

13 B2M2 **215**
13.1 Overview 215
13.2 Differential Equations 215
13.3 Algebraic Equations 216
13.4 Chemical Reactions 216
13.5 State Variables 216
13.6 Intermediate Variables 218
13.7 Parameters 220

1 BrainSignals

1.1 Overview

The full BrainSignals model, with a refactored implementation.

- 9 differential state variables
- 3 algebraic state variables
- 40 intermediate variables
- 139 parameters
- 4 declared inputs
- 33 default outputs

1.2 Differential Equations

$$\frac{\mathrm{d}Cu_{A,o}}{\mathrm{d}t} = 4f_3 - 4f_1 \tag{1.1}$$

$$\frac{\mathrm{d}a_{3,r}}{\mathrm{d}t} = 4f_3 - 4f_3 \tag{1.2}$$

$$\frac{\mathrm{d}\psi}{\mathrm{d}t} = \frac{p_3\, f_3 + p_1\, f_1 + p_3\, f_3 - L}{C_{im}} \tag{1.3}$$

$$\frac{\mathrm{d}H^+}{\mathrm{d}t} = \frac{1}{R_{Hi}}\, L - \frac{p_3}{R_{Hi}}\, f_3 - \frac{p_1}{R_{Hi}}\, f_1 - \frac{p_3}{R_{Hi}}\, f_3 \tag{1.4}$$

$$\frac{\mathrm{d}O_2}{\mathrm{d}t} = \frac{1}{Vol_{mit}}\, J_{O_2} - f_3 \tag{1.5}$$

$$\frac{\mathrm{d}v_{CO_2}}{\mathrm{d}t} = \frac{1}{\tau_{CO_2}}\, \left(Pa_{CO_2} - v_{CO_2}\right) \tag{1.6}$$

$$\frac{\mathrm{d}v_{O_2}}{\mathrm{d}t} = \frac{1}{\tau_{O_2}}\, \left(O_{2,c} - v_{O_2}\right) \tag{1.7}$$

$$\frac{\mathrm{d}v_{P_a}}{\mathrm{d}t} = \frac{1}{\tau_{P_a}}\, \left(P_a - v_{P_a}\right) \tag{1.8}$$

$$\frac{\mathrm{d}\nu_u}{\mathrm{d}t} = \frac{1}{\tau_u}\,(u - \nu_u) \tag{1.9}$$

1.3 Algebraic Equations

$$\phi\left(\frac{S_{c,O_2}}{1 - S_{c,O_2}}\right)^{\frac{1}{n_h}} - O_{2,c} = 0 \tag{1.10}$$

$$T_e + T_m - (P_1 - P_{ic})\,r = 0 \tag{1.11}$$

$$CBF\,(HbO_{2,a} - HbO_{2,v}) - J_{O_2} = 0 \tag{1.12}$$

1.4 Chemical Reactions

$$\xrightarrow{L} \frac{1}{R_{Hi}}\,\mathrm{H^+} \tag{1.13}$$

$$\xrightarrow{J_{O_2}} \frac{1}{Vol_{mit}}\,\mathrm{O_2} \tag{1.14}$$

$$\frac{p_3}{R_{Hi}}\,\mathrm{H^+} \xrightarrow{f_3} 4\,\mathrm{Cu_{A,o}} + 4\,\mathrm{a_{3,r}} \tag{1.15}$$

$$4\,\mathrm{Cu_{A,o}} + \frac{p_1}{R_{Hi}}\,\mathrm{H^+} \xrightarrow{f_1} \tag{1.16}$$

$$\mathrm{O_2} + 4\,\mathrm{a_{3,r}} + \frac{p_3}{R_{Hi}}\,\mathrm{H^+} \xrightarrow{f_3} \tag{1.17}$$

1.5 State Variables

$Cu_{A,o}$
Implementation Name: `a`
Units: mM
Initial value: $Cu_{A,o,n}$
Concentration of oxidised cytochrome c oxidase.

$a_{3,r}$
Implementation Name: `bred`
Units: mM
Initial value: $a_{3,r,n}$
Concentration of reduced cytochrome $\mathrm{a_3}$.

ψ

Implementation Name: `Dpsi`
Units: mV
Initial value: ψ_n
Mitochondrial inner membrane potential. Varies as charge (in the form of protons) is transferred across the membrane capacitance.

H^+

Implementation Name: `H`
Units: mM
Initial value: H_n^+
Mitochondrial proton concentration.

O_2

Implementation Name: `O2`
Units: mM
Initial value: $O_{2,n}$
Mitochondrial oxygen concentration.

$O_{2,c}$

Implementation Name: `O2c`
Units: mM
Initial value: $O_{2,c,n}$
Capillary oxygen concentration.

r

Implementation Name: `r`
Units: cm
Initial value: r_n
Typical blood vessel radius.

ν_{CO_2}

Implementation Name: `v_c`
Units: mmHg
Initial value: $\nu_{CO_2,n}$
Filtered carbon dioxide partial pressure.

ν_{O_2}

Implementation Name: `v_o`
Units: mM
Initial value: $\nu_{O_2,n}$
Filtered capillary oxygen concentration.

ν_{P_a}

Implementation Name: `v_p`
Units: mmHg
Initial value: $\nu_{P_a,n}$
Filtered arterial blood pressure.

ν_u

Implementation Name: `v_u`
Units: dimensionless
Initial value: $\nu_{u,n}$
Filtered demand.

$HbO_{2,v}$

Implementation Name: `XOv`
Units: mM
Initial value: $HbO_{2,v,n}$
Venous concentration of oxygen bound to haemoglobin.

1.6 Intermediate Variables

$Cu_{A,r} = CCO_{tot} - Cu_{A,o}$
Implementation Name: `ared`
Units: mM
Initial value: 0
Concentration of reduced Cu_A.

$a_{3,o} = CCO_{tot} - a_{3,r}$
Implementation Name: `b`
Units: mM
Initial value: 0
Concentration of oxidised cytochrome a_3.

$$C_{0,i} = \frac{10^{-pH_m} - 10^{-pH_m - dpH}}{dpH}$$
Implementation Name: `C_0i`
Units: dimensionless
Initial value: 0
Natural buffering capacity of protons in mitochondria.

$CBF = G\ (P_a - P_v)$
Implementation Name: `CBF`
Units: $ml_{blood}\ ml_{brain}^{-1}\ s^{-1}$
Initial value: CBF_n
Cerebral blood flow.

$\Delta oxCCO = \Delta oxCCO_{off} + 1000 Vol_{mit}\ (Cu_{A,o} - Cu_{A,o,n})$
Implementation Name: `CCO`
Units: uM
Initial value: 0
Cytochrome c oxidase signal measured by NIRS.

$CMRO_2 = f_3\, Vol_{mit}$
Implementation Name: `CMRO2`
Units: mM s^{-1}
Initial value: 0
Rate of cerebral oxygen metabolism.

$\Delta p = \psi + Z\ (pH_m - pH_o)$
Implementation Name: `Dp`
Units: mV
Initial value: 0
Proton motive force across the mitochondrial inner membrane.

$$\eta = R_{P_a}\left(\frac{v_{P_a}}{v_{P_a,n}} - 1\right) + R_{O_2}\left(\frac{v_{O_2}}{v_{O_2,n}} - 1\right) + R_{CO_2}\left(1 - \frac{v_{CO_2}}{v_{CO_2,n}}\right) + R_u\left(1 - \frac{v_u}{v_{u,n}}\right)$$
Implementation Name: `eta`
Units: dimensionless
Initial value: 0
Merged autoregulation stimulus.

$f_1 = k_1\, Cu_{A,o} - k_{-1}\, Cu_{A,r}$
Implementation Name: `f1`
Units: mM s^{-1}
Initial value: 0
Reaction rate for the reduction of Cu_A.

$f_3 = k_2\, Cu_{A,r}\, a_{3,o} - k_{-2}\, Cu_{A,o}\, a_{3,r}$
Implementation Name: `f2`

Units: mM s^{-1}
Initial value: 0
Reaction rate for the reduction of a_3.

$$f_3 = \frac{k_3\, O_2\, a_{3,r} \exp\left(-c_3\ (\Delta p - \Delta p_{30})\right)}{1 + \exp\left(-c_3\ (\Delta p - \Delta p_{30})\right)}$$

Implementation Name: `f3`
Units: mM s^{-1}
Initial value: 0
Reaction rate for the reduction of O_2.

$$G = K_G\, r^4$$

Implementation Name: `G`
Units: $ml_{blood}\ ml_{brain}^{-1}$ $mmHg^{-1}$ s^{-1}
Initial value: 0
Effective conductance of the whole blood flow compartment.

$$h = \mathrm{sqrt}\left(r\, r + 2 r_0\, h_0 + h_0\, h_0\right) - r$$

Implementation Name: `h`
Units: cm
Initial value: h_n
Thickness of the blood vessel walls.

$$HbO_2 = \left(V_a\, HbO_{2,a} + V_v\, HbO_{2,v}\right)\ blood_{hb}$$

Implementation Name: `HbO2`
Units: uM
Initial value: 0
Oxygenated haemoglobin signal measured by NIRS.

$$HbT = \left(V_a + V_v\right)\ Hb_{tot}\, blood_{hb}$$

Implementation Name: `HbT`
Units: uM
Initial value: 0
Total haemoglobin signal measured by NIRS.

$$HHb = HbT - HbO_2$$

Implementation Name: `HHb`
Units: uM
Initial value: 0
Deoxygenated haemoglobin signal measured by NIRS.

$$J_{O_2} = \mathrm{fmin}\left(D_{O_2}\ \left(O_{2,c} - O_2\right), CBF\, HbO_{2,a}\right)$$

Implementation Name: `J_O2`
Units: mM s^{-1}
Initial value: 0
Oxygen flux from blood to tissue.

$$k_1 = k_{1,0} \exp\left(-c_{k_1}\ (\Delta p - \Delta p_n)\right)$$

Implementation Name: `k1`
Units: s^{-1}
Initial value: 0
Forward reaction rate for the reduction of Cu_A.

$$k_2 = k_{2,n} \exp\left(-c_{k_2}\ (\Delta p - \Delta p_n)\right)$$

Implementation Name: `k2`
Units: s^{-1}
Initial value: 0
Forward reaction rate for the reduction of a_3.

$$K_{eq_1} = 10^{\frac{-1}{Z}\left(\frac{p_1\, \Delta p}{4} - E_1\right)}$$

Implementation Name: `Keq1`
Units: dimensionless
Initial value: 0
Equilibrium constant for the Cu_A reduction reaction.

$K_{eq_2} = 10^{\frac{-1}{Z}\left(\frac{p_3 \Delta p}{4} - E_2\right)}$
Implementation Name: `Keq2`
Units: dimensionless
Initial value: 0
Equilibrium constant for the a_3 reduction reaction.

$k_{-1} = \frac{k_1}{K_{eq_1}}$
Implementation Name: `kn1`
Units: s^{-1}
Initial value: 0
Reverse reaction rate for the reduction of Cu_A.

$k_{-2} = \frac{k_2}{K_{eq_2}}$
Implementation Name: `kn2`
Units: s^{-1}
Initial value: 0
Reverse reaction rate for the reduction of a_3.

$L = L_{CV} + L_{lk}$
Implementation Name: `L`
Units: mM s^{-1}
Initial value: 0
Rate of proton return to the mitochondrial matrix.

$L_{CV} = \frac{CV_{inh} \, L_{CV,max} \, (1 - \exp(-\theta))}{1 + r_{CV} \exp(-\theta)}$
Implementation Name: `L_CV`
Units: mM s^{-1}
Initial value: 0
Rate at which protons re-enter the mitochondrial matrix due to ADP phosphorylation.

$L_{lk} = k_{unc} \, L_{lk0} \, (\exp(\Delta p \, k_{lk2}) - 1)$
Implementation Name: `L_lk`
Units: mM s^{-1}
Initial value: 0
Rate at which protons re-enter the mitochondrial matrix via leak channels.

$\mu = \frac{\mu_{min} + \mu_{max} \exp(\eta)}{1 + \exp(\eta)}$
Implementation Name: `mu`
Units: dimensionless
Initial value: 0
Effective strength of the autoregulation reponse.

$pH_m = -\log10\left(\frac{H^+}{1000}\right)$
Implementation Name: `pH_m`
Units: dimensionless
Initial value: 0
Mitochondrial pH.

$r_{buffi} = \frac{C_{buffi}}{C_{0,i}}$

Implementation Name: `r_buffi`
Units: dimensionless
Initial value: 0
Buffering capacity for protons in mitochondria.

$R_{Hi} = r_{buffi}$
Implementation Name: `R_Hi`
Units: dimensionless
Initial value: 0
Relative mitochondrial volume for protons, taking into account buffering effect of pH.

$S_{c,O_2} = \frac{S_{a,O_2} + S_{v,O_2}}{2}$
Implementation Name: `ScO2`
Units: dimensionless
Initial value: $S_{c,O_2,n}$
Capillary oxygen saturation.

$\sigma_e = \sigma_{e,0} \left(\exp\left(\frac{K_\sigma \, (r - r_0)}{r_0} \right) - 1 \right) - \sigma_{coll}$
Implementation Name: `sigma_e`
Units: mm Hg
Initial value: 0
Elastic stress in blood vessel walls.

$S_{v,O_2} = \frac{HbO2_{,v}}{Hb_{tot}}$
Implementation Name: `SvO2`
Units: dimensionless
Initial value: $S_{v,O_2,n}$
Venous oxygen saturation.

$T_e = \sigma_e \, h$
Implementation Name: `T_e`
Units: mm Hg cm
Initial value: 0
Elastic tension in the blood vessel walls.

$T_m = T_{max} \exp\left(-\mathrm{pow}\left(\mathrm{fabs}\left(\frac{r - r_m}{r_t - r_m} \right), n_m \right) \right)$
Implementation Name: `T_m`
Units: mm Hg cm
Initial value: 0
Muscular tension in the blood vessel walls.

$T_{max} = T_{max,0} \, (1 + k_{aut} \, \mu)$
Implementation Name: `T_max`
Units: mm Hg cm
Initial value: 0
Maximal muscular tension in the blood vessel walls.

$\theta = k_{CV} \, (\Delta p + Z \log10 (u) - \Delta p_{CV,0})$
Implementation Name: `theta`
Units: dimensionless
Initial value: 0
Driving force Complex V.

$TOI = \frac{100 HbO_2}{HbT}$
Implementation Name: `TOI`
Units: dimensionless

Initial value: 0
Total oxygenation index.

V_{mca} $= CBF\, CBFscale$
Implementation Name: `Vmca`
Units: cm s^{-1}
Initial value: 0
Blood velocity in the middle cerebral artery.

V_a $= V_{a,n} \left(\frac{r}{r_n}\right)^2$
Implementation Name: `Vol_art`
Units: dimensionless
Initial value: 0
Relative arterial blood volume.

1.7 Parameters

$Cu_{a,frac,n}$
Implementation Name: `a_frac_n`
Units: dimensionless
Initial value: 0.8
Normal oxidised fraction of Cu_A.

$Cu_{A,o,n}$
Implementation Name: `a_n`
Units: mM
Initial value: $CCO_{tot}\, Cu_{a,frac,n}$
Normal concentration of oxidised cytochrome c oxidase.

$Cu_{A,r,n}$
Implementation Name: `ared_n`
Units: mM
Initial value: $CCO_{tot} - Cu_{A,o,n}$
Normal concentration of reduced Cu_A.

$a_{3,o,n}$
Implementation Name: `b_n`
Units: mM
Initial value: $CCO_{tot} - a_{3,r,n}$
Normal concentration of oxidised cytochrome a_3.

$blood_{hb}$
Implementation Name: `blood_hb`
Units: dimensionless
Initial value: 10.00
Factor to convert model haemoglobin concentration to instrumental units. Scales for blood fraction of brain volume, mM to μM, and number of binding sites.

$a_{3,r,n}$
Implementation Name: `bred_n`
Units: mM
Initial value: $\frac{\frac{\frac{f_n}{k_3}}{O_{2,n}}}{\frac{\exp(-c_3\,(\Delta p_n - \Delta p_{30}))}{1+\exp(-c_3\,(\Delta p_n - \Delta p_{30}))}}$
Normal concentration of reduced cytochrome a3.

c_3
Implementation Name: `c3`
Units: mV^{-1}
Initial value: 0.11
Parameter controlling the sensitivity of the reduction of a_3 to Δp.

C_{buffi}
Implementation Name: `C_buffi`
Units: dimensionless
Initial value: 0.022
Buffering capacity of protons in mitochondria.

C_{im}
Implementation Name: `C_im`
Units: mM mV^{-1}
Initial value: 0.00675
Capacitance of the mitochondrial inner membrane.

$C_{NADH} = \frac{Z}{2} \log 10 \left(\frac{1}{\frac{NAD}{NADH}} \right)$
Implementation Name: `C_NADH`
Units: mV
Initial value: 0
Excess redox potential for NADH at normal demand.

$C_{NADH,n}$
Implementation Name: `C_NADH_n`
Units: mV
Initial value: $\frac{Z}{2} \log 10 \left(\frac{1}{\frac{NAD_n}{NADH_n}} \right)$
Normal value of C_{NADH}.

CBF_n
Implementation Name: `CBFn`
Units: $ml_{blood}\ ml_{brain}^{-1}\ s^{-1}$
Initial value: 0.0125
Normal cerebral blood flow.

$CBFscale$
Implementation Name: `CBFscale`
Units: cm
Initial value: 5000
Scale constant relating blood flow to arterial velocity.

$\Delta oxCCO_{off}$
Implementation Name: `CCO_offset`
Units: uM
Initial value: 0
Signal offset for the NIRS CCO measurement.

c_{k_1}
Implementation Name: `ck1`
Units: mV^{-1}
Initial value: 0.01
Parameter controlling sensitivity of k_1 to Δp.

c_{k_2}
Implementation Name: `ck2`
Units: mV^{-1}

Initial value: 0.02
Parameter controlling sensitivity of k_2 to Δp.

$CMRO_{2,n}$
Implementation Name: `CMRO2_n`
Units: mM s^{-1}
Initial value: 0.034
Normal metabolic rate of oxygen consumption.

CV_{inh}
Implementation Name: `CVinh`
Units: dimensionless
Initial value: 1
Control parameter representing the action of Complex V inhibitors.

$CCO_{tot} = \frac{CCO_{tis}}{Vol_{mit}}$
Implementation Name: `cytox_tot`
Units: mM
Initial value: 0
Concentration of cytochrome c oxidase in mitochondria.

CCO_{tis}
Implementation Name: `cytox_tot_tis`
Units: mM
Initial value: 0.0055
Concentration of cytochrome c oxidase in tissue.

D_{NADH}
Implementation Name: `D_NADH`
Units: dimensionless
Initial value: 0.01
Scale parameter for the dependence of NADH redox potential on demand.

D_{O_2}
Implementation Name: `D_O2`
Units: s^{-1}
Initial value: $\frac{J_{O_{2,n}}}{O_{2,c,n} - O_{2,n}}$
Diffusion rate for oxygen between capillaries and mitochondria.

$\Delta p_{3,corr}$
Implementation Name: `Dp3_corr`
Units: mV
Initial value: -25
Difference between Δp_{30} and normal Δp.

$\Delta p_{30} = \Delta p_n + \Delta p_{3,corr}$
Implementation Name: `Dp_30`
Units: mV
Initial value: 0
Value of Δp to which a_3 reduction reaction is maximally sensitive.

$\Delta p_{CV,0}$
Implementation Name: `Dp_CV0`
Units: mV
Initial value: 90
Value of Δp at which L_{CV} is zero under normal demand.

Δp_n
Implementation Name: `Dp_n`

Units: mV
Initial value: $\psi_n + Z\,\Delta pH_n$
Normal value of Δp

dpH
Implementation Name: `dpH`
Units: dimensionless
Initial value: 0.001
Parameter in the mitochondrial proton buffering relationship.

ΔpH_n
Implementation Name: `DpH_n`
Units: dimensionless
Initial value: $pH_{m,n} - pH_{o,n}$
Normal pH difference across the mitochondrial inner membrane.

ψ_n
Implementation Name: `Dpsi_n`
Units: mV
Initial value: 145
Normal mitochondrial inner membrane potential.

$E_{1,NADH} = \mathcal{E}_0(\mathrm{Cu}_A) - \mathcal{E}_0(\mathrm{NADH}) + C_{NADH}$
Implementation Name: `E1NADH`
Units: mV
Initial value: 0
Value of E_1 when the reducing substrate is NADH.

$E_{1,NADH,n}$
Implementation Name: `E1NADH_n`
Units: mV
Initial value: $\mathcal{E}_0(\mathrm{Cu}_A) - \mathcal{E}_0(\mathrm{NADH}) + C_{NADH,n}$
Normal value of $E_{1,NADH}$.

E_1
Implementation Name: `E_1`
Units: mV
Initial value: $E_{1,NADH}$
The energy provided by electron transfer to $\mathrm{Cu}_{A,r}$.

$E_{1,n}$
Implementation Name: `E_1n`
Units: mV
Initial value: $E_{1,NADH,n}$
Normal value of E_1.

E_2
Implementation Name: `E_2`
Units: mV
Initial value: $\mathcal{E}_0(\mathrm{a}_3) - \mathcal{E}_0(\mathrm{Cu}_A)$
Energy provided by the transfer of four electrons from $\mathrm{Cu}_{A,r}$ to $\mathrm{a}_{3,o}$.

$\mathcal{E}_0(\mathrm{a}_3)$
Implementation Name: `E_a30`
Units: mV
Initial value: 350
Standard redox potential for cytochrome a_3.

$\mathcal{E}_0(\mathrm{Cu}_A)$
Implementation Name: `E_c0`
Units: mV

Initial value: 247
Standard redox potential for Cu_A.

$\mathcal{E}_0$(NADH)
Implementation Name: `E_N0`
Units: mV
Initial value: -320
Standard redox potential for NADH.

f_n
Implementation Name: `f_n`
Units: mM s^{-1}
Initial value: $\frac{CMRO_{2,n}}{Vol_{mit}}$
Normal resting value of f_1 and f_2.

G_n
Implementation Name: `Gn`
Units: $ml_{blood}\ ml_{brain}^{-1}$ $mmHg^{-1}\ s^{-1}$
Initial value: $\frac{CBF_n}{P_{a,n} - P_{v,n}}$
Normal blood vessel conductance.

h_0
Implementation Name: `h_0`
Units: cm
Initial value: 0.003
Thickness of the blood vessel walls at which radius is r_0.

H_n^+
Implementation Name: `H_n`
Units: mM
Initial value: $10^{3-pH_{m,n}}$
Normal mitochondrial proton concentration.

h_n
Implementation Name: `h_n`
Units: cm
Initial value: $\mathrm{sqrt}\left(r_n\, r_n + 2r_0\, h_0 + h_0\, h_0\right) - r_n$
Normal thickness of the blood vessel walls.

$J_{O_{2,n}}$
Implementation Name: `J_O2n`
Units: mM s^{-1}
Initial value: $CMRO_{2,n}$
Normal oxygen flux from blood to tissue.

$k_{1,0}$
Implementation Name: `k10`
Units: s^{-1}
Initial value: $\frac{k_{1,n}\, NADH}{NADH_n}$
Forward reaction rate for the reduction of Cu_A at normal Δp.

$k_{1,n}$
Implementation Name: `k1_n`
Units: s^{-1}
Initial value: $\frac{f_n}{Cu_{A,o,n} - \frac{1}{K_{eq_1,n}}\, Cu_{A,r,n}}$
Forward reaction rate for the reduction of Cu_A at normal Δp and NADH.

$k_{2,n}$

Implementation Name: k2_n
Units: s^{-1}
Initial value: $\frac{f_n}{Cu_{A,r,n}\, a_{3,o,n} - \frac{1}{K_{eq_2,n}}\, Cu_{A,o,n}\, a_{3,r,n}}$
Normal forward reaction rate for the reduction of a_3.

$k_3 = \frac{k_{3,0}}{\frac{\exp(-c_3 - \Delta p_{30})}{1+\exp(-c_3 - \Delta p_{30})}}$

Implementation Name: k3
Units: s^{-1}
Initial value: 0
Forward reaction rate for the reduction of O_2.

$k_{3,0}$

Implementation Name: k30
Units: s^{-1}
Initial value: $2.5E+5$
Apparent second order rate constant for reduction of O_2 at zero Δp.

k_{aut}

Implementation Name: k_aut
Units: dimensionless
Initial value: 1
Overall functioning of autoregulatory response.

K_G

Implementation Name: K_G
Units: $ml_{blood}\ ml_{brain}^{-1}\ mmHg^{-1}\ s^{-1}\ cm^{-4}$
Initial value: $\frac{G_n}{\text{pow}\,(r_n, 4)}$
Proportionality constant in Poiseuille relation for conductance.

k_{lk2}

Implementation Name: k_lk2
Units: mV^{-1}
Initial value: 0.038
Constant controlling the depending of the leak rate L_{lk} on Δp.

K_σ

Implementation Name: K_sigma
Units: dimensionless
Initial value: 10
Parameter controlling the sensitivity of σ_e to vessel radius.

k_{unc}

Implementation Name: k_unc
Units: dimensionless
Initial value: 1
Control parameter simulating the effect of adding uncouplers to the system.

$k_{CV} = \frac{-1}{\Delta p_n - \Delta p_{CV,0}} \log\left(\frac{1 - L_{CV,0}}{1 + r_{CV}\, L_{CV,0}}\right)$

Implementation Name: kCV
Units: mV^{-1}
Initial value: 0
Parameter controlling the sensitivity of Complex V flux to driving force.

$K_{eq_1,n}$

Implementation Name: Keq1_n

Units: dimensionless
Initial value: $10^{\frac{-1}{Z}\left(\frac{p_1 \Delta p_n}{4} - E_{1,n}\right)}$
Normal value of the equilibrium constant for the Cu_A reduction reaction.

$K_{eq_2,n}$
Implementation Name: `Keq2_n`
Units: dimensionless
Initial value: $10^{\frac{-1}{Z}\left(\frac{p_3 \Delta p_n}{4} - E_2\right)}$
Normal value of the equilibrium constant for the a_3 reduction reaction.

$L_{CV,0}$
Implementation Name: `L_CV0`
Units: dimensionless
Initial value: 0.4
Normal Complex V flux as a fraction of maximum possible flux.

$L_{CV,frac} = 1 - L_{lk,frac}$
Implementation Name: `L_CVfrac`
Units: dimensionless
Initial value: 0
Normal fraction of proton entry into mitochondria which is due to ADP phosphorylation.

$L_{CV,max} = \dfrac{L_{CV,n}}{L_{CV,0}}$
Implementation Name: `L_CVmax`
Units: mM s^{-1}
Initial value: 0
The maximum rate of proton flow through Complex V.

$L_{CV,n}$
Implementation Name: `L_CVn`
Units: mM s^{-1}
Initial value: $L_n \, L_{CV,frac}$
The resting flow of protons into the matrix through Complex V.

L_{lk0}
Implementation Name: `L_lk0`
Units: mM s^{-1}
Initial value: $\dfrac{L_{lk,n}}{\exp\left(\Delta p_n \, k_{lk2}\right) - 1}$
Constant controlling the depending of the leak rate L_{lk} on Δp.

$L_{lk,frac}$
Implementation Name: `L_lkfrac`
Units: dimensionless
Initial value: 0.25
Normal fraction of proton entry into mitochondria which is via leak channels.

$L_{lk,n}$
Implementation Name: `L_lkn`
Units: mM s^{-1}
Initial value: $L_n \, L_{lk,frac}$
The resting flow of protons into the matrix via leak channels.

$L_n = p_{tot} \, f_n$
Implementation Name: `L_n`
Units: mM s^{-1}
Initial value: 0
The normal total flow of protons back into mitochondria.

μ_{max}
Implementation Name: `mu_max`
Units: dimensionless
Initial value: 1
Upper bound for the transformed stimulus μ.

μ_{min}
Implementation Name: `mu_min`
Units: dimensionless
Initial value: -1
Lower bound for the transformed stimulus μ.

μ_n
Implementation Name: `mu_n`
Units: dimensionless
Initial value: 0
Normal value for the transformed stimulus μ.

n_h
Implementation Name: `n_h`
Units: dimensionless
Initial value: 2.5
Hill coefficient for oxygen dissociation from haemoglobin.

n_m
Implementation Name: `n_m`
Units: dimensionless
Initial value: 1.83
Exponent in the muscular tension relationship.

$NADH = \dfrac{NAD_{pool}}{1 + \frac{NAD}{NADH}}$
Implementation Name: `NADH`
Units: mM
Initial value: 0
Concentration of NADH in the mitochondria.

$NADH_n$
Implementation Name: `NADHn`
Units: mM
Initial value: $\dfrac{NAD_{pool}}{1 + \frac{NAD_n}{NADH_n}}$
Normal concentration of NADH in the mitochondria.

$\frac{NAD}{NADH}$
Implementation Name: `NADNADHrat`
Units: dimensionless
Initial value: $\dfrac{\frac{NAD_n}{NADH_n}}{\text{pow}\,(u, 2D_{NADH})}$
NAD/NADH ratio.

$\frac{NAD_n}{NADH_n}$
Implementation Name: `NADNADHratn`
Units: dimensionless
Initial value: 9
Normal NAD/NADH ratio.

NAD_{pool}
Implementation Name: `NADpool`
Units: dimensionless

Initial value: 3
Relative size of the NAD pool, used to estimate normal mitochondrial NADH.

$O_{2,n}$
Implementation Name: O2_n
Units: mM
Initial value: 0.024
Normal mitochondrial oxygen concentration.

$O_{2,c,n}$
Implementation Name: O2c_n
Units: mM
Initial value: $\phi \,\mathrm{pow}\left(\frac{S_{c,O_2,n}}{1 - S_{c,O_2,n}}, \frac{1}{n_h}\right)$
Normal capillary oxygen concentration.

$p_1 = p_{tot} - p_{23}$
Implementation Name: p1
Units: dimensionless
Initial value: 0
Proton cost of the reaction reducing Cu_A.

p_3
Implementation Name: p2
Units: dimensionless
Initial value: 4
Proton cost of the reaction reducing a_3.

p_{23}
Implementation Name: p23
Units: dimensionless
Initial value: 8
Total protons removed from the mitochondrial matrix by the reductions of a_3 and O_2.

p_3
Implementation Name: p3
Units: dimensionless
Initial value: $p_{23} - p_3$
Proton cost of the reaction reducing O_2.

$P_1 = \frac{P_a + P_v}{2}$
Implementation Name: P_1
Units: mm Hg
Initial value: $P_{1,n}$
Average pressure in the blood vessels.

$P_{1,n}$
Implementation Name: P_1n
Units: mm Hg
Initial value: $\frac{P_{a,n} + P_{v,n}}{2}$
Normal value for the average pressure in the blood vessels.

P_a
Implementation Name: P_a
Units: mmHg
Initial value: $P_{a,n}$
Mean arterial blood pressure.

$P_{a,n}$
Implementation Name: P_an

Units: mmHg
Initial value: 100
Normal arterial blood pressure.

p_{C1}
Implementation Name: p_C1
Units: dimensionless
Initial value: 8
Protons pumped by Complex I.

p_{C3}
Implementation Name: p_C3
Units: dimensionless
Initial value: 4
Protons pumped by Complex III.

P_{ic}
Implementation Name: P_ic
Units: mm Hg
Initial value: 9.5
Intracranial pressure.

P_{icn}
Implementation Name: P_icn
Units: mm Hg
Initial value: 9.5
Normal intracranial pressure.

p_{tot}
Implementation Name: p_tot
Units: dimensionless
Initial value: $p_{tot,NADH}$
Total protons removed from the mitochondrial matrix by the three modelled electron transport reactions.

$p_{tot,NADH} = p_{C1} + p_{C3} + p_{23}$
Implementation Name: p_totNADH
Units: dimensionless
Initial value: 0
Total protons pumped when the reducing agent is NADH.

P_v
Implementation Name: P_v
Units: mmHg
Initial value: $P_{v,n}$
Venous blood pressure.

$P_{v,n}$
Implementation Name: P_vn
Units: mmHg
Initial value: 4
Normal venous blood pressure.

Pa_{CO_2}
Implementation Name: Pa_CO2
Units: mmHg
Initial value: $Pa_{CO_{2,n}}$
Arterial partial pressure of carbon dioxide.

$Pa_{CO_{2,n}}$
Implementation Name: Pa_CO2n

Units: mmHg
Initial value: 40
Normal arterial partial pressure of carbon dioxide.

$pH_{m,n}$
Implementation Name: `pH_mn`
Units: dimensionless
Initial value: 7.4
Normal mitochondrial pH.

pH_o
Implementation Name: `pH_o`
Units: dimensionless
Initial value: 7
Extra-mitochondrial pH.

$pH_{o,n}$
Implementation Name: `pH_on`
Units: dimensionless
Initial value: 7
Normal extra-mitochondrial pH.

ϕ
Implementation Name: `phi`
Units: mM
Initial value: 0.036
Oxygen concentration at half-maximal saturation.

r_0
Implementation Name: `r_0`
Units: cm
Initial value: 0.0126
Radius in the elastic tension relationship.

R_{CO_2}
Implementation Name: `R_autc`
Units: dimensionless
Initial value: 2.2
Autoregulatory reactivity to carbon dioxide.

R_{O_2}
Implementation Name: `R_auto`
Units: dimensionless
Initial value: 1.5
Autoregulatory reactivity to oxygen.

R_{P_a}
Implementation Name: `R_autp`
Units: dimensionless
Initial value: 4
Autoregulatory reactivity to blood pressure.

R_u
Implementation Name: `R_autu`
Units: dimensionless
Initial value: 0.5
Autoregulatory reactivity to demand.

r_{CV}
Implementation Name: `r_CV`
Units: dimensionless

Initial value: 5
Parameter controlling the ratio of maximal to minimal rates of oxidative phosphorylation.

r_m
Implementation Name: `r_m`
Units: cm
Initial value: 0.027
Vessel radius at which muscular tension is maximal.

r_n
Implementation Name: `r_n`
Units: cm
Initial value: 0.0187
Normal blood vessel radius. Normal effective blood vessel radius.

r_t
Implementation Name: `r_t`
Units: cm
Initial value: 0.018
Radius in the muscular tension relationship.

$S_{a,O_2,n}$
Implementation Name: `SaO2_n`
Units: dimensionless
Initial value: 0.96
Normal arterial oxygen saturation.

S_{a,O_2}
Implementation Name: `SaO2sup`
Units: dimensionless
Initial value: $S_{a,O_2,n}$
Arterial oxygen saturation.

$S_{c,O_2,n}$
Implementation Name: `ScO2_n`
Units: dimensionless
Initial value: $\frac{S_{a,O_2,n} + S_{v,O_2,n}}{2}$
Normal capillary oxygen saturation.

σ_{coll}
Implementation Name: `sigma_coll`
Units: mm Hg
Initial value: 62.79
Pressure at which blood vessels collapse.

$\sigma_{e,0}$
Implementation Name: `sigma_e0`
Units: mm Hg
Initial value: 0.1425
Parameter in the elastic tension relationship.

$\sigma_{e,n}$
Implementation Name: `sigma_en`
Units: mm Hg
Initial value: $\sigma_{e,0}\left(\exp\left(\frac{K_\sigma\ (r_n - r_0)}{r_0}\right) - 1\right) - \sigma_{coll}$
Normal elastic stress in blood vessel walls.

$S_{v,O_2,n}$
Implementation Name: `SvO2_n`

Units: dimensionless
Initial value: $\frac{HbO_{2,v,n}}{Hb_{tot,n}}$
Normal venous oxygen saturation.

t

Implementation Name: t
Units: s
Initial value: 0
Time over which the system evolves.

τ_{CO_2}

Implementation Name: t_c
Units: s
Initial value: 5
Filter time constant for stimulus effect of carbon dioxide.

$T_{e,n}$

Implementation Name: T_en
Units: mm Hg cm
Initial value: $\sigma_{e,n}\, h_n$
Normal elastic tension in the blood vessel walls.

$T_{max,0}$

Implementation Name: T_max0
Units: mm Hg cm
Initial value: $\frac{T_{max,n}}{1 + k_{aut}\, \mu_n}$
Maximal muscular tension under normal regulatory stimulus ($\mu = \mu_n$).

$T_{max,n}$

Implementation Name: T_maxn
Units: mm Hg cm
Initial value: $\frac{T_{m,n}}{\exp\left(-\text{pow}\left(\text{fabs}\left(\frac{r_n - r_m}{r_t - r_m}\right), n_m\right)\right)}$
Normal maximal muscular tension.

$T_{m,n}$

Implementation Name: T_mn
Units: mm Hg cm
Initial value: $(P_{1,n} - P_{icn})\; r_n - T_{e,n}$
Normal muscular tension in the blood vessel walls.

τ_{O_2}

Implementation Name: t_o
Units: s
Initial value: 20
Filter time constant for stimulus effect of capillary oxygen.

τ_{P_a}

Implementation Name: t_p
Units: s
Initial value: 5
Filter time constant for stimulus effect of blood pressure.

τ_u

Implementation Name: t_u
Units: s
Initial value: 0.5
Filter time constant for stimulus effect of demand.

u
Implementation Name: `u`
Units: dimensionless
Initial value: u_n
Parameter indicating metabolic demand.

u_n
Implementation Name: `u_n`
Units: dimensionless
Initial value: 1
Normal demand.

$v_{CO_2,n}$
Implementation Name: `v_cn`
Units: mmHg
Initial value: $Pa_{CO_{2,n}}$
Normal filtered carbon dioxide partial pressure. Normal filtered carbon dioxide partial pressure.

$v_{O_2,n}$
Implementation Name: `v_on`
Units: mM
Initial value: $O_{2,c,n}$
Normal filtered capillary oxygen concentration. Normal filtered capillary oxygen concentration.

$v_{P_a,n}$
Implementation Name: `v_pn`
Units: mmHg
Initial value: $P_{a,n}$
Normal filtered arterial blood pressure. Normal filtered blood pressure.

$v_{u,n}$
Implementation Name: `v_un`
Units: dimensionless
Initial value: u_n
Normal filtered demand. Normal filtered demand.

$VArat_n$
Implementation Name: `VArat_n`
Units: dimensionless
Initial value: 3
Normal volume ratio of veins to arteries in brain tissue.

$V_{a,n}$
Implementation Name: `Vol_artn`
Units: dimensionless
Initial value: $\frac{1}{1 + VArat_n}$
Normal relative arterial blood volume.

Vol_{mit}
Implementation Name: `Vol_mit`
Units: dimensionless
Initial value: 0.067
Fraction of brain tissue volume that is mitochondria.

V_v
Implementation Name: `Vol_ven`
Units: dimensionless

Initial value: $\dfrac{VArat_n}{1 + VArat_n}$
Relative venous blood volume.

$HbO_{2,a} \; = Hb_{tot} \, S_{a,O_2}$
Implementation Name: `XOa`
Units: mM
Initial value: $HbO_{2,a,n}$
Arterial concentration of oxygen bound to haemoglobin.

$HbO_{2,a,n}$
Implementation Name: `XOa_n`
Units: mM
Initial value: $Hb_{tot,n} \, S_{a,O_2,n}$
Normal arterial concentration of oxygen bound to haemoglobin.

$HbO_{2,v,n}$
Implementation Name: `XOv_n`
Units: mM
Initial value: $\dfrac{CBF_n \, HbO_{2,a,n} - J_{O_{2,n}}}{CBF_n}$
Normal venous concentration of oxygen bound to haemoglobin.

Hb_{tot}
Implementation Name: `Xtot`
Units: mM
Initial value: 9.1
Total concentration of haemoglobin O_2 binding sites in blood (4 times haemoglobin concentration).

$Hb_{tot,n}$
Implementation Name: `Xtot_n`
Units: mM
Initial value: 9.1
Normal total concentration of haemoglobin O_2 binding sites in blood (4 times haemoglobin concentration).

Z
Implementation Name: `Z`
Units: mV
Initial value: 59.028
Proportionality constant in calculation of driving forces due to concentration differences. Defined as RT/F, where F is Faraday's constant, R the ideal gas constant and T the absolute temperature.

2 BSB1

2.1 Overview

Simplified model in which the blood flow submodel is replaced with variant B1.

- 9 differential state variables
- 3 algebraic state variables
- 35 intermediate variables
- 122 parameters
- 4 declared inputs
- 33 default outputs

2.2 Differential Equations

$$\frac{\mathrm{d}Cu_{A,o}}{\mathrm{d}t} = 4f_3 - 4f_1 \tag{2.1}$$

$$\frac{\mathrm{d}a_{3,r}}{\mathrm{d}t} = 4f_3 - 4f_3 \tag{2.2}$$

$$\frac{\mathrm{d}\psi}{\mathrm{d}t} = \frac{p_3\, f_3 + p_1\, f_1 + p_3\, f_3 - L}{C_{im}} \tag{2.3}$$

$$\frac{\mathrm{d}H^+}{\mathrm{d}t} = \frac{1}{R_{Hi}}\, L - \frac{p_3}{R_{Hi}}\, f_3 - \frac{p_1}{R_{Hi}}\, f_1 - \frac{p_3}{R_{Hi}}\, f_3 \tag{2.4}$$

$$\frac{\mathrm{d}O_2}{\mathrm{d}t} = \frac{1}{Vol_{mit}}\, J_{O_2} - f_3 \tag{2.5}$$

$$\frac{\mathrm{d}v_{CO_2}}{\mathrm{d}t} = \frac{1}{\tau_{CO_2}}\, \left(Pa_{CO_2} - v_{CO_2}\right) \tag{2.6}$$

$$\frac{\mathrm{d}v_{O_2}}{\mathrm{d}t} = \frac{1}{\tau_{O_2}}\, \left(O_{2,c} - v_{O_2}\right) \tag{2.7}$$

$$\frac{\mathrm{d}v_{P_a}}{\mathrm{d}t} = \frac{1}{\tau_{P_a}}\, \left(P_a - v_{P_a}\right) \tag{2.8}$$

$$\frac{\mathrm{d}\nu_u}{\mathrm{d}t} = \frac{1}{\tau_u}\,(u - \nu_u) \tag{2.9}$$

2.3 Algebraic Equations

$$\phi\left(\frac{S_{c,O_2}}{1 - S_{c,O_2}}\right)^{\frac{1}{n_h}} - O_{2,c} = 0 \tag{2.10}$$

$$\lambda_0 + \frac{\lambda_{P_a}}{P_a} + \lambda_\mu\,\mu + \frac{\lambda_{P_a,\mu}\,\mu}{P_a} - r = 0 \tag{2.11}$$

$$CBF\,(HbO_{2,a} - HbO_{2,v}) - J_{O_2} = 0 \tag{2.12}$$

2.4 Chemical Reactions

$$\xrightarrow{L} \frac{1}{R_{Hi}}\,\mathrm{H}^+ \tag{2.13}$$

$$\xrightarrow{J_{O_2}} \frac{1}{Vol_{mit}}\,\mathrm{O}_2 \tag{2.14}$$

$$\frac{p_3}{R_{Hi}}\,\mathrm{H}^+ \xrightarrow{f_3} 4\,\mathrm{Cu}_{\mathrm{A,o}} + 4\,\mathrm{a}_{3,\mathrm{r}} \tag{2.15}$$

$$4\,\mathrm{Cu}_{\mathrm{A,o}} + \frac{p_1}{R_{Hi}}\,\mathrm{H}^+ \xrightarrow{f_1} \tag{2.16}$$

$$\mathrm{O}_2 + 4\,\mathrm{a}_{3,\mathrm{r}} + \frac{p_3}{R_{Hi}}\,\mathrm{H}^+ \xrightarrow{f_3} \tag{2.17}$$

2.5 State Variables

$Cu_{A,o}$
Implementation Name: `a`
Units: mM
Initial value: $Cu_{A,o,n}$
Concentration of oxidised cytochrome c oxidase.

$a_{3,r}$
Implementation Name: `bred`
Units: mM
Initial value: $a_{3,r,n}$
Concentration of reduced cytochrome a_3.

ψ
Implementation Name: `Dpsi`
Units: mV
Initial value: ψ_n
Mitochondrial inner membrane potential. Varies as charge (in the form of protons) is transferred across the membrane capacitance.

H^+
Implementation Name: `H`
Units: mM
Initial value: H_n^+
Mitochondrial proton concentration.

O_2
Implementation Name: `O2`
Units: mM
Initial value: $O_{2,n}$
Mitochondrial oxygen concentration.

$O_{2,c}$
Implementation Name: `O2c`
Units: mM
Initial value: $O_{2,c,n}$
Capillary oxygen concentration.

r
Implementation Name: `r`
Units: cm
Initial value: r_n
Typical blood vessel radius.

ν_{CO_2}
Implementation Name: `v_c`
Units: mmHg
Initial value: $\nu_{CO_2,n}$
Filtered carbon dioxide partial pressure.

ν_{O_2}
Implementation Name: `v_o`
Units: mM
Initial value: $\nu_{O_2,n}$
Filtered capillary oxygen concentration.

ν_{P_a}
Implementation Name: `v_p`
Units: mmHg
Initial value: $\nu_{P_a,n}$
Filtered arterial blood pressure.

ν_u
Implementation Name: `v_u`
Units: dimensionless
Initial value: $\nu_{u,n}$
Filtered demand.

$HbO_{2,v}$
Implementation Name: `XOv`
Units: mM
Initial value: $HbO_{2,v,n}$
Venous concentration of oxygen bound to haemoglobin.

2.6 Intermediate Variables

$Cu_{A,r} = CCO_{tot} - Cu_{A,o}$
Implementation Name: `ared`
Units: mM
Initial value: 0
Concentration of reduced Cu_A.

$a_{3,o} = CCO_{tot} - a_{3,r}$
Implementation Name: `b`
Units: mM
Initial value: 0
Concentration of oxidised cytochrome a_3.

$C_{0,i} = \dfrac{10^{-pH_m} - 10^{-pH_m - dpH}}{dpH}$
Implementation Name: `C_0i`
Units: dimensionless
Initial value: 0
Natural buffering capacity of protons in mitochondria.

$CBF = G\ (P_a - P_v)$
Implementation Name: `CBF`
Units: $ml_{blood}\ ml_{brain}^{-1}\ s^{-1}$
Initial value: CBF_n
Cerebral blood flow.

$\Delta oxCCO = \Delta oxCCO_{off} + 1000 Vol_{mit}\ (Cu_{A,o} - Cu_{A,o,n})$
Implementation Name: `CCO`
Units: uM
Initial value: 0
Cytochrome c oxidase signal measured by NIRS.

$CMRO_2 = f_3\, Vol_{mit}$
Implementation Name: `CMRO2`
Units: mM s^{-1}
Initial value: 0
Rate of cerebral oxygen metabolism.

$\Delta p = \psi + Z\ (pH_m - pH_o)$
Implementation Name: `Dp`
Units: mV
Initial value: 0
Proton motive force across the mitochondrial inner membrane.

$\eta = R_{P_a} \left(\dfrac{v_{P_a}}{v_{P_a,n}} - 1 \right) + R_{O_2} \left(\dfrac{v_{O_2}}{v_{O_2,n}} - 1 \right) + R_{CO_2} \left(1 - \dfrac{v_{CO_2}}{v_{CO_2,n}} \right) + R_u \left(1 - \dfrac{v_u}{v_{u,n}} \right)$
Implementation Name: `eta`
Units: dimensionless
Initial value: 0
Merged autoregulation stimulus.

$f_1 = k_1\, Cu_{A,o} - k_{-1}\, Cu_{A,r}$
Implementation Name: `f1`
Units: mM s^{-1}
Initial value: 0
Reaction rate for the reduction of Cu_A.

$f_3 = k_2\, Cu_{A,r}\, a_{3,o} - k_{-2}\, Cu_{A,o}\, a_{3,r}$
Implementation Name: `f2`

Units: mM s^{-1}
Initial value: 0
Reaction rate for the reduction of a_3.

$f_3 = \frac{k_3\, O_2\, a_{3,r} \exp\left(-c_3\, (\Delta p - \Delta p_{30})\right)}{1 + \exp\left(-c_3\, (\Delta p - \Delta p_{30})\right)}$
Implementation Name: `f3`
Units: mM s^{-1}
Initial value: 0
Reaction rate for the reduction of O_2.

$G = K_G\, r^4$
Implementation Name: `G`
Units: $ml_{blood}\; ml_{brain}^{-1}\; mmHg^{-1}\; s^{-1}$
Initial value: 0
Effective conductance of the whole blood flow compartment.

$HbO_2 = (V_a\, HbO_{2,a} + V_v\, HbO_{2,v})\; blood_{hb}$
Implementation Name: `HbO2`
Units: uM
Initial value: 0
Oxygenated haemoglobin signal measured by NIRS.

$HbT = (V_a + V_v)\; Hb_{tot}\, blood_{hb}$
Implementation Name: `HbT`
Units: uM
Initial value: 0
Total haemoglobin signal measured by NIRS.

$HHb = HbT - HbO_2$
Implementation Name: `HHb`
Units: uM
Initial value: 0
Deoxygenated haemoglobin signal measured by NIRS.

$J_{O_2} = \text{fmin}\left(D_{O_2}\, (O_{2,c} - O_2)\, , CBF\, HbO_{2,a}\right)$
Implementation Name: `J_O2`
Units: mM s^{-1}
Initial value: 0
Oxygen flux from blood to tissue.

$k_1 = k_{1,0} \exp\left(-c_{k_1}\, (\Delta p - \Delta p_n)\right)$
Implementation Name: `k1`
Units: s^{-1}
Initial value: 0
Forward reaction rate for the reduction of Cu_A.

$k_2 = k_{2,n} \exp\left(-c_{k_2}\, (\Delta p - \Delta p_n)\right)$
Implementation Name: `k2`
Units: s^{-1}
Initial value: 0
Forward reaction rate for the reduction of a_3.

$K_{eq_1} = 10^{\frac{-1}{Z}\left(\frac{p_1\, \Delta p}{4} - E_1\right)}$
Implementation Name: `Keq1`
Units: dimensionless
Initial value: 0
Equilibrium constant for the Cu_A reduction reaction.

$K_{eq_2} = 10^{\frac{-1}{Z}\left(\frac{p_3\, \Delta p}{4} - E_2\right)}$

Implementation Name: `Keq2`
Units: dimensionless
Initial value: 0
Equilibrium constant for the a_3 reduction reaction.

$k_{-1} = \frac{k_1}{K_{eq_1}}$
Implementation Name: `kn1`
Units: s^{-1}
Initial value: 0
Reverse reaction rate for the reduction of Cu_A.

$k_{-2} = \frac{k_2}{K_{eq_2}}$
Implementation Name: `kn2`
Units: s^{-1}
Initial value: 0
Reverse reaction rate for the reduction of a_3.

$L = L_{CV} + L_{lk}$
Implementation Name: `L`
Units: mM s^{-1}
Initial value: 0
Rate of proton return to the mitochondrial matrix.

$L_{CV} = \frac{CV_{inh}\, L_{CV,max}\, (1 - \exp(-\theta))}{1 + r_{CV} \exp(-\theta)}$
Implementation Name: `L_CV`
Units: mM s^{-1}
Initial value: 0
Rate at which protons re-enter the mitochondrial matrix due to ADP phosphorylation.

$L_{lk} = k_{unc}\, L_{lk0}\, (\exp(\Delta p\, k_{lk2}) - 1)$
Implementation Name: `L_lk`
Units: mM s^{-1}
Initial value: 0
Rate at which protons re-enter the mitochondrial matrix via leak channels.

$\mu = \frac{k_{aut}\, (\exp(\eta) - 1)}{\exp(\eta) + 1}$
Implementation Name: `mu`
Units: dimensionless
Initial value: 0
Effective strength of the autoregulation reponse.

$pH_m = -\text{log10}\left(\frac{H^+}{1000}\right)$
Implementation Name: `pH_m`
Units: dimensionless
Initial value: 0
Mitochondrial pH.

$r_{buffi} = \frac{C_{buffi}}{C_{0,i}}$
Implementation Name: `r_buffi`
Units: dimensionless
Initial value: 0
Buffering capacity for protons in mitochondria.

$R_{Hi} = r_{buffi}$

Implementation Name: `R_Hi`
Units: dimensionless
Initial value: 0
Relative mitochondrial volume for protons, taking into account buffering effect of pH.

$S_{c,O_2} = \frac{S_{a,O_2} + S_{v,O_2}}{2}$
Implementation Name: `ScO2`
Units: dimensionless
Initial value: $S_{c,O_2,n}$
Capillary oxygen saturation.

$S_{v,O_2} = \frac{HbO_{2,v}}{Hb_{tot}}$
Implementation Name: `SvO2`
Units: dimensionless
Initial value: $S_{v,O_2,n}$
Venous oxygen saturation.

$\theta = k_{CV} \left(\Delta p + Z \log 10\left(u\right) - \Delta p_{CV,0}\right)$
Implementation Name: `theta`
Units: dimensionless
Initial value: 0
Driving force Complex V.

$TOI = \frac{100 HbO_2}{HbT}$
Implementation Name: `TOI`
Units: dimensionless
Initial value: 0
Total oxygenation index.

$V_{mca} = CBF\, CBFscale$
Implementation Name: `Vmca`
Units: cm s^{-1}
Initial value: 0
Blood velocity in the middle cerebral artery.

$V_a = V_{a,n} \left(\frac{r}{r_n}\right)^2$
Implementation Name: `Vol_art`
Units: dimensionless
Initial value: 0
Relative arterial blood volume.

2.7 Parameters

$Cu_{a,frac,n}$
Implementation Name: `a_frac_n`
Units: dimensionless
Initial value: 0.8
Normal oxidised fraction of Cu_A.

$Cu_{A,o,n}$
Implementation Name: `a_n`
Units: mM
Initial value: $CCO_{tot}\, Cu_{a,frac,n}$
Normal concentration of oxidised cytochrome c oxidase.

$Cu_{A,r,n}$
Implementation Name: `ared_n`
Units: mM
Initial value: $CCO_{tot} - Cu_{A,o,n}$
Normal concentration of reduced Cu_A.

$a_{3,o,n}$
Implementation Name: `b_n`
Units: mM
Initial value: $CCO_{tot} - a_{3,r,n}$
Normal concentration of oxidised cytochrome a_3.

$blood_{hb}$
Implementation Name: `blood_hb`
Units: dimensionless
Initial value: 10.00
Factor to convert model haemoglobin concentration to instrumental units. Scales for blood fraction of brain volume, mM to μM, and number of binding sites.

$a_{3,r,n}$
Implementation Name: `bred_n`
Units: mM
Initial value: $\frac{\frac{\frac{f_n}{k_3}}{O_{2,n}}}{\frac{\exp(-c_3\,(\Delta p_n - \Delta p_{30}))}{1+\exp(-c_3\,(\Delta p_n - \Delta p_{30}))}}$
Normal concentration of reduced cytochrome a3.

c_3
Implementation Name: `c3`
Units: mV^{-1}
Initial value: 0.11
Parameter controlling the sensitivity of the reduction of a_3 to Δp.

C_{buffi}
Implementation Name: `C_buffi`
Units: dimensionless
Initial value: 0.022
Buffering capacity of protons in mitochondria.

C_{im}
Implementation Name: `C_im`
Units: mM mV^{-1}
Initial value: 0.00675
Capacitance of the mitochondrial inner membrane.

$C_{NADH} = \frac{Z}{2}\log 10\left(\frac{1}{\frac{NAD}{NADH}}\right)$
Implementation Name: `C_NADH`
Units: mV
Initial value: 0
Excess redox potential for NADH at normal demand.

$C_{NADH,n}$
Implementation Name: `C_NADH_n`
Units: mV
Initial value: $\frac{Z}{2}\log 10\left(\frac{1}{\frac{NAD_n}{NADH_n}}\right)$
Normal value of C_{NADH}.

CBF_n
Implementation Name: `CBFn`
Units: $ml_{blood}\ ml_{brain}^{-1}\ s^{-1}$
Initial value: 0.0125
Normal cerebral blood flow.

$CBFscale$
Implementation Name: `CBFscale`
Units: cm
Initial value: 5000
Scale constant relating blood flow to arterial velocity.

$\Delta oxCCO_{off}$
Implementation Name: `CCO_offset`
Units: uM
Initial value: 0
Signal offset for the NIRS CCO measurement.

c_{k_1}
Implementation Name: `ck1`
Units: mV^{-1}
Initial value: 0.01
Parameter controlling sensitivity of k_1 to Δp.

c_{k_2}
Implementation Name: `ck2`
Units: mV^{-1}
Initial value: 0.02
Parameter controlling sensitivity of k_2 to Δp.

$CMRO_{2,n}$
Implementation Name: `CMRO2_n`
Units: $mM\ s^{-1}$
Initial value: 0.034
Normal metabolic rate of oxygen consumption.

CV_{inh}
Implementation Name: `CVinh`
Units: dimensionless
Initial value: 1
Control parameter representing the action of Complex V inhibitors.

$CCO_{tot} = \dfrac{CCO_{tis}}{Vol_{mit}}$
Implementation Name: `cytox_tot`
Units: mM
Initial value: 0
Concentration of cytochrome c oxidase in mitochondria.

CCO_{tis}
Implementation Name: `cytox_tot_tis`
Units: mM
Initial value: 0.0055
Concentration of cytochrome c oxidase in tissue.

D_{NADH}
Implementation Name: `D_NADH`
Units: dimensionless
Initial value: 0.01
Scale parameter for the dependence of NADH redox potential on demand.

D_{O_2}
Implementation Name: D_O2
Units: s^{-1}
Initial value: $\dfrac{J_{O_{2,n}}}{O_{2,c,n} - O_{2,n}}$
Diffusion rate for oxygen between capillaries and mitochondria.

$\Delta p_{3,corr}$
Implementation Name: Dp3_corr
Units: mV
Initial value: -25
Difference between Δp_{30} and normal Δp.

$\Delta p_{30} = \Delta p_n + \Delta p_{3,corr}$
Implementation Name: Dp_30
Units: mV
Initial value: 0
Value of Δp to which a_3 reduction reaction is maximally sensitive.

$\Delta p_{CV,0}$
Implementation Name: Dp_CV0
Units: mV
Initial value: 90
Value of Δp at which L_{CV} is zero under normal demand.

Δp_n
Implementation Name: Dp_n
Units: mV
Initial value: $\psi_n + Z\,\Delta pH_n$
Normal value of Δp

dpH
Implementation Name: dpH
Units: dimensionless
Initial value: 0.001
Parameter in the mitochondrial proton buffering relationship.

ΔpH_n
Implementation Name: DpH_n
Units: dimensionless
Initial value: $pH_{m,n} - pH_{o,n}$
Normal pH difference across the mitochondrial inner membrane.

ψ_n
Implementation Name: Dpsi_n
Units: mV
Initial value: 145
Normal mitochondrial inner membrane potential.

$E_{1,NADH} = \mathcal{E}_0(\mathrm{Cu}_A) - \mathcal{E}_0(\mathrm{NADH}) + C_{NADH}$
Implementation Name: E1NADH
Units: mV
Initial value: 0
Value of E_1 when the reducing substrate is NADH.

$E_{1,NADH,n}$
Implementation Name: E1NADH_n
Units: mV
Initial value: $\mathcal{E}_0(\mathrm{Cu}_A) - \mathcal{E}_0(\mathrm{NADH}) + C_{NADH,n}$
Normal value of $E_{1,NADH}$.

E_1
Implementation Name: E_1
Units: mV
Initial value: $E_{1,NADH}$
The energy provided by electron transfer to $\text{Cu}_{A,r}$.

$E_{1,n}$
Implementation Name: E_1n
Units: mV
Initial value: $E_{1,NADH,n}$
Normal value of E_1.

E_2
Implementation Name: E_2
Units: mV
Initial value: $\mathcal{E}_0(\text{a}_3) - \mathcal{E}_0(\text{Cu}_A)$
Energy provided by the transfer of four electrons from $\text{Cu}_{A,r}$ to $\text{a}_{3,o}$.

$\mathcal{E}_0(\text{a}_3)$
Implementation Name: E_a30
Units: mV
Initial value: 350
Standard redox potential for cytochrome a_3.

$\mathcal{E}_0(\text{Cu}_A)$
Implementation Name: E_c0
Units: mV
Initial value: 247
Standard redox potential for Cu_A.

$\mathcal{E}_0(\text{NADH})$
Implementation Name: E_N0
Units: mV
Initial value: -320
Standard redox potential for NADH.

f_n
Implementation Name: f_n
Units: mM s^{-1}
Initial value: $\frac{CMRO_{2,n}}{Vol_{mit}}$
Normal resting value of f_1 and f_2.

G_n
Implementation Name: Gn
Units: $\text{ml}_{blood}\ \text{ml}_{brain}^{-1}\ \text{mmHg}^{-1}\ \text{s}^{-1}$
Initial value: $\frac{CBF_n}{P_{a,n} - P_{v,n}}$
Normal blood vessel conductance.

H_n^+
Implementation Name: H_n
Units: mM
Initial value: $10^{3-pH_{m,n}}$
Normal mitochondrial proton concentration.

$J_{O_{2,n}}$
Implementation Name: J_O2n
Units: mM s^{-1}
Initial value: $CMRO_{2,n}$
Normal oxygen flux from blood to tissue.

$k_{1,0}$

Implementation Name: `k10`
Units: s^{-1}
Initial value: $\frac{k_{1,n}\, NADH}{NADH_n}$
Forward reaction rate for the reduction of Cu_A at normal Δp.

$k_{1,n}$

Implementation Name: `k1_n`
Units: s^{-1}
Initial value: $\frac{f_n}{Cu_{A,o,n} - \frac{1}{K_{eq_1,n}}\, Cu_{A,r,n}}$
Forward reaction rate for the reduction of Cu_A at normal Δp and NADH.

$k_{2,n}$

Implementation Name: `k2_n`
Units: s^{-1}
Initial value: $\frac{f_n}{Cu_{A,r,n}\, a_{3,o,n} - \frac{1}{K_{eq_2,n}}\, Cu_{A,o,n}\, a_{3,r,n}}$
Normal forward reaction rate for the reduction of a_3.

$k_3 = \frac{k_{3,0}}{\frac{\exp(-c_3 - \Delta p_{30})}{1+\exp(-c_3 - \Delta p_{30})}}$

Implementation Name: `k3`
Units: s^{-1}
Initial value: 0
Forward reaction rate for the reduction of O_2.

$k_{3,0}$

Implementation Name: `k30`
Units: s^{-1}
Initial value: $2.5E+5$
Apparent second order rate constant for reduction of O_2 at zero Δp.

k_{aut}

Implementation Name: `k_aut`
Units: dimensionless
Initial value: 1
Overall functioning of autoregulatory response.

K_G

Implementation Name: `K_G`
Units: $ml_{blood}\ ml_{brain}^{-1}\ mmHg^{-1}\ s^{-1}\ cm^{-4}$
Initial value: $\frac{G_n}{\mathrm{pow}\,(r_n, 4)}$
Proportionality constant in Poiseuille relation for conductance.

k_{lk2}

Implementation Name: `k_lk2`
Units: mV^{-1}
Initial value: 0.038
Constant controlling the depending of the leak rate L_{lk} on Δp.

k_{unc}

Implementation Name: `k_unc`
Units: dimensionless
Initial value: 1
Control parameter simulating the effect of adding uncouplers to the system.

$k_{CV} = \dfrac{-1}{\Delta p_n - \Delta p_{CV,0}} \log\left(\dfrac{1 - L_{CV,0}}{1 + r_{CV}\, L_{CV,0}}\right)$
Implementation Name: `kCV`
Units: mV^{-1}
Initial value: 0
Parameter controlling the sensitivity of Complex V flux to driving force.

$K_{eq_1,n}$
Implementation Name: `Keq1_n`
Units: dimensionless
Initial value: $10^{\frac{-1}{Z}\left(\frac{p_1 \Delta p_n}{4} - E_{1,n}\right)}$
Normal value of the equilibrium constant for the Cu_A reduction reaction.

$K_{eq_2,n}$
Implementation Name: `Keq2_n`
Units: dimensionless
Initial value: $10^{\frac{-1}{Z}\left(\frac{p_3 \Delta p_n}{4} - E_2\right)}$
Normal value of the equilibrium constant for the a_3 reduction reaction.

$L_{CV,0}$
Implementation Name: `L_CV0`
Units: dimensionless
Initial value: 0.4
Normal Complex V flux as a fraction of maximum possible flux.

$L_{CV,frac} = 1 - L_{lk,frac}$
Implementation Name: `L_CVfrac`
Units: dimensionless
Initial value: 0
Normal fraction of proton entry into mitochondria which is due to ADP phosphorylation.

$L_{CV,max} = \dfrac{L_{CV,n}}{L_{CV,0}}$
Implementation Name: `L_CVmax`
Units: $mM\ s^{-1}$
Initial value: 0
The maximum rate of proton flow through Complex V.

$L_{CV,n}$
Implementation Name: `L_CVn`
Units: $mM\ s^{-1}$
Initial value: $L_n\, L_{CV,frac}$
The resting flow of protons into the matrix through Complex V.

L_{lk0}
Implementation Name: `L_lk0`
Units: $mM\ s^{-1}$
Initial value: $\dfrac{L_{lk,n}}{\exp\left(\Delta p_n\, k_{lk2}\right) - 1}$
Constant controlling the depending of the leak rate L_{lk} on Δp.

$L_{lk,frac}$
Implementation Name: `L_lkfrac`
Units: dimensionless
Initial value: 0.25
Normal fraction of proton entry into mitochondria which is via leak channels.

$L_{lk,n}$
Implementation Name: `L_lkn`
Units: $mM\ s^{-1}$

Initial value: $L_n \, L_{lk,frac}$
The resting flow of protons into the matrix via leak channels.

$L_n = p_{tot} \, f_n$
Implementation Name: L_n
Units: mM s^{-1}
Initial value: 0
The normal total flow of protons back into mitochondria.

λ_0
Implementation Name: lam_0
Units: cm
Initial value: 0.02507
Intercept of the fitted linear model for blood vessel radius.

λ_μ
Implementation Name: lam_mu
Units: cm
Initial value: -0.0004422
Fitted linear dependence of blood vessel radius on autoregulatory stimuli.

λ_{P_a}
Implementation Name: lam_p
Units: cm mmHg
Initial value: -0.6327
Fitted linear dependence of blood vessel radius on reciprocal of blood pressure.

$\lambda_{P_a,\mu}$
Implementation Name: lam_p_mu
Units: cm mmHg
Initial value: -0.5286
Fitted joint dependence of blood vessel radius on autoregulatory stimuli and reciprocal of blood pressure.

n_h
Implementation Name: n_h
Units: dimensionless
Initial value: 2.5
Hill coefficient for oxygen dissociation from haemoglobin.

$NADH = \dfrac{NAD_{pool}}{1 + \frac{NAD}{NADH}}$
Implementation Name: NADH
Units: mM
Initial value: 0
Concentration of NADH in the mitochondria.

$NADH_n$
Implementation Name: NADHn
Units: mM
Initial value: $\dfrac{NAD_{pool}}{1 + \frac{NAD_n}{NADH_n}}$
Normal concentration of NADH in the mitochondria.

$\frac{NAD}{NADH}$
Implementation Name: NADNADHrat
Units: dimensionless
Initial value: $\dfrac{\frac{NAD_n}{NADH_n}}{\text{pow}\,(u, 2D_{NADH})}$
NAD/NADH ratio.

$\frac{NAD_n}{NADH_n}$
Implementation Name: `NADNADHratn`
Units: dimensionless
Initial value: 9
Normal NAD/NADH ratio.

NAD_{pool}
Implementation Name: `NADpool`
Units: dimensionless
Initial value: 3
Relative size of the NAD pool, used to estimate normal mitochondrial NADH.

$O_{2,n}$
Implementation Name: `O2_n`
Units: mM
Initial value: 0.024
Normal mitochondrial oxygen concentration.

$O_{2,c,n}$
Implementation Name: `O2c_n`
Units: mM
Initial value: $\phi\,\mathrm{pow}\left(\frac{S_{c,O_2,n}}{1-S_{c,O_2,n}}, \frac{1}{n_h}\right)$
Normal capillary oxygen concentration.

$p_1 \ = p_{tot} - p_{23}$
Implementation Name: `p1`
Units: dimensionless
Initial value: 0
Proton cost of the reaction reducing Cu_A.

p_3
Implementation Name: `p2`
Units: dimensionless
Initial value: 4
Proton cost of the reaction reducing a_3.

p_{23}
Implementation Name: `p23`
Units: dimensionless
Initial value: 8
Total protons removed from the mitochondrial matrix by the reductions of a_3 and O_2.

p_3
Implementation Name: `p3`
Units: dimensionless
Initial value: $p_{23} - p_3$
Proton cost of the reaction reducing O_2.

P_a
Implementation Name: `P_a`
Units: mmHg
Initial value: $P_{a,n}$
Mean arterial blood pressure.

$P_{a,n}$
Implementation Name: `P_an`
Units: mmHg
Initial value: 100
Normal arterial blood pressure.

p_{C1}
Implementation Name: p_C1
Units: dimensionless
Initial value: 8
Protons pumped by Complex I.

p_{C3}
Implementation Name: p_C3
Units: dimensionless
Initial value: 4
Protons pumped by Complex III.

p_{tot}
Implementation Name: p_tot
Units: dimensionless
Initial value: $p_{tot,NADH}$
Total protons removed from the mitochondrial matrix by the three modelled electron transport reactions.

$p_{tot,NADH} = p_{C1} + p_{C3} + p_{23}$
Implementation Name: p_totNADH
Units: dimensionless
Initial value: 0
Total protons pumped when the reducing agent is NADH.

P_v
Implementation Name: P_v
Units: mmHg
Initial value: $P_{v,n}$
Venous blood pressure.

$P_{v,n}$
Implementation Name: P_vn
Units: mmHg
Initial value: 4
Normal venous blood pressure.

Pa_{CO_2}
Implementation Name: Pa_CO2
Units: mmHg
Initial value: $Pa_{CO_{2,n}}$
Arterial partial pressure of carbon dioxide.

$Pa_{CO_{2,n}}$
Implementation Name: Pa_CO2n
Units: mmHg
Initial value: 40
Normal arterial partial pressure of carbon dioxide.

$pH_{m,n}$
Implementation Name: pH_mn
Units: dimensionless
Initial value: 7.4
Normal mitochondrial pH.

pH_o
Implementation Name: pH_o
Units: dimensionless
Initial value: 7
Extra-mitochondrial pH.

$pH_{o,n}$
Implementation Name: `pH_on`
Units: dimensionless
Initial value: 7
Normal extra-mitochondrial pH.

ϕ
Implementation Name: `phi`
Units: mM
Initial value: 0.036
Oxygen concentration at half-maximal saturation.

R_{CO_2}
Implementation Name: `R_autc`
Units: dimensionless
Initial value: 2.2
Autoregulatory reactivity to carbon dioxide.

R_{O_2}
Implementation Name: `R_auto`
Units: dimensionless
Initial value: 1.5
Autoregulatory reactivity to oxygen.

R_{P_a}
Implementation Name: `R_autp`
Units: dimensionless
Initial value: 4
Autoregulatory reactivity to blood pressure.

R_u
Implementation Name: `R_autu`
Units: dimensionless
Initial value: 0.5
Autoregulatory reactivity to demand.

r_{CV}
Implementation Name: `r_CV`
Units: dimensionless
Initial value: 5
Parameter controlling the ratio of maximal to minimal rates of oxidative phosphorylation.

r_n
Implementation Name: `r_n`
Units: cm
Initial value: 0.0187
Normal blood vessel radius. Normal effective blood vessel radius.

$S_{a,O_2,n}$
Implementation Name: `SaO2_n`
Units: dimensionless
Initial value: 0.96
Normal arterial oxygen saturation.

S_{a,O_2}
Implementation Name: `SaO2sup`
Units: dimensionless
Initial value: $S_{a,O_2,n}$
Arterial oxygen saturation.

$S_{c,O_2,n}$
Implementation Name: `ScO2_n`
Units: dimensionless
Initial value: $\dfrac{S_{a,O_2,n} + S_{v,O_2,n}}{2}$
Normal capillary oxygen saturation.

$S_{v,O_2,n}$
Implementation Name: `SvO2_n`
Units: dimensionless
Initial value: $\dfrac{HbO_{2,v,n}}{Hb_{tot,n}}$
Normal venous oxygen saturation.

t
Implementation Name: `t`
Units: s
Initial value: 0
Time over which the system evolves.

τ_{CO_2}
Implementation Name: `t_c`
Units: s
Initial value: 5
Filter time constant for stimulus effect of carbon dioxide.

τ_{O_2}
Implementation Name: `t_o`
Units: s
Initial value: 20
Filter time constant for stimulus effect of capillary oxygen.

τ_{P_a}
Implementation Name: `t_p`
Units: s
Initial value: 5
Filter time constant for stimulus effect of blood pressure.

τ_u
Implementation Name: `t_u`
Units: s
Initial value: 0.5
Filter time constant for stimulus effect of demand.

u
Implementation Name: `u`
Units: dimensionless
Initial value: u_n
Parameter indicating metabolic demand.

u_n
Implementation Name: `u_n`
Units: dimensionless
Initial value: 1
Normal demand.

$v_{CO_2,n}$
Implementation Name: `v_cn`
Units: mmHg
Initial value: $Pa_{CO_{2,n}}$

Normal filtered carbon dioxide partial pressure. Normal filtered carbon dioxide partial pressure.

$v_{O_2,n}$
Implementation Name: `v_on`
Units: mM
Initial value: $O_{2,c,n}$
Normal filtered capillary oxygen concentration. Normal filtered capillary oxygen concentration.

$v_{P_a,n}$
Implementation Name: `v_pn`
Units: mmHg
Initial value: $P_{a,n}$
Normal filtered arterial blood pressure. Normal filtered blood pressure.

$v_{u,n}$
Implementation Name: `v_un`
Units: dimensionless
Initial value: u_n
Normal filtered demand. Normal filtered demand.

$VArat_n$
Implementation Name: `VArat_n`
Units: dimensionless
Initial value: 3
Normal volume ratio of veins to arteries in brain tissue.

$V_{a,n}$
Implementation Name: `Vol_artn`
Units: dimensionless
Initial value: $\frac{1}{1 + VArat_n}$
Normal relative arterial blood volume.

Vol_{mit}
Implementation Name: `Vol_mit`
Units: dimensionless
Initial value: 0.067
Fraction of brain tissue volume that is mitochondria.

V_v
Implementation Name: `Vol_ven`
Units: dimensionless
Initial value: $\frac{VArat_n}{1 + VArat_n}$
Relative venous blood volume.

$HbO_{2,a} = Hb_{tot} \, S_{a,O_2}$
Implementation Name: `XOa`
Units: mM
Initial value: $HbO_{2,a,n}$
Arterial concentration of oxygen bound to haemoglobin.

$HbO_{2,a,n}$
Implementation Name: `XOa_n`
Units: mM
Initial value: $Hb_{tot,n} \, S_{a,O_2,n}$
Normal arterial concentration of oxygen bound to haemoglobin.

$HbO_{2,v,n}$
Implementation Name: `XOv_n`

Units: mM
Initial value: $\frac{CBF_n\, HbO_{2,a,n} - J_{O_{2},n}}{CBF_n}$
Normal venous concentration of oxygen bound to haemoglobin.

Hb_{tot}
Implementation Name: `Xtot`
Units: mM
Initial value: 9.1
Total concentration of haemoglobin O_2 binding sites in blood (4 times haemoglobin concentration).

$Hb_{tot,n}$
Implementation Name: `Xtot_n`
Units: mM
Initial value: 9.1
Normal total concentration of haemoglobin O_2 binding sites in blood (4 times haemoglobin concentration).

Z
Implementation Name: `Z`
Units: mV
Initial value: 59.028
Proportionality constant in calculation of driving forces due to concentration differences. Defined as RT/F, where F is Faraday's constant, R the ideal gas constant and T the absolute temperature.

3 BSB2

3.1 Overview

Simplified model in which the blood flow submodel is replaced with variant B2.

- 9 differential state variables
- 3 algebraic state variables
- 35 intermediate variables
- 121 parameters
- 4 declared inputs
- 33 default outputs

3.2 Differential Equations

$$\frac{\mathrm{d}Cu_{A,o}}{\mathrm{d}t} = 4f_3 - 4f_1 \tag{3.1}$$

$$\frac{\mathrm{d}a_{3,r}}{\mathrm{d}t} = 4f_3 - 4f_3 \tag{3.2}$$

$$\frac{\mathrm{d}\psi}{\mathrm{d}t} = \frac{p_3\, f_3 + p_1\, f_1 + p_3\, f_3 - L}{C_{im}} \tag{3.3}$$

$$\frac{\mathrm{d}H^+}{\mathrm{d}t} = \frac{1}{R_{Hi}}\, L - \frac{p_3}{R_{Hi}}\, f_3 - \frac{p_1}{R_{Hi}}\, f_1 - \frac{p_3}{R_{Hi}}\, f_3 \tag{3.4}$$

$$\frac{\mathrm{d}O_2}{\mathrm{d}t} = \frac{1}{Vol_{mit}}\, J_{O_2} - f_3 \tag{3.5}$$

$$\frac{\mathrm{d}\nu_{CO_2}}{\mathrm{d}t} = \frac{1}{\tau_{CO_2}}\, \left(Pa_{CO_2} - \nu_{CO_2}\right) \tag{3.6}$$

$$\frac{\mathrm{d}\nu_{O_2}}{\mathrm{d}t} = \frac{1}{\tau_{O_2}}\, \left(O_{2,c} - \nu_{O_2}\right) \tag{3.7}$$

$$\frac{\mathrm{d}\nu_{P_a}}{\mathrm{d}t} = \frac{1}{\tau_{P_a}}\, \left(P_a - \nu_{P_a}\right) \tag{3.8}$$

$$\frac{\mathrm{d}\nu_u}{\mathrm{d}t} = \frac{1}{\tau_u}\,(u - \nu_u) \tag{3.9}$$

3.3 Algebraic Equations

$$\phi\left(\frac{S_{c,O_2}}{1 - S_{c,O_2}}\right)^{\frac{1}{n_h}} - O_{2,c} = 0 \tag{3.10}$$

$$\lambda_0 + \frac{\lambda_{P_a}}{P_a} + \lambda_\mu\,\mu - r = 0 \tag{3.11}$$

$$CBF\,(HbO_{2,a} - HbO_{2,v}) - J_{O_2} = 0 \tag{3.12}$$

3.4 Chemical Reactions

$$\xrightarrow{L} \frac{1}{R_{Hi}}\,\mathrm{H}^+ \tag{3.13}$$

$$\xrightarrow{J_{O_2}} \frac{1}{Vol_{mit}}\,\mathrm{O_2} \tag{3.14}$$

$$\frac{p_3}{R_{Hi}}\,\mathrm{H}^+ \xrightarrow{f_3} 4\,\mathrm{Cu_{A,o}} + 4\,\mathrm{a_{3,r}} \tag{3.15}$$

$$4\,\mathrm{Cu_{A,o}} + \frac{p_1}{R_{Hi}}\,\mathrm{H}^+ \xrightarrow{f_1} \tag{3.16}$$

$$\mathrm{O_2} + 4\,\mathrm{a_{3,r}} + \frac{p_3}{R_{Hi}}\,\mathrm{H}^+ \xrightarrow{f_3} \tag{3.17}$$

3.5 State Variables

$Cu_{A,o}$
: Implementation Name: `a`
Units: mM
Initial value: $Cu_{A,o,n}$
Concentration of oxidised cytochrome c oxidase.

$a_{3,r}$
: Implementation Name: `bred`
Units: mM
Initial value: $a_{3,r,n}$
Concentration of reduced cytochrome $\mathrm{a_3}$.

ψ
Implementation Name: `Dpsi`
Units: mV
Initial value: ψ_n
Mitochondrial inner membrane potential. Varies as charge (in the form of protons) is transferred across the membrane capacitance.

H^+
Implementation Name: `H`
Units: mM
Initial value: H^+_n
Mitochondrial proton concentration.

O_2
Implementation Name: `O2`
Units: mM
Initial value: $O_{2,n}$
Mitochondrial oxygen concentration.

$O_{2,c}$
Implementation Name: `O2c`
Units: mM
Initial value: $O_{2,c,n}$
Capillary oxygen concentration.

r
Implementation Name: `r`
Units: cm
Initial value: r_n
Typical blood vessel radius.

ν_{CO_2}
Implementation Name: `v_c`
Units: mmHg
Initial value: $\nu_{CO_2,n}$
Filtered carbon dioxide partial pressure.

ν_{O_2}
Implementation Name: `v_o`
Units: mM
Initial value: $\nu_{O_2,n}$
Filtered capillary oxygen concentration.

ν_{P_a}
Implementation Name: `v_p`
Units: mmHg
Initial value: $\nu_{P_a,n}$
Filtered arterial blood pressure.

ν_u
Implementation Name: `v_u`
Units: dimensionless
Initial value: $\nu_{u,n}$
Filtered demand.

$HbO_{2,v}$
Implementation Name: `XOv`
Units: mM
Initial value: $HbO_{2,v,n}$
Venous concentration of oxygen bound to haemoglobin.

3.6 Intermediate Variables

$Cu_{A,r} = CCO_{tot} - Cu_{A,o}$
Implementation Name: `ared`
Units: mM
Initial value: 0
Concentration of reduced Cu_A.

$a_{3,o} = CCO_{tot} - a_{3,r}$
Implementation Name: `b`
Units: mM
Initial value: 0
Concentration of oxidised cytochrome a_3.

$$C_{0,i} = \frac{10^{-pH_m} - 10^{-pH_m - dpH}}{dpH}$$
Implementation Name: `C_0i`
Units: dimensionless
Initial value: 0
Natural buffering capacity of protons in mitochondria.

$CBF = G\ (P_a - P_v)$
Implementation Name: `CBF`
Units: $\text{ml}_{blood}\ \text{ml}_{brain}^{-1}\ \text{s}^{-1}$
Initial value: CBF_n
Cerebral blood flow.

$\Delta oxCCO = \Delta oxCCO_{off} + 1000 Vol_{mit}\ (Cu_{A,o} - Cu_{A,o,n})$
Implementation Name: `CCO`
Units: uM
Initial value: 0
Cytochrome c oxidase signal measured by NIRS.

$CMRO_2 = f_3\, Vol_{mit}$
Implementation Name: `CMRO2`
Units: mM s^{-1}
Initial value: 0
Rate of cerebral oxygen metabolism.

$\Delta p = \psi + Z\ (pH_m - pH_o)$
Implementation Name: `Dp`
Units: mV
Initial value: 0
Proton motive force across the mitochondrial inner membrane.

$$\eta = R_{P_a}\left(\frac{v_{P_a}}{v_{P_a,n}} - 1\right) + R_{O_2}\left(\frac{v_{O_2}}{v_{O_2,n}} - 1\right) + R_{CO_2}\left(1 - \frac{v_{CO_2}}{v_{CO_2,n}}\right) + R_u\left(1 - \frac{v_u}{v_{u,n}}\right)$$
Implementation Name: `eta`
Units: dimensionless
Initial value: 0
Merged autoregulation stimulus.

$f_1 = k_1\, Cu_{A,o} - k_{-1}\, Cu_{A,r}$
Implementation Name: `f1`
Units: mM s^{-1}
Initial value: 0
Reaction rate for the reduction of Cu_A.

$f_3 = k_2\, Cu_{A,r}\, a_{3,o} - k_{-2}\, Cu_{A,o}\, a_{3,r}$
Implementation Name: `f2`

Units: mM s^{-1}
Initial value: 0
Reaction rate for the reduction of a_3.

$f_3 = \frac{k_3\, O_2\, a_{3,r} \exp\left(-c_3\ (\Delta p - \Delta p_{30})\right)}{1 + \exp\left(-c_3\ (\Delta p - \Delta p_{30})\right)}$
Implementation Name: `f3`
Units: mM s^{-1}
Initial value: 0
Reaction rate for the reduction of O_2.

$G = K_G\, r^4$
Implementation Name: `G`
Units: $ml_{blood}\ ml_{brain}^{-1}\ mmHg^{-1}\ s^{-1}$
Initial value: 0
Effective conductance of the whole blood flow compartment.

$HbO_2 = (V_a\, HbO_{2,a} + V_v\, HbO_{2,v})\ blood_{hb}$
Implementation Name: `HbO2`
Units: uM
Initial value: 0
Oxygenated haemoglobin signal measured by NIRS.

$HbT = (V_a + V_v)\ Hb_{tot}\, blood_{hb}$
Implementation Name: `HbT`
Units: uM
Initial value: 0
Total haemoglobin signal measured by NIRS.

$HHb = HbT - HbO_2$
Implementation Name: `HHb`
Units: uM
Initial value: 0
Deoxygenated haemoglobin signal measured by NIRS.

$J_{O_2} = \text{fmin}\left(D_{O_2}\ (O_{2,c} - O_2)\,, CBF\, HbO_{2,a}\right)$
Implementation Name: `J_O2`
Units: mM s^{-1}
Initial value: 0
Oxygen flux from blood to tissue.

$k_1 = k_{1,0} \exp\left(-c_{k_1}\ (\Delta p - \Delta p_n)\right)$
Implementation Name: `k1`
Units: s^{-1}
Initial value: 0
Forward reaction rate for the reduction of Cu_A.

$k_2 = k_{2,n} \exp\left(-c_{k_2}\ (\Delta p - \Delta p_n)\right)$
Implementation Name: `k2`
Units: s^{-1}
Initial value: 0
Forward reaction rate for the reduction of a_3.

$K_{eq_1} = 10^{\frac{-1}{Z}\left(\frac{p_1\,\Delta p}{4} - E_1\right)}$
Implementation Name: `Keq1`
Units: dimensionless
Initial value: 0
Equilibrium constant for the Cu_A reduction reaction.

$K_{eq_2} = 10^{\frac{-1}{Z}\left(\frac{p_3\,\Delta p}{4} - E_2\right)}$

Implementation Name: `Keq2`
Units: dimensionless
Initial value: 0
Equilibrium constant for the a_3 reduction reaction.

$$k_{-1} = \frac{k_1}{K_{eq_1}}$$

Implementation Name: `kn1`
Units: s^{-1}
Initial value: 0
Reverse reaction rate for the reduction of Cu_A.

$$k_{-2} = \frac{k_2}{K_{eq_2}}$$

Implementation Name: `kn2`
Units: s^{-1}
Initial value: 0
Reverse reaction rate for the reduction of a_3.

$$L = L_{CV} + L_{lk}$$

Implementation Name: `L`
Units: mM s^{-1}
Initial value: 0
Rate of proton return to the mitochondrial matrix.

$$L_{CV} = \frac{CV_{inh}\, L_{CV,max}\, (1 - \exp(-\theta))}{1 + r_{CV} \exp(-\theta)}$$

Implementation Name: `L_CV`
Units: mM s^{-1}
Initial value: 0
Rate at which protons re-enter the mitochondrial matrix due to ADP phosphorylation.

$$L_{lk} = k_{unc}\, L_{lk0}\, (\exp(\Delta p\, k_{lk2}) - 1)$$

Implementation Name: `L_lk`
Units: mM s^{-1}
Initial value: 0
Rate at which protons re-enter the mitochondrial matrix via leak channels.

$$\mu = \frac{k_{aut}\, (\exp(\eta) - 1)}{\exp(\eta) + 1}$$

Implementation Name: `mu`
Units: dimensionless
Initial value: 0
Effective strength of the autoregulation reponse.

$$pH_m = -\log 10 \left(\frac{H^+}{1000}\right)$$

Implementation Name: `pH_m`
Units: dimensionless
Initial value: 0
Mitochondrial pH.

$$r_{buffi} = \frac{C_{buffi}}{C_{0,i}}$$

Implementation Name: `r_buffi`
Units: dimensionless
Initial value: 0
Buffering capacity for protons in mitochondria.

$$R_{Hi} = r_{buffi}$$

Implementation Name: `R_Hi`
Units: dimensionless
Initial value: 0
Relative mitochondrial volume for protons, taking into account buffering effect of pH.

$S_{c,O_2} = \frac{S_{a,O_2} + S_{v,O_2}}{2}$
Implementation Name: `ScO2`
Units: dimensionless
Initial value: $S_{c,O_2,n}$
Capillary oxygen saturation.

$S_{v,O_2} = \frac{HbO_{2,v}}{Hb_{tot}}$
Implementation Name: `SvO2`
Units: dimensionless
Initial value: $S_{v,O_2,n}$
Venous oxygen saturation.

$\theta = k_{CV} \left(\Delta p + Z \log10\left(u\right) - \Delta p_{CV,0}\right)$
Implementation Name: `theta`
Units: dimensionless
Initial value: 0
Driving force Complex V.

$TOI = \frac{100 HbO_2}{HbT}$
Implementation Name: `TOI`
Units: dimensionless
Initial value: 0
Total oxygenation index.

$V_{mca} = CBF\, CBFscale$
Implementation Name: `Vmca`
Units: cm s^{-1}
Initial value: 0
Blood velocity in the middle cerebral artery.

$V_a = V_{a,n} \left(\frac{r}{r_n}\right)^2$
Implementation Name: `Vol_art`
Units: dimensionless
Initial value: 0
Relative arterial blood volume.

3.7 Parameters

$Cu_{a,frac,n}$
Implementation Name: `a_frac_n`
Units: dimensionless
Initial value: 0.8
Normal oxidised fraction of Cu_A.

$Cu_{A,o,n}$
Implementation Name: `a_n`
Units: mM
Initial value: $CCO_{tot}\, Cu_{a,frac,n}$
Normal concentration of oxidised cytochrome c oxidase.

$Cu_{A,r,n}$
Implementation Name: `ared_n`
Units: mM
Initial value: $CCO_{tot} - Cu_{A,o,n}$
Normal concentration of reduced Cu_A.

$a_{3,o,n}$
Implementation Name: `b_n`
Units: mM
Initial value: $CCO_{tot} - a_{3,r,n}$
Normal concentration of oxidised cytochrome a_3.

$blood_{hb}$
Implementation Name: `blood_hb`
Units: dimensionless
Initial value: 10.00
Factor to convert model haemoglobin concentration to instrumental units. Scales for blood fraction of brain volume, mM to μM, and number of binding sites.

$a_{3,r,n}$
Implementation Name: `bred_n`
Units: mM
Initial value: $\frac{\frac{\frac{f_n}{k_3}}{O_{2,n}}}{\frac{\exp(-c_3\,(\Delta p_n - \Delta p_{30}))}{1+\exp(-c_3\,(\Delta p_n - \Delta p_{30}))}}$
Normal concentration of reduced cytochrome a3.

c_3
Implementation Name: `c3`
Units: mV^{-1}
Initial value: 0.11
Parameter controlling the sensitivity of the reduction of a_3 to Δp.

C_{buffi}
Implementation Name: `C_buffi`
Units: dimensionless
Initial value: 0.022
Buffering capacity of protons in mitochondria.

C_{im}
Implementation Name: `C_im`
Units: mM mV^{-1}
Initial value: 0.00675
Capacitance of the mitochondrial inner membrane.

$C_{NADH} = \frac{Z}{2}\log10\left(\frac{1}{\frac{NAD}{NADH}}\right)$
Implementation Name: `C_NADH`
Units: mV
Initial value: 0
Excess redox potential for NADH at normal demand.

$C_{NADH,n}$
Implementation Name: `C_NADH_n`
Units: mV
Initial value: $\frac{Z}{2}\log10\left(\frac{1}{\frac{NAD_n}{NADH_n}}\right)$
Normal value of C_{NADH}.

CBF_n
Implementation Name: `CBFn`
Units: $ml_{blood}\ ml_{brain}^{-1}\ s^{-1}$
Initial value: 0.0125
Normal cerebral blood flow.

$CBFscale$
Implementation Name: `CBFscale`
Units: cm
Initial value: 5000
Scale constant relating blood flow to arterial velocity.

$\Delta oxCCO_{off}$
Implementation Name: `CCO_offset`
Units: uM
Initial value: 0
Signal offset for the NIRS CCO measurement.

c_{k_1}
Implementation Name: `ck1`
Units: mV^{-1}
Initial value: 0.01
Parameter controlling sensitivity of k_1 to Δp.

c_{k_2}
Implementation Name: `ck2`
Units: mV^{-1}
Initial value: 0.02
Parameter controlling sensitivity of k_2 to Δp.

$CMRO_{2,n}$
Implementation Name: `CMRO2_n`
Units: $mM\ s^{-1}$
Initial value: 0.034
Normal metabolic rate of oxygen consumption.

CV_{inh}
Implementation Name: `CVinh`
Units: dimensionless
Initial value: 1
Control parameter representing the action of Complex V inhibitors.

$CCO_{tot} = \dfrac{CCO_{tis}}{Vol_{mit}}$
Implementation Name: `cytox_tot`
Units: mM
Initial value: 0
Concentration of cytochrome c oxidase in mitochondria.

CCO_{tis}
Implementation Name: `cytox_tot_tis`
Units: mM
Initial value: 0.0055
Concentration of cytochrome c oxidase in tissue.

D_{NADH}
Implementation Name: `D_NADH`
Units: dimensionless
Initial value: 0.01
Scale parameter for the dependence of NADH redox potential on demand.

D_{O_2}
Implementation Name: D_O2
Units: s^{-1}
Initial value: $\frac{J_{O_{2,n}}}{O_{2,c,n} - O_{2,n}}$
Diffusion rate for oxygen between capillaries and mitochondria.

$\Delta p_{3,corr}$
Implementation Name: Dp3_corr
Units: mV
Initial value: -25
Difference between Δp_{30} and normal Δp.

$\Delta p_{30} = \Delta p_n + \Delta p_{3,corr}$
Implementation Name: Dp_30
Units: mV
Initial value: 0
Value of Δp to which a_3 reduction reaction is maximally sensitive.

$\Delta p_{CV,0}$
Implementation Name: Dp_CV0
Units: mV
Initial value: 90
Value of Δp at which L_{CV} is zero under normal demand.

Δp_n
Implementation Name: Dp_n
Units: mV
Initial value: $\psi_n + Z\,\Delta pH_n$
Normal value of Δp

dpH
Implementation Name: dpH
Units: dimensionless
Initial value: 0.001
Parameter in the mitochondrial proton buffering relationship.

ΔpH_n
Implementation Name: DpH_n
Units: dimensionless
Initial value: $pH_{m,n} - pH_{o,n}$
Normal pH difference across the mitochondrial inner membrane.

ψ_n
Implementation Name: Dpsi_n
Units: mV
Initial value: 145
Normal mitochondrial inner membrane potential.

$E_{1,NADH} = \mathcal{E}_0(\mathrm{Cu}_A) - \mathcal{E}_0(\mathrm{NADH}) + C_{NADH}$
Implementation Name: E1NADH
Units: mV
Initial value: 0
Value of E_1 when the reducing substrate is NADH.

$E_{1,NADH,n}$
Implementation Name: E1NADH_n
Units: mV
Initial value: $\mathcal{E}_0(\mathrm{Cu}_A) - \mathcal{E}_0(\mathrm{NADH}) + C_{NADH,n}$
Normal value of $E_{1,NADH}$.

E_1
Implementation Name: `E_1`
Units: mV
Initial value: $E_{1,NADH}$
The energy provided by electron transfer to $Cu_{A,r}$.

$E_{1,n}$
Implementation Name: `E_1n`
Units: mV
Initial value: $E_{1,NADH,n}$
Normal value of E_1.

E_2
Implementation Name: `E_2`
Units: mV
Initial value: $\mathcal{E}_0(a_3) - \mathcal{E}_0(Cu_A)$
Energy provided by the transfer of four electrons from $Cu_{A,r}$ to $a_{3,o}$.

$\mathcal{E}_0(a_3)$
Implementation Name: `E_a30`
Units: mV
Initial value: 350
Standard redox potential for cytochrome a_3.

$\mathcal{E}_0(Cu_A)$
Implementation Name: `E_c0`
Units: mV
Initial value: 247
Standard redox potential for Cu_A.

$\mathcal{E}_0$(NADH)
Implementation Name: `E_N0`
Units: mV
Initial value: -320
Standard redox potential for NADH.

f_n
Implementation Name: `f_n`
Units: mM s^{-1}
Initial value: $\frac{CMRO_{2,n}}{Vol_{mit}}$
Normal resting value of f_1 and f_2.

G_n
Implementation Name: `Gn`
Units: ml_{blood} ml_{brain}^{-1} $mmHg^{-1}$ s^{-1}
Initial value: $\frac{CBF_n}{P_{a,n} - P_{v,n}}$
Normal blood vessel conductance.

H_n^+
Implementation Name: `H_n`
Units: mM
Initial value: $10^{3-pH_{m,n}}$
Normal mitochondrial proton concentration.

$J_{O_{2,n}}$
Implementation Name: `J_O2n`
Units: mM s^{-1}
Initial value: $CMRO_{2,n}$
Normal oxygen flux from blood to tissue.

$k_{1,0}$
Implementation Name: `k10`
Units: s^{-1}
Initial value: $\frac{k_{1,n}\, NADH}{NADH_n}$
Forward reaction rate for the reduction of Cu_A at normal Δp.

$k_{1,n}$
Implementation Name: `k1_n`
Units: s^{-1}
Initial value: $\frac{f_n}{Cu_{A,o,n} - \frac{1}{K_{eq_1,n}}\, Cu_{A,r,n}}$
Forward reaction rate for the reduction of Cu_A at normal Δp and NADH.

$k_{2,n}$
Implementation Name: `k2_n`
Units: s^{-1}
Initial value: $\frac{f_n}{Cu_{A,r,n}\, a_{3,o,n} - \frac{1}{K_{eq_2,n}}\, Cu_{A,o,n}\, a_{3,r,n}}$
Normal forward reaction rate for the reduction of a_3.

$k_3 = \frac{k_{3,0}}{\frac{\exp(-c_3 - \Delta p_{30})}{1+\exp(-c_3 - \Delta p_{30})}}$
Implementation Name: `k3`
Units: s^{-1}
Initial value: 0
Forward reaction rate for the reduction of O_2.

$k_{3,0}$
Implementation Name: `k30`
Units: s^{-1}
Initial value: $2.5E + 5$
Apparent second order rate constant for reduction of O_2 at zero Δp.

k_{aut}
Implementation Name: `k_aut`
Units: dimensionless
Initial value: 1
Overall functioning of autoregulatory response.

K_G
Implementation Name: `K_G`
Units: $ml_{blood}\ ml_{brain}^{-1}\ mmHg^{-1}\ s^{-1}\ cm^{-4}$
Initial value: $\frac{G_n}{\text{pow}\,(r_n, 4)}$
Proportionality constant in Poiseuille relation for conductance.

k_{lk2}
Implementation Name: `k_lk2`
Units: mV^{-1}
Initial value: 0.038
Constant controlling the depending of the leak rate L_{lk} on Δp.

k_{unc}
Implementation Name: `k_unc`
Units: dimensionless
Initial value: 1
Control parameter simulating the effect of adding uncouplers to the system.

$k_{CV} = \dfrac{-1}{\Delta p_n - \Delta p_{CV,0}} \log\left(\dfrac{1 - L_{CV,0}}{1 + r_{CV}\, L_{CV,0}}\right)$
Implementation Name: `kCV`
Units: mV^{-1}
Initial value: 0
Parameter controlling the sensitivity of Complex V flux to driving force.

$K_{eq_1,n}$
Implementation Name: `Keq1_n`
Units: dimensionless
Initial value: $10^{\frac{-1}{Z}\left(\frac{p_1\,\Delta p_n}{4} - E_{1,n}\right)}$
Normal value of the equilibrium constant for the Cu_A reduction reaction.

$K_{eq_2,n}$
Implementation Name: `Keq2_n`
Units: dimensionless
Initial value: $10^{\frac{-1}{Z}\left(\frac{p_3\,\Delta p_n}{4} - E_2\right)}$
Normal value of the equilibrium constant for the a_3 reduction reaction.

$L_{CV,0}$
Implementation Name: `L_CV0`
Units: dimensionless
Initial value: 0.4
Normal Complex V flux as a fraction of maximum possible flux.

$L_{CV,frac} = 1 - L_{lk,frac}$
Implementation Name: `L_CVfrac`
Units: dimensionless
Initial value: 0
Normal fraction of proton entry into mitochondria which is due to ADP phosphorylation.

$L_{CV,max} = \dfrac{L_{CV,n}}{L_{CV,0}}$
Implementation Name: `L_CVmax`
Units: $\mathrm{mM\ s}^{-1}$
Initial value: 0
The maximum rate of proton flow through Complex V.

$L_{CV,n}$
Implementation Name: `L_CVn`
Units: $\mathrm{mM\ s}^{-1}$
Initial value: $L_n\, L_{CV,frac}$
The resting flow of protons into the matrix through Complex V.

L_{lk0}
Implementation Name: `L_lk0`
Units: $\mathrm{mM\ s}^{-1}$
Initial value: $\dfrac{L_{lk,n}}{\exp\left(\Delta p_n\, k_{lk2}\right) - 1}$
Constant controlling the depending of the leak rate L_{lk} on Δp.

$L_{lk,frac}$
Implementation Name: `L_lkfrac`
Units: dimensionless
Initial value: 0.25
Normal fraction of proton entry into mitochondria which is via leak channels.

$L_{lk,n}$
Implementation Name: `L_lkn`
Units: $\mathrm{mM\ s}^{-1}$

Initial value: $L_n\, L_{lk,frac}$
The resting flow of protons into the matrix via leak channels.

$L_n \;= p_{tot}\, f_n$
Implementation Name: `L_n`
Units: mM s^{-1}
Initial value: 0
The normal total flow of protons back into mitochondria.

λ_0
Implementation Name: `lam_0`
Units: cm
Initial value: 0.02327
Intercept of the fitted linear model for blood vessel radius.

λ_μ
Implementation Name: `lam_mu`
Units: cm
Initial value: -0.006375
Fitted linear dependence of blood vessel radius on autoregulatory stimuli.

λ_{P_a}
Implementation Name: `lam_p`
Units: cm mmHg
Initial value: -0.4697
Fitted linear dependence of blood vessel radius on reciprocal of blood pressure.

n_h
Implementation Name: `n_h`
Units: dimensionless
Initial value: 2.5
Hill coefficient for oxygen dissociation from haemoglobin.

$NADH \;= \dfrac{NAD_{pool}}{1 + \frac{NAD}{NADH}}$
Implementation Name: `NADH`
Units: mM
Initial value: 0
Concentration of NADH in the mitochondria.

$NADH_n$
Implementation Name: `NADHn`
Units: mM
Initial value: $\dfrac{NAD_{pool}}{1 + \frac{NAD_n}{NADH_n}}$
Normal concentration of NADH in the mitochondria.

$\frac{NAD}{NADH}$
Implementation Name: `NADNADHrat`
Units: dimensionless
Initial value: $\dfrac{\frac{NAD_n}{NADH_n}}{\mathrm{pow}\,(u, 2D_{NADH})}$
NAD/NADH ratio.

$\frac{NAD_n}{NADH_n}$
Implementation Name: `NADNADHratn`
Units: dimensionless
Initial value: 9
Normal NAD/NADH ratio.

NAD_{pool}
Implementation Name: NADpool
Units: dimensionless
Initial value: 3
Relative size of the NAD pool, used to estimate normal mitochondrial NADH.

$O_{2,n}$
Implementation Name: O2_n
Units: mM
Initial value: 0.024
Normal mitochondrial oxygen concentration.

$O_{2,c,n}$
Implementation Name: O2c_n
Units: mM
Initial value: $\phi \, \mathrm{pow}\left(\frac{S_{c,O_2,n}}{1 - S_{c,O_2,n}}, \frac{1}{n_h}\right)$
Normal capillary oxygen concentration.

$p_1 \; = p_{tot} - p_{23}$
Implementation Name: p1
Units: dimensionless
Initial value: 0
Proton cost of the reaction reducing Cu_A.

p_3
Implementation Name: p2
Units: dimensionless
Initial value: 4
Proton cost of the reaction reducing a_3.

p_{23}
Implementation Name: p23
Units: dimensionless
Initial value: 8
Total protons removed from the mitochondrial matrix by the reductions of a_3 and O_2.

p_3
Implementation Name: p3
Units: dimensionless
Initial value: $p_{23} - p_3$
Proton cost of the reaction reducing O_2.

P_a
Implementation Name: P_a
Units: mmHg
Initial value: $P_{a,n}$
Mean arterial blood pressure.

$P_{a,n}$
Implementation Name: P_an
Units: mmHg
Initial value: 100
Normal arterial blood pressure.

p_{C1}
Implementation Name: p_C1
Units: dimensionless
Initial value: 8
Protons pumped by Complex I.

p_{C3}
Implementation Name: p_C3
Units: dimensionless
Initial value: 4
Protons pumped by Complex III.

p_{tot}
Implementation Name: p_tot
Units: dimensionless
Initial value: $p_{tot,NADH}$
Total protons removed from the mitochondrial matrix by the three modelled electron transport reactions.

$p_{tot,NADH} = p_{C1} + p_{C3} + p_{23}$
Implementation Name: p_totNADH
Units: dimensionless
Initial value: 0
Total protons pumped when the reducing agent is NADH.

P_v
Implementation Name: P_v
Units: mmHg
Initial value: $P_{v,n}$
Venous blood pressure.

$P_{v,n}$
Implementation Name: P_vn
Units: mmHg
Initial value: 4
Normal venous blood pressure.

Pa_{CO_2}
Implementation Name: Pa_CO2
Units: mmHg
Initial value: $Pa_{CO_{2,n}}$
Arterial partial pressure of carbon dioxide.

$Pa_{CO_{2,n}}$
Implementation Name: Pa_CO2n
Units: mmHg
Initial value: 40
Normal arterial partial pressure of carbon dioxide.

$pH_{m,n}$
Implementation Name: pH_mn
Units: dimensionless
Initial value: 7.4
Normal mitochondrial pH.

pH_o
Implementation Name: pH_o
Units: dimensionless
Initial value: 7
Extra-mitochondrial pH.

$pH_{o,n}$
Implementation Name: pH_on
Units: dimensionless
Initial value: 7
Normal extra-mitochondrial pH.

ϕ
Implementation Name: phi
Units: mM
Initial value: 0.036
Oxygen concentration at half-maximal saturation.

R_{CO_2}
Implementation Name: R_autc
Units: dimensionless
Initial value: 2.2
Autoregulatory reactivity to carbon dioxide.

R_{O_2}
Implementation Name: R_auto
Units: dimensionless
Initial value: 1.5
Autoregulatory reactivity to oxygen.

R_{P_a}
Implementation Name: R_autp
Units: dimensionless
Initial value: 4
Autoregulatory reactivity to blood pressure.

R_u
Implementation Name: R_autu
Units: dimensionless
Initial value: 0.5
Autoregulatory reactivity to demand.

r_{CV}
Implementation Name: r_CV
Units: dimensionless
Initial value: 5
Parameter controlling the ratio of maximal to minimal rates of oxidative phosphorylation.

r_n
Implementation Name: r_n
Units: cm
Initial value: 0.0187
Normal blood vessel radius. Normal effective blood vessel radius.

$S_{a,O_2,n}$
Implementation Name: SaO2_n
Units: dimensionless
Initial value: 0.96
Normal arterial oxygen saturation.

S_{a,O_2}
Implementation Name: SaO2sup
Units: dimensionless
Initial value: $S_{a,O_2,n}$
Arterial oxygen saturation.

$S_{c,O_2,n}$
Implementation Name: ScO2_n
Units: dimensionless
Initial value: $\frac{S_{a,O_2,n} + S_{v,O_2,n}}{2}$
Normal capillary oxygen saturation.

$S_{v,O_2,n}$
Implementation Name: `SvO2_n`
Units: dimensionless
Initial value: $\frac{HbO_{2,v,n}}{Hb_{tot,n}}$
Normal venous oxygen saturation.

t
Implementation Name: `t`
Units: s
Initial value: 0
Time over which the system evolves.

τ_{CO_2}
Implementation Name: `t_c`
Units: s
Initial value: 5
Filter time constant for stimulus effect of carbon dioxide.

τ_{O_2}
Implementation Name: `t_o`
Units: s
Initial value: 20
Filter time constant for stimulus effect of capillary oxygen.

τ_{P_a}
Implementation Name: `t_p`
Units: s
Initial value: 5
Filter time constant for stimulus effect of blood pressure.

τ_u
Implementation Name: `t_u`
Units: s
Initial value: 0.5
Filter time constant for stimulus effect of demand.

u
Implementation Name: `u`
Units: dimensionless
Initial value: u_n
Parameter indicating metabolic demand.

u_n
Implementation Name: `u_n`
Units: dimensionless
Initial value: 1
Normal demand.

$v_{CO_2,n}$
Implementation Name: `v_cn`
Units: mmHg
Initial value: $Pa_{CO_{2,n}}$
Normal filtered carbon dioxide partial pressure. Normal filtered carbon dioxide partial pressure.

$v_{O_2,n}$
Implementation Name: `v_on`
Units: mM
Initial value: $O_{2,c,n}$

Normal filtered capillary oxygen concentration. Normal filtered capillary oxygen concentration.

$v_{P_{a},n}$
Implementation Name: v_pn
Units: mmHg
Initial value: $P_{a,n}$
Normal filtered arterial blood pressure. Normal filtered blood pressure.

$v_{u,n}$
Implementation Name: v_un
Units: dimensionless
Initial value: u_n
Normal filtered demand. Normal filtered demand.

$VArat_n$
Implementation Name: VArat_n
Units: dimensionless
Initial value: 3
Normal volume ratio of veins to arteries in brain tissue.

$V_{a,n}$
Implementation Name: Vol_artn
Units: dimensionless
Initial value: $\frac{1}{1 + VArat_n}$
Normal relative arterial blood volume.

Vol_{mit}
Implementation Name: Vol_mit
Units: dimensionless
Initial value: 0.067
Fraction of brain tissue volume that is mitochondria.

V_v
Implementation Name: Vol_ven
Units: dimensionless
Initial value: $\frac{VArat_n}{1 + VArat_n}$
Relative venous blood volume.

$HbO_{2,a} = Hb_{tot}\, S_{a,O_2}$
Implementation Name: XOa
Units: mM
Initial value: $HbO_{2,a,n}$
Arterial concentration of oxygen bound to haemoglobin.

$HbO_{2,a,n}$
Implementation Name: XOa_n
Units: mM
Initial value: $Hb_{tot,n}\, S_{a,O_2,n}$
Normal arterial concentration of oxygen bound to haemoglobin.

$HbO_{2,v,n}$
Implementation Name: XOv_n
Units: mM
Initial value: $\frac{CBF_n\, HbO_{2,a,n} - J_{O_{2,n}}}{CBF_n}$
Normal venous concentration of oxygen bound to haemoglobin.

Hb_{tot}
Implementation Name: Xtot

Units: mM
Initial value: 9.1
Total concentration of haemoglobin O_2 binding sites in blood (4 times haemoglobin concentration).

$Hb_{tot,n}$
Implementation Name: `Xtot_n`
Units: mM
Initial value: 9.1
Normal total concentration of haemoglobin O_2 binding sites in blood (4 times haemoglobin concentration).

Z
Implementation Name: `Z`
Units: mV
Initial value: 59.028
Proportionality constant in calculation of driving forces due to concentration differences. Defined as RT/F, where F is Faraday's constant, R the ideal gas constant and T the absolute temperature.

4 BSB3

4.1 Overview

Simplified model in which the blood flow submodel is replaced with variant B3.

- 9 differential state variables
- 3 algebraic state variables
- 35 intermediate variables
- 120 parameters
- 4 declared inputs
- 33 default outputs

4.2 Differential Equations

$$\frac{\mathrm{d}Cu_{A,o}}{\mathrm{d}t} = 4f_3 - 4f_1 \tag{4.1}$$

$$\frac{\mathrm{d}a_{3,r}}{\mathrm{d}t} = 4f_3 - 4f_3 \tag{4.2}$$

$$\frac{\mathrm{d}\psi}{\mathrm{d}t} = \frac{p_3\, f_3 + p_1\, f_1 + p_3\, f_3 - L}{C_{im}} \tag{4.3}$$

$$\frac{\mathrm{d}H^+}{\mathrm{d}t} = \frac{1}{R_{Hi}}\, L - \frac{p_3}{R_{Hi}}\, f_3 - \frac{p_1}{R_{Hi}}\, f_1 - \frac{p_3}{R_{Hi}}\, f_3 \tag{4.4}$$

$$\frac{\mathrm{d}O_2}{\mathrm{d}t} = \frac{1}{Vol_{mit}}\, J_{O_2} - f_3 \tag{4.5}$$

$$\frac{\mathrm{d}\nu_{CO_2}}{\mathrm{d}t} = \frac{1}{\tau_{CO_2}}\, \left(Pa_{CO_2} - \nu_{CO_2}\right) \tag{4.6}$$

$$\frac{\mathrm{d}\nu_{O_2}}{\mathrm{d}t} = \frac{1}{\tau_{O_2}}\, \left(O_{2,c} - \nu_{O_2}\right) \tag{4.7}$$

$$\frac{\mathrm{d}\nu_{P_a}}{\mathrm{d}t} = \frac{1}{\tau_{P_a}}\, \left(P_a - \nu_{P_a}\right) \tag{4.8}$$

$$\frac{\mathrm{d}\nu_u}{\mathrm{d}t} = \frac{1}{\tau_u}\,(u - \nu_u) \tag{4.9}$$

4.3 Algebraic Equations

$$\phi\left(\frac{S_{c,O_2}}{1 - S_{c,O_2}}\right)^{\frac{1}{n_h}} - O_{2,c} = 0 \tag{4.10}$$

$$\lambda_0 + \lambda_\mu\,\mu - r = 0 \tag{4.11}$$

$$CBF\,(HbO_{2,a} - HbO_{2,v}) - J_{O_2} = 0 \tag{4.12}$$

4.4 Chemical Reactions

$$\xrightarrow{L} \frac{1}{R_{Hi}}\,\mathrm{H^+} \tag{4.13}$$

$$\xrightarrow{J_{O_2}} \frac{1}{Vol_{mit}}\,\mathrm{O_2} \tag{4.14}$$

$$\frac{p_3}{R_{Hi}}\,\mathrm{H^+} \xrightarrow{f_3} 4\,\mathrm{Cu_{A,o}} + 4\,\mathrm{a_{3,r}} \tag{4.15}$$

$$4\,\mathrm{Cu_{A,o}} + \frac{p_1}{R_{Hi}}\,\mathrm{H^+} \xrightarrow{f_1} \tag{4.16}$$

$$\mathrm{O_2} + 4\,\mathrm{a_{3,r}} + \frac{p_3}{R_{Hi}}\,\mathrm{H^+} \xrightarrow{f_3} \tag{4.17}$$

4.5 State Variables

$Cu_{A,o}$
Implementation Name: `a`
Units: mM
Initial value: $Cu_{A,o,n}$
Concentration of oxidised cytochrome c oxidase.

$a_{3,r}$
Implementation Name: `bred`
Units: mM
Initial value: $a_{3,r,n}$
Concentration of reduced cytochrome $\mathrm{a_3}$.

ψ
Implementation Name: `Dpsi`
Units: mV
Initial value: ψ_n
Mitochondrial inner membrane potential. Varies as charge (in the form of protons) is transferred across the membrane capacitance.

H^+
Implementation Name: `H`
Units: mM
Initial value: H_n^+
Mitochondrial proton concentration.

O_2
Implementation Name: `O2`
Units: mM
Initial value: $O_{2,n}$
Mitochondrial oxygen concentration.

$O_{2,c}$
Implementation Name: `O2c`
Units: mM
Initial value: $O_{2,c,n}$
Capillary oxygen concentration.

r
Implementation Name: `r`
Units: cm
Initial value: r_n
Typical blood vessel radius.

ν_{CO_2}
Implementation Name: `v_c`
Units: mmHg
Initial value: $\nu_{CO_2,n}$
Filtered carbon dioxide partial pressure.

ν_{O_2}
Implementation Name: `v_o`
Units: mM
Initial value: $\nu_{O_2,n}$
Filtered capillary oxygen concentration.

ν_{P_a}
Implementation Name: `v_p`
Units: mmHg
Initial value: $\nu_{P_a,n}$
Filtered arterial blood pressure.

ν_u
Implementation Name: `v_u`
Units: dimensionless
Initial value: $\nu_{u,n}$
Filtered demand.

$HbO_{2,v}$
Implementation Name: `XOv`
Units: mM
Initial value: $HbO_{2,v,n}$
Venous concentration of oxygen bound to haemoglobin.

4.6 Intermediate Variables

$Cu_{A,r} = CCO_{tot} - Cu_{A,o}$

Implementation Name: `ared`
Units: mM
Initial value: 0
Concentration of reduced Cu_A.

$a_{3,o} = CCO_{tot} - a_{3,r}$

Implementation Name: `b`
Units: mM
Initial value: 0
Concentration of oxidised cytochrome a_3.

$C_{0,i} = \frac{10^{-pH_m} - 10^{-pH_m - dpH}}{dpH}$

Implementation Name: `C_0i`
Units: dimensionless
Initial value: 0
Natural buffering capacity of protons in mitochondria.

$CBF = G\,(P_a - P_v)$

Implementation Name: `CBF`
Units: $ml_{blood}\ ml_{brain}^{-1}\ s^{-1}$
Initial value: CBF_n
Cerebral blood flow.

$\Delta oxCCO = \Delta oxCCO_{off} + 1000 Vol_{mit}\,(Cu_{A,o} - Cu_{A,o,n})$

Implementation Name: `CCO`
Units: uM
Initial value: 0
Cytochrome c oxidase signal measured by NIRS.

$CMRO_2 = f_3\, Vol_{mit}$

Implementation Name: `CMRO2`
Units: mM s^{-1}
Initial value: 0
Rate of cerebral oxygen metabolism.

$\Delta p = \psi + Z\,(pH_m - pH_o)$

Implementation Name: `Dp`
Units: mV
Initial value: 0
Proton motive force across the mitochondrial inner membrane.

$\eta = R_{P_a}\left(\frac{v_{P_a}}{v_{P_a,n}} - 1\right) + R_{O_2}\left(\frac{v_{O_2}}{v_{O_2,n}} - 1\right) + R_{CO_2}\left(1 - \frac{v_{CO_2}}{v_{CO_2,n}}\right) + R_u\left(1 - \frac{v_u}{v_{u,n}}\right)$

Implementation Name: `eta`
Units: dimensionless
Initial value: 0
Merged autoregulation stimulus.

$f_1 = k_1\, Cu_{A,o} - k_{-1}\, Cu_{A,r}$

Implementation Name: `f1`
Units: mM s^{-1}
Initial value: 0
Reaction rate for the reduction of Cu_A.

$f_3 = k_2\, Cu_{A,r}\, a_{3,o} - k_{-2}\, Cu_{A,o}\, a_{3,r}$

Implementation Name: `f2`

Units: mM s^{-1}
Initial value: 0
Reaction rate for the reduction of a_3.

$f_3 = \frac{k_3\, O_2\, a_{3,r} \exp\left(-c_3\, \left(\Delta p - \Delta p_{30}\right)\right)}{1 + \exp\left(-c_3\, \left(\Delta p - \Delta p_{30}\right)\right)}$
Implementation Name: `f3`
Units: mM s^{-1}
Initial value: 0
Reaction rate for the reduction of O_2.

$G = K_G\, r^4$
Implementation Name: `G`
Units: ml_{blood} ml_{brain}^{-1} $mmHg^{-1}$ s^{-1}
Initial value: 0
Effective conductance of the whole blood flow compartment.

$HbO_2 = \left(V_a\, HbO_{2,a} + V_v\, HbO_{2,v}\right)\, blood_{hb}$
Implementation Name: `HbO2`
Units: uM
Initial value: 0
Oxygenated haemoglobin signal measured by NIRS.

$HbT = \left(V_a + V_v\right)\, Hb_{tot}\, blood_{hb}$
Implementation Name: `HbT`
Units: uM
Initial value: 0
Total haemoglobin signal measured by NIRS.

$HHb = HbT - HbO_2$
Implementation Name: `HHb`
Units: uM
Initial value: 0
Deoxygenated haemoglobin signal measured by NIRS.

$J_{O_2} = \mathrm{fmin}\left(D_{O_2}\, \left(O_{2,c} - O_2\right), CBF\, HbO_{2,a}\right)$
Implementation Name: `J_O2`
Units: mM s^{-1}
Initial value: 0
Oxygen flux from blood to tissue.

$k_1 = k_{1,0} \exp\left(-c_{k_1}\, \left(\Delta p - \Delta p_n\right)\right)$
Implementation Name: `k1`
Units: s^{-1}
Initial value: 0
Forward reaction rate for the reduction of Cu_A.

$k_2 = k_{2,n} \exp\left(-c_{k_2}\, \left(\Delta p - \Delta p_n\right)\right)$
Implementation Name: `k2`
Units: s^{-1}
Initial value: 0
Forward reaction rate for the reduction of a_3.

$K_{eq_1} = 10^{\frac{-1}{Z}\left(\frac{p_1\, \Delta p}{4} - E_1\right)}$
Implementation Name: `Keq1`
Units: dimensionless
Initial value: 0
Equilibrium constant for the Cu_A reduction reaction.

$K_{eq_2} = 10^{\frac{-1}{Z}\left(\frac{p_3\, \Delta p}{4} - E_2\right)}$

Implementation Name: Keq2
Units: dimensionless
Initial value: 0
Equilibrium constant for the a_3 reduction reaction.

$k_{-1} = \frac{k_1}{K_{eq_1}}$
Implementation Name: kn1
Units: s^{-1}
Initial value: 0
Reverse reaction rate for the reduction of Cu_A.

$k_{-2} = \frac{k_2}{K_{eq_2}}$
Implementation Name: kn2
Units: s^{-1}
Initial value: 0
Reverse reaction rate for the reduction of a_3.

$L = L_{CV} + L_{lk}$
Implementation Name: L
Units: mM s^{-1}
Initial value: 0
Rate of proton return to the mitochondrial matrix.

$L_{CV} = \frac{CV_{inh}\, L_{CV,max}\, (1 - \exp(-\theta))}{1 + r_{CV} \exp(-\theta)}$
Implementation Name: L_CV
Units: mM s^{-1}
Initial value: 0
Rate at which protons re-enter the mitochondrial matrix due to ADP phosphorylation.

$L_{lk} = k_{unc}\, L_{lk0}\, (\exp(\Delta p\, k_{lk2}) - 1)$
Implementation Name: L_lk
Units: mM s^{-1}
Initial value: 0
Rate at which protons re-enter the mitochondrial matrix via leak channels.

$\mu = \frac{k_{aut}\, (\exp(\eta) - 1)}{\exp(\eta) + 1}$
Implementation Name: mu
Units: dimensionless
Initial value: 0
Effective strength of the autoregulation reponse.

$pH_m = -\log10\left(\frac{H^+}{1000}\right)$
Implementation Name: pH_m
Units: dimensionless
Initial value: 0
Mitochondrial pH.

$r_{buffi} = \frac{C_{buffi}}{C_{0,i}}$
Implementation Name: r_buffi
Units: dimensionless
Initial value: 0
Buffering capacity for protons in mitochondria.

$R_{Hi} = r_{buffi}$

Implementation Name: `R_Hi`
Units: dimensionless
Initial value: 0
Relative mitochondrial volume for protons, taking into account buffering effect of pH.

$S_{c,O_2} = \frac{S_{a,O_2} + S_{v,O_2}}{2}$
Implementation Name: `ScO2`
Units: dimensionless
Initial value: $S_{c,O_2,n}$
Capillary oxygen saturation.

$S_{v,O_2} = \frac{HbO_{2,v}}{Hb_{tot}}$
Implementation Name: `SvO2`
Units: dimensionless
Initial value: $S_{v,O_2,n}$
Venous oxygen saturation.

$\theta = k_{CV} \left(\Delta p + Z \log10 (u) - \Delta p_{CV,0}\right)$
Implementation Name: `theta`
Units: dimensionless
Initial value: 0
Driving force Complex V.

$TOI = \frac{100 HbO_2}{HbT}$
Implementation Name: `TOI`
Units: dimensionless
Initial value: 0
Total oxygenation index.

$V_{mca} = CBF\, CBFscale$
Implementation Name: `Vmca`
Units: cm s^{-1}
Initial value: 0
Blood velocity in the middle cerebral artery.

$V_a = V_{a,n} \left(\frac{r}{r_n}\right)^2$
Implementation Name: `Vol_art`
Units: dimensionless
Initial value: 0
Relative arterial blood volume.

4.7 Parameters

$Cu_{a,frac,n}$
Implementation Name: `a_frac_n`
Units: dimensionless
Initial value: 0.8
Normal oxidised fraction of Cu_A.

$Cu_{A,o,n}$
Implementation Name: `a_n`
Units: mM
Initial value: $CCO_{tot}\, Cu_{a,frac,n}$
Normal concentration of oxidised cytochrome c oxidase.

$Cu_{A,r,n}$
Implementation Name: `ared_n`
Units: mM
Initial value: $CCO_{tot} - Cu_{A,o,n}$
Normal concentration of reduced Cu_A.

$a_{3,o,n}$
Implementation Name: `b_n`
Units: mM
Initial value: $CCO_{tot} - a_{3,r,n}$
Normal concentration of oxidised cytochrome a_3.

$blood_{hb}$
Implementation Name: `blood_hb`
Units: dimensionless
Initial value: 10.00
Factor to convert model haemoglobin concentration to instrumental units. Scales for blood fraction of brain volume, mM to μM, and number of binding sites.

$a_{3,r,n}$
Implementation Name: `bred_n`
Units: mM
Initial value: $\frac{\frac{\frac{f_n}{k_3}}{O_{2,n}}}{\frac{\exp(-c_3(\Delta p_n - \Delta p_{30}))}{1+\exp(-c_3(\Delta p_n - \Delta p_{30}))}}$
Normal concentration of reduced cytochrome a3.

c_3
Implementation Name: `c3`
Units: mV^{-1}
Initial value: 0.11
Parameter controlling the sensitivity of the reduction of a_3 to Δp.

C_{buffi}
Implementation Name: `C_buffi`
Units: dimensionless
Initial value: 0.022
Buffering capacity of protons in mitochondria.

C_{im}
Implementation Name: `C_im`
Units: mM mV^{-1}
Initial value: 0.00675
Capacitance of the mitochondrial inner membrane.

$C_{NADH} = \frac{Z}{2}\log 10\left(\frac{1}{\frac{NAD}{NADH}}\right)$
Implementation Name: `C_NADH`
Units: mV
Initial value: 0
Excess redox potential for NADH at normal demand.

$C_{NADH,n}$
Implementation Name: `C_NADH_n`
Units: mV
Initial value: $\frac{Z}{2}\log 10\left(\frac{1}{\frac{NAD_n}{NADH_n}}\right)$
Normal value of C_{NADH}.

CBF_n
Implementation Name: `CBFn`
Units: $ml_{blood}\ ml_{brain}^{-1}\ s^{-1}$
Initial value: 0.0125
Normal cerebral blood flow.

$CBFscale$
Implementation Name: `CBFscale`
Units: cm
Initial value: 5000
Scale constant relating blood flow to arterial velocity.

$\Delta oxCCO_{off}$
Implementation Name: `CCO_offset`
Units: uM
Initial value: 0
Signal offset for the NIRS CCO measurement.

c_{k_1}
Implementation Name: `ck1`
Units: mV^{-1}
Initial value: 0.01
Parameter controlling sensitivity of k_1 to Δp.

c_{k_2}
Implementation Name: `ck2`
Units: mV^{-1}
Initial value: 0.02
Parameter controlling sensitivity of k_2 to Δp.

$CMRO_{2,n}$
Implementation Name: `CMRO2_n`
Units: $mM\ s^{-1}$
Initial value: 0.034
Normal metabolic rate of oxygen consumption.

CV_{inh}
Implementation Name: `CVinh`
Units: dimensionless
Initial value: 1
Control parameter representing the action of Complex V inhibitors.

$CCO_{tot} = \dfrac{CCO_{tis}}{Vol_{mit}}$
Implementation Name: `cytox_tot`
Units: mM
Initial value: 0
Concentration of cytochrome c oxidase in mitochondria.

CCO_{tis}
Implementation Name: `cytox_tot_tis`
Units: mM
Initial value: 0.0055
Concentration of cytochrome c oxidase in tissue.

D_{NADH}
Implementation Name: `D_NADH`
Units: dimensionless
Initial value: 0.01
Scale parameter for the dependence of NADH redox potential on demand.

D_{O_2}
Implementation Name: D_O2
Units: s^{-1}
Initial value: $\frac{J_{O_{2,n}}}{O_{2,c,n} - O_{2,n}}$
Diffusion rate for oxygen between capillaries and mitochondria.

$\Delta p_{3,corr}$
Implementation Name: Dp3_corr
Units: mV
Initial value: -25
Difference between Δp_{30} and normal Δp.

$\Delta p_{30} = \Delta p_n + \Delta p_{3,corr}$
Implementation Name: Dp_30
Units: mV
Initial value: 0
Value of Δp to which a_3 reduction reaction is maximally sensitive.

$\Delta p_{CV,0}$
Implementation Name: Dp_CV0
Units: mV
Initial value: 90
Value of Δp at which L_{CV} is zero under normal demand.

Δp_n
Implementation Name: Dp_n
Units: mV
Initial value: $\psi_n + Z\,\Delta pH_n$
Normal value of Δp

dpH
Implementation Name: dpH
Units: dimensionless
Initial value: 0.001
Parameter in the mitochondrial proton buffering relationship.

ΔpH_n
Implementation Name: DpH_n
Units: dimensionless
Initial value: $pH_{m,n} - pH_{o,n}$
Normal pH difference across the mitochondrial inner membrane.

ψ_n
Implementation Name: Dpsi_n
Units: mV
Initial value: 145
Normal mitochondrial inner membrane potential.

$E_{1,NADH} = \mathcal{E}_0(\mathrm{Cu}_A) - \mathcal{E}_0(\mathrm{NADH}) + C_{NADH}$
Implementation Name: E1NADH
Units: mV
Initial value: 0
Value of E_1 when the reducing substrate is NADH.

$E_{1,NADH,n}$
Implementation Name: E1NADH_n
Units: mV
Initial value: $\mathcal{E}_0(\mathrm{Cu}_A) - \mathcal{E}_0(\mathrm{NADH}) + C_{NADH,n}$
Normal value of $E_{1,NADH}$.

E_1

Implementation Name: `E_1`
Units: mV
Initial value: $E_{1,NADH}$
The energy provided by electron transfer to $Cu_{A,r}$.

$E_{1,n}$

Implementation Name: `E_1n`
Units: mV
Initial value: $E_{1,NADH,n}$
Normal value of E_1.

E_2

Implementation Name: `E_2`
Units: mV
Initial value: $\mathcal{E}_0(\mathrm{a}_3) - \mathcal{E}_0(\mathrm{Cu}_A)$
Energy provided by the transfer of four electrons from $Cu_{A,r}$ to $a_{3,o}$.

$\mathcal{E}_0(\mathrm{a}_3)$

Implementation Name: `E_a30`
Units: mV
Initial value: 350
Standard redox potential for cytochrome a_3.

$\mathcal{E}_0(\mathrm{Cu}_A)$

Implementation Name: `E_c0`
Units: mV
Initial value: 247
Standard redox potential for Cu_A.

$\mathcal{E}_0(\mathrm{NADH})$

Implementation Name: `E_N0`
Units: mV
Initial value: -320
Standard redox potential for NADH.

f_n

Implementation Name: `f_n`
Units: mM s^{-1}
Initial value: $\frac{CMRO_{2,n}}{Vol_{mit}}$
Normal resting value of f_1 and f_2.

G_n

Implementation Name: `Gn`
Units: $ml_{blood}\ ml_{brain}^{-1}\ mmHg^{-1}\ s^{-1}$
Initial value: $\frac{CBF_n}{P_{a,n} - P_{v,n}}$
Normal blood vessel conductance.

H_n^+

Implementation Name: `H_n`
Units: mM
Initial value: $10^{3-pH_{m,n}}$
Normal mitochondrial proton concentration.

$J_{O_{2,n}}$

Implementation Name: `J_O2n`
Units: mM s^{-1}
Initial value: $CMRO_{2,n}$
Normal oxygen flux from blood to tissue.

$k_{1,0}$
Implementation Name: `k10`
Units: s^{-1}
Initial value: $\frac{k_{1,n}\, NADH}{NADH_n}$
Forward reaction rate for the reduction of Cu_A at normal Δp.

$k_{1,n}$
Implementation Name: `k1_n`
Units: s^{-1}
Initial value: $\frac{f_n}{Cu_{A,o,n} - \frac{1}{K_{eq_1,n}}\, Cu_{A,r,n}}$
Forward reaction rate for the reduction of Cu_A at normal Δp and NADH.

$k_{2,n}$
Implementation Name: `k2_n`
Units: s^{-1}
Initial value: $\frac{f_n}{Cu_{A,r,n}\, a_{3,o,n} - \frac{1}{K_{eq_2,n}}\, Cu_{A,o,n}\, a_{3,r,n}}$
Normal forward reaction rate for the reduction of a_3.

$k_3 = \frac{k_{3,0}}{\frac{\exp(-c_3 - \Delta p_{30})}{1+\exp(-c_3 - \Delta p_{30})}}$
Implementation Name: `k3`
Units: s^{-1}
Initial value: 0
Forward reaction rate for the reduction of O_2.

$k_{3,0}$
Implementation Name: `k30`
Units: s^{-1}
Initial value: $2.5E+5$
Apparent second order rate constant for reduction of O_2 at zero Δp.

k_{aut}
Implementation Name: `k_aut`
Units: dimensionless
Initial value: 1
Overall functioning of autoregulatory response.

K_G
Implementation Name: `K_G`
Units: $ml_{blood}\ ml_{brain}^{-1}\ mmHg^{-1}\ s^{-1}\ cm^{-4}$
Initial value: $\frac{G_n}{\text{pow}\,(r_n, 4)}$
Proportionality constant in Poiseuille relation for conductance.

k_{lk2}
Implementation Name: `k_lk2`
Units: mV^{-1}
Initial value: 0.038
Constant controlling the depending of the leak rate L_{lk} on Δp.

k_{unc}
Implementation Name: `k_unc`
Units: dimensionless
Initial value: 1
Control parameter simulating the effect of adding uncouplers to the system.

$k_{CV} = \dfrac{-1}{\Delta p_n - \Delta p_{CV,0}} \log \left(\dfrac{1 - L_{CV,0}}{1 + r_{CV}\, L_{CV,0}} \right)$
Implementation Name: `kCV`
Units: mV^{-1}
Initial value: 0
Parameter controlling the sensitivity of Complex V flux to driving force.

$K_{eq_1,n}$
Implementation Name: `Keq1_n`
Units: dimensionless
Initial value: $10^{\frac{-1}{Z}\left(\frac{p_1\, \Delta p_n}{4} - E_{1,n}\right)}$
Normal value of the equilibrium constant for the Cu_A reduction reaction.

$K_{eq_2,n}$
Implementation Name: `Keq2_n`
Units: dimensionless
Initial value: $10^{\frac{-1}{Z}\left(\frac{p_3\, \Delta p_n}{4} - E_2\right)}$
Normal value of the equilibrium constant for the a_3 reduction reaction.

$L_{CV,0}$
Implementation Name: `L_CV0`
Units: dimensionless
Initial value: 0.4
Normal Complex V flux as a fraction of maximum possible flux.

$L_{CV,frac} = 1 - L_{lk,frac}$
Implementation Name: `L_CVfrac`
Units: dimensionless
Initial value: 0
Normal fraction of proton entry into mitochondria which is due to ADP phosphorylation.

$L_{CV,max} = \dfrac{L_{CV,n}}{L_{CV,0}}$
Implementation Name: `L_CVmax`
Units: $mM\ s^{-1}$
Initial value: 0
The maximum rate of proton flow through Complex V.

$L_{CV,n}$
Implementation Name: `L_CVn`
Units: $mM\ s^{-1}$
Initial value: $L_n\, L_{CV,frac}$
The resting flow of protons into the matrix through Complex V.

L_{lk0}
Implementation Name: `L_lk0`
Units: $mM\ s^{-1}$
Initial value: $\dfrac{L_{lk,n}}{\exp\left(\Delta p_n\, k_{lk2}\right) - 1}$
Constant controlling the depending of the leak rate L_{lk} on Δp.

$L_{lk,frac}$
Implementation Name: `L_lkfrac`
Units: dimensionless
Initial value: 0.25
Normal fraction of proton entry into mitochondria which is via leak channels.

$L_{lk,n}$
Implementation Name: `L_lkn`
Units: $mM\ s^{-1}$

Initial value: $L_n \, L_{lk,frac}$
The resting flow of protons into the matrix via leak channels.

$L_n \; = p_{tot} \, f_n$
Implementation Name: L_n
Units: mM s^{-1}
Initial value: 0
The normal total flow of protons back into mitochondria.

λ_0
Implementation Name: lam_0
Units: cm
Initial value: 0.01856
Intercept of the fitted linear model for blood vessel radius.

λ_μ
Implementation Name: lam_mu
Units: cm
Initial value: -0.003935
Fitted linear dependence of blood vessel radius on autoregulatory stimuli.

n_h
Implementation Name: n_h
Units: dimensionless
Initial value: 2.5
Hill coefficient for oxygen dissociation from haemoglobin.

$NADH \; = \dfrac{NAD_{pool}}{1 + \frac{NAD}{NADH}}$
Implementation Name: NADH
Units: mM
Initial value: 0
Concentration of NADH in the mitochondria.

$NADH_n$
Implementation Name: NADHn
Units: mM
Initial value: $\dfrac{NAD_{pool}}{1 + \frac{NAD_n}{NADH_n}}$
Normal concentration of NADH in the mitochondria.

$\frac{NAD}{NADH}$
Implementation Name: NADNADHrat
Units: dimensionless
Initial value: $\dfrac{\frac{NAD_n}{NADH_n}}{\mathrm{pow}\left(u, 2D_{NADH}\right)}$
NAD/NADH ratio.

$\frac{NAD_n}{NADH_n}$
Implementation Name: NADNADHratn
Units: dimensionless
Initial value: 9
Normal NAD/NADH ratio.

NAD_{pool}
Implementation Name: NADpool
Units: dimensionless
Initial value: 3
Relative size of the NAD pool, used to estimate normal mitochondrial NADH.

$O_{2,n}$
Implementation Name: `O2_n`
Units: mM
Initial value: 0.024
Normal mitochondrial oxygen concentration.

$O_{2,c,n}$
Implementation Name: `O2c_n`
Units: mM
Initial value: $\phi \, \mathrm{pow}\left(\frac{S_{c,O_2,n}}{1 - S_{c,O_2,n}}, \frac{1}{n_h}\right)$
Normal capillary oxygen concentration.

$p_1 = p_{tot} - p_{23}$
Implementation Name: `p1`
Units: dimensionless
Initial value: 0
Proton cost of the reaction reducing Cu_A.

p_3
Implementation Name: `p2`
Units: dimensionless
Initial value: 4
Proton cost of the reaction reducing a_3.

p_{23}
Implementation Name: `p23`
Units: dimensionless
Initial value: 8
Total protons removed from the mitochondrial matrix by the reductions of a_3 and O_2.

p_3
Implementation Name: `p3`
Units: dimensionless
Initial value: $p_{23} - p_3$
Proton cost of the reaction reducing O_2.

P_a
Implementation Name: `P_a`
Units: mmHg
Initial value: $P_{a,n}$
Mean arterial blood pressure.

$P_{a,n}$
Implementation Name: `P_an`
Units: mmHg
Initial value: 100
Normal arterial blood pressure.

p_{C1}
Implementation Name: `p_C1`
Units: dimensionless
Initial value: 8
Protons pumped by Complex I.

p_{C3}
Implementation Name: `p_C3`
Units: dimensionless
Initial value: 4
Protons pumped by Complex III.

p_{tot}
Implementation Name: p_tot
Units: dimensionless
Initial value: $p_{tot,NADH}$
Total protons removed from the mitochondrial matrix by the three modelled electron transport reactions.

$p_{tot,NADH} = p_{C1} + p_{C3} + p_{23}$
Implementation Name: p_totNADH
Units: dimensionless
Initial value: 0
Total protons pumped when the reducing agent is NADH.

P_v
Implementation Name: P_v
Units: mmHg
Initial value: $P_{v,n}$
Venous blood pressure.

$P_{v,n}$
Implementation Name: P_vn
Units: mmHg
Initial value: 4
Normal venous blood pressure.

Pa_{CO_2}
Implementation Name: Pa_CO2
Units: mmHg
Initial value: $Pa_{CO_{2,n}}$
Arterial partial pressure of carbon dioxide.

$Pa_{CO_{2,n}}$
Implementation Name: Pa_CO2n
Units: mmHg
Initial value: 40
Normal arterial partial pressure of carbon dioxide.

$pH_{m,n}$
Implementation Name: pH_mn
Units: dimensionless
Initial value: 7.4
Normal mitochondrial pH.

pH_o
Implementation Name: pH_o
Units: dimensionless
Initial value: 7
Extra-mitochondrial pH.

$pH_{o,n}$
Implementation Name: pH_on
Units: dimensionless
Initial value: 7
Normal extra-mitochondrial pH.

ϕ
Implementation Name: phi
Units: mM
Initial value: 0.036
Oxygen concentration at half-maximal saturation.

R_{CO_2}
Implementation Name: R_autc
Units: dimensionless
Initial value: 2.2
Autoregulatory reactivity to carbon dioxide.

R_{O_2}
Implementation Name: R_auto
Units: dimensionless
Initial value: 1.5
Autoregulatory reactivity to oxygen.

R_{P_a}
Implementation Name: R_autp
Units: dimensionless
Initial value: 4
Autoregulatory reactivity to blood pressure.

R_u
Implementation Name: R_autu
Units: dimensionless
Initial value: 0.5
Autoregulatory reactivity to demand.

r_{CV}
Implementation Name: r_CV
Units: dimensionless
Initial value: 5
Parameter controlling the ratio of maximal to minimal rates of oxidative phosphorylation.

r_n
Implementation Name: r_n
Units: cm
Initial value: 0.0187
Normal blood vessel radius. Normal effective blood vessel radius.

$S_{a,O_2,n}$
Implementation Name: SaO2_n
Units: dimensionless
Initial value: 0.96
Normal arterial oxygen saturation.

S_{a,O_2}
Implementation Name: SaO2sup
Units: dimensionless
Initial value: $S_{a,O_2,n}$
Arterial oxygen saturation.

$S_{c,O_2,n}$
Implementation Name: ScO2_n
Units: dimensionless
Initial value: $\frac{S_{a,O_2,n} + S_{v,O_2,n}}{2}$
Normal capillary oxygen saturation.

$S_{v,O_2,n}$
Implementation Name: SvO2_n
Units: dimensionless
Initial value: $\frac{HbO_{2,v,n}}{Hb_{tot,n}}$
Normal venous oxygen saturation.

t
Implementation Name: `t`
Units: s
Initial value: 0
Time over which the system evolves.

τ_{CO_2}
Implementation Name: `t_c`
Units: s
Initial value: 5
Filter time constant for stimulus effect of carbon dioxide.

τ_{O_2}
Implementation Name: `t_o`
Units: s
Initial value: 20
Filter time constant for stimulus effect of capillary oxygen.

τ_{P_a}
Implementation Name: `t_p`
Units: s
Initial value: 5
Filter time constant for stimulus effect of blood pressure.

τ_u
Implementation Name: `t_u`
Units: s
Initial value: 0.5
Filter time constant for stimulus effect of demand.

u
Implementation Name: `u`
Units: dimensionless
Initial value: u_n
Parameter indicating metabolic demand.

u_n
Implementation Name: `u_n`
Units: dimensionless
Initial value: 1
Normal demand.

$v_{CO_2,n}$
Implementation Name: `v_cn`
Units: mmHg
Initial value: $Pa_{CO_{2,n}}$
Normal filtered carbon dioxide partial pressure. Normal filtered carbon dioxide partial pressure.

$v_{O_2,n}$
Implementation Name: `v_on`
Units: mM
Initial value: $O_{2,c,n}$
Normal filtered capillary oxygen concentration. Normal filtered capillary oxygen concentration.

$v_{P_a,n}$
Implementation Name: `v_pn`
Units: mmHg
Initial value: $P_{a,n}$

Normal filtered arterial blood pressure. Normal filtered blood pressure.

$v_{u,n}$
Implementation Name: `v_un`
Units: dimensionless
Initial value: u_n
Normal filtered demand. Normal filtered demand.

$VArat_n$
Implementation Name: `VArat_n`
Units: dimensionless
Initial value: 3
Normal volume ratio of veins to arteries in brain tissue.

$V_{a,n}$
Implementation Name: `Vol_artn`
Units: dimensionless
Initial value: $\frac{1}{1 + VArat_n}$
Normal relative arterial blood volume.

Vol_{mit}
Implementation Name: `Vol_mit`
Units: dimensionless
Initial value: 0.067
Fraction of brain tissue volume that is mitochondria.

V_v
Implementation Name: `Vol_ven`
Units: dimensionless
Initial value: $\frac{VArat_n}{1 + VArat_n}$
Relative venous blood volume.

$HbO_{2,a} = Hb_{tot}\, S_{a,O_2}$
Implementation Name: `XOa`
Units: mM
Initial value: $HbO_{2,a,n}$
Arterial concentration of oxygen bound to haemoglobin.

$HbO_{2,a,n}$
Implementation Name: `XOa_n`
Units: mM
Initial value: $Hb_{tot,n}\, S_{a,O_2,n}$
Normal arterial concentration of oxygen bound to haemoglobin.

$HbO_{2,v,n}$
Implementation Name: `XOv_n`
Units: mM
Initial value: $\frac{CBF_n\, HbO_{2,a,n} - J_{O_{2,n}}}{CBF_n}$
Normal venous concentration of oxygen bound to haemoglobin.

Hb_{tot}
Implementation Name: `Xtot`
Units: mM
Initial value: 9.1
Total concentration of haemoglobin O_2 binding sites in blood (4 times haemoglobin concentration).

$Hb_{tot,n}$
Implementation Name: `Xtot_n`

Units: mM
Initial value: 9.1
Normal total concentration of haemoglobin O_2 binding sites in blood (4 times haemoglobin concentration).

Z

Implementation Name: `Z`
Units: mV
Initial value: 59.028
Proportionality constant in calculation of driving forces due to concentration differences. Defined as RT/F, where F is Faraday's constant, R the ideal gas constant and T the absolute temperature.

5 BSB4

5.1 Overview

Simplified model in which the blood flow submodel is replaced with variant B4.

- 9 differential state variables
- 3 algebraic state variables
- 35 intermediate variables
- 120 parameters
- 4 declared inputs
- 33 default outputs

5.2 Differential Equations

$$\frac{\mathrm{d}Cu_{A,o}}{\mathrm{d}t} = 4f_3 - 4f_1 \tag{5.1}$$

$$\frac{\mathrm{d}a_{3,r}}{\mathrm{d}t} = 4f_3 - 4f_3 \tag{5.2}$$

$$\frac{\mathrm{d}\psi}{\mathrm{d}t} = \frac{p_3\,f_3 + p_1\,f_1 + p_3\,f_3 - L}{C_{im}} \tag{5.3}$$

$$\frac{\mathrm{d}H^+}{\mathrm{d}t} = \frac{1}{R_{Hi}}\,L - \frac{p_3}{R_{Hi}}\,f_3 - \frac{p_1}{R_{Hi}}\,f_1 - \frac{p_3}{R_{Hi}}\,f_3 \tag{5.4}$$

$$\frac{\mathrm{d}O_2}{\mathrm{d}t} = \frac{1}{Vol_{mit}}\,J_{O_2} - f_3 \tag{5.5}$$

$$\frac{\mathrm{d}\nu_{CO_2}}{\mathrm{d}t} = \frac{1}{\tau_{CO_2}}\,\left(Pa_{CO_2} - \nu_{CO_2}\right) \tag{5.6}$$

$$\frac{\mathrm{d}\nu_{O_2}}{\mathrm{d}t} = \frac{1}{\tau_{O_2}}\,\left(O_{2,c} - \nu_{O_2}\right) \tag{5.7}$$

$$\frac{\mathrm{d}\nu_{P_a}}{\mathrm{d}t} = \frac{1}{\tau_{P_a}}\,\left(P_a - \nu_{P_a}\right) \tag{5.8}$$

$$\frac{d\nu_u}{dt} = \frac{1}{\tau_u}\,(u - \nu_u) \tag{5.9}$$

5.3 Algebraic Equations

$$\phi\left(\frac{S_{c,O_2}}{1 - S_{c,O_2}}\right)^{\frac{1}{n_h}} - O_{2,c} = 0 \tag{5.10}$$

$$\lambda_0 + \frac{\lambda_{P_a}}{P_a} - r = 0 \tag{5.11}$$

$$CBF\,(HbO_{2,a} - HbO_{2,v}) - J_{O_2} = 0 \tag{5.12}$$

5.4 Chemical Reactions

$$\xrightarrow{L} \frac{1}{R_{Hi}}\,\mathrm{H}^+ \tag{5.13}$$

$$\xrightarrow{J_{O_2}} \frac{1}{Vol_{mit}}\,\mathrm{O}_2 \tag{5.14}$$

$$\frac{p_3}{R_{Hi}}\,\mathrm{H}^+ \xrightarrow{f_3} 4\,\mathrm{Cu}_{\mathrm{A,o}} + 4\,\mathrm{a}_{3,\mathrm{r}} \tag{5.15}$$

$$4\,\mathrm{Cu}_{\mathrm{A,o}} + \frac{p_1}{R_{Hi}}\,\mathrm{H}^+ \xrightarrow{f_1} \tag{5.16}$$

$$\mathrm{O}_2 + 4\,\mathrm{a}_{3,\mathrm{r}} + \frac{p_3}{R_{Hi}}\,\mathrm{H}^+ \xrightarrow{f_3} \tag{5.17}$$

5.5 State Variables

$Cu_{A,o}$

Implementation Name: `a`
Units: mM
Initial value: $Cu_{A,o,n}$
Concentration of oxidised cytochrome c oxidase.

$a_{3,r}$

Implementation Name: `bred`
Units: mM
Initial value: $a_{3,r,n}$
Concentration of reduced cytochrome a_3.

ψ
Implementation Name: `Dpsi`
Units: mV
Initial value: ψ_n
Mitochondrial inner membrane potential. Varies as charge (in the form of protons) is transferred across the membrane capacitance.

H^+
Implementation Name: `H`
Units: mM
Initial value: H_n^+
Mitochondrial proton concentration.

O_2
Implementation Name: `O2`
Units: mM
Initial value: $O_{2,n}$
Mitochondrial oxygen concentration.

$O_{2,c}$
Implementation Name: `O2c`
Units: mM
Initial value: $O_{2,c,n}$
Capillary oxygen concentration.

r
Implementation Name: `r`
Units: cm
Initial value: r_n
Typical blood vessel radius.

ν_{CO_2}
Implementation Name: `v_c`
Units: mmHg
Initial value: $\nu_{CO_2,n}$
Filtered carbon dioxide partial pressure.

ν_{O_2}
Implementation Name: `v_o`
Units: mM
Initial value: $\nu_{O_2,n}$
Filtered capillary oxygen concentration.

ν_{P_a}
Implementation Name: `v_p`
Units: mmHg
Initial value: $\nu_{P_a,n}$
Filtered arterial blood pressure.

ν_u
Implementation Name: `v_u`
Units: dimensionless
Initial value: $\nu_{u,n}$
Filtered demand.

$HbO_{2,v}$
Implementation Name: `XOv`
Units: mM
Initial value: $HbO_{2,v,n}$
Venous concentration of oxygen bound to haemoglobin.

5.6 Intermediate Variables

$Cu_{A,r} = CCO_{tot} - Cu_{A,o}$
Implementation Name: `ared`
Units: mM
Initial value: 0
Concentration of reduced Cu_A.

$a_{3,o} = CCO_{tot} - a_{3,r}$
Implementation Name: `b`
Units: mM
Initial value: 0
Concentration of oxidised cytochrome a_3.

$$C_{0,i} = \frac{10^{-pH_m} - 10^{-pH_m - dpH}}{dpH}$$
Implementation Name: `C_0i`
Units: dimensionless
Initial value: 0
Natural buffering capacity of protons in mitochondria.

$CBF = G\ (P_a - P_v)$
Implementation Name: `CBF`
Units: $ml_{blood}\ ml_{brain}^{-1}\ s^{-1}$
Initial value: CBF_n
Cerebral blood flow.

$\Delta oxCCO = \Delta oxCCO_{off} + 1000 Vol_{mit}\ (Cu_{A,o} - Cu_{A,o,n})$
Implementation Name: `CCO`
Units: uM
Initial value: 0
Cytochrome c oxidase signal measured by NIRS.

$CMRO_2 = f_3\, Vol_{mit}$
Implementation Name: `CMRO2`
Units: mM s^{-1}
Initial value: 0
Rate of cerebral oxygen metabolism.

$\Delta p = \psi + Z\ (pH_m - pH_o)$
Implementation Name: `Dp`
Units: mV
Initial value: 0
Proton motive force across the mitochondrial inner membrane.

$$\eta = R_{P_a}\left(\frac{v_{P_a}}{v_{P_a,n}} - 1\right) + R_{O_2}\left(\frac{v_{O_2}}{v_{O_2,n}} - 1\right) + R_{CO_2}\left(1 - \frac{v_{CO_2}}{v_{CO_2,n}}\right) + R_u\left(1 - \frac{v_u}{v_{u,n}}\right)$$
Implementation Name: `eta`
Units: dimensionless
Initial value: 0
Merged autoregulation stimulus.

$f_1 = k_1\, Cu_{A,o} - k_{-1}\, Cu_{A,r}$
Implementation Name: `f1`
Units: mM s^{-1}
Initial value: 0
Reaction rate for the reduction of Cu_A.

$f_3 = k_2\, Cu_{A,r}\, a_{3,o} - k_{-2}\, Cu_{A,o}\, a_{3,r}$
Implementation Name: `f2`

Units: mM s^{-1}
Initial value: 0
Reaction rate for the reduction of a_3.

$f_3 = \frac{k_3\, O_2\, a_{3,r} \exp\left(-c_3\, (\Delta p - \Delta p_{30})\right)}{1 + \exp\left(-c_3\, (\Delta p - \Delta p_{30})\right)}$
Implementation Name: `f3`
Units: mM s^{-1}
Initial value: 0
Reaction rate for the reduction of O_2.

$G = K_G\, r^4$
Implementation Name: `G`
Units: $ml_{blood}\ ml_{brain}^{-1}\ mmHg^{-1}\ s^{-1}$
Initial value: 0
Effective conductance of the whole blood flow compartment.

$HbO_2 = (V_a\, HbO_{2,a} + V_v\, HbO_{2,v})\ blood_{hb}$
Implementation Name: `HbO2`
Units: uM
Initial value: 0
Oxygenated haemoglobin signal measured by NIRS.

$HbT = (V_a + V_v)\ Hb_{tot}\, blood_{hb}$
Implementation Name: `HbT`
Units: uM
Initial value: 0
Total haemoglobin signal measured by NIRS.

$HHb = HbT - HbO_2$
Implementation Name: `HHb`
Units: uM
Initial value: 0
Deoxygenated haemoglobin signal measured by NIRS.

$J_{O_2} = \text{fmin}\left(D_{O_2}\ (O_{2,c} - O_2)\,, CBF\, HbO_{2,a}\right)$
Implementation Name: `J_O2`
Units: mM s^{-1}
Initial value: 0
Oxygen flux from blood to tissue.

$k_1 = k_{1,0} \exp\left(-c_{k_1}\ (\Delta p - \Delta p_n)\right)$
Implementation Name: `k1`
Units: s^{-1}
Initial value: 0
Forward reaction rate for the reduction of Cu_A.

$k_2 = k_{2,n} \exp\left(-c_{k_2}\ (\Delta p - \Delta p_n)\right)$
Implementation Name: `k2`
Units: s^{-1}
Initial value: 0
Forward reaction rate for the reduction of a_3.

$K_{eq_1} = 10^{\frac{-1}{Z}\left(\frac{p_1 \Delta p}{4} - E_1\right)}$
Implementation Name: `Keq1`
Units: dimensionless
Initial value: 0
Equilibrium constant for the Cu_A reduction reaction.

$K_{eq_2} = 10^{\frac{-1}{Z}\left(\frac{p_3 \Delta p}{4} - E_2\right)}$

Implementation Name: Keq2
Units: dimensionless
Initial value: 0
Equilibrium constant for the a_3 reduction reaction.

$$k_{-1} = \frac{k_1}{K_{eq_1}}$$
Implementation Name: kn1
Units: s^{-1}
Initial value: 0
Reverse reaction rate for the reduction of Cu_A.

$$k_{-2} = \frac{k_2}{K_{eq_2}}$$
Implementation Name: kn2
Units: s^{-1}
Initial value: 0
Reverse reaction rate for the reduction of a_3.

$$L = L_{CV} + L_{lk}$$
Implementation Name: L
Units: mM s^{-1}
Initial value: 0
Rate of proton return to the mitochondrial matrix.

$$L_{CV} = \frac{CV_{inh}\, L_{CV,max}\, (1 - \exp(-\theta))}{1 + r_{CV} \exp(-\theta)}$$
Implementation Name: L_CV
Units: mM s^{-1}
Initial value: 0
Rate at which protons re-enter the mitochondrial matrix due to ADP phosphorylation.

$$L_{lk} = k_{unc}\, L_{lk0}\, (\exp(\Delta p\, k_{lk2}) - 1)$$
Implementation Name: L_lk
Units: mM s^{-1}
Initial value: 0
Rate at which protons re-enter the mitochondrial matrix via leak channels.

$$\mu = \frac{k_{aut}\, (\exp(\eta) - 1)}{\exp(\eta) + 1}$$
Implementation Name: mu
Units: dimensionless
Initial value: 0
Effective strength of the autoregulation reponse.

$$pH_m = -\log 10 \left(\frac{H^+}{1000} \right)$$
Implementation Name: pH_m
Units: dimensionless
Initial value: 0
Mitochondrial pH.

$$r_{buffi} = \frac{C_{buffi}}{C_{0,i}}$$
Implementation Name: r_buffi
Units: dimensionless
Initial value: 0
Buffering capacity for protons in mitochondria.

$$R_{Hi} = r_{buffi}$$

Implementation Name: `R_Hi`
Units: dimensionless
Initial value: 0
Relative mitochondrial volume for protons, taking into account buffering effect of pH.

$S_{c,O_2} = \dfrac{S_{a,O_2} + S_{v,O_2}}{2}$
Implementation Name: `ScO2`
Units: dimensionless
Initial value: $S_{c,O_2,n}$
Capillary oxygen saturation.

$S_{v,O_2} = \dfrac{HbO_{2,v}}{Hb_{tot}}$
Implementation Name: `SvO2`
Units: dimensionless
Initial value: $S_{v,O_2,n}$
Venous oxygen saturation.

$\theta = k_{CV}\ (\Delta p + Z \log 10\,(u) - \Delta p_{CV,0})$
Implementation Name: `theta`
Units: dimensionless
Initial value: 0
Driving force Complex V.

$TOI = \dfrac{100 HbO_2}{HbT}$
Implementation Name: `TOI`
Units: dimensionless
Initial value: 0
Total oxygenation index.

$V_{mca} = CBF\, CBFscale$
Implementation Name: `Vmca`
Units: cm s^{-1}
Initial value: 0
Blood velocity in the middle cerebral artery.

$V_a = V_{a,n} \left(\dfrac{r}{r_n}\right)^2$
Implementation Name: `Vol_art`
Units: dimensionless
Initial value: 0
Relative arterial blood volume.

5.7 Parameters

$Cu_{a,frac,n}$
Implementation Name: `a_frac_n`
Units: dimensionless
Initial value: 0.8
Normal oxidised fraction of Cu$_A$.

$Cu_{A,o,n}$
Implementation Name: `a_n`
Units: mM
Initial value: $CCO_{tot}\, Cu_{a,frac,n}$
Normal concentration of oxidised cytochrome c oxidase.

$Cu_{A,r,n}$
Implementation Name: ared_n
Units: mM
Initial value: $CCO_{tot} - Cu_{A,o,n}$
Normal concentration of reduced Cu_A.

$a_{3,o,n}$
Implementation Name: b_n
Units: mM
Initial value: $CCO_{tot} - a_{3,r,n}$
Normal concentration of oxidised cytochrome a_3.

$blood_{hb}$
Implementation Name: blood_hb
Units: dimensionless
Initial value: 10.00
Factor to convert model haemoglobin concentration to instrumental units. Scales for blood fraction of brain volume, mM to μM, and number of binding sites.

$a_{3,r,n}$
Implementation Name: bred_n
Units: mM
Initial value: $\frac{\frac{\frac{f_n}{k_3}}{O_{2,n}}}{\frac{\exp(-c_3\,(\Delta p_n - \Delta p_{30}))}{1+\exp(-c_3\,(\Delta p_n - \Delta p_{30}))}}$
Normal concentration of reduced cytochrome a3.

c_3
Implementation Name: c3
Units: mV^{-1}
Initial value: 0.11
Parameter controlling the sensitivity of the reduction of a_3 to Δp.

C_{buffi}
Implementation Name: C_buffi
Units: dimensionless
Initial value: 0.022
Buffering capacity of protons in mitochondria.

C_{im}
Implementation Name: C_im
Units: mM mV^{-1}
Initial value: 0.00675
Capacitance of the mitochondrial inner membrane.

$C_{NADH} = \frac{Z}{2}\log 10\left(\frac{1}{\frac{NAD}{NADH}}\right)$
Implementation Name: C_NADH
Units: mV
Initial value: 0
Excess redox potential for NADH at normal demand.

$C_{NADH,n}$
Implementation Name: C_NADH_n
Units: mV
Initial value: $\frac{Z}{2}\log 10\left(\frac{1}{\frac{NAD_n}{NADH_n}}\right)$
Normal value of C_{NADH}.

CBF_n
Implementation Name: `CBFn`
Units: $ml_{blood}\ ml_{brain}^{-1}\ s^{-1}$
Initial value: 0.0125
Normal cerebral blood flow.

$CBFscale$
Implementation Name: `CBFscale`
Units: cm
Initial value: 5000
Scale constant relating blood flow to arterial velocity.

$\Delta oxCCO_{off}$
Implementation Name: `CCO_offset`
Units: uM
Initial value: 0
Signal offset for the NIRS CCO measurement.

c_{k_1}
Implementation Name: `ck1`
Units: mV^{-1}
Initial value: 0.01
Parameter controlling sensitivity of k_1 to Δp.

c_{k_2}
Implementation Name: `ck2`
Units: mV^{-1}
Initial value: 0.02
Parameter controlling sensitivity of k_2 to Δp.

$CMRO_{2,n}$
Implementation Name: `CMRO2_n`
Units: $mM\ s^{-1}$
Initial value: 0.034
Normal metabolic rate of oxygen consumption.

CV_{inh}
Implementation Name: `CVinh`
Units: dimensionless
Initial value: 1
Control parameter representing the action of Complex V inhibitors.

$CCO_{tot} = \dfrac{CCO_{tis}}{Vol_{mit}}$
Implementation Name: `cytox_tot`
Units: mM
Initial value: 0
Concentration of cytochrome c oxidase in mitochondria.

CCO_{tis}
Implementation Name: `cytox_tot_tis`
Units: mM
Initial value: 0.0055
Concentration of cytochrome c oxidase in tissue.

D_{NADH}
Implementation Name: `D_NADH`
Units: dimensionless
Initial value: 0.01
Scale parameter for the dependence of NADH redox potential on demand.

D_{O_2}
Implementation Name: `D_O2`
Units: s^{-1}
Initial value: $\frac{J_{O_{2,n}}}{O_{2,c,n} - O_{2,n}}$
Diffusion rate for oxygen between capillaries and mitochondria.

$\Delta p_{3,corr}$
Implementation Name: `Dp3_corr`
Units: mV
Initial value: -25
Difference between Δp_{30} and normal Δp.

$\Delta p_{30} = \Delta p_n + \Delta p_{3,corr}$
Implementation Name: `Dp_30`
Units: mV
Initial value: 0
Value of Δp to which a_3 reduction reaction is maximally sensitive.

$\Delta p_{CV,0}$
Implementation Name: `Dp_CV0`
Units: mV
Initial value: 90
Value of Δp at which L_{CV} is zero under normal demand.

Δp_n
Implementation Name: `Dp_n`
Units: mV
Initial value: $\psi_n + Z\,\Delta pH_n$
Normal value of Δp

dpH
Implementation Name: `dpH`
Units: dimensionless
Initial value: 0.001
Parameter in the mitochondrial proton buffering relationship.

ΔpH_n
Implementation Name: `DpH_n`
Units: dimensionless
Initial value: $pH_{m,n} - pH_{o,n}$
Normal pH difference across the mitochondrial inner membrane.

ψ_n
Implementation Name: `Dpsi_n`
Units: mV
Initial value: 145
Normal mitochondrial inner membrane potential.

$E_{1,NADH} = \mathcal{E}_0(\mathrm{Cu}_A) - \mathcal{E}_0(\mathrm{NADH}) + C_{NADH}$
Implementation Name: `E1NADH`
Units: mV
Initial value: 0
Value of E_1 when the reducing substrate is NADH.

$E_{1,NADH,n}$
Implementation Name: `E1NADH_n`
Units: mV
Initial value: $\mathcal{E}_0(\mathrm{Cu}_A) - \mathcal{E}_0(\mathrm{NADH}) + C_{NADH,n}$
Normal value of $E_{1,NADH}$.

E_1

Implementation Name: `E_1`
Units: mV
Initial value: $E_{1,NADH}$
The energy provided by electron transfer to $Cu_{A,r}$.

$E_{1,n}$

Implementation Name: `E_1n`
Units: mV
Initial value: $E_{1,NADH,n}$
Normal value of E_1.

E_2

Implementation Name: `E_2`
Units: mV
Initial value: $\mathcal{E}_0(a_3) - \mathcal{E}_0(Cu_A)$
Energy provided by the transfer of four electrons from $Cu_{A,r}$ to $a_{3,o}$.

$\mathcal{E}_0(a_3)$

Implementation Name: `E_a30`
Units: mV
Initial value: 350
Standard redox potential for cytochrome a_3.

$\mathcal{E}_0(Cu_A)$

Implementation Name: `E_c0`
Units: mV
Initial value: 247
Standard redox potential for Cu_A.

$\mathcal{E}_0$(NADH)

Implementation Name: `E_N0`
Units: mV
Initial value: -320
Standard redox potential for NADH.

f_n

Implementation Name: `f_n`
Units: mM s^{-1}
Initial value: $\frac{CMRO_{2,n}}{Vol_{mit}}$
Normal resting value of f_1 and f_2.

G_n

Implementation Name: `Gn`
Units: $ml_{blood}\ ml_{brain}^{-1}\ mmHg^{-1}\ s^{-1}$
Initial value: $\frac{CBF_n}{P_{a,n} - P_{v,n}}$
Normal blood vessel conductance.

H_n^+

Implementation Name: `H_n`
Units: mM
Initial value: $10^{3-pH_{m,n}}$
Normal mitochondrial proton concentration.

$J_{O_{2,n}}$

Implementation Name: `J_O2n`
Units: mM s^{-1}
Initial value: $CMRO_{2,n}$
Normal oxygen flux from blood to tissue.

$k_{1,0}$
Implementation Name: `k10`
Units: s^{-1}
Initial value: $\frac{k_{1,n}\,NADH}{NADH_n}$
Forward reaction rate for the reduction of Cu_A at normal Δp.

$k_{1,n}$
Implementation Name: `k1_n`
Units: s^{-1}
Initial value: $\frac{f_n}{Cu_{A,o,n} - \frac{1}{K_{eq_1,n}}\,Cu_{A,r,n}}$
Forward reaction rate for the reduction of Cu_A at normal Δp and NADH.

$k_{2,n}$
Implementation Name: `k2_n`
Units: s^{-1}
Initial value: $\frac{f_n}{Cu_{A,r,n}\,a_{3,o,n} - \frac{1}{K_{eq_2,n}}\,Cu_{A,o,n}\,a_{3,r,n}}$
Normal forward reaction rate for the reduction of a_3.

$k_3 = \frac{k_{3,0}}{\frac{\exp(-c_3 - \Delta p_{30})}{1+\exp(-c_3 - \Delta p_{30})}}$
Implementation Name: `k3`
Units: s^{-1}
Initial value: 0
Forward reaction rate for the reduction of O_2.

$k_{3,0}$
Implementation Name: `k30`
Units: s^{-1}
Initial value: $2.5E+5$
Apparent second order rate constant for reduction of O_2 at zero Δp.

k_{aut}
Implementation Name: `k_aut`
Units: dimensionless
Initial value: 1
Overall functioning of autoregulatory response.

K_G
Implementation Name: `K_G`
Units: $ml_{blood}\ ml_{brain}^{-1}\ mmHg^{-1}\ s^{-1}\ cm^{-4}$
Initial value: $\frac{G_n}{\mathrm{pow}\,(r_n, 4)}$
Proportionality constant in Poiseuille relation for conductance.

k_{lk2}
Implementation Name: `k_lk2`
Units: mV^{-1}
Initial value: 0.038
Constant controlling the depending of the leak rate L_{lk} on Δp.

k_{unc}
Implementation Name: `k_unc`
Units: dimensionless
Initial value: 1
Control parameter simulating the effect of adding uncouplers to the system.

$k_{CV} = \dfrac{-1}{\Delta p_n - \Delta p_{CV,0}} \log\left(\dfrac{1 - L_{CV,0}}{1 + r_{CV}\, L_{CV,0}}\right)$
Implementation Name: `kCV`
Units: mV^{-1}
Initial value: 0
Parameter controlling the sensitivity of Complex V flux to driving force.

$K_{eq_1,n}$
Implementation Name: `Keq1_n`
Units: dimensionless
Initial value: $10^{\frac{-1}{Z}\left(\frac{p_1\,\Delta p_n}{4} - E_{1,n}\right)}$
Normal value of the equilibrium constant for the Cu_A reduction reaction.

$K_{eq_2,n}$
Implementation Name: `Keq2_n`
Units: dimensionless
Initial value: $10^{\frac{-1}{Z}\left(\frac{p_3\,\Delta p_n}{4} - E_2\right)}$
Normal value of the equilibrium constant for the a_3 reduction reaction.

$L_{CV,0}$
Implementation Name: `L_CV0`
Units: dimensionless
Initial value: 0.4
Normal Complex V flux as a fraction of maximum possible flux.

$L_{CV,frac} = 1 - L_{lk,frac}$
Implementation Name: `L_CVfrac`
Units: dimensionless
Initial value: 0
Normal fraction of proton entry into mitochondria which is due to ADP phosphorylation.

$L_{CV,max} = \dfrac{L_{CV,n}}{L_{CV,0}}$
Implementation Name: `L_CVmax`
Units: $mM\ s^{-1}$
Initial value: 0
The maximum rate of proton flow through Complex V.

$L_{CV,n}$
Implementation Name: `L_CVn`
Units: $mM\ s^{-1}$
Initial value: $L_n\, L_{CV,frac}$
The resting flow of protons into the matrix through Complex V.

L_{lk0}
Implementation Name: `L_lk0`
Units: $mM\ s^{-1}$
Initial value: $\dfrac{L_{lk,n}}{\exp\left(\Delta p_n\, k_{lk2}\right) - 1}$
Constant controlling the depending of the leak rate L_{lk} on Δp.

$L_{lk,frac}$
Implementation Name: `L_lkfrac`
Units: dimensionless
Initial value: 0.25
Normal fraction of proton entry into mitochondria which is via leak channels.

$L_{lk,n}$
Implementation Name: `L_lkn`
Units: $mM\ s^{-1}$

Initial value: $L_n\,L_{lk,frac}$
The resting flow of protons into the matrix via leak channels.

$L_n\ = p_{tot}\,f_n$
Implementation Name: `L_n`
Units: mM s^{-1}
Initial value: 0
The normal total flow of protons back into mitochondria.

λ_0
Implementation Name: `lam_0`
Units: cm
Initial value: 0.01650
Intercept of the fitted linear model for blood vessel radius.

λ_{P_a}
Implementation Name: `lam_p`
Units: cm mmHg
Initial value: 0.2483
Fitted linear dependence of blood vessel radius on reciprocal of blood pressure.

n_h
Implementation Name: `n_h`
Units: dimensionless
Initial value: 2.5
Hill coefficient for oxygen dissociation from haemoglobin.

$NADH\ = \dfrac{NAD_{pool}}{1 + \frac{NAD}{NADH}}$
Implementation Name: `NADH`
Units: mM
Initial value: 0
Concentration of NADH in the mitochondria.

$NADH_n$
Implementation Name: `NADHn`
Units: mM
Initial value: $\dfrac{NAD_{pool}}{1 + \frac{NAD_n}{NADH_n}}$
Normal concentration of NADH in the mitochondria.

$\frac{NAD}{NADH}$
Implementation Name: `NADNADHrat`
Units: dimensionless
Initial value: $\dfrac{\frac{NAD_n}{NADH_n}}{\text{pow}\,(u, 2D_{NADH})}$
NAD/NADH ratio.

$\frac{NAD_n}{NADH_n}$
Implementation Name: `NADNADHratn`
Units: dimensionless
Initial value: 9
Normal NAD/NADH ratio.

NAD_{pool}
Implementation Name: `NADpool`
Units: dimensionless
Initial value: 3
Relative size of the NAD pool, used to estimate normal mitochondrial NADH.

$O_{2,n}$
Implementation Name: O2_n
Units: mM
Initial value: 0.024
Normal mitochondrial oxygen concentration.

$O_{2,c,n}$
Implementation Name: O2c_n
Units: mM
Initial value: $\phi \, \text{pow}\left(\frac{S_{c,O_2,n}}{1 - S_{c,O_2,n}}, \frac{1}{n_h}\right)$
Normal capillary oxygen concentration.

$p_1 \; = p_{tot} - p_{23}$
Implementation Name: p1
Units: dimensionless
Initial value: 0
Proton cost of the reaction reducing Cu_A.

p_3
Implementation Name: p2
Units: dimensionless
Initial value: 4
Proton cost of the reaction reducing a_3.

p_{23}
Implementation Name: p23
Units: dimensionless
Initial value: 8
Total protons removed from the mitochondrial matrix by the reductions of a_3 and O_2.

p_3
Implementation Name: p3
Units: dimensionless
Initial value: $p_{23} - p_3$
Proton cost of the reaction reducing O_2.

P_a
Implementation Name: P_a
Units: mmHg
Initial value: $P_{a,n}$
Mean arterial blood pressure.

$P_{a,n}$
Implementation Name: P_an
Units: mmHg
Initial value: 100
Normal arterial blood pressure.

p_{C1}
Implementation Name: p_C1
Units: dimensionless
Initial value: 8
Protons pumped by Complex I.

p_{C3}
Implementation Name: p_C3
Units: dimensionless
Initial value: 4
Protons pumped by Complex III.

p_{tot}
Implementation Name: p_tot
Units: dimensionless
Initial value: $p_{tot,NADH}$
Total protons removed from the mitochondrial matrix by the three modelled electron transport reactions.

$p_{tot,NADH} = p_{C1} + p_{C3} + p_{23}$
Implementation Name: p_totNADH
Units: dimensionless
Initial value: 0
Total protons pumped when the reducing agent is NADH.

P_v
Implementation Name: P_v
Units: mmHg
Initial value: $P_{v,n}$
Venous blood pressure.

$P_{v,n}$
Implementation Name: P_vn
Units: mmHg
Initial value: 4
Normal venous blood pressure.

Pa_{CO_2}
Implementation Name: Pa_CO2
Units: mmHg
Initial value: $Pa_{CO_{2,n}}$
Arterial partial pressure of carbon dioxide.

$Pa_{CO_{2,n}}$
Implementation Name: Pa_CO2n
Units: mmHg
Initial value: 40
Normal arterial partial pressure of carbon dioxide.

$pH_{m,n}$
Implementation Name: pH_mn
Units: dimensionless
Initial value: 7.4
Normal mitochondrial pH.

pH_o
Implementation Name: pH_o
Units: dimensionless
Initial value: 7
Extra-mitochondrial pH.

$pH_{o,n}$
Implementation Name: pH_on
Units: dimensionless
Initial value: 7
Normal extra-mitochondrial pH.

ϕ
Implementation Name: phi
Units: mM
Initial value: 0.036
Oxygen concentration at half-maximal saturation.

R_{CO_2}
Implementation Name: R_autc
Units: dimensionless
Initial value: 2.2
Autoregulatory reactivity to carbon dioxide.

R_{O_2}
Implementation Name: R_auto
Units: dimensionless
Initial value: 1.5
Autoregulatory reactivity to oxygen.

R_{P_a}
Implementation Name: R_autp
Units: dimensionless
Initial value: 4
Autoregulatory reactivity to blood pressure.

R_u
Implementation Name: R_autu
Units: dimensionless
Initial value: 0.5
Autoregulatory reactivity to demand.

r_{CV}
Implementation Name: r_CV
Units: dimensionless
Initial value: 5
Parameter controlling the ratio of maximal to minimal rates of oxidative phosphorylation.

r_n
Implementation Name: r_n
Units: cm
Initial value: 0.0187
Normal blood vessel radius. Normal effective blood vessel radius.

$S_{a,O_2,n}$
Implementation Name: SaO2_n
Units: dimensionless
Initial value: 0.96
Normal arterial oxygen saturation.

S_{a,O_2}
Implementation Name: SaO2sup
Units: dimensionless
Initial value: $S_{a,O_2,n}$
Arterial oxygen saturation.

$S_{c,O_2,n}$
Implementation Name: ScO2_n
Units: dimensionless
Initial value: $\frac{S_{a,O_2,n} + S_{v,O_2,n}}{2}$
Normal capillary oxygen saturation.

$S_{v,O_2,n}$
Implementation Name: SvO2_n
Units: dimensionless
Initial value: $\frac{HbO_{2,v,n}}{Hb_{tot,n}}$
Normal venous oxygen saturation.

t
Implementation Name: `t`
Units: s
Initial value: 0
Time over which the system evolves.

τ_{CO_2}
Implementation Name: `t_c`
Units: s
Initial value: 5
Filter time constant for stimulus effect of carbon dioxide.

τ_{O_2}
Implementation Name: `t_o`
Units: s
Initial value: 20
Filter time constant for stimulus effect of capillary oxygen.

τ_{P_a}
Implementation Name: `t_p`
Units: s
Initial value: 5
Filter time constant for stimulus effect of blood pressure.

τ_u
Implementation Name: `t_u`
Units: s
Initial value: 0.5
Filter time constant for stimulus effect of demand.

u
Implementation Name: `u`
Units: dimensionless
Initial value: u_n
Parameter indicating metabolic demand.

u_n
Implementation Name: `u_n`
Units: dimensionless
Initial value: 1
Normal demand.

$v_{CO_2,n}$
Implementation Name: `v_cn`
Units: mmHg
Initial value: $Pa_{CO_{2,n}}$
Normal filtered carbon dioxide partial pressure. Normal filtered carbon dioxide partial pressure.

$v_{O_2,n}$
Implementation Name: `v_on`
Units: mM
Initial value: $O_{2,c,n}$
Normal filtered capillary oxygen concentration. Normal filtered capillary oxygen concentration.

$v_{P_a,n}$
Implementation Name: `v_pn`
Units: mmHg
Initial value: $P_{a,n}$

Normal filtered arterial blood pressure. Normal filtered blood pressure.

$v_{u,n}$
Implementation Name: v_un
Units: dimensionless
Initial value: u_n
Normal filtered demand. Normal filtered demand.

$VArat_n$
Implementation Name: VArat_n
Units: dimensionless
Initial value: 3
Normal volume ratio of veins to arteries in brain tissue.

$V_{a,n}$
Implementation Name: Vol_artn
Units: dimensionless
Initial value: $\frac{1}{1 + VArat_n}$
Normal relative arterial blood volume.

Vol_{mit}
Implementation Name: Vol_mit
Units: dimensionless
Initial value: 0.067
Fraction of brain tissue volume that is mitochondria.

V_v
Implementation Name: Vol_ven
Units: dimensionless
Initial value: $\frac{VArat_n}{1 + VArat_n}$
Relative venous blood volume.

$HbO_{2,a} = Hb_{tot}\, S_{a,O_2}$
Implementation Name: XOa
Units: mM
Initial value: $HbO_{2,a,n}$
Arterial concentration of oxygen bound to haemoglobin.

$HbO_{2,a,n}$
Implementation Name: XOa_n
Units: mM
Initial value: $Hb_{tot,n}\, S_{a,O_2,n}$
Normal arterial concentration of oxygen bound to haemoglobin.

$HbO_{2,v,n}$
Implementation Name: XOv_n
Units: mM
Initial value: $\frac{CBF_n\, HbO_{2,a,n} - J_{O_{2,n}}}{CBF_n}$
Normal venous concentration of oxygen bound to haemoglobin.

Hb_{tot}
Implementation Name: Xtot
Units: mM
Initial value: 9.1
Total concentration of haemoglobin O_2 binding sites in blood (4 times haemoglobin concentration).

$Hb_{tot,n}$
Implementation Name: Xtot_n

Units: mM
Initial value: 9.1
Normal total concentration of haemoglobin O_2 binding sites in blood (4 times haemoglobin concentration).

Z

Implementation Name: `Z`
Units: mV
Initial value: 59.028
Proportionality constant in calculation of driving forces due to concentration differences. Defined as RT/F, where F is Faraday's constant, R the ideal gas constant and T the absolute temperature.

6 BSM0

6.1 Overview

Simplified model in which the metabolic submodel is replaced with variant M0.

- 9 differential state variables
- 3 algebraic state variables
- 27 intermediate variables
- 96 parameters
- 4 declared inputs
- 33 default outputs

6.2 Differential Equations

$$\frac{\mathrm{d}Cu_{A,o}}{\mathrm{d}t} = 4f_3 - 4f_1 \tag{6.1}$$

$$\frac{\mathrm{d}a_{3,r}}{\mathrm{d}t} = 4f_3 - 4f_3 \tag{6.2}$$

$$\frac{\mathrm{d}\psi}{\mathrm{d}t} = \frac{p_3\, f_3 + p_1\, f_1 + p_3\, f_3 - L}{C_{im}} \tag{6.3}$$

$$\frac{\mathrm{d}H^+}{\mathrm{d}t} = \frac{1}{R_{Hi}}\, L - \frac{p_3}{R_{Hi}}\, f_3 - \frac{p_1}{R_{Hi}}\, f_1 - \frac{p_3}{R_{Hi}}\, f_3 \tag{6.4}$$

$$\frac{\mathrm{d}O_2}{\mathrm{d}t} = \frac{1}{Vol_{mit}}\, J_{O_2} - f_3 \tag{6.5}$$

$$\frac{\mathrm{d}\nu_{CO_2}}{\mathrm{d}t} = \frac{1}{\tau_{CO_2}} \left(Pa_{CO_2} - \nu_{CO_2}\right) \tag{6.6}$$

$$\frac{\mathrm{d}\nu_{O_2}}{\mathrm{d}t} = \frac{1}{\tau_{O_2}} \left(O_{2,c} - \nu_{O_2}\right) \tag{6.7}$$

$$\frac{\mathrm{d}\nu_{P_a}}{\mathrm{d}t} = \frac{1}{\tau_{P_a}} \left(P_a - \nu_{P_a}\right) \tag{6.8}$$

$$\frac{\mathrm{d}\nu_u}{\mathrm{d}t} = \frac{1}{\tau_u} \left(u - \nu_u\right) \tag{6.9}$$

6.3 Algebraic Equations

$$\phi \left(\frac{S_{c,O_2}}{1 - S_{c,O_2}}\right)^{\frac{1}{n_h}} - O_{2,c} = 0 \tag{6.10}$$

$$T_e + T_m - \left(P_1 - P_{ic}\right) r = 0 \tag{6.11}$$

$$CBF\ \left(HbO_{2,a} - HbO_{2,v}\right) - J_{O_2} = 0 \tag{6.12}$$

6.4 Chemical Reactions

$$\xrightarrow{L} \frac{1}{R_{Hi}}\ \mathrm{H}^+ \tag{6.13}$$

$$\xrightarrow{J_{O_2}} \frac{1}{Vol_{mit}}\ \mathrm{O}_2 \tag{6.14}$$

$$\frac{p_3}{R_{Hi}}\ \mathrm{H}^+ \xrightarrow{f_3} 4\ \mathrm{Cu}_{\mathrm{A,o}} + 4\ \mathrm{a}_{3,\mathrm{r}} \tag{6.15}$$

$$4\ \mathrm{Cu}_{\mathrm{A,o}} + \frac{p_1}{R_{Hi}}\ \mathrm{H}^+ \xrightarrow{f_1} \tag{6.16}$$

$$\mathrm{O}_2 + 4\ \mathrm{a}_{3,\mathrm{r}} + \frac{p_3}{R_{Hi}}\ \mathrm{H}^+ \xrightarrow{f_3} \tag{6.17}$$

6.5 State Variables

$Cu_{A,o}$
Implementation Name: `a`
Units: mM
Initial value: $Cu_{A,o,n}$
Concentration of oxidised cytochrome c oxidase.

$a_{3,r}$
Implementation Name: `bred`
Units: mM
Initial value: $a_{3,r,n}$
Concentration of reduced cytochrome a_3.

ψ
Implementation Name: `Dpsi`
Units: mV
Initial value: ψ_n
Mitochondrial inner membrane potential. Varies as charge (in the form of protons) is transferred across the membrane capacitance.

H^+
Implementation Name: `H`
Units: mM
Initial value: H_n^+
Mitochondrial proton concentration.

O_2
Implementation Name: `O2`
Units: mM
Initial value: $O_{2,n}$
Mitochondrial oxygen concentration.

$O_{2,c}$
Implementation Name: `O2c`
Units: mM
Initial value: $O_{2,c,n}$
Capillary oxygen concentration.

r
Implementation Name: `r`
Units: cm
Initial value: r_n
Typical blood vessel radius.

ν_{CO_2}
Implementation Name: `v_c`
Units: mmHg
Initial value: $\nu_{CO_2,n}$
Filtered carbon dioxide partial pressure.

ν_{O_2}
Implementation Name: `v_o`
Units: mM
Initial value: $\nu_{O_2,n}$
Filtered capillary oxygen concentration.

ν_{P_a}
Implementation Name: `v_p`
Units: mmHg
Initial value: $\nu_{P_a,n}$
Filtered arterial blood pressure.

ν_u
Implementation Name: `v_u`
Units: dimensionless
Initial value: $\nu_{u,n}$
Filtered demand.

$HbO_{2,v}$
Implementation Name: `XOv`
Units: mM
Initial value: $HbO_{2,v,n}$
Venous concentration of oxygen bound to haemoglobin.

6.6 Intermediate Variables

$CBF = G\,(P_a - P_v)$
Implementation Name: `CBF`
Units: $ml_{blood}\ ml_{brain}^{-1}\ s^{-1}$
Initial value: CBF_n
Cerebral blood flow.

$\Delta oxCCO = \Delta oxCCO_{off} + 1000Vol_{mit}\,(Cu_{A,o} - Cu_{A,o,n})$
Implementation Name: `CCO`
Units: uM
Initial value: 0
Cytochrome c oxidase signal measured by NIRS.

$CMRO_2 = f_3\,Vol_{mit}$
Implementation Name: `CMRO2`
Units: mM s^{-1}
Initial value: 0
Rate of cerebral oxygen metabolism.

$\Delta p = \psi - Z\,(4 + \log 10\,(H^+))$
Implementation Name: `Dp`
Units: mV
Initial value: 0
Proton motive force across the mitochondrial inner membrane.

$\eta = R_{P_a}\left(\frac{v_{P_a}}{v_{P_a,n}} - 1\right) + R_{O_2}\left(\frac{v_{O_2}}{v_{O_2,n}} - 1\right) + R_{CO_2}\left(1 - \frac{v_{CO_2}}{v_{CO_2,n}}\right) + R_u\left(1 - \frac{v_u}{v_{u,n}}\right)$
Implementation Name: `eta`
Units: dimensionless
Initial value: 0
Merged autoregulation stimulus.

$f_1 = \lambda_{f_1} + \lambda_{f_1,u}\log(u) + \lambda_{f_1,p}\,\Delta p + \lambda_{f_1,a}\log(Cu_{A,o})$
Implementation Name: `f1`
Units: mM s^{-1}
Initial value: 0
Reaction rate for the reduction of Cu_A.

$f_3 = \lambda_{f_2} + \lambda_{f_2,p}\log(\Delta p) + \lambda_{f_2,a}\log(Cu_{A,o}) + \lambda_{f_2,b}\log(a_{3,r})$
Implementation Name: `f2`
Units: mM s^{-1}
Initial value: 0
Reaction rate for the reduction of a_3.

$f_3 = \lambda_{f_3} + \lambda_{f_3,p}\log(\Delta p) + \lambda_{f_3,O}\log(O_2) + \lambda_{f_3,b}\log(a_{3,r})$
Implementation Name: `f3`
Units: mM s^{-1}
Initial value: 0
Reaction rate for the reduction of O_2.

$G = K_G\,r^4$
Implementation Name: `G`
Units: $ml_{blood}\ ml_{brain}^{-1}\ mmHg^{-1}\ s^{-1}$
Initial value: 0
Effective conductance of the whole blood flow compartment.

$h = \mathrm{sqrt}\,(r\,r + 2r_0\,h_0 + h_0\,h_0) - r$
Implementation Name: `h`
Units: cm

Initial value: h_n
Thickness of the blood vessel walls.

$HbO_2 = (V_a\, HbO_{2,a} + V_v\, HbO_{2,v})\ blood_{hb}$
Implementation Name: `HbO2`
Units: uM
Initial value: 0
Oxygenated haemoglobin signal measured by NIRS.

$HbT = (V_a + V_v)\ Hb_{tot}\, blood_{hb}$
Implementation Name: `HbT`
Units: uM
Initial value: 0
Total haemoglobin signal measured by NIRS.

$HHb = HbT - HbO_2$
Implementation Name: `HHb`
Units: uM
Initial value: 0
Deoxygenated haemoglobin signal measured by NIRS.

$J_{O_2} = \mathrm{fmin}\left(D_{O_2}\ (O_{2,c} - O_2)\,, CBF\, HbO_{2,a}\right)$
Implementation Name: `J_O2`
Units: mM s^{-1}
Initial value: 0
Oxygen flux from blood to tissue.

$L = \lambda_L + \lambda_{L,\theta}\,\theta + \lambda_{L,p}\,\Delta p$
Implementation Name: `L`
Units: mM s^{-1}
Initial value: 0
Rate of proton return to the mitochondrial matrix.

$\mu = \dfrac{\mu_{min} + \mu_{max}\exp(\eta)}{1 + \exp(\eta)}$
Implementation Name: `mu`
Units: dimensionless
Initial value: 0
Effective strength of the autoregulation reponse.

$R_{Hi} = \dfrac{R_{Hi,H}}{H^+}$
Implementation Name: `R_Hi`
Units: dimensionless
Initial value: 0
Relative mitochondrial volume for protons, taking into account buffering effect of pH.

$S_{c,O_2} = \dfrac{S_{a,O_2} + S_{v,O_2}}{2}$
Implementation Name: `ScO2`
Units: dimensionless
Initial value: $S_{c,O_2,n}$
Capillary oxygen saturation.

$\sigma_e = \sigma_{e,0}\left(\exp\left(\dfrac{K_\sigma\ (r - r_0)}{r_0}\right) - 1\right) - \sigma_{coll}$
Implementation Name: `sigma_e`
Units: mm Hg
Initial value: 0
Elastic stress in blood vessel walls.

$S_{v,O_2} = \frac{HbO_{2,v}}{Hb_{tot}}$
Implementation Name: `SvO2`
Units: dimensionless
Initial value: $S_{v,O_2,n}$
Venous oxygen saturation.

$T_e = \sigma_e h$
Implementation Name: `T_e`
Units: mm Hg cm
Initial value: 0
Elastic tension in the blood vessel walls.

$T_m = T_{max} \exp\left(-\mathrm{pow}\left(\mathrm{fabs}\left(\frac{r - r_m}{r_t - r_m}\right), n_m\right)\right)$
Implementation Name: `T_m`
Units: mm Hg cm
Initial value: 0
Muscular tension in the blood vessel walls.

$T_{max} = T_{max,0}\ (1 + k_{aut}\ \mu)$
Implementation Name: `T_max`
Units: mm Hg cm
Initial value: 0
Maximal muscular tension in the blood vessel walls.

$\theta = kCV\ (\Delta p + Z \log 10\,(u) - 90)$
Implementation Name: `theta`
Units: dimensionless
Initial value: 0
Driving force Complex V.

$TOI = \frac{100 HbO_2}{HbT}$
Implementation Name: `TOI`
Units: dimensionless
Initial value: 0
Total oxygenation index.

$V_{mca} = CBF\, CBFscale$
Implementation Name: `Vmca`
Units: cm s^{-1}
Initial value: 0
Blood velocity in the middle cerebral artery.

$V_a = V_{a,n} \left(\frac{r}{r_n}\right)^2$
Implementation Name: `Vol_art`
Units: dimensionless
Initial value: 0
Relative arterial blood volume.

6.7 Parameters

$Cu_{A,o,n}$
Implementation Name: `a_n`
Units: mM

Initial value: 0.06567
Normal concentration of oxidised cytochrome c oxidase.

$blood_{hb}$
Implementation Name: `blood_hb`
Units: dimensionless
Initial value: 10.00
Factor to convert model haemoglobin concentration to instrumental units. Scales for blood fraction of brain volume, mM to μM, and number of binding sites.

$a_{3,r,n}$
Implementation Name: `bred_n`
Units: mM
Initial value: 0.001408
Normal concentration of reduced cytochrome a3.

C_{im}
Implementation Name: `C_im`
Units: mM mV^{-1}
Initial value: 0.00675
Capacitance of the mitochondrial inner membrane.

CBF_n
Implementation Name: `CBFn`
Units: $\mathrm{ml}_{blood}\ \mathrm{ml}_{brain}^{-1}\ \mathrm{s}^{-1}$
Initial value: 0.0125
Normal cerebral blood flow.

$CBFscale$
Implementation Name: `CBFscale`
Units: cm
Initial value: 5000
Scale constant relating blood flow to arterial velocity.

$\Delta oxCCO_{off}$
Implementation Name: `CCO_offset`
Units: uM
Initial value: 0
Signal offset for the NIRS CCO measurement.

$CMRO_{2,n}$
Implementation Name: `CMRO2_n`
Units: mM s^{-1}
Initial value: 0.034
Normal metabolic rate of oxygen consumption.

D_{O_2}
Implementation Name: `D_O2`
Units: s^{-1}
Initial value: $\dfrac{J_{O_{2,n}}}{O_{2,c,n} - O_{2,n}}$
Diffusion rate for oxygen between capillaries and mitochondria.

ψ_n
Implementation Name: `Dpsi_n`
Units: mV
Initial value: 145
Normal mitochondrial inner membrane potential.

λ_{f_1}
Implementation Name: `f1_0`

Units: mM s^{-1}
Initial value: 0.1221
Fitted intercept for the linear model for f_1.

$\lambda_{f_1,a}$
Implementation Name: f1_a
Units: mM s^{-1}
Initial value: 0.1848
Fitted linear dependence of f_1 on logarithm of $Cu_{A,ox}$.

$\lambda_{f_1,p}$
Implementation Name: f1_Dp
Units: mM s^{-1} mV^{-1}
Initial value: 0.005270
Fitted linear dependence of f_1 on Δp.

$\lambda_{f_1,u}$
Implementation Name: f1_u
Units: mM s^{-1}
Initial value: 0.1087
Fitted linear dependence of f_1 on logarithm of demand.

λ_{f_2}
Implementation Name: f2_0
Units: mM s^{-1}
Initial value: 5.432
Fitted intercept for the linear model for f_2.

$\lambda_{f_2,a}$
Implementation Name: f2_a
Units: mM s^{-1}
Initial value: 1.173
Fitted linear dependence of f_2 on logarithm of $Cu_{A,ox}$.

$\lambda_{f_2,b}$
Implementation Name: f2_bred
Units: mM s^{-1}
Initial value: 0.08545
Fitted linear dependence of f_2 on logarithm of $a_{3,red}$.

$\lambda_{f_2,p}$
Implementation Name: f2_Dp
Units: mM s^{-1} mV^{-1}
Initial value: -0.006935
Fitted linear dependence of f_2 on Δp.

λ_{f_3}
Implementation Name: f3_0
Units: mM s^{-1}
Initial value: 11.69
Fitted intercept for the linear model for f_3.

$\lambda_{f_3,b}$
Implementation Name: f3_bred
Units: mM s^{-1}
Initial value: 0.3649
Fitted linear dependence of f_3 on logarithm of $a_{3,red}$.

$\lambda_{f_3,p}$
Implementation Name: f3_Dp
Units: mM s^{-1} mV^{-1}

Initial value: -0.04345
Fitted linear dependence of f_3 on Δp.

$\lambda_{f_3,O}$
Implementation Name: f3_O2
Units: mM s^{-1}
Initial value: 0.3923
Fitted linear dependence of f_3 on logarithm of O_2.

G_n
Implementation Name: Gn
Units: $ml_{blood}\ ml_{brain}^{-1}\ mmHg^{-1}\ s^{-1}$
Initial value: $\frac{CBF_n}{P_{a,n} - P_{v,n}}$
Normal blood vessel conductance.

h_0
Implementation Name: h_0
Units: cm
Initial value: 0.003
Thickness of the blood vessel walls at which radius is r_0.

H_n^+
Implementation Name: H_n
Units: mM
Initial value: 0.00003981
Normal mitochondrial proton concentration.

h_n
Implementation Name: h_n
Units: cm
Initial value: $\mathrm{sqrt}\left(r_n\, r_n + 2 r_0\, h_0 + h_0\, h_0\right) - r_n$
Normal thickness of the blood vessel walls.

$J_{O_{2,n}}$
Implementation Name: J_O2n
Units: mM s^{-1}
Initial value: $CMRO_{2,n}$
Normal oxygen flux from blood to tissue.

k_{aut}
Implementation Name: k_aut
Units: dimensionless
Initial value: 1
Overall functioning of autoregulatory response.

K_G
Implementation Name: K_G
Units: $ml_{blood}\ ml_{brain}^{-1}\ mmHg^{-1}\ s^{-1}\ cm^{-4}$
Initial value: $\frac{G_n}{\mathrm{pow}\left(r_n, 4\right)}$
Proportionality constant in Poiseuille relation for conductance.

K_σ
Implementation Name: K_sigma
Units: dimensionless
Initial value: 10
Parameter controlling the sensitivity of σ_e to vessel radius.

kCV
Implementation Name: kCV

Units: mV^{-1}
Initial value: 0.02047339
Factor relating the Complex V driving force to the membrane potential and demand.

λ_L
Implementation Name: L_0
Units: mM s^{-1}
Initial value: -15.339464
Fitted intercept for the linear model for L.

$\lambda_{L,p}$
Implementation Name: L_Dp
Units: mM s^{-1} mV^{-1}
Initial value: 0.097097
Fitted linear dependence of L on Δp.

$\lambda_{L,\theta}$
Implementation Name: L_th
Units: mM s^{-1}
Initial value: 5.665904
Fitted linear dependence of L on θ.

μ_{max}
Implementation Name: mu_max
Units: dimensionless
Initial value: 1
Upper bound for the transformed stimulus μ.

μ_{min}
Implementation Name: mu_min
Units: dimensionless
Initial value: -1
Lower bound for the transformed stimulus μ.

μ_n
Implementation Name: mu_n
Units: dimensionless
Initial value: 0
Normal value for the transformed stimulus μ.

n_h
Implementation Name: n_h
Units: dimensionless
Initial value: 2.5
Hill coefficient for oxygen dissociation from haemoglobin.

n_m
Implementation Name: n_m
Units: dimensionless
Initial value: 1.83
Exponent in the muscular tension relationship.

$O_{2,n}$
Implementation Name: O2_n
Units: mM
Initial value: 0.024
Normal mitochondrial oxygen concentration.

$O_{2,c,n}$
Implementation Name: O2c_n
Units: mM

Initial value: $\phi\,\mathrm{pow}\left(\frac{S_{c,O_2,n}}{1-S_{c,O_2,n}}, \frac{1}{n_h}\right)$
Normal capillary oxygen concentration.

p_1
Implementation Name: `p1`
Units: dimensionless
Initial value: 12
Proton cost of the reaction reducing Cu_A.

p_3
Implementation Name: `p2`
Units: dimensionless
Initial value: 4
Proton cost of the reaction reducing a_3.

p_3
Implementation Name: `p3`
Units: dimensionless
Initial value: 4
Proton cost of the reaction reducing O_2.

$P_1 = \frac{P_a + P_v}{2}$
Implementation Name: `P_1`
Units: mm Hg
Initial value: $P_{1,n}$
Average pressure in the blood vessels.

$P_{1,n}$
Implementation Name: `P_1n`
Units: mm Hg
Initial value: $\frac{P_{a,n} + P_{v,n}}{2}$
Normal value for the average pressure in the blood vessels.

P_a
Implementation Name: `P_a`
Units: mmHg
Initial value: $P_{a,n}$
Mean arterial blood pressure.

$P_{a,n}$
Implementation Name: `P_an`
Units: mmHg
Initial value: 100
Normal arterial blood pressure.

P_{ic}
Implementation Name: `P_ic`
Units: mm Hg
Initial value: 9.5
Intracranial pressure.

P_{icn}
Implementation Name: `P_icn`
Units: mm Hg
Initial value: 9.5
Normal intracranial pressure.

P_v

Implementation Name: P_v
Units: mmHg
Initial value: $P_{v,n}$
Venous blood pressure.

$P_{v,n}$
Implementation Name: P_vn
Units: mmHg
Initial value: 4
Normal venous blood pressure.

Pa_{CO_2}
Implementation Name: Pa_CO2
Units: mmHg
Initial value: $Pa_{CO_{2,n}}$
Arterial partial pressure of carbon dioxide.

$Pa_{CO_{2,n}}$
Implementation Name: Pa_CO2n
Units: mmHg
Initial value: 40
Normal arterial partial pressure of carbon dioxide.

ϕ
Implementation Name: phi
Units: mM
Initial value: 0.036
Oxygen concentration at half-maximal saturation.

r_0
Implementation Name: r_0
Units: cm
Initial value: 0.0126
Radius in the elastic tension relationship.

R_{CO_2}
Implementation Name: R_autc
Units: dimensionless
Initial value: 2.2
Autoregulatory reactivity to carbon dioxide.

R_{O_2}
Implementation Name: R_auto
Units: dimensionless
Initial value: 1.5
Autoregulatory reactivity to oxygen.

R_{P_a}
Implementation Name: R_autp
Units: dimensionless
Initial value: 4
Autoregulatory reactivity to blood pressure.

R_u
Implementation Name: R_autu
Units: dimensionless
Initial value: 0.5
Autoregulatory reactivity to demand.

$R_{Hi,H}$
Implementation Name: R_Hi_H

Units: mM
Initial value: 9.565483
Proton buffering factor.

r_m
Implementation Name: r_m
Units: cm
Initial value: 0.027
Vessel radius at which muscular tension is maximal.

r_n
Implementation Name: r_n
Units: cm
Initial value: 0.0187
Normal blood vessel radius. Normal effective blood vessel radius.

r_t
Implementation Name: r_t
Units: cm
Initial value: 0.018
Radius in the muscular tension relationship.

$S_{a,O_2,n}$
Implementation Name: SaO2_n
Units: dimensionless
Initial value: 0.96
Normal arterial oxygen saturation.

S_{a,O_2}
Implementation Name: SaO2sup
Units: dimensionless
Initial value: $S_{a,O_2,n}$
Arterial oxygen saturation.

$S_{c,O_2,n}$
Implementation Name: ScO2_n
Units: dimensionless
Initial value: $\frac{S_{a,O_2,n} + S_{v,O_2,n}}{2}$
Normal capillary oxygen saturation.

σ_{coll}
Implementation Name: sigma_coll
Units: mm Hg
Initial value: 62.79
Pressure at which blood vessels collapse.

$\sigma_{e,0}$
Implementation Name: sigma_e0
Units: mm Hg
Initial value: 0.1425
Parameter in the elastic tension relationship.

$\sigma_{e,n}$
Implementation Name: sigma_en
Units: mm Hg
Initial value: $\sigma_{e,0}\left(\exp\left(\frac{K_\sigma\ (r_n - r_0)}{r_0}\right) - 1\right) - \sigma_{coll}$
Normal elastic stress in blood vessel walls.

$S_{v,O_2,n}$

Implementation Name: SvO2_n
Units: dimensionless
Initial value: $\frac{HbO_{2,v,n}}{Hb_{tot,n}}$
Normal venous oxygen saturation.

t
Implementation Name: t
Units: s
Initial value: 0
Time over which the system evolves.

τ_{CO_2}
Implementation Name: t_c
Units: s
Initial value: 5
Filter time constant for stimulus effect of carbon dioxide.

$T_{e,n}$
Implementation Name: T_en
Units: mm Hg cm
Initial value: $\sigma_{e,n}\, h_n$
Normal elastic tension in the blood vessel walls.

$T_{max,0}$
Implementation Name: T_max0
Units: mm Hg cm
Initial value: $\frac{T_{max,n}}{1 + k_{aut}\, \mu_n}$
Maximal muscular tension under normal regulatory stimulus ($\mu = \mu_n$).

$T_{max,n}$
Implementation Name: T_maxn
Units: mm Hg cm
Initial value: $\frac{T_{m,n}}{\exp\left(-\text{pow}\left(\text{fabs}\left(\frac{r_n - r_m}{r_t - r_m}\right), n_m\right)\right)}$
Normal maximal muscular tension.

$T_{m,n}$
Implementation Name: T_mn
Units: mm Hg cm
Initial value: $(P_{1,n} - P_{icn})\; r_n - T_{e,n}$
Normal muscular tension in the blood vessel walls.

τ_{O_2}
Implementation Name: t_o
Units: s
Initial value: 20
Filter time constant for stimulus effect of capillary oxygen.

τ_{P_a}
Implementation Name: t_p
Units: s
Initial value: 5
Filter time constant for stimulus effect of blood pressure.

τ_u
Implementation Name: t_u
Units: s

Initial value: 0.5
Filter time constant for stimulus effect of demand.

u
Implementation Name: `u`
Units: dimensionless
Initial value: u_n
Parameter indicating metabolic demand.

u_n
Implementation Name: `u_n`
Units: dimensionless
Initial value: 1
Normal demand.

$v_{CO_2,n}$
Implementation Name: `v_cn`
Units: mmHg
Initial value: $Pa_{CO_{2,n}}$
Normal filtered carbon dioxide partial pressure. Normal filtered carbon dioxide partial pressure.

$v_{O_2,n}$
Implementation Name: `v_on`
Units: mM
Initial value: $O_{2,c,n}$
Normal filtered capillary oxygen concentration. Normal filtered capillary oxygen concentration.

$v_{P_a,n}$
Implementation Name: `v_pn`
Units: mmHg
Initial value: $P_{a,n}$
Normal filtered arterial blood pressure. Normal filtered blood pressure.

$v_{u,n}$
Implementation Name: `v_un`
Units: dimensionless
Initial value: u_n
Normal filtered demand. Normal filtered demand.

$VArat_n$
Implementation Name: `VArat_n`
Units: dimensionless
Initial value: 3
Normal volume ratio of veins to arteries in brain tissue.

$V_{a,n}$
Implementation Name: `Vol_artn`
Units: dimensionless
Initial value: $\frac{1}{1+VArat_n}$
Normal relative arterial blood volume.

Vol_{mit}
Implementation Name: `Vol_mit`
Units: dimensionless
Initial value: 0.067
Fraction of brain tissue volume that is mitochondria.

V_v

Implementation Name: Vol_ven
Units: dimensionless
Initial value: $\dfrac{VArat_n}{1 + VArat_n}$
Relative venous blood volume.

$HbO_{2,a} = Hb_{tot}\, S_{a,O_2}$
Implementation Name: XOa
Units: mM
Initial value: $HbO_{2,a,n}$
Arterial concentration of oxygen bound to haemoglobin.

$HbO_{2,a,n}$
Implementation Name: XOa_n
Units: mM
Initial value: $Hb_{tot,n}\, S_{a,O_2,n}$
Normal arterial concentration of oxygen bound to haemoglobin.

$HbO_{2,v,n}$
Implementation Name: XOv_n
Units: mM
Initial value: $\dfrac{CBF_n\, HbO_{2,a,n} - J_{O_{2,n}}}{CBF_n}$
Normal venous concentration of oxygen bound to haemoglobin.

Hb_{tot}
Implementation Name: Xtot
Units: mM
Initial value: 9.1
Total concentration of haemoglobin O_2 binding sites in blood (4 times haemoglobin concentration).

$Hb_{tot,n}$
Implementation Name: Xtot_n
Units: mM
Initial value: 9.1
Normal total concentration of haemoglobin O_2 binding sites in blood (4 times haemoglobin concentration).

Z
Implementation Name: Z
Units: mV
Initial value: 59.028
Proportionality constant in calculation of driving forces due to concentration differences. Defined as RT/F, where F is Faraday's constant, R the ideal gas constant and T the absolute temperature.

7 BSM1

7.1 Overview

Simplified model in which the metabolic submodel is replaced with variant M1.

- 9 differential state variables
- 3 algebraic state variables
- 27 intermediate variables
- 91 parameters
- 4 declared inputs
- 33 default outputs

7.2 Differential Equations

$$\frac{\mathrm{d}Cu_{A,o}}{\mathrm{d}t} = 4f_3 - 4f_1 \tag{7.1}$$

$$\frac{\mathrm{d}a_{3,r}}{\mathrm{d}t} = 4f_3 - 4f_3 \tag{7.2}$$

$$\frac{\mathrm{d}\psi}{\mathrm{d}t} = \frac{p_3\, f_3 + p_1\, f_1 + p_3\, f_3 - L}{C_{im}} \tag{7.3}$$

$$\frac{\mathrm{d}H^+}{\mathrm{d}t} = \frac{1}{R_{Hi}}\, L - \frac{p_3}{R_{Hi}}\, f_3 - \frac{p_1}{R_{Hi}}\, f_1 - \frac{p_3}{R_{Hi}}\, f_3 \tag{7.4}$$

$$\frac{\mathrm{d}O_2}{\mathrm{d}t} = \frac{1}{Vol_{mit}}\, J_{O_2} - f_3 \tag{7.5}$$

$$\frac{\mathrm{d}v_{CO_2}}{\mathrm{d}t} = \frac{1}{\tau_{CO_2}}\, \left(Pa_{CO_2} - v_{CO_2}\right) \tag{7.6}$$

$$\frac{\mathrm{d}v_{O_2}}{\mathrm{d}t} = \frac{1}{\tau_{O_2}}\, \left(O_{2,c} - v_{O_2}\right) \tag{7.7}$$

$$\frac{\mathrm{d}v_{P_a}}{\mathrm{d}t} = \frac{1}{\tau_{P_a}}\, \left(P_a - v_{P_a}\right) \tag{7.8}$$

$$\frac{\mathrm{d}\nu_u}{\mathrm{d}t} = \frac{1}{\tau_u}\,(u - \nu_u) \tag{7.9}$$

7.3 Algebraic Equations

$$\phi\left(\frac{S_{c,O_2}}{1 - S_{c,O_2}}\right)^{\frac{1}{n_h}} - O_{2,c} = 0 \tag{7.10}$$

$$T_e + T_m - (P_1 - P_{ic})\,r = 0 \tag{7.11}$$

$$CBF\,(HbO_{2,a} - HbO_{2,v}) - J_{O_2} = 0 \tag{7.12}$$

7.4 Chemical Reactions

$$\xrightarrow{L} \frac{1}{R_{Hi}}\,\mathrm{H}^+ \tag{7.13}$$

$$\xrightarrow{J_{O_2}} \frac{1}{Vol_{mit}}\,\mathrm{O}_2 \tag{7.14}$$

$$\frac{p_3}{R_{Hi}}\,\mathrm{H}^+ \xrightarrow{f_3} 4\,\mathrm{Cu}_{\mathrm{A,o}} + 4\,\mathrm{a}_{3,\mathrm{r}} \tag{7.15}$$

$$4\,\mathrm{Cu}_{\mathrm{A,o}} + \frac{p_1}{R_{Hi}}\,\mathrm{H}^+ \xrightarrow{f_1} \tag{7.16}$$

$$\mathrm{O}_2 + 4\,\mathrm{a}_{3,\mathrm{r}} + \frac{p_3}{R_{Hi}}\,\mathrm{H}^+ \xrightarrow{f_3} \tag{7.17}$$

7.5 State Variables

$Cu_{A,o}$
Implementation Name: `a`
Units: mM
Initial value: $Cu_{A,o,n}$
Concentration of oxidised cytochrome c oxidase.

$a_{3,r}$
Implementation Name: `bred`
Units: mM
Initial value: $a_{3,r,n}$
Concentration of reduced cytochrome a_3.

ψ
Implementation Name: `Dpsi`
Units: mV
Initial value: ψ_n
Mitochondrial inner membrane potential. Varies as charge (in the form of protons) is transferred across the membrane capacitance.

H^+
Implementation Name: `H`
Units: mM
Initial value: H_n^+
Mitochondrial proton concentration.

O_2
Implementation Name: `O2`
Units: mM
Initial value: $O_{2,n}$
Mitochondrial oxygen concentration.

$O_{2,c}$
Implementation Name: `O2c`
Units: mM
Initial value: $O_{2,c,n}$
Capillary oxygen concentration.

r
Implementation Name: `r`
Units: cm
Initial value: r_n
Typical blood vessel radius.

ν_{CO_2}
Implementation Name: `v_c`
Units: mmHg
Initial value: $\nu_{CO_2,n}$
Filtered carbon dioxide partial pressure.

ν_{O_2}
Implementation Name: `v_o`
Units: mM
Initial value: $\nu_{O_2,n}$
Filtered capillary oxygen concentration.

ν_{P_a}
Implementation Name: `v_p`
Units: mmHg
Initial value: $\nu_{P_a,n}$
Filtered arterial blood pressure.

ν_u
Implementation Name: `v_u`
Units: dimensionless
Initial value: $\nu_{u,n}$
Filtered demand.

$HbO_{2,v}$
Implementation Name: `XOv`
Units: mM
Initial value: $HbO_{2,v,n}$
Venous concentration of oxygen bound to haemoglobin.

7.6 Intermediate Variables

$CBF \; = G \; (P_a - P_v)$
Implementation Name: `CBF`
Units: $\mathrm{ml}_{blood}\ \mathrm{ml}_{brain}^{-1}\ \mathrm{s}^{-1}$
Initial value: CBF_n
Cerebral blood flow.

$\Delta oxCCO \; = \Delta oxCCO_{off} + 1000 Vol_{mit} \; (Cu_{A,o} - Cu_{A,o,n})$
Implementation Name: `CCO`
Units: uM
Initial value: 0
Cytochrome c oxidase signal measured by NIRS.

$CMRO_2 \; = f_3 \, Vol_{mit}$
Implementation Name: `CMRO2`
Units: $\mathrm{mM\ s}^{-1}$
Initial value: 0
Rate of cerebral oxygen metabolism.

$\Delta p \; = \psi - Z \; (4 + \log10\left(H^+\right))$
Implementation Name: `Dp`
Units: mV
Initial value: 0
Proton motive force across the mitochondrial inner membrane.

$\eta \; = R_{P_a} \left(\frac{\nu_{P_a}}{\nu_{P_a,n}} - 1 \right) + R_{O_2} \left(\frac{\nu_{O_2}}{\nu_{O_2,n}} - 1 \right) + R_{CO_2} \left(1 - \frac{\nu_{CO_2}}{\nu_{CO_2,n}} \right) + R_u \left(1 - \frac{\nu_u}{\nu_{u,n}} \right)$
Implementation Name: `eta`
Units: dimensionless
Initial value: 0
Merged autoregulation stimulus.

$f_1 \; = \lambda_{f_1} + \lambda_{f_1,u} \log\left(u\right) + \lambda_{f_1,a} \log\left(Cu_{A,o}\right)$
Implementation Name: `f1`
Units: $\mathrm{mM\ s}^{-1}$
Initial value: 0
Reaction rate for the reduction of Cu_A.

$f_3 \; = \lambda_{f_2} + \lambda_{f_2,b} \log\left(a_{3,r}\right)$
Implementation Name: `f2`
Units: $\mathrm{mM\ s}^{-1}$
Initial value: 0
Reaction rate for the reduction of a_3.

$f_3 \; = \lambda_{f_3} + \lambda_{f_3,O} \log\left(O_2\right)$
Implementation Name: `f3`
Units: $\mathrm{mM\ s}^{-1}$
Initial value: 0
Reaction rate for the reduction of O_2.

$G \; = K_G \, r^4$
Implementation Name: `G`
Units: $\mathrm{ml}_{blood}\ \mathrm{ml}_{brain}^{-1}\ \mathrm{mmHg}^{-1}\ \mathrm{s}^{-1}$
Initial value: 0
Effective conductance of the whole blood flow compartment.

$h \; = \mathrm{sqrt}\left(r\, r + 2 r_0\, h_0 + h_0\, h_0\right) - r$
Implementation Name: `h`
Units: cm

Initial value: h_n
Thickness of the blood vessel walls.

$HbO_2 = (V_a\, HbO_{2,a} + V_v\, HbO_{2,v})\; blood_{hb}$
Implementation Name: `HbO2`
Units: uM
Initial value: 0
Oxygenated haemoglobin signal measured by NIRS.

$HbT = (V_a + V_v)\; Hb_{tot}\, blood_{hb}$
Implementation Name: `HbT`
Units: uM
Initial value: 0
Total haemoglobin signal measured by NIRS.

$HHb = HbT - HbO_2$
Implementation Name: `HHb`
Units: uM
Initial value: 0
Deoxygenated haemoglobin signal measured by NIRS.

$J_{O_2} = \mathrm{fmin}\left(D_{O_2}\,(O_{2,c} - O_2)\,, CBF\, HbO_{2,a}\right)$
Implementation Name: `J_O2`
Units: mM s^{-1}
Initial value: 0
Oxygen flux from blood to tissue.

$L = \lambda_L + \lambda_{L,\theta}\,\theta + \lambda_{L,p}\,\Delta p$
Implementation Name: `L`
Units: mM s^{-1}
Initial value: 0
Rate of proton return to the mitochondrial matrix.

$\mu = \dfrac{\mu_{min} + \mu_{max}\exp(\eta)}{1 + \exp(\eta)}$
Implementation Name: `mu`
Units: dimensionless
Initial value: 0
Effective strength of the autoregulation reponse.

$R_{Hi} = \dfrac{R_{Hi,H}}{H^+}$
Implementation Name: `R_Hi`
Units: dimensionless
Initial value: 0
Relative mitochondrial volume for protons, taking into account buffering effect of pH.

$S_{c,O_2} = \dfrac{S_{a,O_2} + S_{v,O_2}}{2}$
Implementation Name: `ScO2`
Units: dimensionless
Initial value: $S_{c,O_2,n}$
Capillary oxygen saturation.

$\sigma_e = \sigma_{e,0}\left(\exp\left(\dfrac{K_\sigma\,(r - r_0)}{r_0}\right) - 1\right) - \sigma_{coll}$
Implementation Name: `sigma_e`
Units: mm Hg
Initial value: 0
Elastic stress in blood vessel walls.

$S_{v,O_2} = \frac{HbO_{2,v}}{Hb_{tot}}$
Implementation Name: `SvO2`
Units: dimensionless
Initial value: $S_{v,O_2,n}$
Venous oxygen saturation.

$T_e = \sigma_e h$
Implementation Name: `T_e`
Units: mm Hg cm
Initial value: 0
Elastic tension in the blood vessel walls.

$T_m = T_{max} \exp\left(-\mathrm{pow}\left(\mathrm{fabs}\left(\frac{r - r_m}{r_t - r_m}\right), n_m\right)\right)$
Implementation Name: `T_m`
Units: mm Hg cm
Initial value: 0
Muscular tension in the blood vessel walls.

$T_{max} = T_{max,0}\ (1 + k_{aut}\ \mu)$
Implementation Name: `T_max`
Units: mm Hg cm
Initial value: 0
Maximal muscular tension in the blood vessel walls.

$\theta = kCV\ (\Delta p + Z \log10(u) - 90)$
Implementation Name: `theta`
Units: dimensionless
Initial value: 0
Driving force Complex V.

$TOI = \frac{100HbO_2}{HbT}$
Implementation Name: `TOI`
Units: dimensionless
Initial value: 0
Total oxygenation index.

$V_{mca} = CBF\,CBFscale$
Implementation Name: `Vmca`
Units: cm s^{-1}
Initial value: 0
Blood velocity in the middle cerebral artery.

$V_a = V_{a,n} \left(\frac{r}{r_n}\right)^2$
Implementation Name: `Vol_art`
Units: dimensionless
Initial value: 0
Relative arterial blood volume.

7.7 Parameters

$Cu_{A,o,n}$
Implementation Name: `a_n`
Units: mM

Initial value: 0.06567
Normal concentration of oxidised cytochrome c oxidase.

$blood_{hb}$
Implementation Name: `blood_hb`
Units: dimensionless
Initial value: 10.00
Factor to convert model haemoglobin concentration to instrumental units. Scales for blood fraction of brain volume, mM to μM, and number of binding sites.

$a_{3,r,n}$
Implementation Name: `bred_n`
Units: mM
Initial value: 0.001408
Normal concentration of reduced cytochrome a3.

C_{im}
Implementation Name: `C_im`
Units: mM mV^{-1}
Initial value: 0.00675
Capacitance of the mitochondrial inner membrane.

CBF_n
Implementation Name: `CBFn`
Units: ml$_{blood}$ ml$_{brain}^{-1}$ s^{-1}
Initial value: 0.0125
Normal cerebral blood flow.

$CBFscale$
Implementation Name: `CBFscale`
Units: cm
Initial value: 5000
Scale constant relating blood flow to arterial velocity.

$\Delta oxCCO_{off}$
Implementation Name: `CCO_offset`
Units: uM
Initial value: 0
Signal offset for the NIRS CCO measurement.

$CMRO_{2,n}$
Implementation Name: `CMRO2_n`
Units: mM s^{-1}
Initial value: 0.034
Normal metabolic rate of oxygen consumption.

D_{O_2}
Implementation Name: `D_O2`
Units: s^{-1}
Initial value: $\dfrac{J_{O_{2,n}}}{O_{2,c,n} - O_{2,n}}$
Diffusion rate for oxygen between capillaries and mitochondria.

ψ_n
Implementation Name: `Dpsi_n`
Units: mV
Initial value: 145
Normal mitochondrial inner membrane potential.

λ_{f_1}
Implementation Name: `f1_0`

Units: mM s^{-1}
Initial value: 1.490
Fitted intercept for the linear model for f_1.

$\lambda_{f_1,a}$
Implementation Name: `f1_a`
Units: mM s^{-1}
Initial value: 0.3609
Fitted linear dependence of f_1 on logarithm of $Cu_{A,ox}$.

$\lambda_{f_1,u}$
Implementation Name: `f1_u`
Units: mM s^{-1}
Initial value: 0.06985
Fitted linear dependence of f_1 on logarithm of demand.

λ_{f_2}
Implementation Name: `f2_0`
Units: mM s^{-1}
Initial value: 0.1473
Fitted intercept for the linear model for f_2.

$\lambda_{f_2,b}$
Implementation Name: `f2_bred`
Units: mM s^{-1}
Initial value: -0.05484
Fitted linear dependence of f_2 on logarithm of $a_{3,red}$.

λ_{f_3}
Implementation Name: `f3_0`
Units: mM s^{-1}
Initial value: 0.6324
Fitted intercept for the linear model for f_3.

$\lambda_{f_3,O}$
Implementation Name: `f3_O2`
Units: mM s^{-1}
Initial value: 0.03352
Fitted linear dependence of f_3 on logarithm of O_2.

G_n
Implementation Name: `Gn`
Units: $ml_{blood}\ ml_{brain}^{-1}\ mmHg^{-1}\ s^{-1}$
Initial value: $\dfrac{CBF_n}{P_{a,n} - P_{v,n}}$
Normal blood vessel conductance.

h_0
Implementation Name: `h_0`
Units: cm
Initial value: 0.003
Thickness of the blood vessel walls at which radius is r_0.

H_n^+
Implementation Name: `H_n`
Units: mM
Initial value: 0.00003981
Normal mitochondrial proton concentration.

h_n
Implementation Name: `h_n`

Units: cm
Initial value: sqrt $(r_n\, r_n + 2r_0\, h_0 + h_0\, h_0) - r_n$
Normal thickness of the blood vessel walls.

$J_{O_{2,n}}$
Implementation Name: J_O2n
Units: mM s^{-1}
Initial value: $CMRO_{2,n}$
Normal oxygen flux from blood to tissue.

k_{aut}
Implementation Name: k_aut
Units: dimensionless
Initial value: 1
Overall functioning of autoregulatory response.

K_G
Implementation Name: K_G
Units: $ml_{blood}\ ml_{brain}^{-1}\ mmHg^{-1}\ s^{-1}\ cm^{-4}$
Initial value: $\frac{G_n}{\text{pow}\,(r_n, 4)}$
Proportionality constant in Poiseuille relation for conductance.

K_σ
Implementation Name: K_sigma
Units: dimensionless
Initial value: 10
Parameter controlling the sensitivity of σ_e to vessel radius.

kCV
Implementation Name: kCV
Units: mV^{-1}
Initial value: 0.02047339
Factor relating the Complex V driving force to the membrane potential and demand.

λ_L
Implementation Name: L_0
Units: mM s^{-1}
Initial value: -15.339464
Fitted intercept for the linear model for L.

$\lambda_{L,p}$
Implementation Name: L_Dp
Units: mM s^{-1} mV^{-1}
Initial value: 0.097097
Fitted linear dependence of L on Δp.

$\lambda_{L,\theta}$
Implementation Name: L_th
Units: mM s^{-1}
Initial value: 5.665904
Fitted linear dependence of L on θ.

μ_{max}
Implementation Name: mu_max
Units: dimensionless
Initial value: 1
Upper bound for the transformed stimulus μ.

μ_{min}
Implementation Name: mu_min

Units: dimensionless
Initial value: -1
Lower bound for the transformed stimulus μ.

μ_n
Implementation Name: mu_n
Units: dimensionless
Initial value: 0
Normal value for the transformed stimulus μ.

n_h
Implementation Name: n_h
Units: dimensionless
Initial value: 2.5
Hill coefficient for oxygen dissociation from haemoglobin.

n_m
Implementation Name: n_m
Units: dimensionless
Initial value: 1.83
Exponent in the muscular tension relationship.

$O_{2,n}$
Implementation Name: O2_n
Units: mM
Initial value: 0.024
Normal mitochondrial oxygen concentration.

$O_{2,c,n}$
Implementation Name: O2c_n
Units: mM
Initial value: $\phi\,\mathrm{pow}\left(\frac{S_{c,O_2,n}}{1-S_{c,O_2,n}}, \frac{1}{n_h}\right)$
Normal capillary oxygen concentration.

p_1
Implementation Name: p1
Units: dimensionless
Initial value: 12
Proton cost of the reaction reducing Cu_A.

p_3
Implementation Name: p2
Units: dimensionless
Initial value: 4
Proton cost of the reaction reducing a_3.

p_3
Implementation Name: p3
Units: dimensionless
Initial value: 4
Proton cost of the reaction reducing O_2.

$P_1 = \frac{P_a + P_v}{2}$
Implementation Name: P_1
Units: mm Hg
Initial value: $P_{1,n}$
Average pressure in the blood vessels.

$P_{1,n}$

Implementation Name: P_1n
Units: mm Hg
Initial value: $\frac{P_{a,n} + P_{v,n}}{2}$
Normal value for the average pressure in the blood vessels.

P_a
Implementation Name: P_a
Units: mmHg
Initial value: $P_{a,n}$
Mean arterial blood pressure.

$P_{a,n}$
Implementation Name: P_an
Units: mmHg
Initial value: 100
Normal arterial blood pressure.

P_{ic}
Implementation Name: P_ic
Units: mm Hg
Initial value: 9.5
Intracranial pressure.

P_{icn}
Implementation Name: P_icn
Units: mm Hg
Initial value: 9.5
Normal intracranial pressure.

P_v
Implementation Name: P_v
Units: mmHg
Initial value: $P_{v,n}$
Venous blood pressure.

$P_{v,n}$
Implementation Name: P_vn
Units: mmHg
Initial value: 4
Normal venous blood pressure.

Pa_{CO_2}
Implementation Name: Pa_CO2
Units: mmHg
Initial value: $Pa_{CO_{2,n}}$
Arterial partial pressure of carbon dioxide.

$Pa_{CO_{2,n}}$
Implementation Name: Pa_CO2n
Units: mmHg
Initial value: 40
Normal arterial partial pressure of carbon dioxide.

ϕ
Implementation Name: phi
Units: mM
Initial value: 0.036
Oxygen concentration at half-maximal saturation.

r_0

Implementation Name: r_0
Units: cm
Initial value: 0.0126
Radius in the elastic tension relationship.

R_{CO_2}
Implementation Name: R_autc
Units: dimensionless
Initial value: 2.2
Autoregulatory reactivity to carbon dioxide.

R_{O_2}
Implementation Name: R_auto
Units: dimensionless
Initial value: 1.5
Autoregulatory reactivity to oxygen.

R_{P_a}
Implementation Name: R_autp
Units: dimensionless
Initial value: 4
Autoregulatory reactivity to blood pressure.

R_u
Implementation Name: R_autu
Units: dimensionless
Initial value: 0.5
Autoregulatory reactivity to demand.

$R_{Hi,H}$
Implementation Name: R_Hi_H
Units: mM
Initial value: 9.565483
Proton buffering factor.

r_m
Implementation Name: r_m
Units: cm
Initial value: 0.027
Vessel radius at which muscular tension is maximal.

r_n
Implementation Name: r_n
Units: cm
Initial value: 0.0187
Normal blood vessel radius. Normal effective blood vessel radius.

r_t
Implementation Name: r_t
Units: cm
Initial value: 0.018
Radius in the muscular tension relationship.

$S_{a,O_2,n}$
Implementation Name: SaO2_n
Units: dimensionless
Initial value: 0.96
Normal arterial oxygen saturation.

S_{a,O_2}
Implementation Name: SaO2sup

Units: dimensionless
Initial value: $S_{a,O_2,n}$
Arterial oxygen saturation.

$S_{c,O_2,n}$
Implementation Name: `ScO2_n`
Units: dimensionless
Initial value: $\frac{S_{a,O_2,n} + S_{v,O_2,n}}{2}$
Normal capillary oxygen saturation.

σ_{coll}
Implementation Name: `sigma_coll`
Units: mm Hg
Initial value: 62.79
Pressure at which blood vessels collapse.

$\sigma_{e,0}$
Implementation Name: `sigma_e0`
Units: mm Hg
Initial value: 0.1425
Parameter in the elastic tension relationship.

$\sigma_{e,n}$
Implementation Name: `sigma_en`
Units: mm Hg
Initial value: $\sigma_{e,0}\left(\exp\left(\frac{K_\sigma\,(r_n - r_0)}{r_0}\right) - 1\right) - \sigma_{coll}$
Normal elastic stress in blood vessel walls.

$S_{v,O_2,n}$
Implementation Name: `SvO2_n`
Units: dimensionless
Initial value: $\frac{HbO_{2,v,n}}{Hb_{tot,n}}$
Normal venous oxygen saturation.

t
Implementation Name: `t`
Units: s
Initial value: 0
Time over which the system evolves.

τ_{CO_2}
Implementation Name: `t_c`
Units: s
Initial value: 5
Filter time constant for stimulus effect of carbon dioxide.

$T_{e,n}$
Implementation Name: `T_en`
Units: mm Hg cm
Initial value: $\sigma_{e,n}\,h_n$
Normal elastic tension in the blood vessel walls.

$T_{max,0}$
Implementation Name: `T_max0`
Units: mm Hg cm
Initial value: $\frac{T_{max,n}}{1 + k_{aut}\,\mu_n}$
Maximal muscular tension under normal regulatory stimulus ($\mu = \mu_n$).

$T_{max,n}$

Implementation Name: T_maxn
Units: mm Hg cm
Initial value: $\frac{T_{m,n}}{\exp\left(-\text{pow}\left(\text{fabs}\left(\frac{r_n - r_m}{r_t - r_m}\right), n_m\right)\right)}$
Normal maximal muscular tension.

$T_{m,n}$

Implementation Name: T_mn
Units: mm Hg cm
Initial value: $(P_{1,n} - P_{icn})\, r_n - T_{e,n}$
Normal muscular tension in the blood vessel walls.

τ_{O_2}

Implementation Name: t_o
Units: s
Initial value: 20
Filter time constant for stimulus effect of capillary oxygen.

τ_{P_a}

Implementation Name: t_p
Units: s
Initial value: 5
Filter time constant for stimulus effect of blood pressure.

τ_u

Implementation Name: t_u
Units: s
Initial value: 0.5
Filter time constant for stimulus effect of demand.

u

Implementation Name: u
Units: dimensionless
Initial value: u_n
Parameter indicating metabolic demand.

u_n

Implementation Name: u_n
Units: dimensionless
Initial value: 1
Normal demand.

$v_{CO_2,n}$

Implementation Name: v_cn
Units: mmHg
Initial value: $Pa_{CO_{2,n}}$
Normal filtered carbon dioxide partial pressure. Normal filtered carbon dioxide partial pressure.

$v_{O_2,n}$

Implementation Name: v_on
Units: mM
Initial value: $O_{2,c,n}$
Normal filtered capillary oxygen concentration. Normal filtered capillary oxygen concentration.

$v_{P_a,n}$

Implementation Name: v_pn
Units: mmHg

Initial value: $P_{a,n}$
Normal filtered arterial blood pressure. Normal filtered blood pressure.

$v_{u,n}$
Implementation Name: `v_un`
Units: dimensionless
Initial value: u_n
Normal filtered demand. Normal filtered demand.

$VArat_n$
Implementation Name: `VArat_n`
Units: dimensionless
Initial value: 3
Normal volume ratio of veins to arteries in brain tissue.

$V_{a,n}$
Implementation Name: `Vol_artn`
Units: dimensionless
Initial value: $\frac{1}{1 + VArat_n}$
Normal relative arterial blood volume.

Vol_{mit}
Implementation Name: `Vol_mit`
Units: dimensionless
Initial value: 0.067
Fraction of brain tissue volume that is mitochondria.

V_v
Implementation Name: `Vol_ven`
Units: dimensionless
Initial value: $\frac{VArat_n}{1 + VArat_n}$
Relative venous blood volume.

$HbO_{2,a} \; = Hb_{tot}\, S_{a,O_2}$
Implementation Name: `XOa`
Units: mM
Initial value: $HbO_{2,a,n}$
Arterial concentration of oxygen bound to haemoglobin.

$HbO_{2,a,n}$
Implementation Name: `XOa_n`
Units: mM
Initial value: $Hb_{tot,n}\, S_{a,O_2,n}$
Normal arterial concentration of oxygen bound to haemoglobin.

$HbO_{2,v,n}$
Implementation Name: `XOv_n`
Units: mM
Initial value: $\frac{CBF_n\, HbO_{2,a,n} - J_{O_{2,n}}}{CBF_n}$
Normal venous concentration of oxygen bound to haemoglobin.

Hb_{tot}
Implementation Name: `Xtot`
Units: mM
Initial value: 9.1
Total concentration of haemoglobin O_2 binding sites in blood (4 times haemoglobin concentration).

$Hb_{tot,n}$

Implementation Name: `Xtot_n`
Units: mM
Initial value: 9.1
Normal total concentration of haemoglobin O_2 binding sites in blood (4 times haemoglobin concentration).

Z

Implementation Name: `Z`
Units: mV
Initial value: 59.028
Proportionality constant in calculation of driving forces due to concentration differences. Defined as RT/F, where F is Faraday's constant, R the ideal gas constant and T the absolute temperature.

8 BSM2

8.1 Overview

Simplified model in which the metabolic submodel is replaced with variant M2.

- 9 differential state variables
- 3 algebraic state variables
- 27 intermediate variables
- 90 parameters
- 4 declared inputs
- 33 default outputs

8.2 Differential Equations

$$\frac{\mathrm{d}Cu_{A,o}}{\mathrm{d}t} = 4f_3 - 4f_1 \tag{8.1}$$

$$\frac{\mathrm{d}a_{3,r}}{\mathrm{d}t} = 4f_3 - 4f_3 \tag{8.2}$$

$$\frac{\mathrm{d}\psi}{\mathrm{d}t} = \frac{p_3\, f_3 + p_1\, f_1 + p_3\, f_3 - L}{C_{im}} \tag{8.3}$$

$$\frac{\mathrm{d}H^+}{\mathrm{d}t} = \frac{1}{R_{Hi}}\, L - \frac{p_3}{R_{Hi}}\, f_3 - \frac{p_1}{R_{Hi}}\, f_1 - \frac{p_3}{R_{Hi}}\, f_3 \tag{8.4}$$

$$\frac{\mathrm{d}O_2}{\mathrm{d}t} = \frac{1}{Vol_{mit}}\, J_{O_2} - f_3 \tag{8.5}$$

$$\frac{\mathrm{d}\nu_{CO_2}}{\mathrm{d}t} = \frac{1}{\tau_{CO_2}}\, \left(Pa_{CO_2} - \nu_{CO_2}\right) \tag{8.6}$$

$$\frac{\mathrm{d}\nu_{O_2}}{\mathrm{d}t} = \frac{1}{\tau_{O_2}}\, \left(O_{2,c} - \nu_{O_2}\right) \tag{8.7}$$

$$\frac{\mathrm{d}\nu_{P_a}}{\mathrm{d}t} = \frac{1}{\tau_{P_a}}\, \left(P_a - \nu_{P_a}\right) \tag{8.8}$$

$$\frac{\mathrm{d}\nu_u}{\mathrm{d}t} = \frac{1}{\tau_u}\,(u - \nu_u) \tag{8.9}$$

8.3 Algebraic Equations

$$\phi\left(\frac{S_{c,O_2}}{1 - S_{c,O_2}}\right)^{\frac{1}{n_h}} - O_{2,c} = 0 \tag{8.10}$$

$$T_e + T_m - (P_1 - P_{ic})\,r = 0 \tag{8.11}$$

$$CBF\,(HbO_{2,a} - HbO_{2,v}) - J_{O_2} = 0 \tag{8.12}$$

8.4 Chemical Reactions

$$\xrightarrow{L} \frac{1}{R_{Hi}}\,\mathrm{H}^+ \tag{8.13}$$

$$\xrightarrow{J_{O_2}} \frac{1}{Vol_{mit}}\,\mathrm{O}_2 \tag{8.14}$$

$$\frac{p_3}{R_{Hi}}\,\mathrm{H}^+ \xrightarrow{f_3} 4\,\mathrm{Cu}_{\mathrm{A,o}} + 4\,\mathrm{a}_{3,\mathrm{r}} \tag{8.15}$$

$$4\,\mathrm{Cu}_{\mathrm{A,o}} + \frac{p_1}{R_{Hi}}\,\mathrm{H}^+ \xrightarrow{f_1} \tag{8.16}$$

$$\mathrm{O}_2 + 4\,\mathrm{a}_{3,\mathrm{r}} + \frac{p_3}{R_{Hi}}\,\mathrm{H}^+ \xrightarrow{f_3} \tag{8.17}$$

8.5 State Variables

$Cu_{A,o}$

Implementation Name: `a`
Units: mM
Initial value: $Cu_{A,o,n}$
Concentration of oxidised cytochrome c oxidase.

$a_{3,r}$

Implementation Name: `bred`
Units: mM
Initial value: $a_{3,r,n}$
Concentration of reduced cytochrome a_3.

ψ
Implementation Name: `Dpsi`
Units: mV
Initial value: ψ_n
Mitochondrial inner membrane potential. Varies as charge (in the form of protons) is transferred across the membrane capacitance.

H^+
Implementation Name: `H`
Units: mM
Initial value: H_n^+
Mitochondrial proton concentration.

O_2
Implementation Name: `O2`
Units: mM
Initial value: $O_{2,n}$
Mitochondrial oxygen concentration.

$O_{2,c}$
Implementation Name: `O2c`
Units: mM
Initial value: $O_{2,c,n}$
Capillary oxygen concentration.

r
Implementation Name: `r`
Units: cm
Initial value: r_n
Typical blood vessel radius.

ν_{CO_2}
Implementation Name: `v_c`
Units: mmHg
Initial value: $\nu_{CO_2,n}$
Filtered carbon dioxide partial pressure.

ν_{O_2}
Implementation Name: `v_o`
Units: mM
Initial value: $\nu_{O_2,n}$
Filtered capillary oxygen concentration.

ν_{P_a}
Implementation Name: `v_p`
Units: mmHg
Initial value: $\nu_{P_a,n}$
Filtered arterial blood pressure.

ν_u
Implementation Name: `v_u`
Units: dimensionless
Initial value: $\nu_{u,n}$
Filtered demand.

$HbO_{2,v}$
Implementation Name: `XOv`
Units: mM
Initial value: $HbO_{2,v,n}$
Venous concentration of oxygen bound to haemoglobin.

8.6 Intermediate Variables

$CBF = G\,(P_a - P_v)$
Implementation Name: `CBF`
Units: $ml_{blood}\ ml_{brain}^{-1}\ s^{-1}$
Initial value: CBF_n
Cerebral blood flow.

$\Delta oxCCO = \Delta oxCCO_{off} + 1000 Vol_{mit}\,(Cu_{A,o} - Cu_{A,o,n})$
Implementation Name: `CCO`
Units: uM
Initial value: 0
Cytochrome c oxidase signal measured by NIRS.

$CMRO_2 = f_3\, Vol_{mit}$
Implementation Name: `CMRO2`
Units: mM s^{-1}
Initial value: 0
Rate of cerebral oxygen metabolism.

$\Delta p = \psi - Z\,\left(4 + \log10\left(H^+\right)\right)$
Implementation Name: `Dp`
Units: mV
Initial value: 0
Proton motive force across the mitochondrial inner membrane.

$\eta = R_{P_a}\left(\frac{\nu_{P_a}}{\nu_{P_a,n}} - 1\right) + R_{O_2}\left(\frac{\nu_{O_2}}{\nu_{O_2,n}} - 1\right) + R_{CO_2}\left(1 - \frac{\nu_{CO_2}}{\nu_{CO_2,n}}\right) + R_u\left(1 - \frac{\nu_u}{\nu_{u,n}}\right)$
Implementation Name: `eta`
Units: dimensionless
Initial value: 0
Merged autoregulation stimulus.

$f_1 = \lambda_{f_1} + \lambda_{f_1,a}\log\left(Cu_{A,o}\right)$
Implementation Name: `f1`
Units: mM s^{-1}
Initial value: 0
Reaction rate for the reduction of Cu_A.

$f_3 = \lambda_{f_2} + \lambda_{f_2,b}\log\left(a_{3,r}\right)$
Implementation Name: `f2`
Units: mM s^{-1}
Initial value: 0
Reaction rate for the reduction of a_3.

$f_3 = \lambda_{f_3} + \lambda_{f_3,O}\log\left(O_2\right)$
Implementation Name: `f3`
Units: mM s^{-1}
Initial value: 0
Reaction rate for the reduction of O_2.

$G = K_G\, r^4$
Implementation Name: `G`
Units: $ml_{blood}\ ml_{brain}^{-1}\ mmHg^{-1}\ s^{-1}$
Initial value: 0
Effective conductance of the whole blood flow compartment.

$h = \mathrm{sqrt}\left(r\,r + 2 r_0\, h_0 + h_0\, h_0\right) - r$
Implementation Name: `h`
Units: cm

Initial value: h_n
Thickness of the blood vessel walls.

$HbO_2 = (V_a\, HbO_{2,a} + V_v\, HbO_{2,v})\, blood_{hb}$
Implementation Name: `HbO2`
Units: uM
Initial value: 0
Oxygenated haemoglobin signal measured by NIRS.

$HbT = (V_a + V_v)\, Hb_{tot}\, blood_{hb}$
Implementation Name: `HbT`
Units: uM
Initial value: 0
Total haemoglobin signal measured by NIRS.

$HHb = HbT - HbO_2$
Implementation Name: `HHb`
Units: uM
Initial value: 0
Deoxygenated haemoglobin signal measured by NIRS.

$J_{O_2} = \text{fmin}\left(D_{O_2}\,(O_{2,c} - O_2)\,, CBF\, HbO_{2,a}\right)$
Implementation Name: `J_O2`
Units: mM s^{-1}
Initial value: 0
Oxygen flux from blood to tissue.

$L = \lambda_L + \lambda_{L,\theta}\,\theta + \lambda_{L,p}\,\Delta p$
Implementation Name: `L`
Units: mM s^{-1}
Initial value: 0
Rate of proton return to the mitochondrial matrix.

$\mu = \dfrac{\mu_{min} + \mu_{max}\exp(\eta)}{1 + \exp(\eta)}$
Implementation Name: `mu`
Units: dimensionless
Initial value: 0
Effective strength of the autoregulation reponse.

$R_{Hi} = \dfrac{R_{Hi,H}}{H^+}$
Implementation Name: `R_Hi`
Units: dimensionless
Initial value: 0
Relative mitochondrial volume for protons, taking into account buffering effect of pH.

$S_{c,O_2} = \dfrac{S_{a,O_2} + S_{v,O_2}}{2}$
Implementation Name: `ScO2`
Units: dimensionless
Initial value: $S_{c,O_2,n}$
Capillary oxygen saturation.

$\sigma_e = \sigma_{e,0}\left(\exp\left(\dfrac{K_\sigma\,(r - r_0)}{r_0}\right) - 1\right) - \sigma_{coll}$
Implementation Name: `sigma_e`
Units: mm Hg
Initial value: 0
Elastic stress in blood vessel walls.

$S_{v,O_2} = \frac{HbO_{2,v}}{Hb_{tot}}$
Implementation Name: SvO2
Units: dimensionless
Initial value: $S_{v,O_2,n}$
Venous oxygen saturation.

$T_e = \sigma_e h$
Implementation Name: T_e
Units: mm Hg cm
Initial value: 0
Elastic tension in the blood vessel walls.

$T_m = T_{max} \exp\left(-\text{pow}\left(\text{fabs}\left(\frac{r - r_m}{r_t - r_m}\right), n_m\right)\right)$
Implementation Name: T_m
Units: mm Hg cm
Initial value: 0
Muscular tension in the blood vessel walls.

$T_{max} = T_{max,0}\ (1 + k_{aut}\ \mu)$
Implementation Name: T_max
Units: mm Hg cm
Initial value: 0
Maximal muscular tension in the blood vessel walls.

$\theta = kCV\ (\Delta p + Z \log10(u) - 90)$
Implementation Name: theta
Units: dimensionless
Initial value: 0
Driving force Complex V.

$TOI = \frac{100 HbO_2}{HbT}$
Implementation Name: TOI
Units: dimensionless
Initial value: 0
Total oxygenation index.

$V_{mca} = CBF\, CBFscale$
Implementation Name: Vmca
Units: cm s^{-1}
Initial value: 0
Blood velocity in the middle cerebral artery.

$V_a = V_{a,n} \left(\frac{r}{r_n}\right)^2$
Implementation Name: Vol_art
Units: dimensionless
Initial value: 0
Relative arterial blood volume.

8.7 Parameters

$Cu_{A,o,n}$
Implementation Name: a_n
Units: mM

Initial value: 0.06567
Normal concentration of oxidised cytochrome c oxidase.

$blood_{hb}$
Implementation Name: `blood_hb`
Units: dimensionless
Initial value: 10.00
Factor to convert model haemoglobin concentration to instrumental units. Scales for blood fraction of brain volume, mM to μM, and number of binding sites.

$a_{3,r,n}$
Implementation Name: `bred_n`
Units: mM
Initial value: 0.001408
Normal concentration of reduced cytochrome a3.

C_{im}
Implementation Name: `C_im`
Units: mM mV^{-1}
Initial value: 0.00675
Capacitance of the mitochondrial inner membrane.

CBF_n
Implementation Name: `CBFn`
Units: ml$_{blood}$ ml$^{-1}_{brain}$ s^{-1}
Initial value: 0.0125
Normal cerebral blood flow.

$CBFscale$
Implementation Name: `CBFscale`
Units: cm
Initial value: 5000
Scale constant relating blood flow to arterial velocity.

$\Delta oxCCO_{off}$
Implementation Name: `CCO_offset`
Units: uM
Initial value: 0
Signal offset for the NIRS CCO measurement.

$CMRO_{2,n}$
Implementation Name: `CMRO2_n`
Units: mM s^{-1}
Initial value: 0.034
Normal metabolic rate of oxygen consumption.

D_{O_2}
Implementation Name: `D_O2`
Units: s^{-1}
Initial value: $\dfrac{J_{O_{2,n}}}{O_{2,c,n} - O_{2,n}}$
Diffusion rate for oxygen between capillaries and mitochondria.

ψ_n
Implementation Name: `Dpsi_n`
Units: mV
Initial value: 145
Normal mitochondrial inner membrane potential.

λ_{f_1}
Implementation Name: `f1_0`

Units: mM s^{-1}
Initial value: 1.504
Fitted intercept for the linear model for f_1.

$\lambda_{f_1,a}$

Implementation Name: f1_a
Units: mM s^{-1}
Initial value: 0.3658
Fitted linear dependence of f_1 on logarithm of $\mathrm{Cu}_{A,ox}$.

λ_{f_2}

Implementation Name: f2_0
Units: mM s^{-1}
Initial value: 0.1473
Fitted intercept for the linear model for f_2.

$\lambda_{f_2,b}$

Implementation Name: f2_bred
Units: mM s^{-1}
Initial value: -0.05484
Fitted linear dependence of f_2 on logarithm of $\mathrm{a}_{3,red}$.

λ_{f_3}

Implementation Name: f3_0
Units: mM s^{-1}
Initial value: 0.6324
Fitted intercept for the linear model for f_3.

$\lambda_{f_3,O}$

Implementation Name: f3_O2
Units: mM s^{-1}
Initial value: 0.03352
Fitted linear dependence of f_3 on logarithm of O_2.

G_n

Implementation Name: Gn
Units: $\mathrm{ml}_{blood}\ \mathrm{ml}_{brain}^{-1}\ \mathrm{mmHg}^{-1}\ \mathrm{s}^{-1}$
Initial value: $\frac{CBF_n}{P_{a,n} - P_{v,n}}$
Normal blood vessel conductance.

h_0

Implementation Name: h_0
Units: cm
Initial value: 0.003
Thickness of the blood vessel walls at which radius is r_0.

H_n^+

Implementation Name: H_n
Units: mM
Initial value: 0.00003981
Normal mitochondrial proton concentration.

h_n

Implementation Name: h_n
Units: cm
Initial value: $\mathrm{sqrt}\,(r_n\, r_n + 2 r_0\, h_0 + h_0\, h_0) - r_n$
Normal thickness of the blood vessel walls.

$J_{O_2,n}$

Implementation Name: J_O2n

Units: mM s^{-1}
Initial value: $CMRO_{2,n}$
Normal oxygen flux from blood to tissue.

k_{aut}

Implementation Name: k_aut
Units: dimensionless
Initial value: 1
Overall functioning of autoregulatory response.

K_G

Implementation Name: K_G
Units: $ml_{blood}\ ml_{brain}^{-1}\ mmHg^{-1}\ s^{-1}\ cm^{-4}$
Initial value: $\frac{G_n}{\text{pow}\,(r_n, 4)}$
Proportionality constant in Poiseuille relation for conductance.

K_σ

Implementation Name: K_sigma
Units: dimensionless
Initial value: 10
Parameter controlling the sensitivity of σ_e to vessel radius.

kCV

Implementation Name: kCV
Units: mV^{-1}
Initial value: 0.02047339
Factor relating the Complex V driving force to the membrane potential and demand.

λ_L

Implementation Name: L_0
Units: mM s^{-1}
Initial value: -15.339464
Fitted intercept for the linear model for L.

$\lambda_{L,p}$

Implementation Name: L_Dp
Units: mM s^{-1} mV^{-1}
Initial value: 0.097097
Fitted linear dependence of L on Δp.

$\lambda_{L,\theta}$

Implementation Name: L_th
Units: mM s^{-1}
Initial value: 5.665904
Fitted linear dependence of L on θ.

μ_{max}

Implementation Name: mu_max
Units: dimensionless
Initial value: 1
Upper bound for the transformed stimulus μ.

μ_{min}

Implementation Name: mu_min
Units: dimensionless
Initial value: -1
Lower bound for the transformed stimulus μ.

μ_n

Implementation Name: mu_n

Units: dimensionless
Initial value: 0
Normal value for the transformed stimulus μ.

n_h
Implementation Name: n_h
Units: dimensionless
Initial value: 2.5
Hill coefficient for oxygen dissociation from haemoglobin.

n_m
Implementation Name: n_m
Units: dimensionless
Initial value: 1.83
Exponent in the muscular tension relationship.

$O_{2,n}$
Implementation Name: O2_n
Units: mM
Initial value: 0.024
Normal mitochondrial oxygen concentration.

$O_{2,c,n}$
Implementation Name: O2c_n
Units: mM
Initial value: $\phi\,\mathrm{pow}\left(\frac{S_{c,O_2,n}}{1-S_{c,O_2,n}}, \frac{1}{n_h}\right)$
Normal capillary oxygen concentration.

p_1
Implementation Name: p1
Units: dimensionless
Initial value: 12
Proton cost of the reaction reducing Cu_A.

p_3
Implementation Name: p2
Units: dimensionless
Initial value: 4
Proton cost of the reaction reducing a_3.

p_3
Implementation Name: p3
Units: dimensionless
Initial value: 4
Proton cost of the reaction reducing O_2.

$P_1 = \frac{P_a + P_v}{2}$
Implementation Name: P_1
Units: mm Hg
Initial value: $P_{1,n}$
Average pressure in the blood vessels.

$P_{1,n}$
Implementation Name: P_1n
Units: mm Hg
Initial value: $\frac{P_{a,n} + P_{v,n}}{2}$
Normal value for the average pressure in the blood vessels.

P_a
Implementation Name: P_a
Units: mmHg
Initial value: $P_{a,n}$
Mean arterial blood pressure.

$P_{a,n}$
Implementation Name: P_an
Units: mmHg
Initial value: 100
Normal arterial blood pressure.

P_{ic}
Implementation Name: P_ic
Units: mm Hg
Initial value: 9.5
Intracranial pressure.

P_{icn}
Implementation Name: P_icn
Units: mm Hg
Initial value: 9.5
Normal intracranial pressure.

P_v
Implementation Name: P_v
Units: mmHg
Initial value: $P_{v,n}$
Venous blood pressure.

$P_{v,n}$
Implementation Name: P_vn
Units: mmHg
Initial value: 4
Normal venous blood pressure.

Pa_{CO_2}
Implementation Name: Pa_CO2
Units: mmHg
Initial value: $Pa_{CO_{2,n}}$
Arterial partial pressure of carbon dioxide.

$Pa_{CO_{2,n}}$
Implementation Name: Pa_CO2n
Units: mmHg
Initial value: 40
Normal arterial partial pressure of carbon dioxide.

ϕ
Implementation Name: phi
Units: mM
Initial value: 0.036
Oxygen concentration at half-maximal saturation.

r_0
Implementation Name: r_0
Units: cm
Initial value: 0.0126
Radius in the elastic tension relationship.

R_{CO_2}
Implementation Name: `R_autc`
Units: dimensionless
Initial value: 2.2
Autoregulatory reactivity to carbon dioxide.

R_{O_2}
Implementation Name: `R_auto`
Units: dimensionless
Initial value: 1.5
Autoregulatory reactivity to oxygen.

R_{P_a}
Implementation Name: `R_autp`
Units: dimensionless
Initial value: 4
Autoregulatory reactivity to blood pressure.

R_u
Implementation Name: `R_autu`
Units: dimensionless
Initial value: 0.5
Autoregulatory reactivity to demand.

$R_{Hi,H}$
Implementation Name: `R_Hi_H`
Units: mM
Initial value: 9.565483
Proton buffering factor.

r_m
Implementation Name: `r_m`
Units: cm
Initial value: 0.027
Vessel radius at which muscular tension is maximal.

r_n
Implementation Name: `r_n`
Units: cm
Initial value: 0.0187
Normal blood vessel radius. Normal effective blood vessel radius.

r_t
Implementation Name: `r_t`
Units: cm
Initial value: 0.018
Radius in the muscular tension relationship.

$S_{a,O_2,n}$
Implementation Name: `SaO2_n`
Units: dimensionless
Initial value: 0.96
Normal arterial oxygen saturation.

S_{a,O_2}
Implementation Name: `SaO2sup`
Units: dimensionless
Initial value: $S_{a,O_2,n}$
Arterial oxygen saturation.

$S_{c,O_2,n}$
Implementation Name: ScO2_n
Units: dimensionless
Initial value: $\frac{S_{a,O_2,n} + S_{v,O_2,n}}{2}$
Normal capillary oxygen saturation.

σ_{coll}
Implementation Name: sigma_coll
Units: mm Hg
Initial value: 62.79
Pressure at which blood vessels collapse.

$\sigma_{e,0}$
Implementation Name: sigma_e0
Units: mm Hg
Initial value: 0.1425
Parameter in the elastic tension relationship.

$\sigma_{e,n}$
Implementation Name: sigma_en
Units: mm Hg
Initial value: $\sigma_{e,0} \left(\exp\left(\frac{K_\sigma \ (r_n - r_0)}{r_0} \right) - 1 \right) - \sigma_{coll}$
Normal elastic stress in blood vessel walls.

$S_{v,O_2,n}$
Implementation Name: SvO2_n
Units: dimensionless
Initial value: $\frac{HbO_{2,v,n}}{Hb_{tot,n}}$
Normal venous oxygen saturation.

t
Implementation Name: t
Units: s
Initial value: 0
Time over which the system evolves.

τ_{CO_2}
Implementation Name: t_c
Units: s
Initial value: 5
Filter time constant for stimulus effect of carbon dioxide.

$T_{e,n}$
Implementation Name: T_en
Units: mm Hg cm
Initial value: $\sigma_{e,n} \, h_n$
Normal elastic tension in the blood vessel walls.

$T_{max,0}$
Implementation Name: T_max0
Units: mm Hg cm
Initial value: $\frac{T_{max,n}}{1 + k_{aut} \, \mu_n}$
Maximal muscular tension under normal regulatory stimulus ($\mu = \mu_n$).

$T_{max,n}$
Implementation Name: T_maxn
Units: mm Hg cm

Initial value: $\frac{T_{m,n}}{\exp\left(-\text{pow}\left(\text{fabs}\left(\frac{r_n - r_m}{r_t - r_m}\right), n_m\right)\right)}$
Normal maximal muscular tension.

$T_{m,n}$
Implementation Name: `T_mn`
Units: mm Hg cm
Initial value: $(P_{1,n} - P_{icn})\, r_n - T_{e,n}$
Normal muscular tension in the blood vessel walls.

τ_{O_2}
Implementation Name: `t_o`
Units: s
Initial value: 20
Filter time constant for stimulus effect of capillary oxygen.

τ_{P_a}
Implementation Name: `t_p`
Units: s
Initial value: 5
Filter time constant for stimulus effect of blood pressure.

τ_u
Implementation Name: `t_u`
Units: s
Initial value: 0.5
Filter time constant for stimulus effect of demand.

u
Implementation Name: `u`
Units: dimensionless
Initial value: u_n
Parameter indicating metabolic demand.

u_n
Implementation Name: `u_n`
Units: dimensionless
Initial value: 1
Normal demand.

$v_{CO_2,n}$
Implementation Name: `v_cn`
Units: mmHg
Initial value: $Pa_{CO_{2,n}}$
Normal filtered carbon dioxide partial pressure. Normal filtered carbon dioxide partial pressure.

$v_{O_2,n}$
Implementation Name: `v_on`
Units: mM
Initial value: $O_{2,c,n}$
Normal filtered capillary oxygen concentration. Normal filtered capillary oxygen concentration.

$v_{P_a,n}$
Implementation Name: `v_pn`
Units: mmHg
Initial value: $P_{a,n}$
Normal filtered arterial blood pressure. Normal filtered blood pressure.

$v_{u,n}$
Implementation Name: `v_un`
Units: dimensionless
Initial value: u_n
Normal filtered demand. Normal filtered demand.

$VArat_n$
Implementation Name: `VArat_n`
Units: dimensionless
Initial value: 3
Normal volume ratio of veins to arteries in brain tissue.

$V_{a,n}$
Implementation Name: `Vol_artn`
Units: dimensionless
Initial value: $\frac{1}{1 + VArat_n}$
Normal relative arterial blood volume.

Vol_{mit}
Implementation Name: `Vol_mit`
Units: dimensionless
Initial value: 0.067
Fraction of brain tissue volume that is mitochondria.

V_v
Implementation Name: `Vol_ven`
Units: dimensionless
Initial value: $\frac{VArat_n}{1 + VArat_n}$
Relative venous blood volume.

$HbO_{2,a} = Hb_{tot}\, S_{a,O_2}$
Implementation Name: `XOa`
Units: mM
Initial value: $HbO_{2,a,n}$
Arterial concentration of oxygen bound to haemoglobin.

$HbO_{2,a,n}$
Implementation Name: `XOa_n`
Units: mM
Initial value: $Hb_{tot,n}\, S_{a,O_2,n}$
Normal arterial concentration of oxygen bound to haemoglobin.

$HbO_{2,v,n}$
Implementation Name: `XOv_n`
Units: mM
Initial value: $\frac{CBF_n\, HbO_{2,a,n} - J_{O_{2,n}}}{CBF_n}$
Normal venous concentration of oxygen bound to haemoglobin.

Hb_{tot}
Implementation Name: `Xtot`
Units: mM
Initial value: 9.1
Total concentration of haemoglobin O_2 binding sites in blood (4 times haemoglobin concentration).

$Hb_{tot,n}$
Implementation Name: `Xtot_n`
Units: mM

Initial value: 9.1
Normal total concentration of haemoglobin O_2 binding sites in blood (4 times haemoglobin concentration).

Z

Implementation Name: `Z`
Units: mV
Initial value: 59.028
Proportionality constant in calculation of driving forces due to concentration differences. Defined as RT/F, where F is Faraday's constant, R the ideal gas constant and T the absolute temperature.

9 BSM3

9.1 Overview

Simplified model in which the metabolic submodel is replaced with variant M3.

- 9 differential state variables
- 3 algebraic state variables
- 25 intermediate variables
- 88 parameters
- 4 declared inputs
- 31 default outputs

9.2 Differential Equations

$$\frac{\mathrm{d}Cu_{A,o}}{\mathrm{d}t} = 4f^{\star} - 4f^{\star} \tag{9.1}$$

$$\frac{\mathrm{d}a_{3,r}}{\mathrm{d}t} = 4f^{\star} - 4f^{\star} \tag{9.2}$$

$$\frac{\mathrm{d}\psi}{\mathrm{d}t} = \frac{p_3\, f^{\star} + p_1\, f^{\star} + p_3\, f^{\star} - L}{C_{im}} \tag{9.3}$$

$$\frac{\mathrm{d}H^{+}}{\mathrm{d}t} = \frac{1}{R_{Hi}}\, L - \frac{p_3}{R_{Hi}}\, f^{\star} - \frac{p_1}{R_{Hi}}\, f^{\star} - \frac{p_3}{R_{Hi}}\, f^{\star} \tag{9.4}$$

$$\frac{\mathrm{d}O_2}{\mathrm{d}t} = \frac{1}{Vol_{mit}}\, J_{O_2} - f^{\star} \tag{9.5}$$

$$\frac{\mathrm{d}\nu_{CO_2}}{\mathrm{d}t} = \frac{1}{\tau_{CO_2}}\, \left(Pa_{CO_2} - \nu_{CO_2}\right) \tag{9.6}$$

$$\frac{\mathrm{d}\nu_{O_2}}{\mathrm{d}t} = \frac{1}{\tau_{O_2}}\, \left(O_{2,c} - \nu_{O_2}\right) \tag{9.7}$$

$$\frac{\mathrm{d}\nu_{P_a}}{\mathrm{d}t} = \frac{1}{\tau_{P_a}}\, \left(P_a - \nu_{P_a}\right) \tag{9.8}$$

$$\frac{\mathrm{d}\nu_u}{\mathrm{d}t} = \frac{1}{\tau_u}\,(u - \nu_u) \tag{9.9}$$

9.3 Algebraic Equations

$$\phi\left(\frac{S_{c,O_2}}{1 - S_{c,O_2}}\right)^{\frac{1}{n_h}} - O_{2,c} = 0 \tag{9.10}$$

$$T_e + T_m - (P_1 - P_{ic})\,r = 0 \tag{9.11}$$

$$CBF\,(HbO_{2,a} - HbO_{2,v}) - J_{O_2} = 0 \tag{9.12}$$

9.4 Chemical Reactions

$$\xrightarrow{L} \frac{1}{R_{Hi}}\,\mathrm{H}^+ \tag{9.13}$$

$$\xrightarrow{J_{O_2}} \frac{1}{Vol_{mit}}\,\mathrm{O}_2 \tag{9.14}$$

$$\frac{p_3}{R_{Hi}}\,\mathrm{H}^+ \xrightarrow{f^\star} 4\,\mathrm{Cu}_{\mathrm{A,o}} + 4\,\mathrm{a}_{3,\mathrm{r}} \tag{9.15}$$

$$4\,\mathrm{Cu}_{\mathrm{A,o}} + \frac{p_1}{R_{Hi}}\,\mathrm{H}^+ \xrightarrow{f^\star} \tag{9.16}$$

$$\mathrm{O}_2 + 4\,\mathrm{a}_{3,\mathrm{r}} + \frac{p_3}{R_{Hi}}\,\mathrm{H}^+ \xrightarrow{f^\star} \tag{9.17}$$

9.5 State Variables

$Cu_{A,o}$
Implementation Name: `a`
Units: mM
Initial value: $Cu_{A,o,n}$
Concentration of oxidised cytochrome c oxidase.

$a_{3,r}$
Implementation Name: `bred`
Units: mM
Initial value: $a_{3,r,n}$
Concentration of reduced cytochrome a_3.

ψ
: Implementation Name: `Dpsi`
Units: mV
Initial value: ψ_n
Mitochondrial inner membrane potential. Varies as charge (in the form of protons) is transferred across the membrane capacitance.

H^+
: Implementation Name: `H`
Units: mM
Initial value: H_n^+
Mitochondrial proton concentration.

O_2
: Implementation Name: `O2`
Units: mM
Initial value: $O_{2,n}$
Mitochondrial oxygen concentration.

$O_{2,c}$
: Implementation Name: `O2c`
Units: mM
Initial value: $O_{2,c,n}$
Capillary oxygen concentration.

r
: Implementation Name: `r`
Units: cm
Initial value: r_n
Typical blood vessel radius.

ν_{CO_2}
: Implementation Name: `v_c`
Units: mmHg
Initial value: $\nu_{CO_2,n}$
Filtered carbon dioxide partial pressure.

ν_{O_2}
: Implementation Name: `v_o`
Units: mM
Initial value: $\nu_{O_2,n}$
Filtered capillary oxygen concentration.

ν_{P_a}
: Implementation Name: `v_p`
Units: mmHg
Initial value: $\nu_{P_a,n}$
Filtered arterial blood pressure.

ν_u
: Implementation Name: `v_u`
Units: dimensionless
Initial value: $\nu_{u,n}$
Filtered demand.

$HbO_{2,v}$
: Implementation Name: `XOv`
Units: mM
Initial value: $HbO_{2,v,n}$
Venous concentration of oxygen bound to haemoglobin.

9.6 Intermediate Variables

$CBF \; = G \; (P_a - P_v)$
Implementation Name: `CBF`
Units: $\text{ml}_{blood}\ \text{ml}_{brain}^{-1}\ \text{s}^{-1}$
Initial value: CBF_n
Cerebral blood flow.

$\Delta oxCCO \; = \Delta oxCCO_{off} + 1000 Vol_{mit}\ (Cu_{A,o} - Cu_{A,o,n})$
Implementation Name: `CCO`
Units: uM
Initial value: 0
Cytochrome c oxidase signal measured by NIRS.

$CMRO_2 \; = f^{\star}\ Vol_{mit}$
Implementation Name: `CMRO2`
Units: mM s^{-1}
Initial value: 0
Rate of cerebral oxygen metabolism.

$\Delta p \; = \psi - Z\ \left(4 + \text{log10}\left(H^+\right)\right)$
Implementation Name: `Dp`
Units: mV
Initial value: 0
Proton motive force across the mitochondrial inner membrane.

$\eta \; = R_{P_a}\left(\frac{\nu_{P_a}}{\nu_{P_a,n}} - 1\right) + R_{O_2}\left(\frac{\nu_{O_2}}{\nu_{O_2,n}} - 1\right) + R_{CO_2}\left(1 - \frac{\nu_{CO_2}}{\nu_{CO_2,n}}\right) + R_u\left(1 - \frac{\nu_u}{\nu_{u,n}}\right)$
Implementation Name: `eta`
Units: dimensionless
Initial value: 0
Merged autoregulation stimulus.

$f^{\star} \; = \lambda_{f^{\star}} + \lambda_{f^{\star},p}\,\Delta p + \lambda_{f^{\star},O} \log\left(O_2\right) + \lambda_{f^{\star},a} \log\left(Cu_{A,o}\right)$
Implementation Name: `f3`
Units: mM s^{-1}
Initial value: 0
Shared reaction rate for all three electron transport reactions.

$G \; = K_G\, r^4$
Implementation Name: `G`
Units: $\text{ml}_{blood}\ \text{ml}_{brain}^{-1}\ \text{mmHg}^{-1}\ \text{s}^{-1}$
Initial value: 0
Effective conductance of the whole blood flow compartment.

$h \; = \text{sqrt}\left(r\, r + 2 r_0\, h_0 + h_0\, h_0\right) - r$
Implementation Name: `h`
Units: cm
Initial value: h_n
Thickness of the blood vessel walls.

$HbO_2 \; = \left(V_a\, HbO_{2,a} + V_v\, HbO_{2,v}\right)\ blood_{hb}$
Implementation Name: `HbO2`
Units: uM
Initial value: 0
Oxygenated haemoglobin signal measured by NIRS.

$HbT \; = \left(V_a + V_v\right)\ Hb_{tot}\, blood_{hb}$
Implementation Name: `HbT`
Units: uM

Initial value: 0
Total haemoglobin signal measured by NIRS.

$HHb = HbT - HbO_2$
Implementation Name: HHb
Units: uM
Initial value: 0
Deoxygenated haemoglobin signal measured by NIRS.

$J_{O_2} = \text{fmin}\left(D_{O_2}\left(O_{2,c} - O_2\right), CBF\, HbO_{2,a}\right)$
Implementation Name: J_O2
Units: mM s^{-1}
Initial value: 0
Oxygen flux from blood to tissue.

$L = \lambda_L + \lambda_{L,\theta}\,\theta + \lambda_{L,p}\,\Delta p$
Implementation Name: L
Units: mM s^{-1}
Initial value: 0
Rate of proton return to the mitochondrial matrix.

$\mu = \dfrac{\mu_{min} + \mu_{max}\exp(\eta)}{1 + \exp(\eta)}$
Implementation Name: mu
Units: dimensionless
Initial value: 0
Effective strength of the autoregulation reponse.

$R_{Hi} = \dfrac{R_{Hi,H}}{H^+}$
Implementation Name: R_Hi
Units: dimensionless
Initial value: 0
Relative mitochondrial volume for protons, taking into account buffering effect of pH.

$S_{c,O_2} = \dfrac{S_{a,O_2} + S_{v,O_2}}{2}$
Implementation Name: ScO2
Units: dimensionless
Initial value: $S_{c,O_2,n}$
Capillary oxygen saturation.

$\sigma_e = \sigma_{e,0}\left(\exp\left(\dfrac{K_\sigma\,(r - r_0)}{r_0}\right) - 1\right) - \sigma_{coll}$
Implementation Name: sigma_e
Units: mm Hg
Initial value: 0
Elastic stress in blood vessel walls.

$S_{v,O_2} = \dfrac{HbO_{2,v}}{Hb_{tot}}$
Implementation Name: SvO2
Units: dimensionless
Initial value: $S_{v,O_2,n}$
Venous oxygen saturation.

$T_e = \sigma_e\, h$
Implementation Name: T_e
Units: mm Hg cm
Initial value: 0
Elastic tension in the blood vessel walls.

$T_m = T_{max} \exp\left(-\text{pow}\left(\text{fabs}\left(\frac{r - r_m}{r_t - r_m}\right), n_m\right)\right)$
Implementation Name: T_m
Units: mm Hg cm
Initial value: 0
Muscular tension in the blood vessel walls.

$T_{max} = T_{max,0}\ (1 + k_{aut}\,\mu)$
Implementation Name: T_max
Units: mm Hg cm
Initial value: 0
Maximal muscular tension in the blood vessel walls.

$\theta = kCV\ (\Delta p + Z \log10(u) - 90)$
Implementation Name: theta
Units: dimensionless
Initial value: 0
Driving force Complex V.

$TOI = \frac{100HbO_2}{HbT}$
Implementation Name: TOI
Units: dimensionless
Initial value: 0
Total oxygenation index.

$V_{mca} = CBF\,CBFscale$
Implementation Name: Vmca
Units: cm s^{-1}
Initial value: 0
Blood velocity in the middle cerebral artery.

$V_a = V_{a,n}\left(\frac{r}{r_n}\right)^2$
Implementation Name: Vol_art
Units: dimensionless
Initial value: 0
Relative arterial blood volume.

9.7 Parameters

$Cu_{A,o,n}$
Implementation Name: a_n
Units: mM
Initial value: 0.06567
Normal concentration of oxidised cytochrome c oxidase.

$blood_{hb}$
Implementation Name: blood_hb
Units: dimensionless
Initial value: 10.00
Factor to convert model haemoglobin concentration to instrumental units. Scales for blood fraction of brain volume, mM to μM, and number of binding sites.

$a_{3,r,n}$
Implementation Name: bred_n
Units: mM

Initial value: 0.001408
Normal concentration of reduced cytochrome a3.

C_{im}
Implementation Name: `C_im`
Units: mM mV^{-1}
Initial value: 0.00675
Capacitance of the mitochondrial inner membrane.

CBF_n
Implementation Name: `CBFn`
Units: ml$_{blood}$ ml$_{brain}^{-1}$ s^{-1}
Initial value: 0.0125
Normal cerebral blood flow.

$CBFscale$
Implementation Name: `CBFscale`
Units: cm
Initial value: 5000
Scale constant relating blood flow to arterial velocity.

$\Delta oxCCO_{off}$
Implementation Name: `CCO_offset`
Units: uM
Initial value: 0
Signal offset for the NIRS CCO measurement.

$CMRO_{2,n}$
Implementation Name: `CMRO2_n`
Units: mM s^{-1}
Initial value: 0.034
Normal metabolic rate of oxygen consumption.

D_{O_2}
Implementation Name: `D_O2`
Units: s^{-1}
Initial value: $\dfrac{J_{O_{2,n}}}{O_{2,c,n} - O_{2,n}}$
Diffusion rate for oxygen between capillaries and mitochondria.

ψ_n
Implementation Name: `Dpsi_n`
Units: mV
Initial value: 145
Normal mitochondrial inner membrane potential.

$\lambda_{f^\star}$
Implementation Name: `f3_0`
Units: mM s^{-1}
Initial value: 13.34
Fitted intercept for the linear model for $f^\star$.

$\lambda_{f^\star,a}$
Implementation Name: `f3_a`
Units: mM s^{-1}
Initial value: 1.308
Fitted linear dependence of $f^\star$ on logarithm of $Cu_{A,ox}$.

$\lambda_{f^\star,O}$
Implementation Name: `f3_O2`
Units: mM s^{-1}

Initial value: 0.08064
Fitted linear dependence of $f^\star$ on logarithm of O_2.

$\lambda_{f^\star,p}$

Implementation Name: f3_p
Units: mM s^{-1}
Initial value: -0.05317
Fitted linear dependence of $f^\star$ on Δp.

G_n

Implementation Name: Gn
Units: $ml_{blood}\ ml_{brain}^{-1}\ mmHg^{-1}\ s^{-1}$
Initial value: $\frac{CBF_n}{P_{a,n} - P_{v,n}}$
Normal blood vessel conductance.

h_0

Implementation Name: h_0
Units: cm
Initial value: 0.003
Thickness of the blood vessel walls at which radius is r_0.

H_n^+

Implementation Name: H_n
Units: mM
Initial value: 0.00003981
Normal mitochondrial proton concentration.

h_n

Implementation Name: h_n
Units: cm
Initial value: $\mathrm{sqrt}\left(r_n\, r_n + 2r_0\, h_0 + h_0\, h_0\right) - r_n$
Normal thickness of the blood vessel walls.

$J_{O_{2,n}}$

Implementation Name: J_O2n
Units: mM s^{-1}
Initial value: $CMRO_{2,n}$
Normal oxygen flux from blood to tissue.

k_{aut}

Implementation Name: k_aut
Units: dimensionless
Initial value: 1
Overall functioning of autoregulatory response.

K_G

Implementation Name: K_G
Units: $ml_{blood}\ ml_{brain}^{-1}\ mmHg^{-1}\ s^{-1}\ cm^{-4}$
Initial value: $\frac{G_n}{\mathrm{pow}\left(r_n, 4\right)}$
Proportionality constant in Poiseuille relation for conductance.

K_σ

Implementation Name: K_sigma
Units: dimensionless
Initial value: 10
Parameter controlling the sensitivity of σ_e to vessel radius.

kCV

Implementation Name: kCV

Units: mV^{-1}
Initial value: 0.02047339
Factor relating the Complex V driving force to the membrane potential and demand.

λ_L
Implementation Name: L_0
Units: mM s^{-1}
Initial value: -15.339464
Fitted intercept for the linear model for L.

$\lambda_{L,p}$
Implementation Name: L_Dp
Units: mM s^{-1} mV^{-1}
Initial value: 0.097097
Fitted linear dependence of L on Δp.

$\lambda_{L,\theta}$
Implementation Name: L_th
Units: mM s^{-1}
Initial value: 5.665904
Fitted linear dependence of L on θ.

μ_{max}
Implementation Name: mu_max
Units: dimensionless
Initial value: 1
Upper bound for the transformed stimulus μ.

μ_{min}
Implementation Name: mu_min
Units: dimensionless
Initial value: -1
Lower bound for the transformed stimulus μ.

μ_n
Implementation Name: mu_n
Units: dimensionless
Initial value: 0
Normal value for the transformed stimulus μ.

n_h
Implementation Name: n_h
Units: dimensionless
Initial value: 2.5
Hill coefficient for oxygen dissociation from haemoglobin.

n_m
Implementation Name: n_m
Units: dimensionless
Initial value: 1.83
Exponent in the muscular tension relationship.

$O_{2,n}$
Implementation Name: O2_n
Units: mM
Initial value: 0.024
Normal mitochondrial oxygen concentration.

$O_{2,c,n}$
Implementation Name: O2c_n
Units: mM

Initial value: $\phi \,\mathrm{pow}\left(\frac{S_{c,O_2,n}}{1 - S_{c,O_2,n}}, \frac{1}{n_h}\right)$
Normal capillary oxygen concentration.

p_1
Implementation Name: `p1`
Units: dimensionless
Initial value: 12
Proton cost of the reaction reducing Cu_A.

p_3
Implementation Name: `p2`
Units: dimensionless
Initial value: 4
Proton cost of the reaction reducing a_3.

p_3
Implementation Name: `p3`
Units: dimensionless
Initial value: 4
Proton cost of the reaction reducing O_2.

$P_1 = \frac{P_a + P_v}{2}$
Implementation Name: `P_1`
Units: mm Hg
Initial value: $P_{1,n}$
Average pressure in the blood vessels.

$P_{1,n}$
Implementation Name: `P_1n`
Units: mm Hg
Initial value: $\frac{P_{a,n} + P_{v,n}}{2}$
Normal value for the average pressure in the blood vessels.

P_a
Implementation Name: `P_a`
Units: mmHg
Initial value: $P_{a,n}$
Mean arterial blood pressure.

$P_{a,n}$
Implementation Name: `P_an`
Units: mmHg
Initial value: 100
Normal arterial blood pressure.

P_{ic}
Implementation Name: `P_ic`
Units: mm Hg
Initial value: 9.5
Intracranial pressure.

P_{icn}
Implementation Name: `P_icn`
Units: mm Hg
Initial value: 9.5
Normal intracranial pressure.

P_v

Implementation Name: P_v
Units: mmHg
Initial value: $P_{v,n}$
Venous blood pressure.

$P_{v,n}$
Implementation Name: P_vn
Units: mmHg
Initial value: 4
Normal venous blood pressure.

Pa_{CO_2}
Implementation Name: Pa_CO2
Units: mmHg
Initial value: $Pa_{CO_{2,n}}$
Arterial partial pressure of carbon dioxide.

$Pa_{CO_{2,n}}$
Implementation Name: Pa_CO2n
Units: mmHg
Initial value: 40
Normal arterial partial pressure of carbon dioxide.

ϕ
Implementation Name: phi
Units: mM
Initial value: 0.036
Oxygen concentration at half-maximal saturation.

r_0
Implementation Name: r_0
Units: cm
Initial value: 0.0126
Radius in the elastic tension relationship.

R_{CO_2}
Implementation Name: R_autc
Units: dimensionless
Initial value: 2.2
Autoregulatory reactivity to carbon dioxide.

R_{O_2}
Implementation Name: R_auto
Units: dimensionless
Initial value: 1.5
Autoregulatory reactivity to oxygen.

R_{P_a}
Implementation Name: R_autp
Units: dimensionless
Initial value: 4
Autoregulatory reactivity to blood pressure.

R_u
Implementation Name: R_autu
Units: dimensionless
Initial value: 0.5
Autoregulatory reactivity to demand.

$R_{Hi,H}$
Implementation Name: R_Hi_H

Units: mM
Initial value: 9.565483
Proton buffering factor.

r_m
Implementation Name: `r_m`
Units: cm
Initial value: 0.027
Vessel radius at which muscular tension is maximal.

r_n
Implementation Name: `r_n`
Units: cm
Initial value: 0.0187
Normal blood vessel radius. Normal effective blood vessel radius.

r_t
Implementation Name: `r_t`
Units: cm
Initial value: 0.018
Radius in the muscular tension relationship.

$S_{a,O_2,n}$
Implementation Name: `SaO2_n`
Units: dimensionless
Initial value: 0.96
Normal arterial oxygen saturation.

S_{a,O_2}
Implementation Name: `SaO2sup`
Units: dimensionless
Initial value: $S_{a,O_2,n}$
Arterial oxygen saturation.

$S_{c,O_2,n}$
Implementation Name: `ScO2_n`
Units: dimensionless
Initial value: $\frac{S_{a,O_2,n} + S_{v,O_2,n}}{2}$
Normal capillary oxygen saturation.

σ_{coll}
Implementation Name: `sigma_coll`
Units: mm Hg
Initial value: 62.79
Pressure at which blood vessels collapse.

$\sigma_{e,0}$
Implementation Name: `sigma_e0`
Units: mm Hg
Initial value: 0.1425
Parameter in the elastic tension relationship.

$\sigma_{e,n}$
Implementation Name: `sigma_en`
Units: mm Hg
Initial value: $\sigma_{e,0} \left(\exp\left(\frac{K_\sigma \ (r_n - r_0)}{r_0} \right) - 1 \right) - \sigma_{coll}$
Normal elastic stress in blood vessel walls.

$S_{v,O_2,n}$

Implementation Name: `SvO2_n`
Units: dimensionless
Initial value: $\dfrac{HbO_{2,v,n}}{Hb_{tot,n}}$
Normal venous oxygen saturation.

t
Implementation Name: `t`
Units: s
Initial value: 0
Time over which the system evolves.

τ_{CO_2}
Implementation Name: `t_c`
Units: s
Initial value: 5
Filter time constant for stimulus effect of carbon dioxide.

$T_{e,n}$
Implementation Name: `T_en`
Units: mm Hg cm
Initial value: $\sigma_{e,n}\, h_n$
Normal elastic tension in the blood vessel walls.

$T_{max,0}$
Implementation Name: `T_max0`
Units: mm Hg cm
Initial value: $\dfrac{T_{max,n}}{1 + k_{aut}\, \mu_n}$
Maximal muscular tension under normal regulatory stimulus ($\mu = \mu_n$).

$T_{max,n}$
Implementation Name: `T_maxn`
Units: mm Hg cm
Initial value: $\dfrac{T_{m,n}}{\exp\left(-\mathrm{pow}\left(\mathrm{fabs}\left(\frac{r_n - r_m}{r_t - r_m}\right), n_m\right)\right)}$
Normal maximal muscular tension.

$T_{m,n}$
Implementation Name: `T_mn`
Units: mm Hg cm
Initial value: $(P_{1,n} - P_{icn})\; r_n - T_{e,n}$
Normal muscular tension in the blood vessel walls.

τ_{O_2}
Implementation Name: `t_o`
Units: s
Initial value: 20
Filter time constant for stimulus effect of capillary oxygen.

τ_{P_a}
Implementation Name: `t_p`
Units: s
Initial value: 5
Filter time constant for stimulus effect of blood pressure.

τ_u
Implementation Name: `t_u`
Units: s

Initial value: 0.5
Filter time constant for stimulus effect of demand.

u
Implementation Name: u
Units: dimensionless
Initial value: u_n
Parameter indicating metabolic demand.

u_n
Implementation Name: u_n
Units: dimensionless
Initial value: 1
Normal demand.

$v_{CO_2,n}$
Implementation Name: v_cn
Units: mmHg
Initial value: $Pa_{CO_{2,n}}$
Normal filtered carbon dioxide partial pressure. Normal filtered carbon dioxide partial pressure.

$v_{O_2,n}$
Implementation Name: v_on
Units: mM
Initial value: $O_{2,c,n}$
Normal filtered capillary oxygen concentration. Normal filtered capillary oxygen concentration.

$v_{P_a,n}$
Implementation Name: v_pn
Units: mmHg
Initial value: $P_{a,n}$
Normal filtered arterial blood pressure. Normal filtered blood pressure.

$v_{u,n}$
Implementation Name: v_un
Units: dimensionless
Initial value: u_n
Normal filtered demand. Normal filtered demand.

$VArat_n$
Implementation Name: VArat_n
Units: dimensionless
Initial value: 3
Normal volume ratio of veins to arteries in brain tissue.

$V_{a,n}$
Implementation Name: Vol_artn
Units: dimensionless
Initial value: $\frac{1}{1 + VArat_n}$
Normal relative arterial blood volume.

Vol_{mit}
Implementation Name: Vol_mit
Units: dimensionless
Initial value: 0.067
Fraction of brain tissue volume that is mitochondria.

V_v

Implementation Name: `Vol_ven`
Units: dimensionless
Initial value: $\frac{VArat_n}{1 + VArat_n}$
Relative venous blood volume.

$HbO_{2,a} = Hb_{tot}\, S_{a,O_2}$
Implementation Name: `XOa`
Units: mM
Initial value: $HbO_{2,a,n}$
Arterial concentration of oxygen bound to haemoglobin.

$HbO_{2,a,n}$
Implementation Name: `XOa_n`
Units: mM
Initial value: $Hb_{tot,n}\, S_{a,O_2,n}$
Normal arterial concentration of oxygen bound to haemoglobin.

$HbO_{2,v,n}$
Implementation Name: `XOv_n`
Units: mM
Initial value: $\frac{CBF_n\, HbO_{2,a,n} - J_{O_2,n}}{CBF_n}$
Normal venous concentration of oxygen bound to haemoglobin.

Hb_{tot}
Implementation Name: `Xtot`
Units: mM
Initial value: 9.1
Total concentration of haemoglobin O_2 binding sites in blood (4 times haemoglobin concentration).

$Hb_{tot,n}$
Implementation Name: `Xtot_n`
Units: mM
Initial value: 9.1
Normal total concentration of haemoglobin O_2 binding sites in blood (4 times haemoglobin concentration).

Z
Implementation Name: `Z`
Units: mV
Initial value: 59.028
Proportionality constant in calculation of driving forces due to concentration differences. Defined as RT/F, where F is Faraday's constant, R the ideal gas constant and T the absolute temperature.

10 B1M1

10.1 Overview

Simplified model combining blood flow variant B1 and metabolic variant M1.

- 9 differential state variables
- 3 algebraic state variables
- 22 intermediate variables
- 74 parameters
- 4 declared inputs
- 33 default outputs

10.2 Differential Equations

$$\frac{\mathrm{d}Cu_{A,o}}{\mathrm{d}t} = 4f_3 - 4f_1 \tag{10.1}$$

$$\frac{\mathrm{d}a_{3,r}}{\mathrm{d}t} = 4f_3 - 4f_3 \tag{10.2}$$

$$\frac{\mathrm{d}\psi}{\mathrm{d}t} = \frac{p_3\,f_3 + p_1\,f_1 + p_3\,f_3 - L}{C_{im}} \tag{10.3}$$

$$\frac{\mathrm{d}H^+}{\mathrm{d}t} = \frac{1}{R_{Hi}}\,L - \frac{p_3}{R_{Hi}}\,f_3 - \frac{p_1}{R_{Hi}}\,f_1 - \frac{p_3}{R_{Hi}}\,f_3 \tag{10.4}$$

$$\frac{\mathrm{d}O_2}{\mathrm{d}t} = \frac{1}{Vol_{mit}}\,J_{O_2} - f_3 \tag{10.5}$$

$$\frac{\mathrm{d}\nu_{CO_2}}{\mathrm{d}t} = \frac{1}{\tau_{CO_2}}\,\left(Pa_{CO_2} - \nu_{CO_2}\right) \tag{10.6}$$

$$\frac{\mathrm{d}\nu_{O_2}}{\mathrm{d}t} = \frac{1}{\tau_{O_2}}\,\left(O_{2,c} - \nu_{O_2}\right) \tag{10.7}$$

$$\frac{\mathrm{d}\nu_{P_a}}{\mathrm{d}t} = \frac{1}{\tau_{P_a}}\,\left(P_a - \nu_{P_a}\right) \tag{10.8}$$

$$\frac{\mathrm{d}\nu_u}{\mathrm{d}t} = \frac{1}{\tau_u}\,(u - \nu_u) \tag{10.9}$$

10.3 Algebraic Equations

$$\phi\left(\frac{S_{c,O_2}}{1 - S_{c,O_2}}\right)^{\frac{1}{n_h}} - O_{2,c} = 0 \tag{10.10}$$

$$\lambda_0 + \frac{\lambda_{P_a}}{P_a} + \lambda_\mu\,\mu + \frac{\lambda_{P_a,\mu}\,\mu}{P_a} - r = 0 \tag{10.11}$$

$$CBF\,(HbO_{2,a} - HbO_{2,v}) - J_{O_2} = 0 \tag{10.12}$$

10.4 Chemical Reactions

$$\xrightarrow{L} \frac{1}{R_{Hi}}\,\mathrm{H}^+ \tag{10.13}$$

$$\xrightarrow{J_{O_2}} \frac{1}{Vol_{mit}}\,\mathrm{O_2} \tag{10.14}$$

$$\frac{p_3}{R_{Hi}}\,\mathrm{H}^+ \xrightarrow{f_3} 4\,\mathrm{Cu_{A,o}} + 4\,\mathrm{a_{3,r}} \tag{10.15}$$

$$4\,\mathrm{Cu_{A,o}} + \frac{p_1}{R_{Hi}}\,\mathrm{H}^+ \xrightarrow{f_1} \tag{10.16}$$

$$\mathrm{O_2} + 4\,\mathrm{a_{3,r}} + \frac{p_3}{R_{Hi}}\,\mathrm{H}^+ \xrightarrow{f_3} \tag{10.17}$$

10.5 State Variables

$Cu_{A,o}$
: Implementation Name: `a`
Units: mM
Initial value: $Cu_{A,o,n}$
Concentration of oxidised cytochrome c oxidase.

$a_{3,r}$
: Implementation Name: `bred`
Units: mM
Initial value: $a_{3,r,n}$
Concentration of reduced cytochrome $\mathrm{a_3}$.

ψ
Implementation Name: `Dpsi`
Units: mV
Initial value: ψ_n
Mitochondrial inner membrane potential. Varies as charge (in the form of protons) is transferred across the membrane capacitance.

H^+
Implementation Name: `H`
Units: mM
Initial value: H_n^+
Mitochondrial proton concentration.

O_2
Implementation Name: `O2`
Units: mM
Initial value: $O_{2,n}$
Mitochondrial oxygen concentration.

$O_{2,c}$
Implementation Name: `O2c`
Units: mM
Initial value: $O_{2,c,n}$
Capillary oxygen concentration.

r
Implementation Name: `r`
Units: cm
Initial value: r_n
Typical blood vessel radius.

ν_{CO_2}
Implementation Name: `v_c`
Units: mmHg
Initial value: $\nu_{CO_2,n}$
Filtered carbon dioxide partial pressure.

ν_{O_2}
Implementation Name: `v_o`
Units: mM
Initial value: $\nu_{O_2,n}$
Filtered capillary oxygen concentration.

ν_{P_a}
Implementation Name: `v_p`
Units: mmHg
Initial value: $\nu_{P_a,n}$
Filtered arterial blood pressure.

ν_u
Implementation Name: `v_u`
Units: dimensionless
Initial value: $\nu_{u,n}$
Filtered demand.

$HbO_{2,v}$
Implementation Name: `XOv`
Units: mM
Initial value: $HbO_{2,v,n}$
Venous concentration of oxygen bound to haemoglobin.

10.6 Intermediate Variables

$CBF \ = G \ (P_a - P_v)$
Implementation Name: `CBF`
Units: $\mathrm{ml}_{blood}\ \mathrm{ml}_{brain}^{-1}\ \mathrm{s}^{-1}$
Initial value: CBF_n
Cerebral blood flow.

$\Delta oxCCO \ = \Delta oxCCO_{off} + 1000 Vol_{mit} \ (Cu_{A,o} - Cu_{A,o,n})$
Implementation Name: `CCO`
Units: uM
Initial value: 0
Cytochrome c oxidase signal measured by NIRS.

$CMRO_2 \ = f_3 \, Vol_{mit}$
Implementation Name: `CMRO2`
Units: $\mathrm{mM\ s}^{-1}$
Initial value: 0
Rate of cerebral oxygen metabolism.

$\Delta p \ = \psi - Z \ \left(4 + \log 10 \left(H^+\right)\right)$
Implementation Name: `Dp`
Units: mV
Initial value: 0
Proton motive force across the mitochondrial inner membrane.

$\eta \ = R_{P_a} \left(\frac{\nu_{P_a}}{\nu_{P_a,n}} - 1\right) + R_{O_2} \left(\frac{\nu_{O_2}}{\nu_{O_2,n}} - 1\right) + R_{CO_2} \left(1 - \frac{\nu_{CO_2}}{\nu_{CO_2,n}}\right) + R_u \left(1 - \frac{\nu_u}{\nu_{u,n}}\right)$
Implementation Name: `eta`
Units: dimensionless
Initial value: 0
Merged autoregulation stimulus.

$f_1 \ = \lambda_{f_1} + \lambda_{f_1,u} \log(u) + \lambda_{f_1,a} \log(Cu_{A,o})$
Implementation Name: `f1`
Units: $\mathrm{mM\ s}^{-1}$
Initial value: 0
Reaction rate for the reduction of Cu_A.

$f_3 \ = \lambda_{f_2} + \lambda_{f_2,b} \log(a_{3,r})$
Implementation Name: `f2`
Units: $\mathrm{mM\ s}^{-1}$
Initial value: 0
Reaction rate for the reduction of a_3.

$f_3 \ = \lambda_{f_3} + \lambda_{f_3,O} \log(O_2)$
Implementation Name: `f3`
Units: $\mathrm{mM\ s}^{-1}$
Initial value: 0
Reaction rate for the reduction of O_2.

$G \ = K_G \, r^4$
Implementation Name: `G`
Units: $\mathrm{ml}_{blood}\ \mathrm{ml}_{brain}^{-1}\ \mathrm{mmHg}^{-1}\ \mathrm{s}^{-1}$
Initial value: 0
Effective conductance of the whole blood flow compartment.

$HbO_2 \ = (V_a \, HbO_{2,a} + V_v \, HbO_{2,v}) \ blood_{hb}$
Implementation Name: `HbO2`
Units: uM

Initial value: 0
Oxygenated haemoglobin signal measured by NIRS.

$HbT \ = (V_a + V_v)\ Hb_{tot}\, blood_{hb}$
Implementation Name: `HbT`
Units: uM
Initial value: 0
Total haemoglobin signal measured by NIRS.

$HHb \ = HbT - HbO_2$
Implementation Name: `HHb`
Units: uM
Initial value: 0
Deoxygenated haemoglobin signal measured by NIRS.

$J_{O_2} \ = \text{fmin}\left(D_{O_2}\ (O_{2,c} - O_2)\,, CBF\, HbO_{2,a}\right)$
Implementation Name: `J_O2`
Units: mM s^{-1}
Initial value: 0
Oxygen flux from blood to tissue.

$L \ = \lambda_L + \lambda_{L,\theta}\,\theta + \lambda_{L,p}\,\Delta p$
Implementation Name: `L`
Units: mM s^{-1}
Initial value: 0
Rate of proton return to the mitochondrial matrix.

$\mu \ = \dfrac{k_{aut}\ (\exp(\eta) - 1)}{\exp(\eta) + 1}$
Implementation Name: `mu`
Units: dimensionless
Initial value: 0
Effective strength of the autoregulation reponse.

$R_{Hi} \ = \dfrac{R_{Hi,H}}{H^+}$
Implementation Name: `R_Hi`
Units: dimensionless
Initial value: 0
Relative mitochondrial volume for protons, taking into account buffering effect of pH.

$S_{c,O_2} \ = \dfrac{S_{a,O_2} + S_{v,O_2}}{2}$
Implementation Name: `ScO2`
Units: dimensionless
Initial value: $S_{c,O_2,n}$
Capillary oxygen saturation.

$S_{v,O_2} \ = \dfrac{HbO_{2,v}}{Hb_{tot}}$
Implementation Name: `SvO2`
Units: dimensionless
Initial value: $S_{v,O_2,n}$
Venous oxygen saturation.

$\theta \ = kCV\ (\Delta p + Z\log10(u) - 90)$
Implementation Name: `theta`
Units: dimensionless
Initial value: 0
Driving force Complex V.

$$TOI = \frac{100 HbO_2}{HbT}$$

Implementation Name: `TOI`
Units: dimensionless
Initial value: 0
Total oxygenation index.

$$V_{mca} = CBF\, CBFscale$$

Implementation Name: `Vmca`
Units: cm s^{-1}
Initial value: 0
Blood velocity in the middle cerebral artery.

$$V_a = V_{a,n} \left(\frac{r}{r_n}\right)^2$$

Implementation Name: `Vol_art`
Units: dimensionless
Initial value: 0
Relative arterial blood volume.

10.7 Parameters

$Cu_{A,o,n}$

Implementation Name: `a_n`
Units: mM
Initial value: 0.06567
Normal concentration of oxidised cytochrome c oxidase.

$blood_{hb}$

Implementation Name: `blood_hb`
Units: dimensionless
Initial value: 10.00
Factor to convert model haemoglobin concentration to instrumental units. Scales for blood fraction of brain volume, mM to μM, and number of binding sites.

$a_{3,r,n}$

Implementation Name: `bred_n`
Units: mM
Initial value: 0.001408
Normal concentration of reduced cytochrome a3.

C_{im}

Implementation Name: `C_im`
Units: mM mV^{-1}
Initial value: 0.00675
Capacitance of the mitochondrial inner membrane.

CBF_n

Implementation Name: `CBFn`
Units: $ml_{blood}\, ml_{brain}^{-1}\, s^{-1}$
Initial value: 0.0125
Normal cerebral blood flow.

$CBFscale$

Implementation Name: `CBFscale`
Units: cm
Initial value: 5000
Scale constant relating blood flow to arterial velocity.

$\Delta oxCCO_{off}$
Implementation Name: `CCO_offset`
Units: uM
Initial value: 0
Signal offset for the NIRS CCO measurement.

$CMRO_{2,n}$
Implementation Name: `CMRO2_n`
Units: mM s^{-1}
Initial value: 0.034
Normal metabolic rate of oxygen consumption.

D_{O_2}
Implementation Name: `D_O2`
Units: s^{-1}
Initial value: $\frac{J_{O_{2,n}}}{O_{2,c,n} - O_{2,n}}$
Diffusion rate for oxygen between capillaries and mitochondria.

ψ_n
Implementation Name: `Dpsi_n`
Units: mV
Initial value: 145
Normal mitochondrial inner membrane potential.

λ_{f_1}
Implementation Name: `f1_0`
Units: mM s^{-1}
Initial value: 1.490
Fitted intercept for the linear model for f_1.

$\lambda_{f_1,a}$
Implementation Name: `f1_a`
Units: mM s^{-1}
Initial value: 0.3609
Fitted linear dependence of f_1 on logarithm of $Cu_{A,ox}$.

$\lambda_{f_1,u}$
Implementation Name: `f1_u`
Units: mM s^{-1}
Initial value: 0.06985
Fitted linear dependence of f_1 on logarithm of demand.

λ_{f_2}
Implementation Name: `f2_0`
Units: mM s^{-1}
Initial value: 0.1473
Fitted intercept for the linear model for f_2.

$\lambda_{f_2,b}$
Implementation Name: `f2_bred`
Units: mM s^{-1}
Initial value: -0.05484
Fitted linear dependence of f_2 on logarithm of $a_{3,red}$.

λ_{f_3}
Implementation Name: `f3_0`
Units: mM s^{-1}
Initial value: 0.6324
Fitted intercept for the linear model for f_3.

$\lambda_{f_3,O}$
Implementation Name: `f3_O2`
Units: mM s^{-1}
Initial value: 0.03352
Fitted linear dependence of f_3 on logarithm of O_2.

G_n
Implementation Name: `Gn`
Units: ml_{blood} ml_{brain}^{-1} $mmHg^{-1}$ s^{-1}
Initial value: $\frac{CBF_n}{P_{a,n} - P_{v,n}}$
Normal blood vessel conductance.

H_n^+
Implementation Name: `H_n`
Units: mM
Initial value: 0.00003981
Normal mitochondrial proton concentration.

$J_{O_{2,n}}$
Implementation Name: `J_O2n`
Units: mM s^{-1}
Initial value: $CMRO_{2,n}$
Normal oxygen flux from blood to tissue.

k_{aut}
Implementation Name: `k_aut`
Units: dimensionless
Initial value: 1
Overall functioning of autoregulatory response.

K_G
Implementation Name: `K_G`
Units: ml_{blood} ml_{brain}^{-1} $mmHg^{-1}$ s^{-1} cm^{-4}
Initial value: $\frac{G_n}{\text{pow}\,(r_n, 4)}$
Proportionality constant in Poiseuille relation for conductance.

kCV
Implementation Name: `kCV`
Units: mV^{-1}
Initial value: 0.02047339
Factor relating the Complex V driving force to the membrane potential and demand.

λ_L
Implementation Name: `L_0`
Units: mM s^{-1}
Initial value: -15.339464
Fitted intercept for the linear model for L.

$\lambda_{L,p}$
Implementation Name: `L_Dp`
Units: mM s^{-1} mV^{-1}
Initial value: 0.097097
Fitted linear dependence of L on Δp.

$\lambda_{L,\theta}$
Implementation Name: `L_th`
Units: mM s^{-1}

Initial value: 5.665904
Fitted linear dependence of L on θ.

λ_0
Implementation Name: `lam_0`
Units: cm
Initial value: 0.02507
Intercept of the fitted linear model for blood vessel radius.

λ_μ
Implementation Name: `lam_mu`
Units: cm
Initial value: -0.0004422
Fitted linear dependence of blood vessel radius on autoregulatory stimuli.

λ_{P_a}
Implementation Name: `lam_p`
Units: cm mmHg
Initial value: -0.6327
Fitted linear dependence of blood vessel radius on reciprocal of blood pressure.

$\lambda_{P_a,\mu}$
Implementation Name: `lam_p_mu`
Units: cm mmHg
Initial value: -0.5286
Fitted joint dependence of blood vessel radius on autoregulatory stimuli and reciprocal of blood pressure.

n_h
Implementation Name: `n_h`
Units: dimensionless
Initial value: 2.5
Hill coefficient for oxygen dissociation from haemoglobin.

$O_{2,n}$
Implementation Name: `O2_n`
Units: mM
Initial value: 0.024
Normal mitochondrial oxygen concentration.

$O_{2,c,n}$
Implementation Name: `O2c_n`
Units: mM
Initial value: $\phi \,\mathrm{pow}\left(\frac{S_{c,O_2,n}}{1 - S_{c,O_2,n}}, \frac{1}{n_h}\right)$
Normal capillary oxygen concentration.

p_1
Implementation Name: `p1`
Units: dimensionless
Initial value: 12
Proton cost of the reaction reducing Cu_A.

p_3
Implementation Name: `p2`
Units: dimensionless
Initial value: 4
Proton cost of the reaction reducing a_3.

p_3
Implementation Name: `p3`

Units: dimensionless
Initial value: 4
Proton cost of the reaction reducing O_2.

P_a
Implementation Name: P_a
Units: mmHg
Initial value: $P_{a,n}$
Mean arterial blood pressure.

$P_{a,n}$
Implementation Name: P_an
Units: mmHg
Initial value: 100
Normal arterial blood pressure.

P_v
Implementation Name: P_v
Units: mmHg
Initial value: $P_{v,n}$
Venous blood pressure.

$P_{v,n}$
Implementation Name: P_vn
Units: mmHg
Initial value: 4
Normal venous blood pressure.

Pa_{CO_2}
Implementation Name: Pa_CO2
Units: mmHg
Initial value: $Pa_{CO_{2,n}}$
Arterial partial pressure of carbon dioxide.

$Pa_{CO_{2,n}}$
Implementation Name: Pa_CO2n
Units: mmHg
Initial value: 40
Normal arterial partial pressure of carbon dioxide.

ϕ
Implementation Name: phi
Units: mM
Initial value: 0.036
Oxygen concentration at half-maximal saturation.

R_{CO_2}
Implementation Name: R_autc
Units: dimensionless
Initial value: 2.2
Autoregulatory reactivity to carbon dioxide.

R_{O_2}
Implementation Name: R_auto
Units: dimensionless
Initial value: 1.5
Autoregulatory reactivity to oxygen.

R_{P_a}
Implementation Name: R_autp
Units: dimensionless

Initial value: 4
Autoregulatory reactivity to blood pressure.

R_u
Implementation Name: R_autu
Units: dimensionless
Initial value: 0.5
Autoregulatory reactivity to demand.

$R_{Hi,H}$
Implementation Name: R_Hi_H
Units: mM
Initial value: 9.565483
Proton buffering factor.

r_n
Implementation Name: r_n
Units: cm
Initial value: 0.0187
Normal blood vessel radius. Normal effective blood vessel radius.

$S_{a,O_2,n}$
Implementation Name: SaO2_n
Units: dimensionless
Initial value: 0.96
Normal arterial oxygen saturation.

S_{a,O_2}
Implementation Name: SaO2sup
Units: dimensionless
Initial value: $S_{a,O_2,n}$
Arterial oxygen saturation.

$S_{c,O_2,n}$
Implementation Name: ScO2_n
Units: dimensionless
Initial value: $\frac{S_{a,O_2,n} + S_{v,O_2,n}}{2}$
Normal capillary oxygen saturation.

$S_{v,O_2,n}$
Implementation Name: SvO2_n
Units: dimensionless
Initial value: $\frac{HbO_{2,v,n}}{Hb_{tot,n}}$
Normal venous oxygen saturation.

t
Implementation Name: t
Units: s
Initial value: 0
Time over which the system evolves.

τ_{CO_2}
Implementation Name: t_c
Units: s
Initial value: 5
Filter time constant for stimulus effect of carbon dioxide.

τ_{O_2}
Implementation Name: t_o
Units: s

Initial value: 20
Filter time constant for stimulus effect of capillary oxygen.

τ_{P_a}
Implementation Name: t_p
Units: s
Initial value: 5
Filter time constant for stimulus effect of blood pressure.

τ_u
Implementation Name: t_u
Units: s
Initial value: 0.5
Filter time constant for stimulus effect of demand.

u
Implementation Name: u
Units: dimensionless
Initial value: u_n
Parameter indicating metabolic demand.

u_n
Implementation Name: u_n
Units: dimensionless
Initial value: 1
Normal demand.

$v_{CO_2,n}$
Implementation Name: v_cn
Units: mmHg
Initial value: $Pa_{CO_{2,n}}$
Normal filtered carbon dioxide partial pressure. Normal filtered carbon dioxide partial pressure.

$v_{O_2,n}$
Implementation Name: v_on
Units: mM
Initial value: $O_{2,c,n}$
Normal filtered capillary oxygen concentration. Normal filtered capillary oxygen concentration.

$v_{P_a,n}$
Implementation Name: v_pn
Units: mmHg
Initial value: $P_{a,n}$
Normal filtered arterial blood pressure. Normal filtered blood pressure.

$v_{u,n}$
Implementation Name: v_un
Units: dimensionless
Initial value: u_n
Normal filtered demand. Normal filtered demand.

$VArat_n$
Implementation Name: VArat_n
Units: dimensionless
Initial value: 3
Normal volume ratio of veins to arteries in brain tissue.

$V_{a,n}$
Implementation Name: Vol_artn

Units: dimensionless
Initial value: $\frac{1}{1 + VArat_n}$
Normal relative arterial blood volume.

Vol_{mit}
Implementation Name: `Vol_mit`
Units: dimensionless
Initial value: 0.067
Fraction of brain tissue volume that is mitochondria.

V_v
Implementation Name: `Vol_ven`
Units: dimensionless
Initial value: $\frac{VArat_n}{1 + VArat_n}$
Relative venous blood volume.

$HbO_{2,a} \; = Hb_{tot}\, S_{a,O_2}$
Implementation Name: `XOa`
Units: mM
Initial value: $HbO_{2,a,n}$
Arterial concentration of oxygen bound to haemoglobin.

$HbO_{2,a,n}$
Implementation Name: `XOa_n`
Units: mM
Initial value: $Hb_{tot,n}\, S_{a,O_2,n}$
Normal arterial concentration of oxygen bound to haemoglobin.

$HbO_{2,v,n}$
Implementation Name: `XOv_n`
Units: mM
Initial value: $\frac{CBF_n\, HbO_{2,a,n} - J_{O_{2,n}}}{CBF_n}$
Normal venous concentration of oxygen bound to haemoglobin.

Hb_{tot}
Implementation Name: `Xtot`
Units: mM
Initial value: 9.1
Total concentration of haemoglobin O_2 binding sites in blood (4 times haemoglobin concentration).

$Hb_{tot,n}$
Implementation Name: `Xtot_n`
Units: mM
Initial value: 9.1
Normal total concentration of haemoglobin O_2 binding sites in blood (4 times haemoglobin concentration).

Z
Implementation Name: `Z`
Units: mV
Initial value: 59.028
Proportionality constant in calculation of driving forces due to concentration differences. Defined as RT/F, where F is Faraday's constant, R the ideal gas constant and T the absolute temperature.

11 B1M2

11.1 Overview

Simplified model combining blood flow variant B1 and metabolic variant M2.

- 9 differential state variables
- 3 algebraic state variables
- 22 intermediate variables
- 73 parameters
- 4 declared inputs
- 33 default outputs

11.2 Differential Equations

$$\frac{\mathrm{d}Cu_{A,o}}{\mathrm{d}t} = 4f_3 - 4f_1 \tag{11.1}$$

$$\frac{\mathrm{d}a_{3,r}}{\mathrm{d}t} = 4f_3 - 4f_3 \tag{11.2}$$

$$\frac{\mathrm{d}\psi}{\mathrm{d}t} = \frac{p_3\, f_3 + p_1\, f_1 + p_3\, f_3 - L}{C_{im}} \tag{11.3}$$

$$\frac{\mathrm{d}H^+}{\mathrm{d}t} = \frac{1}{R_{Hi}}\, L - \frac{p_3}{R_{Hi}}\, f_3 - \frac{p_1}{R_{Hi}}\, f_1 - \frac{p_3}{R_{Hi}}\, f_3 \tag{11.4}$$

$$\frac{\mathrm{d}O_2}{\mathrm{d}t} = \frac{1}{Vol_{mit}}\, J_{O_2} - f_3 \tag{11.5}$$

$$\frac{\mathrm{d}v_{CO_2}}{\mathrm{d}t} = \frac{1}{\tau_{CO_2}}\, \left(Pa_{CO_2} - v_{CO_2}\right) \tag{11.6}$$

$$\frac{\mathrm{d}v_{O_2}}{\mathrm{d}t} = \frac{1}{\tau_{O_2}}\, \left(O_{2,c} - v_{O_2}\right) \tag{11.7}$$

$$\frac{\mathrm{d}v_{P_a}}{\mathrm{d}t} = \frac{1}{\tau_{P_a}}\, \left(P_a - v_{P_a}\right) \tag{11.8}$$

$$\frac{\mathrm{d}\nu_u}{\mathrm{d}t} = \frac{1}{\tau_u}\,(u - \nu_u) \qquad (11.9)$$

11.3 Algebraic Equations

$$\phi\left(\frac{S_{c,O_2}}{1 - S_{c,O_2}}\right)^{\frac{1}{n_h}} - O_{2,c} = 0 \qquad (11.10)$$

$$\lambda_0 + \frac{\lambda_{P_a}}{P_a} + \lambda_\mu\,\mu + \frac{\lambda_{P_a,\mu}\,\mu}{P_a} - r = 0 \qquad (11.11)$$

$$CBF\,(HbO_{2,a} - HbO_{2,v}) - J_{O_2} = 0 \qquad (11.12)$$

11.4 Chemical Reactions

$$\xrightarrow{L} \frac{1}{R_{Hi}}\,\mathrm{H}^+ \qquad (11.13)$$

$$\xrightarrow{J_{O_2}} \frac{1}{Vol_{mit}}\,\mathrm{O}_2 \qquad (11.14)$$

$$\frac{p_3}{R_{Hi}}\,\mathrm{H}^+ \xrightarrow{f_3} 4\,\mathrm{Cu}_{\mathrm{A,o}} + 4\,\mathrm{a}_{3,\mathrm{r}} \qquad (11.15)$$

$$4\,\mathrm{Cu}_{\mathrm{A,o}} + \frac{p_1}{R_{Hi}}\,\mathrm{H}^+ \xrightarrow{f_1} \qquad (11.16)$$

$$\mathrm{O}_2 + 4\,\mathrm{a}_{3,\mathrm{r}} + \frac{p_3}{R_{Hi}}\,\mathrm{H}^+ \xrightarrow{f_3} \qquad (11.17)$$

11.5 State Variables

$Cu_{A,o}$
Implementation Name: `a`
Units: mM
Initial value: $Cu_{A,o,n}$
Concentration of oxidised cytochrome c oxidase.

$a_{3,r}$
Implementation Name: `bred`
Units: mM
Initial value: $a_{3,r,n}$
Concentration of reduced cytochrome a_3.

ψ
Implementation Name: `Dpsi`
Units: mV
Initial value: ψ_n
Mitochondrial inner membrane potential. Varies as charge (in the form of protons) is transferred across the membrane capacitance.

H^+
Implementation Name: `H`
Units: mM
Initial value: H_n^+
Mitochondrial proton concentration.

O_2
Implementation Name: `O2`
Units: mM
Initial value: $O_{2,n}$
Mitochondrial oxygen concentration.

$O_{2,c}$
Implementation Name: `O2c`
Units: mM
Initial value: $O_{2,c,n}$
Capillary oxygen concentration.

r
Implementation Name: `r`
Units: cm
Initial value: r_n
Typical blood vessel radius.

ν_{CO_2}
Implementation Name: `v_c`
Units: mmHg
Initial value: $\nu_{CO_2,n}$
Filtered carbon dioxide partial pressure.

ν_{O_2}
Implementation Name: `v_o`
Units: mM
Initial value: $\nu_{O_2,n}$
Filtered capillary oxygen concentration.

ν_{P_a}
Implementation Name: `v_p`
Units: mmHg
Initial value: $\nu_{P_a,n}$
Filtered arterial blood pressure.

ν_u
Implementation Name: `v_u`
Units: dimensionless
Initial value: $\nu_{u,n}$
Filtered demand.

$HbO_{2,v}$
Implementation Name: `XOv`
Units: mM
Initial value: $HbO_{2,v,n}$
Venous concentration of oxygen bound to haemoglobin.

11.6 Intermediate Variables

$CBF = G\ (P_a - P_v)$
Implementation Name: `CBF`
Units: $ml_{blood}\ ml_{brain}^{-1}\ s^{-1}$
Initial value: CBF_n
Cerebral blood flow.

$\Delta oxCCO = \Delta oxCCO_{off} + 1000 Vol_{mit}\ (Cu_{A,o} - Cu_{A,o,n})$
Implementation Name: `CCO`
Units: uM
Initial value: 0
Cytochrome c oxidase signal measured by NIRS.

$CMRO_2 = f_3\ Vol_{mit}$
Implementation Name: `CMRO2`
Units: mM s^{-1}
Initial value: 0
Rate of cerebral oxygen metabolism.

$\Delta p = \psi - Z\ (4 + \log 10\ (H^+))$
Implementation Name: `Dp`
Units: mV
Initial value: 0
Proton motive force across the mitochondrial inner membrane.

$\eta = R_{P_a}\left(\frac{\nu_{P_a}}{\nu_{P_a,n}} - 1\right) + R_{O_2}\left(\frac{\nu_{O_2}}{\nu_{O_2,n}} - 1\right) + R_{CO_2}\left(1 - \frac{\nu_{CO_2}}{\nu_{CO_2,n}}\right) + R_u\left(1 - \frac{\nu_u}{\nu_{u,n}}\right)$
Implementation Name: `eta`
Units: dimensionless
Initial value: 0
Merged autoregulation stimulus.

$f_1 = \lambda_{f_1} + \lambda_{f_1,a} \log\ (Cu_{A,o})$
Implementation Name: `f1`
Units: mM s^{-1}
Initial value: 0
Reaction rate for the reduction of Cu_A.

$f_3 = \lambda_{f_2} + \lambda_{f_2,b} \log\ (a_{3,r})$
Implementation Name: `f2`
Units: mM s^{-1}
Initial value: 0
Reaction rate for the reduction of a_3.

$f_3 = \lambda_{f_3} + \lambda_{f_3,O} \log\ (O_2)$
Implementation Name: `f3`
Units: mM s^{-1}
Initial value: 0
Reaction rate for the reduction of O_2.

$G = K_G\ r^4$
Implementation Name: `G`
Units: $ml_{blood}\ ml_{brain}^{-1}\ mmHg^{-1}\ s^{-1}$
Initial value: 0
Effective conductance of the whole blood flow compartment.

$HbO_2 = (V_a\ HbO_{2,a} + V_v\ HbO_{2,v})\ blood_{hb}$
Implementation Name: `HbO2`
Units: uM

Initial value: 0
Oxygenated haemoglobin signal measured by NIRS.

$HbT = (V_a + V_v)\ Hb_{tot}\, blood_{hb}$
Implementation Name: `HbT`
Units: uM
Initial value: 0
Total haemoglobin signal measured by NIRS.

$HHb = HbT - HbO_2$
Implementation Name: `HHb`
Units: uM
Initial value: 0
Deoxygenated haemoglobin signal measured by NIRS.

$J_{O_2} = \mathrm{fmin}\left(D_{O_2}\ (O_{2,c} - O_2)\,, CBF\, HbO_{2,a}\right)$
Implementation Name: `J_O2`
Units: mM s^{-1}
Initial value: 0
Oxygen flux from blood to tissue.

$L = \lambda_L + \lambda_{L,\theta}\, \theta + \lambda_{L,p}\, \Delta p$
Implementation Name: `L`
Units: mM s^{-1}
Initial value: 0
Rate of proton return to the mitochondrial matrix.

$\mu = \dfrac{k_{aut}\ (\exp(\eta) - 1)}{\exp(\eta) + 1}$
Implementation Name: `mu`
Units: dimensionless
Initial value: 0
Effective strength of the autoregulation reponse.

$R_{Hi} = \dfrac{R_{Hi,H}}{H^+}$
Implementation Name: `R_Hi`
Units: dimensionless
Initial value: 0
Relative mitochondrial volume for protons, taking into account buffering effect of pH.

$S_{c,O_2} = \dfrac{S_{a,O_2} + S_{v,O_2}}{2}$
Implementation Name: `ScO2`
Units: dimensionless
Initial value: $S_{c,O_2,n}$
Capillary oxygen saturation.

$S_{v,O_2} = \dfrac{HbO_{2,v}}{Hb_{tot}}$
Implementation Name: `SvO2`
Units: dimensionless
Initial value: $S_{v,O_2,n}$
Venous oxygen saturation.

$\theta = kCV\ (\Delta p + Z \log10(u) - 90)$
Implementation Name: `theta`
Units: dimensionless
Initial value: 0
Driving force Complex V.

$TOI = \frac{100HbO_2}{HbT}$
Implementation Name: `TOI`
Units: dimensionless
Initial value: 0
Total oxygenation index.

$V_{mca} = CBF\,CBFscale$
Implementation Name: `Vmca`
Units: cm s^{-1}
Initial value: 0
Blood velocity in the middle cerebral artery.

$V_a = V_{a,n}\left(\frac{r}{r_n}\right)^2$
Implementation Name: `Vol_art`
Units: dimensionless
Initial value: 0
Relative arterial blood volume.

11.7 Parameters

$Cu_{A,o,n}$
Implementation Name: `a_n`
Units: mM
Initial value: 0.06567
Normal concentration of oxidised cytochrome c oxidase.

$blood_{hb}$
Implementation Name: `blood_hb`
Units: dimensionless
Initial value: 10.00
Factor to convert model haemoglobin concentration to instrumental units. Scales for blood fraction of brain volume, mM to μM, and number of binding sites.

$a_{3,r,n}$
Implementation Name: `bred_n`
Units: mM
Initial value: 0.001408
Normal concentration of reduced cytochrome a3.

C_{im}
Implementation Name: `C_im`
Units: mM mV^{-1}
Initial value: 0.00675
Capacitance of the mitochondrial inner membrane.

CBF_n
Implementation Name: `CBFn`
Units: $ml_{blood}\ ml_{brain}^{-1}\ s^{-1}$
Initial value: 0.0125
Normal cerebral blood flow.

$CBFscale$
Implementation Name: `CBFscale`
Units: cm
Initial value: 5000
Scale constant relating blood flow to arterial velocity.

$\Delta oxCCO_{off}$
Implementation Name: `CCO_offset`
Units: uM
Initial value: 0
Signal offset for the NIRS CCO measurement.

$CMRO_{2,n}$
Implementation Name: `CMRO2_n`
Units: mM s^{-1}
Initial value: 0.034
Normal metabolic rate of oxygen consumption.

D_{O_2}
Implementation Name: `D_O2`
Units: s^{-1}
Initial value: $\frac{J_{O_{2,n}}}{O_{2,c,n} - O_{2,n}}$
Diffusion rate for oxygen between capillaries and mitochondria.

ψ_n
Implementation Name: `Dpsi_n`
Units: mV
Initial value: 145
Normal mitochondrial inner membrane potential.

λ_{f_1}
Implementation Name: `f1_0`
Units: mM s^{-1}
Initial value: 1.504
Fitted intercept for the linear model for f_1.

$\lambda_{f_1,a}$
Implementation Name: `f1_a`
Units: mM s^{-1}
Initial value: 0.3658
Fitted linear dependence of f_1 on logarithm of $\mathrm{Cu}_{A,ox}$.

λ_{f_2}
Implementation Name: `f2_0`
Units: mM s^{-1}
Initial value: 0.1473
Fitted intercept for the linear model for f_2.

$\lambda_{f_2,b}$
Implementation Name: `f2_bred`
Units: mM s^{-1}
Initial value: -0.05484
Fitted linear dependence of f_2 on logarithm of $\mathrm{a}_{3,red}$.

λ_{f_3}
Implementation Name: `f3_0`
Units: mM s^{-1}
Initial value: 0.6324
Fitted intercept for the linear model for f_3.

$\lambda_{f_3,O}$
Implementation Name: `f3_O2`
Units: mM s^{-1}
Initial value: 0.03352
Fitted linear dependence of f_3 on logarithm of O_2.

G_n

Implementation Name: `Gn`
Units: $ml_{blood}\ ml_{brain}^{-1}\ mmHg^{-1}\ s^{-1}$
Initial value: $\frac{CBF_n}{P_{a,n} - P_{v,n}}$
Normal blood vessel conductance.

H_n^+

Implementation Name: `H_n`
Units: mM
Initial value: 0.00003981
Normal mitochondrial proton concentration.

$J_{O_{2,n}}$

Implementation Name: `J_O2n`
Units: mM s^{-1}
Initial value: $CMRO_{2,n}$
Normal oxygen flux from blood to tissue.

k_{aut}

Implementation Name: `k_aut`
Units: dimensionless
Initial value: 1
Overall functioning of autoregulatory response.

K_G

Implementation Name: `K_G`
Units: $ml_{blood}\ ml_{brain}^{-1}\ mmHg^{-1}\ s^{-1}\ cm^{-4}$
Initial value: $\frac{G_n}{\text{pow}\,(r_n, 4)}$
Proportionality constant in Poiseuille relation for conductance.

kCV

Implementation Name: `kCV`
Units: mV^{-1}
Initial value: 0.02047339
Factor relating the Complex V driving force to the membrane potential and demand.

λ_L

Implementation Name: `L_0`
Units: mM s^{-1}
Initial value: -15.339464
Fitted intercept for the linear model for L.

$\lambda_{L,p}$

Implementation Name: `L_Dp`
Units: mM s^{-1} mV^{-1}
Initial value: 0.097097
Fitted linear dependence of L on Δp.

$\lambda_{L,\theta}$

Implementation Name: `L_th`
Units: mM s^{-1}
Initial value: 5.665904
Fitted linear dependence of L on θ.

λ_0

Implementation Name: `lam_0`
Units: cm

Initial value: 0.02507
Intercept of the fitted linear model for blood vessel radius.

λ_{μ}

Implementation Name: `lam_mu`
Units: cm
Initial value: -0.0004422
Fitted linear dependence of blood vessel radius on autoregulatory stimuli.

λ_{P_a}

Implementation Name: `lam_p`
Units: cm mmHg
Initial value: -0.6327
Fitted linear dependence of blood vessel radius on reciprocal of blood pressure.

$\lambda_{P_a,\mu}$

Implementation Name: `lam_p_mu`
Units: cm mmHg
Initial value: -0.5286
Fitted joint dependence of blood vessel radius on autoregulatory stimuli and reciprocal of blood pressure.

n_h

Implementation Name: `n_h`
Units: dimensionless
Initial value: 2.5
Hill coefficient for oxygen dissociation from haemoglobin.

$O_{2,n}$

Implementation Name: `O2_n`
Units: mM
Initial value: 0.024
Normal mitochondrial oxygen concentration.

$O_{2,c,n}$

Implementation Name: `O2c_n`
Units: mM
Initial value: $\phi \, \mathrm{pow}\left(\frac{S_{c,O_2,n}}{1 - S_{c,O_2,n}}, \frac{1}{n_h}\right)$
Normal capillary oxygen concentration.

p_1

Implementation Name: `p1`
Units: dimensionless
Initial value: 12
Proton cost of the reaction reducing Cu_A.

p_3

Implementation Name: `p2`
Units: dimensionless
Initial value: 4
Proton cost of the reaction reducing a_3.

p_3

Implementation Name: `p3`
Units: dimensionless
Initial value: 4
Proton cost of the reaction reducing O_2.

P_a

Implementation Name: `P_a`

Units: mmHg
Initial value: $P_{a,n}$
Mean arterial blood pressure.

$P_{a,n}$
Implementation Name: P_an
Units: mmHg
Initial value: 100
Normal arterial blood pressure.

P_v
Implementation Name: P_v
Units: mmHg
Initial value: $P_{v,n}$
Venous blood pressure.

$P_{v,n}$
Implementation Name: P_vn
Units: mmHg
Initial value: 4
Normal venous blood pressure.

Pa_{CO_2}
Implementation Name: Pa_CO2
Units: mmHg
Initial value: $Pa_{CO_{2,n}}$
Arterial partial pressure of carbon dioxide.

$Pa_{CO_{2,n}}$
Implementation Name: Pa_CO2n
Units: mmHg
Initial value: 40
Normal arterial partial pressure of carbon dioxide.

ϕ
Implementation Name: phi
Units: mM
Initial value: 0.036
Oxygen concentration at half-maximal saturation.

R_{CO_2}
Implementation Name: R_autc
Units: dimensionless
Initial value: 2.2
Autoregulatory reactivity to carbon dioxide.

R_{O_2}
Implementation Name: R_auto
Units: dimensionless
Initial value: 1.5
Autoregulatory reactivity to oxygen.

R_{P_a}
Implementation Name: R_autp
Units: dimensionless
Initial value: 4
Autoregulatory reactivity to blood pressure.

R_u
Implementation Name: R_autu
Units: dimensionless

Initial value: 0.5
Autoregulatory reactivity to demand.

$R_{Hi,H}$
Implementation Name: R_Hi_H
Units: mM
Initial value: 9.565483
Proton buffering factor.

r_n
Implementation Name: r_n
Units: cm
Initial value: 0.0187
Normal blood vessel radius. Normal effective blood vessel radius.

$S_{a,O_2,n}$
Implementation Name: SaO2_n
Units: dimensionless
Initial value: 0.96
Normal arterial oxygen saturation.

S_{a,O_2}
Implementation Name: SaO2sup
Units: dimensionless
Initial value: $S_{a,O_2,n}$
Arterial oxygen saturation.

$S_{c,O_2,n}$
Implementation Name: ScO2_n
Units: dimensionless
Initial value: $\frac{S_{a,O_2,n} + S_{v,O_2,n}}{2}$
Normal capillary oxygen saturation.

$S_{v,O_2,n}$
Implementation Name: SvO2_n
Units: dimensionless
Initial value: $\frac{HbO_{2,v,n}}{Hb_{tot,n}}$
Normal venous oxygen saturation.

t
Implementation Name: t
Units: s
Initial value: 0
Time over which the system evolves.

τ_{CO_2}
Implementation Name: t_c
Units: s
Initial value: 5
Filter time constant for stimulus effect of carbon dioxide.

τ_{O_2}
Implementation Name: t_o
Units: s
Initial value: 20
Filter time constant for stimulus effect of capillary oxygen.

τ_{P_a}
Implementation Name: t_p
Units: s

Initial value: 5
Filter time constant for stimulus effect of blood pressure.

τ_u
Implementation Name: t_u
Units: s
Initial value: 0.5
Filter time constant for stimulus effect of demand.

u
Implementation Name: u
Units: dimensionless
Initial value: u_n
Parameter indicating metabolic demand.

u_n
Implementation Name: u_n
Units: dimensionless
Initial value: 1
Normal demand.

$v_{CO_2,n}$
Implementation Name: v_cn
Units: mmHg
Initial value: $Pa_{CO_{2,n}}$
Normal filtered carbon dioxide partial pressure. Normal filtered carbon dioxide partial pressure.

$v_{O_2,n}$
Implementation Name: v_on
Units: mM
Initial value: $O_{2,c,n}$
Normal filtered capillary oxygen concentration. Normal filtered capillary oxygen concentration.

$v_{P_a,n}$
Implementation Name: v_pn
Units: mmHg
Initial value: $P_{a,n}$
Normal filtered arterial blood pressure. Normal filtered blood pressure.

$v_{u,n}$
Implementation Name: v_un
Units: dimensionless
Initial value: u_n
Normal filtered demand. Normal filtered demand.

$VArat_n$
Implementation Name: VArat_n
Units: dimensionless
Initial value: 3
Normal volume ratio of veins to arteries in brain tissue.

$V_{a,n}$
Implementation Name: Vol_artn
Units: dimensionless
Initial value: $\dfrac{1}{1 + VArat_n}$
Normal relative arterial blood volume.

Vol_{mit}

Implementation Name: `Vol_mit`
Units: dimensionless
Initial value: 0.067
Fraction of brain tissue volume that is mitochondria.

V_v

Implementation Name: `Vol_ven`
Units: dimensionless
Initial value: $\frac{VArat_n}{1 + VArat_n}$
Relative venous blood volume.

$HbO_{2,a} = Hb_{tot}\, S_{a,O_2}$

Implementation Name: `XOa`
Units: mM
Initial value: $HbO_{2,a,n}$
Arterial concentration of oxygen bound to haemoglobin.

$HbO_{2,a,n}$

Implementation Name: `XOa_n`
Units: mM
Initial value: $Hb_{tot,n}\, S_{a,O_2,n}$
Normal arterial concentration of oxygen bound to haemoglobin.

$HbO_{2,v,n}$

Implementation Name: `XOv_n`
Units: mM
Initial value: $\frac{CBF_n\, HbO_{2,a,n} - J_{O_{2,n}}}{CBF_n}$
Normal venous concentration of oxygen bound to haemoglobin.

Hb_{tot}

Implementation Name: `Xtot`
Units: mM
Initial value: 9.1
Total concentration of haemoglobin O_2 binding sites in blood (4 times haemoglobin concentration).

$Hb_{tot,n}$

Implementation Name: `Xtot_n`
Units: mM
Initial value: 9.1
Normal total concentration of haemoglobin O_2 binding sites in blood (4 times haemoglobin concentration).

Z

Implementation Name: `Z`
Units: mV
Initial value: 59.028
Proportionality constant in calculation of driving forces due to concentration differences. Defined as RT/F, where F is Faraday's constant, R the ideal gas constant and T the absolute temperature.

12 B2M1

12.1 Overview

Simplified model combining blood flow variant B2 and metabolic variant M1.

- 9 differential state variables
- 3 algebraic state variables
- 22 intermediate variables
- 73 parameters
- 4 declared inputs
- 33 default outputs

12.2 Differential Equations

$$\frac{\mathrm{d}Cu_{A,o}}{\mathrm{d}t} = 4f_3 - 4f_1 \tag{12.1}$$

$$\frac{\mathrm{d}a_{3,r}}{\mathrm{d}t} = 4f_3 - 4f_3 \tag{12.2}$$

$$\frac{\mathrm{d}\psi}{\mathrm{d}t} = \frac{p_3\,f_3 + p_1\,f_1 + p_3\,f_3 - L}{C_{im}} \tag{12.3}$$

$$\frac{\mathrm{d}H^+}{\mathrm{d}t} = \frac{1}{R_{Hi}}\,L - \frac{p_3}{R_{Hi}}\,f_3 - \frac{p_1}{R_{Hi}}\,f_1 - \frac{p_3}{R_{Hi}}\,f_3 \tag{12.4}$$

$$\frac{\mathrm{d}O_2}{\mathrm{d}t} = \frac{1}{Vol_{mit}}\,J_{O_2} - f_3 \tag{12.5}$$

$$\frac{\mathrm{d}v_{CO_2}}{\mathrm{d}t} = \frac{1}{\tau_{CO_2}}\,\left(Pa_{CO_2} - v_{CO_2}\right) \tag{12.6}$$

$$\frac{\mathrm{d}v_{O_2}}{\mathrm{d}t} = \frac{1}{\tau_{O_2}}\,\left(O_{2,c} - v_{O_2}\right) \tag{12.7}$$

$$\frac{\mathrm{d}v_{P_a}}{\mathrm{d}t} = \frac{1}{\tau_{P_a}}\,\left(P_a - v_{P_a}\right) \tag{12.8}$$

$$\frac{d\nu_u}{dt} = \frac{1}{\tau_u}\,(u - \nu_u) \tag{12.9}$$

12.3 Algebraic Equations

$$\phi\left(\frac{S_{c,O_2}}{1 - S_{c,O_2}}\right)^{\frac{1}{n_h}} - O_{2,c} = 0 \tag{12.10}$$

$$\lambda_0 + \frac{\lambda_{P_a}}{P_a} + \lambda_\mu\,\mu - r = 0 \tag{12.11}$$

$$CBF\,(HbO_{2,a} - HbO_{2,v}) - J_{O_2} = 0 \tag{12.12}$$

12.4 Chemical Reactions

$$\xrightarrow{L} \frac{1}{R_{Hi}}\,\mathrm{H}^+ \tag{12.13}$$

$$\xrightarrow{J_{O_2}} \frac{1}{Vol_{mit}}\,\mathrm{O}_2 \tag{12.14}$$

$$\frac{p_3}{R_{Hi}}\,\mathrm{H}^+ \xrightarrow{f_3} 4\,\mathrm{Cu}_{\mathrm{A,o}} + 4\,\mathrm{a}_{3,\mathrm{r}} \tag{12.15}$$

$$4\,\mathrm{Cu}_{\mathrm{A,o}} + \frac{p_1}{R_{Hi}}\,\mathrm{H}^+ \xrightarrow{f_1} \tag{12.16}$$

$$\mathrm{O}_2 + 4\,\mathrm{a}_{3,\mathrm{r}} + \frac{p_3}{R_{Hi}}\,\mathrm{H}^+ \xrightarrow{f_3} \tag{12.17}$$

12.5 State Variables

$Cu_{A,o}$
: Implementation Name: `a`
  Units: mM
  Initial value: $Cu_{A,o,n}$
  Concentration of oxidised cytochrome c oxidase.

$a_{3,r}$
: Implementation Name: `bred`
  Units: mM
  Initial value: $a_{3,r,n}$
  Concentration of reduced cytochrome a_3.

ψ
Implementation Name: `Dpsi`
Units: mV
Initial value: ψ_n
Mitochondrial inner membrane potential. Varies as charge (in the form of protons) is transferred across the membrane capacitance.

H^+
Implementation Name: `H`
Units: mM
Initial value: H_n^+
Mitochondrial proton concentration.

O_2
Implementation Name: `O2`
Units: mM
Initial value: $O_{2,n}$
Mitochondrial oxygen concentration.

$O_{2,c}$
Implementation Name: `O2c`
Units: mM
Initial value: $O_{2,c,n}$
Capillary oxygen concentration.

r
Implementation Name: `r`
Units: cm
Initial value: r_n
Typical blood vessel radius.

ν_{CO_2}
Implementation Name: `v_c`
Units: mmHg
Initial value: $\nu_{CO_2,n}$
Filtered carbon dioxide partial pressure.

ν_{O_2}
Implementation Name: `v_o`
Units: mM
Initial value: $\nu_{O_2,n}$
Filtered capillary oxygen concentration.

ν_{P_a}
Implementation Name: `v_p`
Units: mmHg
Initial value: $\nu_{P_a,n}$
Filtered arterial blood pressure.

ν_u
Implementation Name: `v_u`
Units: dimensionless
Initial value: $\nu_{u,n}$
Filtered demand.

$HbO_{2,v}$
Implementation Name: `XOv`
Units: mM
Initial value: $HbO_{2,v,n}$
Venous concentration of oxygen bound to haemoglobin.

12.6 Intermediate Variables

$CBF = G\ (P_a - P_v)$
Implementation Name: `CBF`
Units: $ml_{blood}\ ml_{brain}^{-1}\ s^{-1}$
Initial value: CBF_n
Cerebral blood flow.

$\Delta oxCCO = \Delta oxCCO_{off} + 1000 Vol_{mit}\ (Cu_{A,o} - Cu_{A,o,n})$
Implementation Name: `CCO`
Units: uM
Initial value: 0
Cytochrome c oxidase signal measured by NIRS.

$CMRO_2 = f_3\, Vol_{mit}$
Implementation Name: `CMRO2`
Units: mM s^{-1}
Initial value: 0
Rate of cerebral oxygen metabolism.

$\Delta p = \psi - Z\ \left(4 + \log 10\left(H^+\right)\right)$
Implementation Name: `Dp`
Units: mV
Initial value: 0
Proton motive force across the mitochondrial inner membrane.

$\eta = R_{P_a}\left(\frac{\nu_{P_a}}{\nu_{P_a,n}} - 1\right) + R_{O_2}\left(\frac{\nu_{O_2}}{\nu_{O_2,n}} - 1\right) + R_{CO_2}\left(1 - \frac{\nu_{CO_2}}{\nu_{CO_2,n}}\right) + R_u\left(1 - \frac{\nu_u}{\nu_{u,n}}\right)$
Implementation Name: `eta`
Units: dimensionless
Initial value: 0
Merged autoregulation stimulus.

$f_1 = \lambda_{f_1} + \lambda_{f_1,u} \log(u) + \lambda_{f_1,a} \log(Cu_{A,o})$
Implementation Name: `f1`
Units: mM s^{-1}
Initial value: 0
Reaction rate for the reduction of Cu_A.

$f_3 = \lambda_{f_2} + \lambda_{f_2,b} \log(a_{3,r})$
Implementation Name: `f2`
Units: mM s^{-1}
Initial value: 0
Reaction rate for the reduction of a_3.

$f_3 = \lambda_{f_3} + \lambda_{f_3,O} \log(O_2)$
Implementation Name: `f3`
Units: mM s^{-1}
Initial value: 0
Reaction rate for the reduction of O_2.

$G = K_G\, r^4$
Implementation Name: `G`
Units: $ml_{blood}\ ml_{brain}^{-1}\ mmHg^{-1}\ s^{-1}$
Initial value: 0
Effective conductance of the whole blood flow compartment.

$HbO_2 = (V_a\, HbO_{2,a} + V_v\, HbO_{2,v})\ blood_{hb}$
Implementation Name: `HbO2`
Units: uM

Initial value: 0
Oxygenated haemoglobin signal measured by NIRS.

$HbT = (V_a + V_v)\ Hb_{tot}\ blood_{hb}$
Implementation Name: `HbT`
Units: uM
Initial value: 0
Total haemoglobin signal measured by NIRS.

$HHb = HbT - HbO_2$
Implementation Name: `HHb`
Units: uM
Initial value: 0
Deoxygenated haemoglobin signal measured by NIRS.

$J_{O_2} = \text{fmin}\left(D_{O_2}\ (O_{2,c} - O_2)\,, CBF\ HbO_{2,a}\right)$
Implementation Name: `J_O2`
Units: mM s^{-1}
Initial value: 0
Oxygen flux from blood to tissue.

$L = \lambda_L + \lambda_{L,\theta}\,\theta + \lambda_{L,p}\,\Delta p$
Implementation Name: `L`
Units: mM s^{-1}
Initial value: 0
Rate of proton return to the mitochondrial matrix.

$\mu = \dfrac{k_{aut}\ (\exp(\eta) - 1)}{\exp(\eta) + 1}$
Implementation Name: `mu`
Units: dimensionless
Initial value: 0
Effective strength of the autoregulation reponse.

$R_{Hi} = \dfrac{R_{Hi,H}}{H^+}$
Implementation Name: `R_Hi`
Units: dimensionless
Initial value: 0
Relative mitochondrial volume for protons, taking into account buffering effect of pH.

$S_{c,O_2} = \dfrac{S_{a,O_2} + S_{v,O_2}}{2}$
Implementation Name: `ScO2`
Units: dimensionless
Initial value: $S_{c,O_2,n}$
Capillary oxygen saturation.

$S_{v,O_2} = \dfrac{HbO_{2,v}}{Hb_{tot}}$
Implementation Name: `SvO2`
Units: dimensionless
Initial value: $S_{v,O_2,n}$
Venous oxygen saturation.

$\theta = kCV\ (\Delta p + Z \log10(u) - 90)$
Implementation Name: `theta`
Units: dimensionless
Initial value: 0
Driving force Complex V.

$TOI = \frac{100HbO_2}{HbT}$
Implementation Name: `TOI`
Units: dimensionless
Initial value: 0
Total oxygenation index.

$V_{mca} = CBF\, CBFscale$
Implementation Name: `Vmca`
Units: cm s^{-1}
Initial value: 0
Blood velocity in the middle cerebral artery.

$V_a = V_{a,n} \left(\frac{r}{r_n}\right)^2$
Implementation Name: `Vol_art`
Units: dimensionless
Initial value: 0
Relative arterial blood volume.

12.7 Parameters

$Cu_{A,o,n}$
Implementation Name: `a_n`
Units: mM
Initial value: 0.06567
Normal concentration of oxidised cytochrome c oxidase.

$blood_{hb}$
Implementation Name: `blood_hb`
Units: dimensionless
Initial value: 10.00
Factor to convert model haemoglobin concentration to instrumental units. Scales for blood fraction of brain volume, mM to μM, and number of binding sites.

$a_{3,r,n}$
Implementation Name: `bred_n`
Units: mM
Initial value: 0.001408
Normal concentration of reduced cytochrome a3.

C_{im}
Implementation Name: `C_im`
Units: mM mV^{-1}
Initial value: 0.00675
Capacitance of the mitochondrial inner membrane.

CBF_n
Implementation Name: `CBFn`
Units: $ml_{blood}\ ml_{brain}^{-1}\ s^{-1}$
Initial value: 0.0125
Normal cerebral blood flow.

$CBFscale$
Implementation Name: `CBFscale`
Units: cm
Initial value: 5000
Scale constant relating blood flow to arterial velocity.

$\Delta oxCCO_{off}$
Implementation Name: CCO_offset
Units: uM
Initial value: 0
Signal offset for the NIRS CCO measurement.

$CMRO_{2,n}$
Implementation Name: CMRO2_n
Units: mM s^{-1}
Initial value: 0.034
Normal metabolic rate of oxygen consumption.

D_{O_2}
Implementation Name: D_O2
Units: s^{-1}
Initial value: $\frac{J_{O_{2,n}}}{O_{2,c,n} - O_{2,n}}$
Diffusion rate for oxygen between capillaries and mitochondria.

ψ_n
Implementation Name: Dpsi_n
Units: mV
Initial value: 145
Normal mitochondrial inner membrane potential.

λ_{f_1}
Implementation Name: f1_0
Units: mM s^{-1}
Initial value: 1.490
Fitted intercept for the linear model for f_1.

$\lambda_{f_1,a}$
Implementation Name: f1_a
Units: mM s^{-1}
Initial value: 0.3609
Fitted linear dependence of f_1 on logarithm of $Cu_{A,ox}$.

$\lambda_{f_1,u}$
Implementation Name: f1_u
Units: mM s^{-1}
Initial value: 0.06985
Fitted linear dependence of f_1 on logarithm of demand.

λ_{f_2}
Implementation Name: f2_0
Units: mM s^{-1}
Initial value: 0.1473
Fitted intercept for the linear model for f_2.

$\lambda_{f_2,b}$
Implementation Name: f2_bred
Units: mM s^{-1}
Initial value: -0.05484
Fitted linear dependence of f_2 on logarithm of $a_{3,red}$.

λ_{f_3}
Implementation Name: f3_0
Units: mM s^{-1}
Initial value: 0.6324
Fitted intercept for the linear model for f_3.

$\lambda_{f_3,O}$
Implementation Name: `f3_O2`
Units: mM s^{-1}
Initial value: 0.03352
Fitted linear dependence of f_3 on logarithm of O_2.

G_n
Implementation Name: `Gn`
Units: ml_{blood} ml_{brain}^{-1} $mmHg^{-1}$ s^{-1}
Initial value: $\frac{CBF_n}{P_{a,n} - P_{v,n}}$
Normal blood vessel conductance.

H_n^+
Implementation Name: `H_n`
Units: mM
Initial value: 0.00003981
Normal mitochondrial proton concentration.

$J_{O_{2,n}}$
Implementation Name: `J_O2n`
Units: mM s^{-1}
Initial value: $CMRO_{2,n}$
Normal oxygen flux from blood to tissue.

k_{aut}
Implementation Name: `k_aut`
Units: dimensionless
Initial value: 1
Overall functioning of autoregulatory response.

K_G
Implementation Name: `K_G`
Units: ml_{blood} ml_{brain}^{-1} $mmHg^{-1}$ s^{-1} cm^{-4}
Initial value: $\frac{G_n}{\text{pow}\,(r_n, 4)}$
Proportionality constant in Poiseuille relation for conductance.

kCV
Implementation Name: `kCV`
Units: mV^{-1}
Initial value: 0.02047339
Factor relating the Complex V driving force to the membrane potential and demand.

λ_L
Implementation Name: `L_0`
Units: mM s^{-1}
Initial value: -15.339464
Fitted intercept for the linear model for L.

$\lambda_{L,p}$
Implementation Name: `L_Dp`
Units: mM s^{-1} mV^{-1}
Initial value: 0.097097
Fitted linear dependence of L on Δp.

$\lambda_{L,\theta}$
Implementation Name: `L_th`
Units: mM s^{-1}

Initial value: 5.665904
Fitted linear dependence of L on θ.

λ_0
Implementation Name: lam_0
Units: cm
Initial value: 0.02327
Intercept of the fitted linear model for blood vessel radius.

λ_μ
Implementation Name: lam_mu
Units: cm
Initial value: -0.006375
Fitted linear dependence of blood vessel radius on autoregulatory stimuli.

λ_{P_a}
Implementation Name: lam_p
Units: cm mmHg
Initial value: -0.4697
Fitted linear dependence of blood vessel radius on reciprocal of blood pressure.

n_h
Implementation Name: n_h
Units: dimensionless
Initial value: 2.5
Hill coefficient for oxygen dissociation from haemoglobin.

$O_{2,n}$
Implementation Name: O2_n
Units: mM
Initial value: 0.024
Normal mitochondrial oxygen concentration.

$O_{2,c,n}$
Implementation Name: O2c_n
Units: mM
Initial value: $\phi \operatorname{pow}\left(\frac{S_{c,O_2,n}}{1 - S_{c,O_2,n}}, \frac{1}{n_h}\right)$
Normal capillary oxygen concentration.

p_1
Implementation Name: p1
Units: dimensionless
Initial value: 12
Proton cost of the reaction reducing Cu_A.

p_3
Implementation Name: p2
Units: dimensionless
Initial value: 4
Proton cost of the reaction reducing a_3.

p_3
Implementation Name: p3
Units: dimensionless
Initial value: 4
Proton cost of the reaction reducing O_2.

P_a
Implementation Name: P_a
Units: mmHg

Initial value: $P_{a,n}$
Mean arterial blood pressure.

$P_{a,n}$
Implementation Name: P_an
Units: mmHg
Initial value: 100
Normal arterial blood pressure.

P_v
Implementation Name: P_v
Units: mmHg
Initial value: $P_{v,n}$
Venous blood pressure.

$P_{v,n}$
Implementation Name: P_vn
Units: mmHg
Initial value: 4
Normal venous blood pressure.

Pa_{CO_2}
Implementation Name: Pa_CO2
Units: mmHg
Initial value: $Pa_{CO_{2,n}}$
Arterial partial pressure of carbon dioxide.

$Pa_{CO_{2,n}}$
Implementation Name: Pa_CO2n
Units: mmHg
Initial value: 40
Normal arterial partial pressure of carbon dioxide.

ϕ
Implementation Name: phi
Units: mM
Initial value: 0.036
Oxygen concentration at half-maximal saturation.

R_{CO_2}
Implementation Name: R_autc
Units: dimensionless
Initial value: 2.2
Autoregulatory reactivity to carbon dioxide.

R_{O_2}
Implementation Name: R_auto
Units: dimensionless
Initial value: 1.5
Autoregulatory reactivity to oxygen.

R_{P_a}
Implementation Name: R_autp
Units: dimensionless
Initial value: 4
Autoregulatory reactivity to blood pressure.

R_u
Implementation Name: R_autu
Units: dimensionless

Initial value: 0.5
Autoregulatory reactivity to demand.

$R_{Hi,H}$
Implementation Name: R_Hi_H
Units: mM
Initial value: 9.565483
Proton buffering factor.

r_n
Implementation Name: r_n
Units: cm
Initial value: 0.0187
Normal blood vessel radius. Normal effective blood vessel radius.

$S_{a,O_2,n}$
Implementation Name: SaO2_n
Units: dimensionless
Initial value: 0.96
Normal arterial oxygen saturation.

S_{a,O_2}
Implementation Name: SaO2sup
Units: dimensionless
Initial value: $S_{a,O_2,n}$
Arterial oxygen saturation.

$S_{c,O_2,n}$
Implementation Name: ScO2_n
Units: dimensionless
Initial value: $\frac{S_{a,O_2,n} + S_{v,O_2,n}}{2}$
Normal capillary oxygen saturation.

$S_{v,O_2,n}$
Implementation Name: SvO2_n
Units: dimensionless
Initial value: $\frac{HbO_{2,v,n}}{Hb_{tot,n}}$
Normal venous oxygen saturation.

t
Implementation Name: t
Units: s
Initial value: 0
Time over which the system evolves.

τ_{CO_2}
Implementation Name: t_c
Units: s
Initial value: 5
Filter time constant for stimulus effect of carbon dioxide.

τ_{O_2}
Implementation Name: t_o
Units: s
Initial value: 20
Filter time constant for stimulus effect of capillary oxygen.

τ_{P_a}
Implementation Name: t_p
Units: s

Initial value: 5
Filter time constant for stimulus effect of blood pressure.

τ_u

Implementation Name: `t_u`
Units: s
Initial value: 0.5
Filter time constant for stimulus effect of demand.

u

Implementation Name: `u`
Units: dimensionless
Initial value: u_n
Parameter indicating metabolic demand.

u_n

Implementation Name: `u_n`
Units: dimensionless
Initial value: 1
Normal demand.

$v_{CO_2,n}$

Implementation Name: `v_cn`
Units: mmHg
Initial value: $Pa_{CO_{2,n}}$
Normal filtered carbon dioxide partial pressure. Normal filtered carbon dioxide partial pressure.

$v_{O_2,n}$

Implementation Name: `v_on`
Units: mM
Initial value: $O_{2,c,n}$
Normal filtered capillary oxygen concentration. Normal filtered capillary oxygen concentration.

$v_{P_a,n}$

Implementation Name: `v_pn`
Units: mmHg
Initial value: $P_{a,n}$
Normal filtered arterial blood pressure. Normal filtered blood pressure.

$v_{u,n}$

Implementation Name: `v_un`
Units: dimensionless
Initial value: u_n
Normal filtered demand. Normal filtered demand.

$VArat_n$

Implementation Name: `VArat_n`
Units: dimensionless
Initial value: 3
Normal volume ratio of veins to arteries in brain tissue.

$V_{a,n}$

Implementation Name: `Vol_artn`
Units: dimensionless
Initial value: $\frac{1}{1 + VArat_n}$
Normal relative arterial blood volume.

Vol_{mit}

Implementation Name: `Vol_mit`
Units: dimensionless
Initial value: 0.067
Fraction of brain tissue volume that is mitochondria.

V_v

Implementation Name: `Vol_ven`
Units: dimensionless
Initial value: $\frac{VArat_n}{1 + VArat_n}$
Relative venous blood volume.

$HbO_{2,a} = Hb_{tot}\, S_{a,O_2}$

Implementation Name: `XOa`
Units: mM
Initial value: $HbO_{2,a,n}$
Arterial concentration of oxygen bound to haemoglobin.

$HbO_{2,a,n}$

Implementation Name: `XOa_n`
Units: mM
Initial value: $Hb_{tot,n}\, S_{a,O_2,n}$
Normal arterial concentration of oxygen bound to haemoglobin.

$HbO_{2,v,n}$

Implementation Name: `XOv_n`
Units: mM
Initial value: $\frac{CBF_n\, HbO_{2,a,n} - J_{O_{2,n}}}{CBF_n}$
Normal venous concentration of oxygen bound to haemoglobin.

Hb_{tot}

Implementation Name: `Xtot`
Units: mM
Initial value: 9.1
Total concentration of haemoglobin O_2 binding sites in blood (4 times haemoglobin concentration).

$Hb_{tot,n}$

Implementation Name: `Xtot_n`
Units: mM
Initial value: 9.1
Normal total concentration of haemoglobin O_2 binding sites in blood (4 times haemoglobin concentration).

Z

Implementation Name: `Z`
Units: mV
Initial value: 59.028
Proportionality constant in calculation of driving forces due to concentration differences. Defined as RT/F, where F is Faraday's constant, R the ideal gas constant and T the absolute temperature.

13 B2M2

13.1 Overview

Simplified model combining blood flow variant B2 and metabolic variant M2.

- 9 differential state variables
- 3 algebraic state variables
- 22 intermediate variables
- 72 parameters
- 4 declared inputs
- 33 default outputs

13.2 Differential Equations

$$\frac{\mathrm{d}Cu_{A,o}}{\mathrm{d}t} = 4f_3 - 4f_1 \tag{13.1}$$

$$\frac{\mathrm{d}a_{3,r}}{\mathrm{d}t} = 4f_3 - 4f_3 \tag{13.2}$$

$$\frac{\mathrm{d}\psi}{\mathrm{d}t} = \frac{p_3\,f_3 + p_1\,f_1 + p_3\,f_3 - L}{C_{im}} \tag{13.3}$$

$$\frac{\mathrm{d}H^+}{\mathrm{d}t} = \frac{1}{R_{Hi}}\,L - \frac{p_3}{R_{Hi}}\,f_3 - \frac{p_1}{R_{Hi}}\,f_1 - \frac{p_3}{R_{Hi}}\,f_3 \tag{13.4}$$

$$\frac{\mathrm{d}O_2}{\mathrm{d}t} = \frac{1}{Vol_{mit}}\,J_{O_2} - f_3 \tag{13.5}$$

$$\frac{\mathrm{d}\nu_{CO_2}}{\mathrm{d}t} = \frac{1}{\tau_{CO_2}}\,\left(Pa_{CO_2} - \nu_{CO_2}\right) \tag{13.6}$$

$$\frac{\mathrm{d}\nu_{O_2}}{\mathrm{d}t} = \frac{1}{\tau_{O_2}}\,\left(O_{2,c} - \nu_{O_2}\right) \tag{13.7}$$

$$\frac{\mathrm{d}\nu_{P_a}}{\mathrm{d}t} = \frac{1}{\tau_{P_a}}\,\left(P_a - \nu_{P_a}\right) \tag{13.8}$$

$$\frac{\mathrm{d}\nu_u}{\mathrm{d}t} = \frac{1}{\tau_u}\ (u - \nu_u) \tag{13.9}$$

13.3 Algebraic Equations

$$\phi\ \left(\frac{S_{c,O_2}}{1 - S_{c,O_2}}\right)^{\frac{1}{n_h}} - O_{2,c} = 0 \tag{13.10}$$

$$\lambda_0 + \frac{\lambda_{P_a}}{P_a} + \lambda_\mu\, \mu - r = 0 \tag{13.11}$$

$$CBF\ (HbO_{2,a} - HbO_{2,v}) - J_{O_2} = 0 \tag{13.12}$$

13.4 Chemical Reactions

$$\xrightarrow{L} \frac{1}{R_{Hi}}\ \mathrm{H^+} \tag{13.13}$$

$$\xrightarrow{J_{O_2}} \frac{1}{Vol_{mit}}\ \mathrm{O_2} \tag{13.14}$$

$$\frac{p_3}{R_{Hi}}\ \mathrm{H^+} \xrightarrow{f_3} 4\ \mathrm{Cu_{A,o}} + 4\ \mathrm{a_{3,r}} \tag{13.15}$$

$$4\ \mathrm{Cu_{A,o}} + \frac{p_1}{R_{Hi}}\ \mathrm{H^+} \xrightarrow{f_1} \tag{13.16}$$

$$\mathrm{O_2} + 4\ \mathrm{a_{3,r}} + \frac{p_3}{R_{Hi}}\ \mathrm{H^+} \xrightarrow{f_3} \tag{13.17}$$

13.5 State Variables

$Cu_{A,o}$
: Implementation Name: `a`
Units: mM
Initial value: $Cu_{A,o,n}$
Concentration of oxidised cytochrome c oxidase.

$a_{3,r}$
: Implementation Name: `bred`
Units: mM
Initial value: $a_{3,r,n}$
Concentration of reduced cytochrome $\mathrm{a_3}$.

ψ
Implementation Name: `Dpsi`
Units: mV
Initial value: ψ_n
Mitochondrial inner membrane potential. Varies as charge (in the form of protons) is transferred across the membrane capacitance.

H^+
Implementation Name: `H`
Units: mM
Initial value: H_n^+
Mitochondrial proton concentration.

O_2
Implementation Name: `O2`
Units: mM
Initial value: $O_{2,n}$
Mitochondrial oxygen concentration.

$O_{2,c}$
Implementation Name: `O2c`
Units: mM
Initial value: $O_{2,c,n}$
Capillary oxygen concentration.

r
Implementation Name: `r`
Units: cm
Initial value: r_n
Typical blood vessel radius.

v_{CO_2}
Implementation Name: `v_c`
Units: mmHg
Initial value: $v_{CO_2,n}$
Filtered carbon dioxide partial pressure.

v_{O_2}
Implementation Name: `v_o`
Units: mM
Initial value: $v_{O_2,n}$
Filtered capillary oxygen concentration.

v_{P_a}
Implementation Name: `v_p`
Units: mmHg
Initial value: $v_{P_a,n}$
Filtered arterial blood pressure.

v_u
Implementation Name: `v_u`
Units: dimensionless
Initial value: $v_{u,n}$
Filtered demand.

$HbO_{2,v}$
Implementation Name: `XOv`
Units: mM
Initial value: $HbO_{2,v,n}$
Venous concentration of oxygen bound to haemoglobin.

13.6 Intermediate Variables

$CBF = G\ (P_a - P_v)$
Implementation Name: `CBF`
Units: $\mathrm{ml}_{blood}\ \mathrm{ml}_{brain}^{-1}\ \mathrm{s}^{-1}$
Initial value: CBF_n
Cerebral blood flow.

$\Delta oxCCO = \Delta oxCCO_{off} + 1000 Vol_{mit}\ (Cu_{A,o} - Cu_{A,o,n})$
Implementation Name: `CCO`
Units: uM
Initial value: 0
Cytochrome c oxidase signal measured by NIRS.

$CMRO_2 = f_3\, Vol_{mit}$
Implementation Name: `CMRO2`
Units: $\mathrm{mM\ s}^{-1}$
Initial value: 0
Rate of cerebral oxygen metabolism.

$\Delta p = \psi - Z\ (4 + \log 10\ (H^+))$
Implementation Name: `Dp`
Units: mV
Initial value: 0
Proton motive force across the mitochondrial inner membrane.

$\eta = R_{P_a}\left(\frac{\nu_{P_a}}{\nu_{P_a,n}} - 1\right) + R_{O_2}\left(\frac{\nu_{O_2}}{\nu_{O_2,n}} - 1\right) + R_{CO_2}\left(1 - \frac{\nu_{CO_2}}{\nu_{CO_2,n}}\right) + R_u\left(1 - \frac{\nu_u}{\nu_{u,n}}\right)$
Implementation Name: `eta`
Units: dimensionless
Initial value: 0
Merged autoregulation stimulus.

$f_1 = \lambda_{f_1} + \lambda_{f_1,a} \log\ (Cu_{A,o})$
Implementation Name: `f1`
Units: $\mathrm{mM\ s}^{-1}$
Initial value: 0
Reaction rate for the reduction of Cu_A.

$f_3 = \lambda_{f_2} + \lambda_{f_2,b} \log\ (a_{3,r})$
Implementation Name: `f2`
Units: $\mathrm{mM\ s}^{-1}$
Initial value: 0
Reaction rate for the reduction of a_3.

$f_3 = \lambda_{f_3} + \lambda_{f_3,O} \log\ (O_2)$
Implementation Name: `f3`
Units: $\mathrm{mM\ s}^{-1}$
Initial value: 0
Reaction rate for the reduction of O_2.

$G = K_G\, r^4$
Implementation Name: `G`
Units: $\mathrm{ml}_{blood}\ \mathrm{ml}_{brain}^{-1}\ \mathrm{mmHg}^{-1}\ \mathrm{s}^{-1}$
Initial value: 0
Effective conductance of the whole blood flow compartment.

$HbO_2 = (V_a\, HbO_{2,a} + V_v\, HbO_{2,v})\ blood_{hb}$
Implementation Name: `HbO2`
Units: uM

Initial value: 0
Oxygenated haemoglobin signal measured by NIRS.

$HbT = (V_a + V_v)\ Hb_{tot}\, blood_{hb}$
Implementation Name: `HbT`
Units: uM
Initial value: 0
Total haemoglobin signal measured by NIRS.

$HHb = HbT - HbO_2$
Implementation Name: `HHb`
Units: uM
Initial value: 0
Deoxygenated haemoglobin signal measured by NIRS.

$J_{O_2} = \text{fmin}\left(D_{O_2}\ (O_{2,c} - O_2)\,, CBF\, HbO_{2,a}\right)$
Implementation Name: `J_O2`
Units: mM s^{-1}
Initial value: 0
Oxygen flux from blood to tissue.

$L = \lambda_L + \lambda_{L,\theta}\, \theta + \lambda_{L,p}\, \Delta p$
Implementation Name: `L`
Units: mM s^{-1}
Initial value: 0
Rate of proton return to the mitochondrial matrix.

$\mu = \dfrac{k_{aut}\ (\exp(\eta) - 1)}{\exp(\eta) + 1}$
Implementation Name: `mu`
Units: dimensionless
Initial value: 0
Effective strength of the autoregulation reponse.

$R_{Hi} = \dfrac{R_{Hi,H}}{H^+}$
Implementation Name: `R_Hi`
Units: dimensionless
Initial value: 0
Relative mitochondrial volume for protons, taking into account buffering effect of pH.

$S_{c,O_2} = \dfrac{S_{a,O_2} + S_{v,O_2}}{2}$
Implementation Name: `ScO2`
Units: dimensionless
Initial value: $S_{c,O_2,n}$
Capillary oxygen saturation.

$S_{v,O_2} = \dfrac{HbO_{2,v}}{Hb_{tot}}$
Implementation Name: `SvO2`
Units: dimensionless
Initial value: $S_{v,O_2,n}$
Venous oxygen saturation.

$\theta = kCV\ (\Delta p + Z \log10(u) - 90)$
Implementation Name: `theta`
Units: dimensionless
Initial value: 0
Driving force Complex V.

$TOI = \frac{100HbO_2}{HbT}$
Implementation Name: `TOI`
Units: dimensionless
Initial value: 0
Total oxygenation index.

$V_{mca} = CBF\,CBFscale$
Implementation Name: `Vmca`
Units: cm s^{-1}
Initial value: 0
Blood velocity in the middle cerebral artery.

$V_a = V_{a,n}\left(\frac{r}{r_n}\right)^2$
Implementation Name: `Vol_art`
Units: dimensionless
Initial value: 0
Relative arterial blood volume.

13.7 Parameters

$Cu_{A,o,n}$
Implementation Name: `a_n`
Units: mM
Initial value: 0.06567
Normal concentration of oxidised cytochrome c oxidase.

$blood_{hb}$
Implementation Name: `blood_hb`
Units: dimensionless
Initial value: 10.00
Factor to convert model haemoglobin concentration to instrumental units. Scales for blood fraction of brain volume, mM to μM, and number of binding sites.

$a_{3,r,n}$
Implementation Name: `bred_n`
Units: mM
Initial value: 0.001408
Normal concentration of reduced cytochrome a3.

C_{im}
Implementation Name: `C_im`
Units: mM mV^{-1}
Initial value: 0.00675
Capacitance of the mitochondrial inner membrane.

CBF_n
Implementation Name: `CBFn`
Units: $ml_{blood}\ ml^{-1}_{brain}\ s^{-1}$
Initial value: 0.0125
Normal cerebral blood flow.

$CBFscale$
Implementation Name: `CBFscale`
Units: cm
Initial value: 5000
Scale constant relating blood flow to arterial velocity.

$\Delta oxCCO_{off}$
Implementation Name: `CCO_offset`
Units: uM
Initial value: 0
Signal offset for the NIRS CCO measurement.

$CMRO_{2,n}$
Implementation Name: `CMRO2_n`
Units: mM s^{-1}
Initial value: 0.034
Normal metabolic rate of oxygen consumption.

D_{O_2}
Implementation Name: `D_O2`
Units: s^{-1}
Initial value: $\frac{J_{O_{2,n}}}{O_{2,c,n} - O_{2,n}}$
Diffusion rate for oxygen between capillaries and mitochondria.

ψ_n
Implementation Name: `Dpsi_n`
Units: mV
Initial value: 145
Normal mitochondrial inner membrane potential.

λ_{f_1}
Implementation Name: `f1_0`
Units: mM s^{-1}
Initial value: 1.504
Fitted intercept for the linear model for f_1.

$\lambda_{f_1,a}$
Implementation Name: `f1_a`
Units: mM s^{-1}
Initial value: 0.3658
Fitted linear dependence of f_1 on logarithm of $\mathrm{Cu}_{A,ox}$.

λ_{f_2}
Implementation Name: `f2_0`
Units: mM s^{-1}
Initial value: 0.1473
Fitted intercept for the linear model for f_2.

$\lambda_{f_2,b}$
Implementation Name: `f2_bred`
Units: mM s^{-1}
Initial value: -0.05484
Fitted linear dependence of f_2 on logarithm of $\mathrm{a}_{3,red}$.

λ_{f_3}
Implementation Name: `f3_0`
Units: mM s^{-1}
Initial value: 0.6324
Fitted intercept for the linear model for f_3.

$\lambda_{f_3,O}$
Implementation Name: `f3_O2`
Units: mM s^{-1}
Initial value: 0.03352
Fitted linear dependence of f_3 on logarithm of O_2.

G_n

Implementation Name: `Gn`
Units: ml_{blood} ml_{brain}^{-1} $mmHg^{-1}$ s^{-1}
Initial value: $\frac{CBF_n}{P_{a,n} - P_{v,n}}$
Normal blood vessel conductance.

H_n^+

Implementation Name: `H_n`
Units: mM
Initial value: 0.00003981
Normal mitochondrial proton concentration.

$J_{O_{2,n}}$

Implementation Name: `J_O2n`
Units: mM s^{-1}
Initial value: $CMRO_{2,n}$
Normal oxygen flux from blood to tissue.

k_{aut}

Implementation Name: `k_aut`
Units: dimensionless
Initial value: 1
Overall functioning of autoregulatory response.

K_G

Implementation Name: `K_G`
Units: ml_{blood} ml_{brain}^{-1} $mmHg^{-1}$ s^{-1} cm^{-4}
Initial value: $\frac{G_n}{\mathrm{pow}\,(r_n, 4)}$
Proportionality constant in Poiseuille relation for conductance.

kCV

Implementation Name: `kCV`
Units: mV^{-1}
Initial value: 0.02047339
Factor relating the Complex V driving force to the membrane potential and demand.

λ_L

Implementation Name: `L_0`
Units: mM s^{-1}
Initial value: -15.339464
Fitted intercept for the linear model for L.

$\lambda_{L,p}$

Implementation Name: `L_Dp`
Units: mM s^{-1} mV^{-1}
Initial value: 0.097097
Fitted linear dependence of L on Δp.

$\lambda_{L,\theta}$

Implementation Name: `L_th`
Units: mM s^{-1}
Initial value: 5.665904
Fitted linear dependence of L on θ.

λ_0

Implementation Name: `lam_0`
Units: cm

Initial value: 0.02327
Intercept of the fitted linear model for blood vessel radius.

λ_{μ}
Implementation Name: `lam_mu`
Units: cm
Initial value: -0.006375
Fitted linear dependence of blood vessel radius on autoregulatory stimuli.

λ_{P_a}
Implementation Name: `lam_p`
Units: cm mmHg
Initial value: -0.4697
Fitted linear dependence of blood vessel radius on reciprocal of blood pressure.

n_h
Implementation Name: `n_h`
Units: dimensionless
Initial value: 2.5
Hill coefficient for oxygen dissociation from haemoglobin.

$O_{2,n}$
Implementation Name: `O2_n`
Units: mM
Initial value: 0.024
Normal mitochondrial oxygen concentration.

$O_{2,c,n}$
Implementation Name: `O2c_n`
Units: mM
Initial value: $\phi\,\mathrm{pow}\left(\frac{S_{c,O_2,n}}{1 - S_{c,O_2,n}}, \frac{1}{n_h}\right)$
Normal capillary oxygen concentration.

p_1
Implementation Name: `p1`
Units: dimensionless
Initial value: 12
Proton cost of the reaction reducing Cu_A.

p_3
Implementation Name: `p2`
Units: dimensionless
Initial value: 4
Proton cost of the reaction reducing a_3.

p_3
Implementation Name: `p3`
Units: dimensionless
Initial value: 4
Proton cost of the reaction reducing O_2.

P_a
Implementation Name: `P_a`
Units: mmHg
Initial value: $P_{a,n}$
Mean arterial blood pressure.

$P_{a,n}$
Implementation Name: `P_an`
Units: mmHg

Initial value: 100
Normal arterial blood pressure.

P_v
Implementation Name: P_v
Units: mmHg
Initial value: $P_{v,n}$
Venous blood pressure.

$P_{v,n}$
Implementation Name: P_vn
Units: mmHg
Initial value: 4
Normal venous blood pressure.

Pa_{CO_2}
Implementation Name: Pa_CO2
Units: mmHg
Initial value: $Pa_{CO_{2,n}}$
Arterial partial pressure of carbon dioxide.

$Pa_{CO_{2,n}}$
Implementation Name: Pa_CO2n
Units: mmHg
Initial value: 40
Normal arterial partial pressure of carbon dioxide.

ϕ
Implementation Name: phi
Units: mM
Initial value: 0.036
Oxygen concentration at half-maximal saturation.

R_{CO_2}
Implementation Name: R_autc
Units: dimensionless
Initial value: 2.2
Autoregulatory reactivity to carbon dioxide.

R_{O_2}
Implementation Name: R_auto
Units: dimensionless
Initial value: 1.5
Autoregulatory reactivity to oxygen.

R_{P_a}
Implementation Name: R_autp
Units: dimensionless
Initial value: 4
Autoregulatory reactivity to blood pressure.

R_u
Implementation Name: R_autu
Units: dimensionless
Initial value: 0.5
Autoregulatory reactivity to demand.

$R_{Hi,H}$
Implementation Name: R_Hi_H
Units: mM

Initial value: 9.565483
Proton buffering factor.

r_n
Implementation Name: r_n
Units: cm
Initial value: 0.0187
Normal blood vessel radius. Normal effective blood vessel radius.

$S_{a,O_2,n}$
Implementation Name: SaO2_n
Units: dimensionless
Initial value: 0.96
Normal arterial oxygen saturation.

S_{a,O_2}
Implementation Name: SaO2sup
Units: dimensionless
Initial value: $S_{a,O_2,n}$
Arterial oxygen saturation.

$S_{c,O_2,n}$
Implementation Name: ScO2_n
Units: dimensionless
Initial value: $\frac{S_{a,O_2,n} + S_{v,O_2,n}}{2}$
Normal capillary oxygen saturation.

$S_{v,O_2,n}$
Implementation Name: SvO2_n
Units: dimensionless
Initial value: $\frac{HbO_{2,v,n}}{Hb_{tot,n}}$
Normal venous oxygen saturation.

t
Implementation Name: t
Units: s
Initial value: 0
Time over which the system evolves.

τ_{CO_2}
Implementation Name: t_c
Units: s
Initial value: 5
Filter time constant for stimulus effect of carbon dioxide.

τ_{O_2}
Implementation Name: t_o
Units: s
Initial value: 20
Filter time constant for stimulus effect of capillary oxygen.

τ_{P_a}
Implementation Name: t_p
Units: s
Initial value: 5
Filter time constant for stimulus effect of blood pressure.

τ_u
Implementation Name: t_u
Units: s

Initial value: 0.5
Filter time constant for stimulus effect of demand.

u
Implementation Name: `u`
Units: dimensionless
Initial value: u_n
Parameter indicating metabolic demand.

u_n
Implementation Name: `u_n`
Units: dimensionless
Initial value: 1
Normal demand.

$v_{CO_2,n}$
Implementation Name: `v_cn`
Units: mmHg
Initial value: $Pa_{CO_{2,n}}$
Normal filtered carbon dioxide partial pressure. Normal filtered carbon dioxide partial pressure.

$v_{O_2,n}$
Implementation Name: `v_on`
Units: mM
Initial value: $O_{2,c,n}$
Normal filtered capillary oxygen concentration. Normal filtered capillary oxygen concentration.

$v_{P_a,n}$
Implementation Name: `v_pn`
Units: mmHg
Initial value: $P_{a,n}$
Normal filtered arterial blood pressure. Normal filtered blood pressure.

$v_{u,n}$
Implementation Name: `v_un`
Units: dimensionless
Initial value: u_n
Normal filtered demand. Normal filtered demand.

$VArat_n$
Implementation Name: `VArat_n`
Units: dimensionless
Initial value: 3
Normal volume ratio of veins to arteries in brain tissue.

$V_{a,n}$
Implementation Name: `Vol_artn`
Units: dimensionless
Initial value: $\frac{1}{1 + VArat_n}$
Normal relative arterial blood volume.

Vol_{mit}
Implementation Name: `Vol_mit`
Units: dimensionless
Initial value: 0.067
Fraction of brain tissue volume that is mitochondria.

V_v

Implementation Name: `Vol_ven`
Units: dimensionless
Initial value: $\frac{VArat_n}{1 + VArat_n}$
Relative venous blood volume.

$HbO_{2,a} = Hb_{tot}\, S_{a,O_2}$
Implementation Name: `XOa`
Units: mM
Initial value: $HbO_{2,a,n}$
Arterial concentration of oxygen bound to haemoglobin.

$HbO_{2,a,n}$
Implementation Name: `XOa_n`
Units: mM
Initial value: $Hb_{tot,n}\, S_{a,O_2,n}$
Normal arterial concentration of oxygen bound to haemoglobin.

$HbO_{2,v,n}$
Implementation Name: `XOv_n`
Units: mM
Initial value: $\frac{CBF_n\, HbO_{2,a,n} - J_{O_{2,n}}}{CBF_n}$
Normal venous concentration of oxygen bound to haemoglobin.

Hb_{tot}
Implementation Name: `Xtot`
Units: mM
Initial value: 9.1
Total concentration of haemoglobin O_2 binding sites in blood (4 times haemoglobin concentration).

$Hb_{tot,n}$
Implementation Name: `Xtot_n`
Units: mM
Initial value: 9.1
Normal total concentration of haemoglobin O_2 binding sites in blood (4 times haemoglobin concentration).

Z
Implementation Name: `Z`
Units: mV
Initial value: 59.028
Proportionality constant in calculation of driving forces due to concentration differences. Defined as RT/F, where F is Faraday's constant, R the ideal gas constant and T the absolute temperature.
