# Supplementary figures and images for: BrainSignals Revisited: Simplifying a Computational Model of Cerebral Physiology
Source: PLoS One. 2015 May 11;10(5):e0126695. doi: 10.1371/journal.pone.0126695 (PMC4427507; doi:10.1371/journal.pone.0126695)

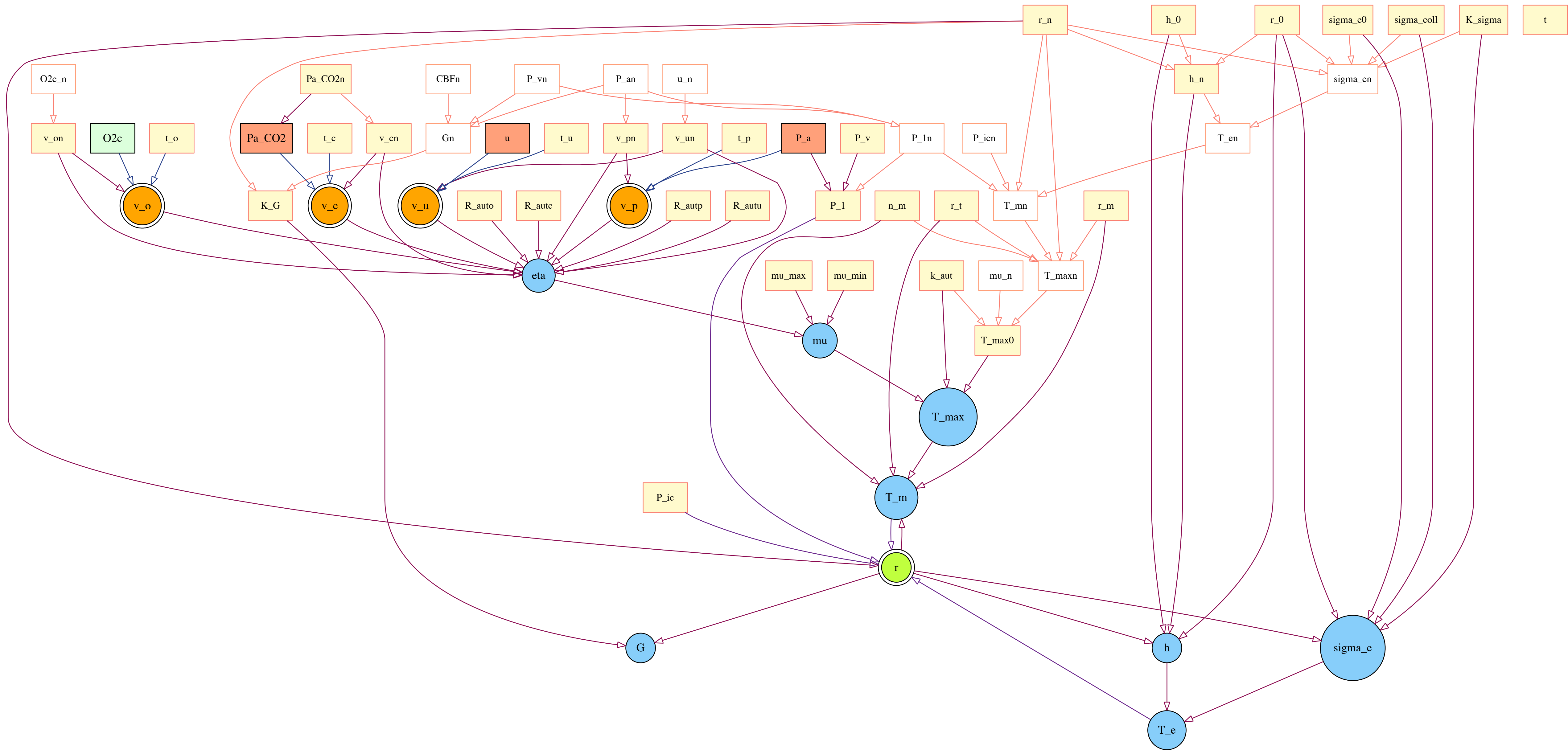

Supplement: S1 Fig — (PDF) [file pone.0126695.s002.pdf]

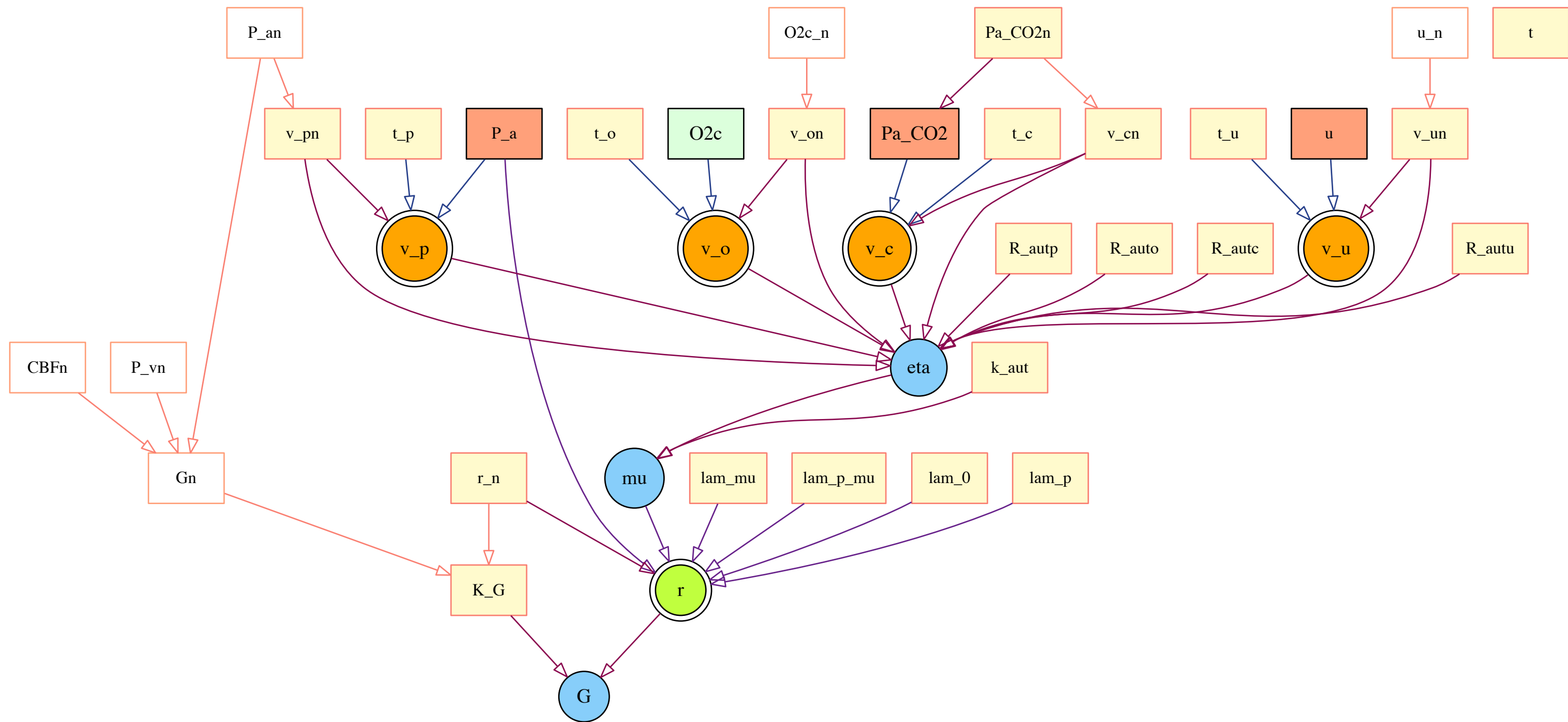

Supplement: S2 Fig — (PDF) [file pone.0126695.s003.pdf]

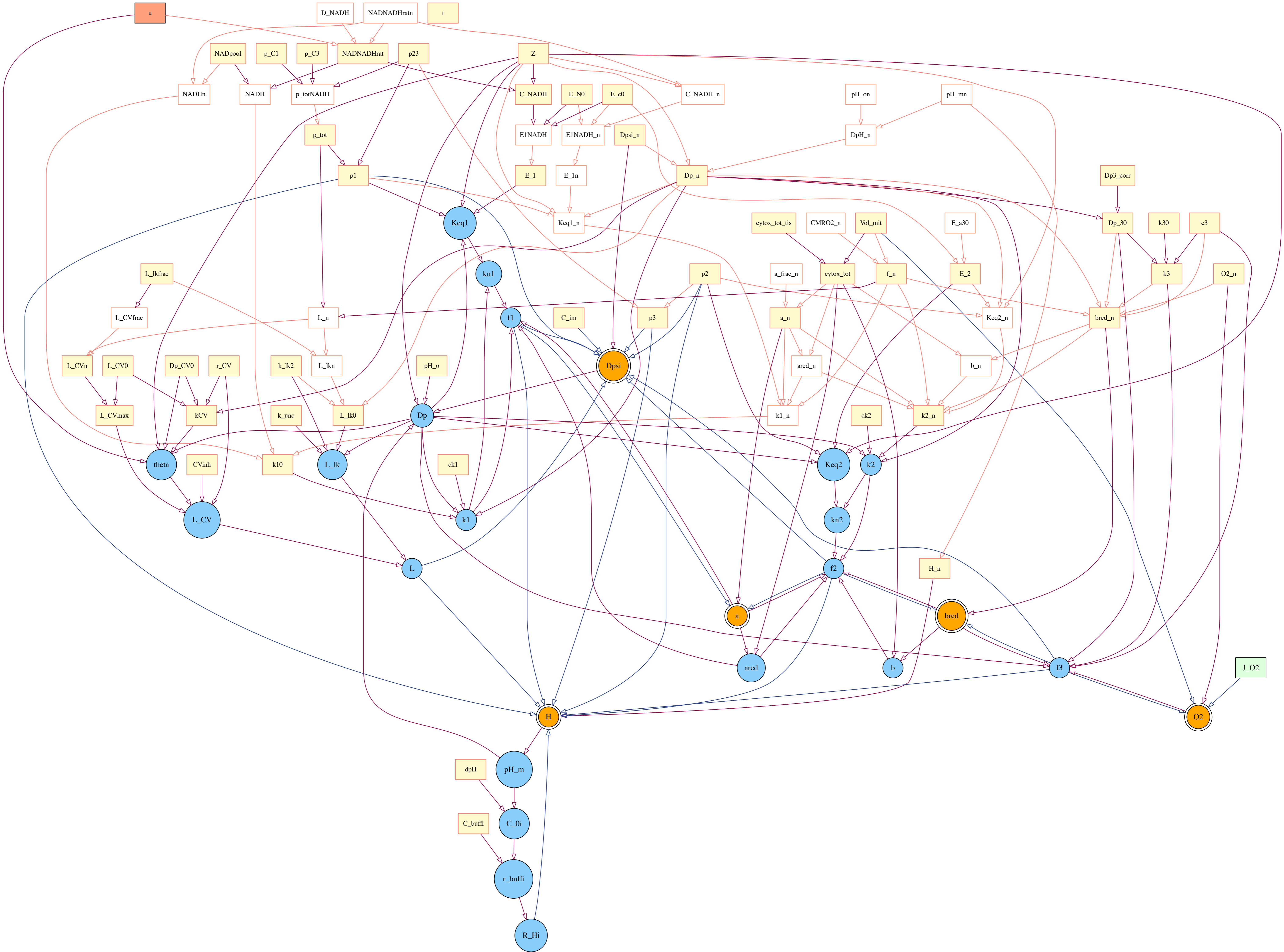

Supplement: S3 Fig — (PDF) [file pone.0126695.s004.pdf]

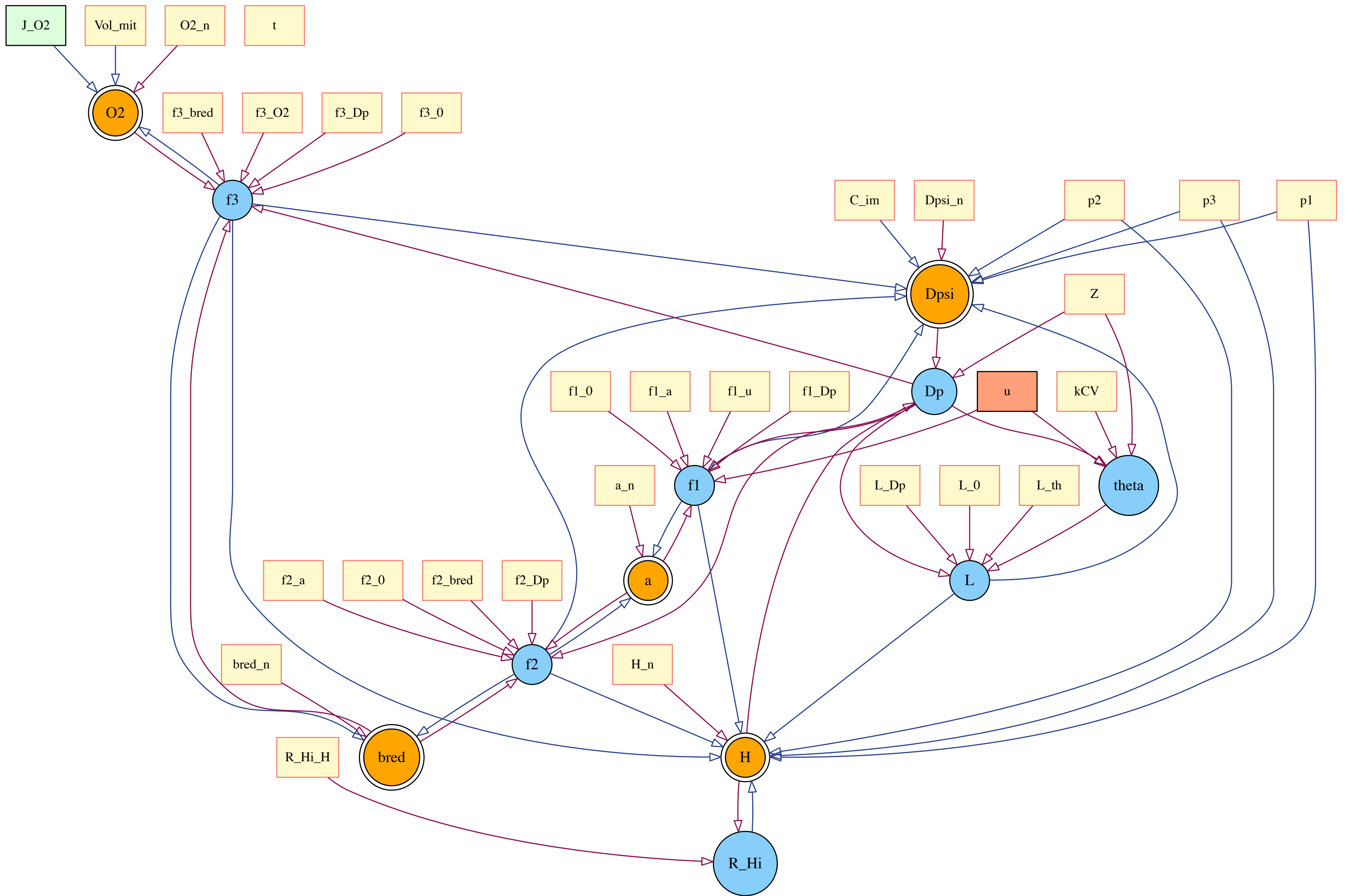

Supplement: S4 Fig — (PDF) [file pone.0126695.s005.pdf]

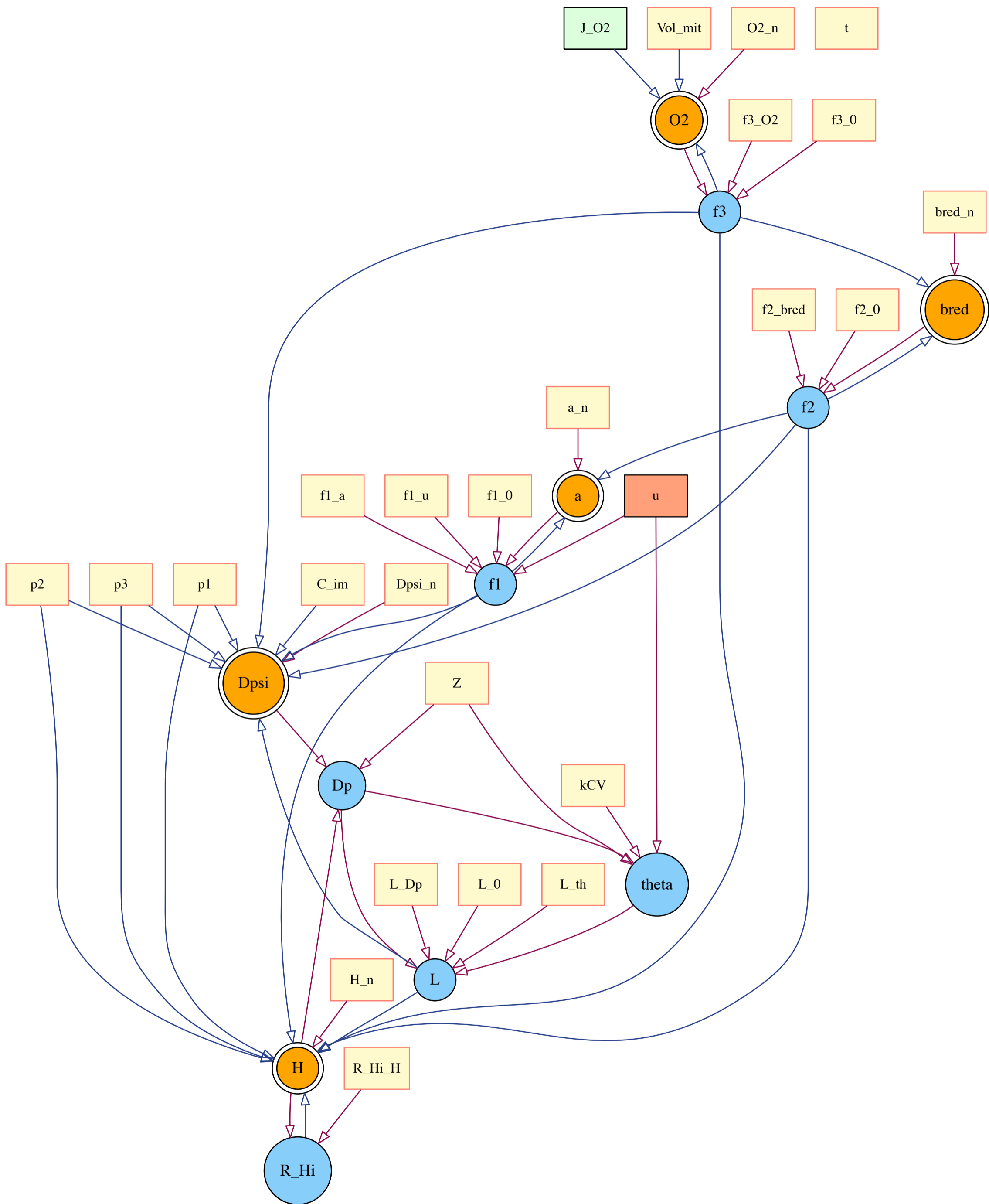

Supplement: S5 Fig — (PDF) [file pone.0126695.s006.pdf]

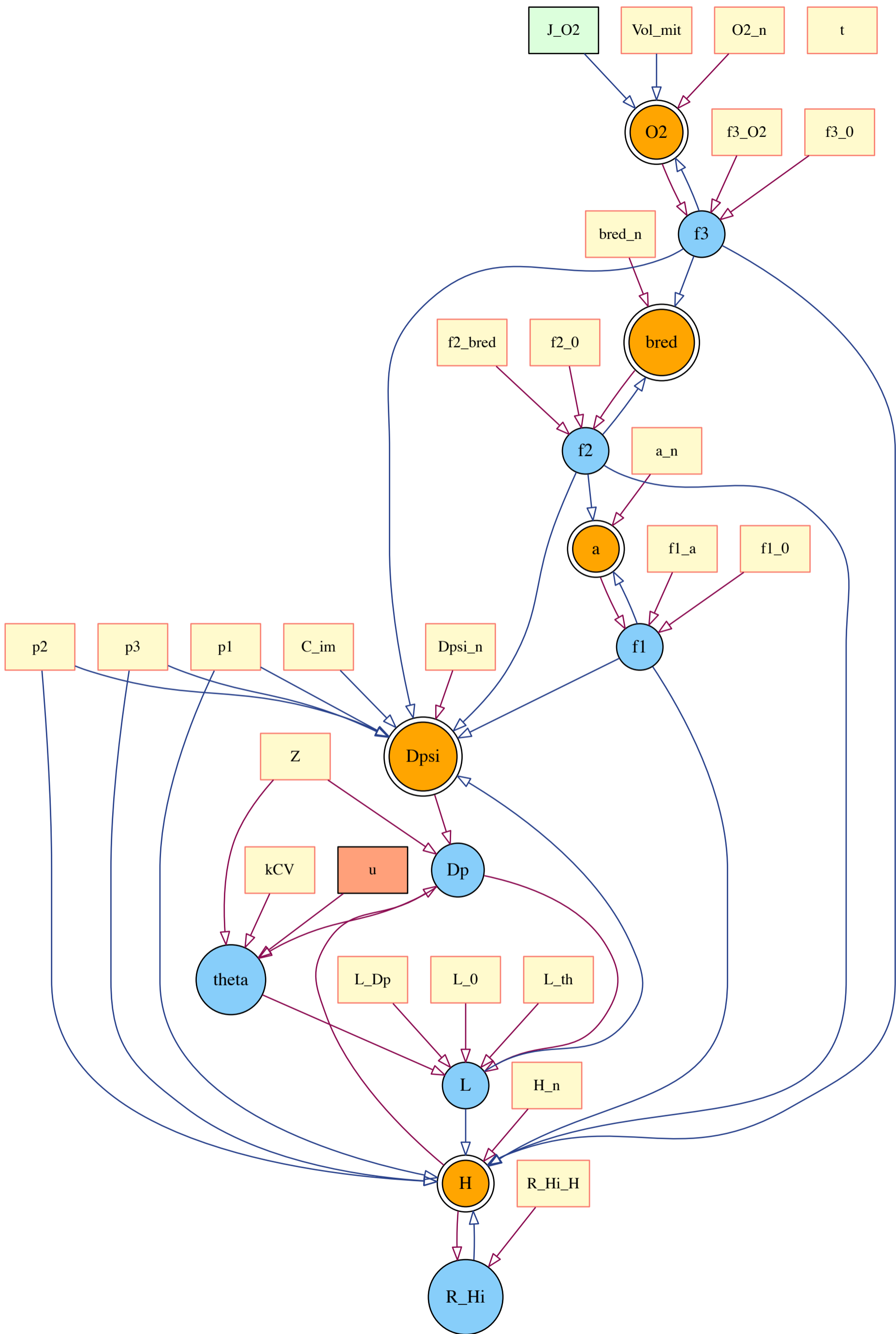

Supplement: S6 Fig — (PDF) [file pone.0126695.s007.pdf]

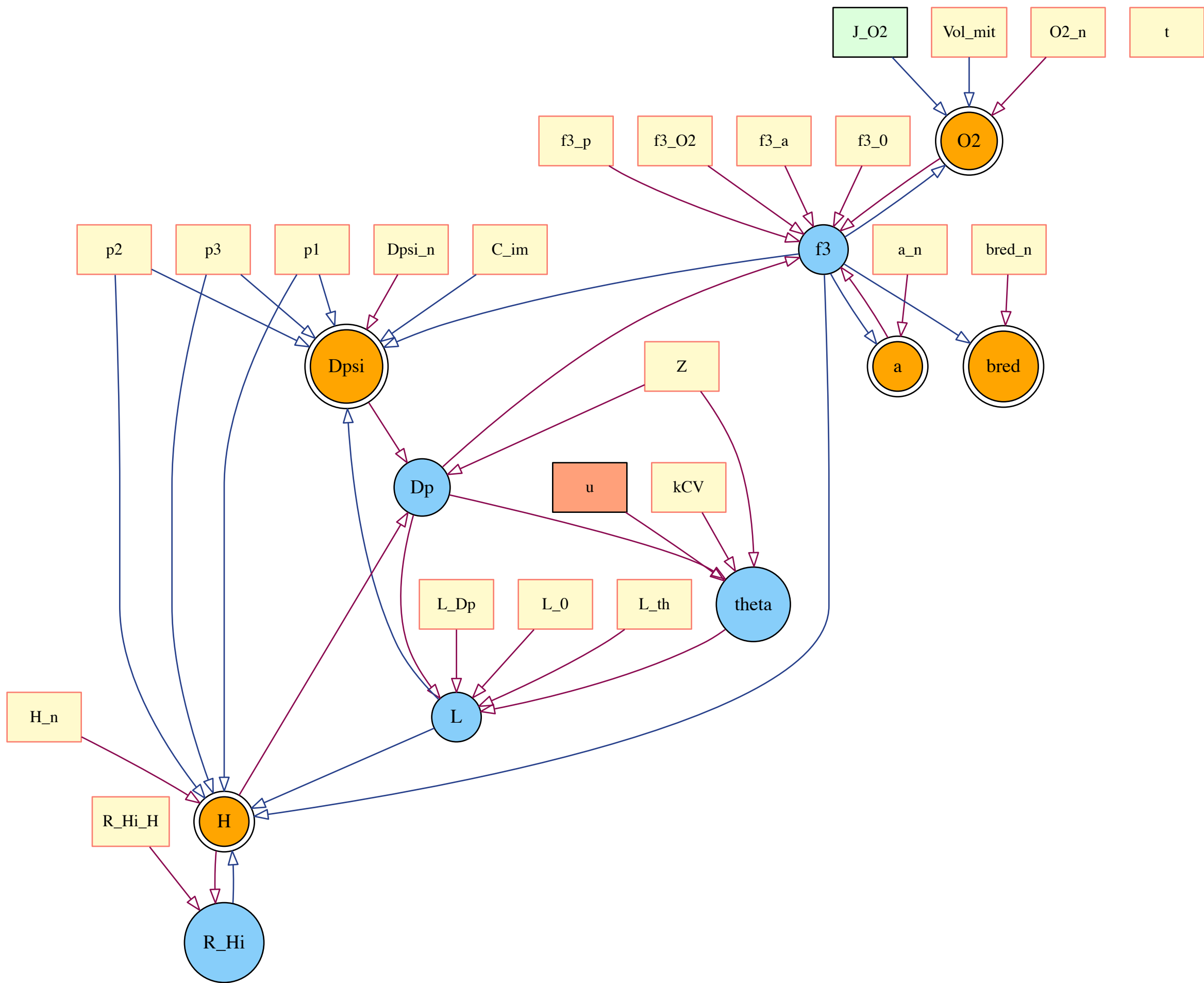

Supplement: S7 Fig — (PDF) [file pone.0126695.s008.pdf]
